# Upregulation of macrophage UPP1 promotes lung adenocarcinoma metastasis through an mtROS-cGAS-NLRP3 inflammasome axis

**DOI:** 10.1038/s41420-026-03226-4

**Published:** 2026-07-06

**Authors:** MingTao Feng, Chao Gao, YueChao Yang, Deheng Li, Changshuai Zhou, Lei Chen, Yongxiang Su, Yiqun Cao, Liangdong Li, Yang Gao

**Affiliations:** 1 Department of Neurosurgery, Fudan University Shanghai Cancer Center, Shanghai, China; 2 Department of Surgery, The First Affiliated Hospital of Xiamen University(Haicang Campus), Xiamen Cancer Hospital, Xiamen, Fujian China; 3 Department of Neurosurgery, Fudan University, Shanghai, China; 4 Department of Thoracic Surgery, The First Affiliated Hospital of Xiamen University(Haicang Campus), Xiamen Cancer Hospital, Xiamen, Fujian China

**Keywords:** Diseases, Cancer

## Abstract

Metastasis and immunosuppression remain major barriers to effective treatment of lung adenocarcinoma (LUAD), yet the metabolic mechanisms governing the pro-tumor functions of tumor-associated macrophages are incompletely understood. In this study, we identified Uridine Phosphorylase 1 (UPP1) as a macrophage-enriched metabolic regulator associated with LUAD progression. By integrating single-cell RNA sequencing with clinical cohort analyses, we found that UPP1 was preferentially expressed in tumor-associated macrophages and was associated with adverse clinical outcomes. Functional and mechanistic studies demonstrated that dysregulated UPP1 disrupted nucleotide homeostasis, leading to mitochondrial reactive oxygen species accumulation and mitochondrial DNA leakage. These mitochondrial stress signals activated the cGAS-STING pathway, which preferentially engaged NLRP3 inflammasome signaling rather than canonical antiviral responses. Consequently, macrophages underwent pyroptosis and released elevated levels of interleukin-1β (IL-1β). Through paracrine signaling, macrophage-derived IL-1β promoted epithelial-mesenchymal transition in LUAD cells and enhanced their invasive capacity in vitro. Consistent with these findings, co-injection of UPP1-overexpressing macrophages significantly increased spontaneous lung metastasis in vivo. Clinically, elevated UPP1 expression served as an independent predictor of poor survival. Furthermore, pharmacological blockade of this signaling cascade or neutralization of IL-1β attenuated macrophage-induced malignant phenotypes in tumor cells, highlighting the therapeutic relevance of this pathway. Collectively, our findings identify a macrophage-specific immunometabolic circuit in which UPP1-driven mitochondrial stress activates the mtROS–cGAS–NLRP3 axis, promoting IL-1β-dependent macrophage–tumor crosstalk and metastatic progression. These results suggest that UPP1 may serve as both a prognostic biomarker and a potential therapeutic target in LUAD.

## Introduction

Lung adenocarcinoma (LUAD) accounts for the majority of lung cancer cases and remains the leading cause of cancer-related mortality worldwide, primarily due to high rates of distant metastasis and recurrence [1, 2]. Despite the transformative impact of immune checkpoint blockade (ICB) targeting PD-1/PD-L1, a substantial proportion of patients fail to respond or develop acquired resistance, leading to dismal clinical outcomes [3–5]. Increasing evidence implicates the complex tumor microenvironment (TME) as an important contributor to this therapeutic failure [6, 7]. Specifically, the establishment of an immunosuppressive niche not only facilitates immune evasion but also actively supports metastatic dissemination [8–10]. Therefore, deciphering the molecular mechanisms governing TME-mediated immunosuppression is critical for identifying novel therapeutic targets to overcome current clinical bottlenecks and improve patient survival.

Within the stromal compartment, tumor-associated macrophages (TAMs) represent the most abundant infiltrating immune cells and serve as central architects of the immunosuppressive landscape [11–13]. Although classically categorized into pro-inflammatory M1 and anti-inflammatory M2 phenotypes, recent single-cell RNA sequencing (scRNA-seq) studies have unveiled a far more complex heterogeneity within macrophage populations [14–16]. TAMs are increasingly recognized as contributors to reduced cytotoxic T-cell activity and tumor progression through metabolic reprogramming and cytokine secretion [10, 17–19]. However, the specific metabolic checkpoints that drive the functional polarization of TAMs towards a pro-metastatic phenotype remain incompletely understood [20, 21]. Identifying molecular regulators linking macrophage metabolism to immunosuppressive function represents a major unmet need in the field.

Uridine Phosphorylase 1 (UPP1) is a key enzyme in pyrimidine salvage pathways, traditionally studied for its role in regulating uridine homeostasis and fluoropyrimidine sensitivity [22, 23]. While elevated UPP1 expression has been observed in various malignancies [24, 25], its specific cellular source and biological function within the complex TME ecosystem have been largely overlooked. Our preliminary single-cell transcriptomic analysis indicates a distinct and preferential enrichment of UPP1 within the myeloid lineage, particularly in TAMs, rather than in malignant epithelial cells. Despite this compelling association, whether and how macrophage-associated UPP1 contributes to the immunosuppressive landscape and metastatic progression of LUAD remains poorly understood.

In this study, we integrated single-cell transcriptomics, clinical cohort analysis, and in vivo phenotypic analyses to characterize the role of UPP1 in LUAD progression. Our findings support a role for UPP1 as a metabolic modulator in TAMs, accompanied by mitochondrial dysfunction and ROS accumulation. These alterations were accompanied by activation of a cGAS-STING-NLRP3 signaling axis, macrophage pyroptosis, and increased IL-1β release. In vitro, macrophage-derived IL-1β promoted epithelial–mesenchymal transition (EMT) in tumor cells via paracrine signaling, while in vivo tumors co-injected with UPP1-overexpressing macrophages exhibited increased metastatic burden. Furthermore, our analyses identify UPP1 as an independent prognostic biomarker and suggest its potential relevance for metabolic and immune modulation in LUAD.

## Results

### Single-cell transcriptomic analysis identifies macrophage-enriched UPP1 expression associated with metastatic features and poor prognosis in LUAD

**Fig. 1:**
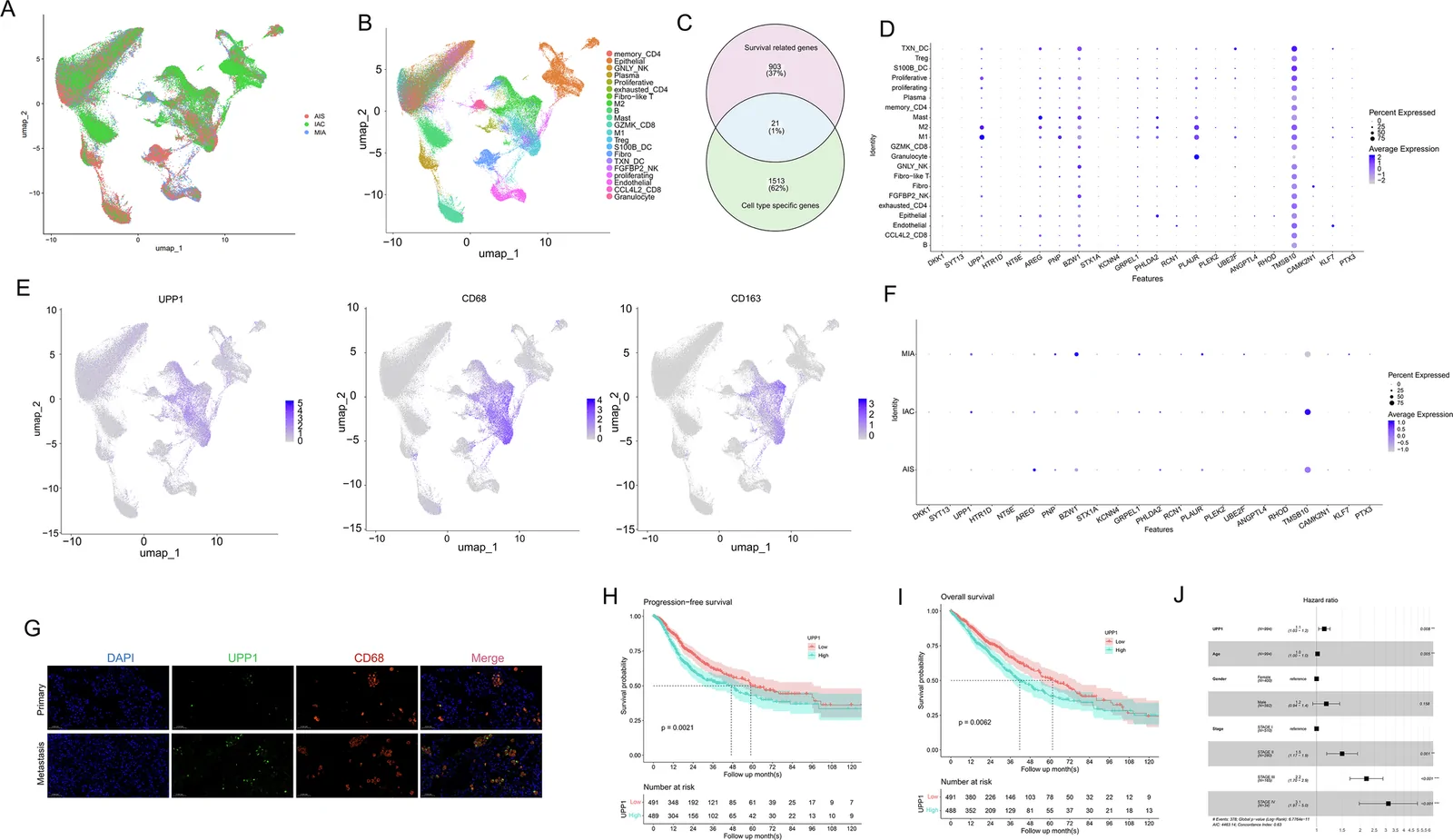
Single-cell transcriptomic analysis identifies macrophage-enriched UPP1 expression associated with metastatic features and poor prognosis in LUAD. **A** Uniform Manifold Approximation and Projection (UMAP) plot visualizing dimension reduction analysis of single cells derived from lung adenocarcinoma (LUAD) samples, color-coded by pathological stages: Adenocarcinoma in situ (AIS), Invasive adenocarcinoma (IAC), and Minimally invasive adenocarcinoma (MIA). **B** UMAP projection showing the landscape of major cell populations within the tumor microenvironment (TME), annotated into distinct lineages based on canonical marker gene expression. **C** Venn diagram illustrating the combinatorial screening strategy, intersecting genes significantly associated with patient survival (*n* = 903) and genes exhibiting cell-type-specific expression patterns (*n* = 1513). **D** Dot plot displaying the expression profile of 21 candidate genes across distinct cell clusters; dot size represents the percentage of expressing cells, and color intensity indicates average scaled expression. **E** Feature plots visualizing the spatial expression of UPP1 alongside canonical macrophage markers CD68 and CD163 on the UMAP embedding. **F** Dot plot quantifying dynamic expression changes of candidate genes across AIS, IAC, and MIA stages. **G** Representative immunofluorescence (IF) images of paired primary and metastatic LUAD tissue sections stained for nuclei (DAPI, blue), UPP1 (green), and CD68 (red). Scale bars, 50 µm. **H**, **I** Kaplan-Meier survival curves comparing (**H**) Progression-Free Survival (PFS) and (**I**) Overall Survival (OS) between UPP1-high and UPP1-low patient groups in the TCGA-LUAD cohort (P-values by two-sided log-rank test). **J** Forest plot summarizing multivariate Cox proportional hazards regression analysis for UPP1 expression and clinical covariates (Age, Gender, TNM Stage). Error bars indicate 95% Confidence Intervals (CI).

To characterize the cellular landscape and transcriptional features associated with LUAD progression, we performed dimension reduction analysis on scRNA-seq data (GSE189357). The UMAP projection visualized the distribution of cells across distinct pathological stages, ranging from Adenocarcinoma in situ (AIS) to Invasive adenocarcinoma (IAC) (Fig. 1A). Unsupervised clustering subsequently annotated these cells into major lineages, including T cells, B cells, NK cells, fibroblasts, endothelial cells, and myeloid populations (Fig. 1B). The identity of these clusters was defined by the expression of canonical lineage markers (e.g., MS4A1, LYZ, CD3D), as confirmed by dot plot analysis (Suppl. Fig. S1A).

To identify candidate genes with potential clinical relevance, we devised a combinatorial screening strategy by intersecting genes significantly associated with patient survival (903 genes) with those exhibiting cell-type-specific expression patterns (1513 genes), yielding 21 potential candidates (Fig. 1C). Notably, among these candidates, UPP1 emerged as a prominent hit and displayed relatively enriched expression in macrophage-related myeloid clusters, particularly M1- and M2-like macrophage populations (Fig. 1D). Feature plots further refined the cellular source of UPP1, demonstrating a overlap with CD68 and CD163, thereby indicating predominant expression in TAM populations (Fig. 1E).

We next investigated the temporal dynamics of UPP1 during disease evolution. Analysis across pathological stages revealed a progressive upregulation of UPP1 during the transition from in situ (AIS) to invasive carcinoma (IAC) (Fig. 1F), suggesting an association with tumor progression. To explore the immunological consequences of this upregulation, we interrogated the TCGA cohort. Consistent with immune-related transcriptomic alterations, UPP1 levels were negatively correlated with inferred CD8 + T cell abundance (Suppl. Fig. 1B) while positively correlating with M2 macrophage abundance (Suppl. Fig. 1C) and PD-L1 expression (Suppl. Fig. 1D). While these correlations are statistically significant, they primarily provide hypothesis-generating insights that serve as a rationale for our subsequent mechanistic investigation.

These transcriptomic insights were further validated at the protein level via immunofluorescence staining of paired primary and metastatic tissues. The results showed strong co-localization of UPP1 with CD68, with metastatic samples displaying markedly higher UPP1 intensity compared to primary tumors (Fig. 1G). RNA-sequencing of lung cancer cell lines revealed that UPP1 clusters with genes upregulated in highly metastatic lines (Suppl. Fig. 1E), with quantitative analysis confirming significantly elevated FPKM values in the high-metastatic group (Suppl. Fig. 1F). Finally, the clinical significance of UPP1 was underscored by survival analyses, where high expression predicted shorter progression-free survival (PFS) (Fig. 1H) and overall survival (OS) (Fig. 1I). Multivariate Cox regression further identified UPP1 as an independent risk factor for adverse outcomes (Fig. 1J). Given the observed association between macrophage-enriched UPP1 expression and adverse clinical features, we selected this molecule for deeper investigation into the underlying mechanisms.

### UPP1-associated metabolic alterations are linked to mitochondrial stress and activation of the cGAS–STING pathway

**Fig. 2:**
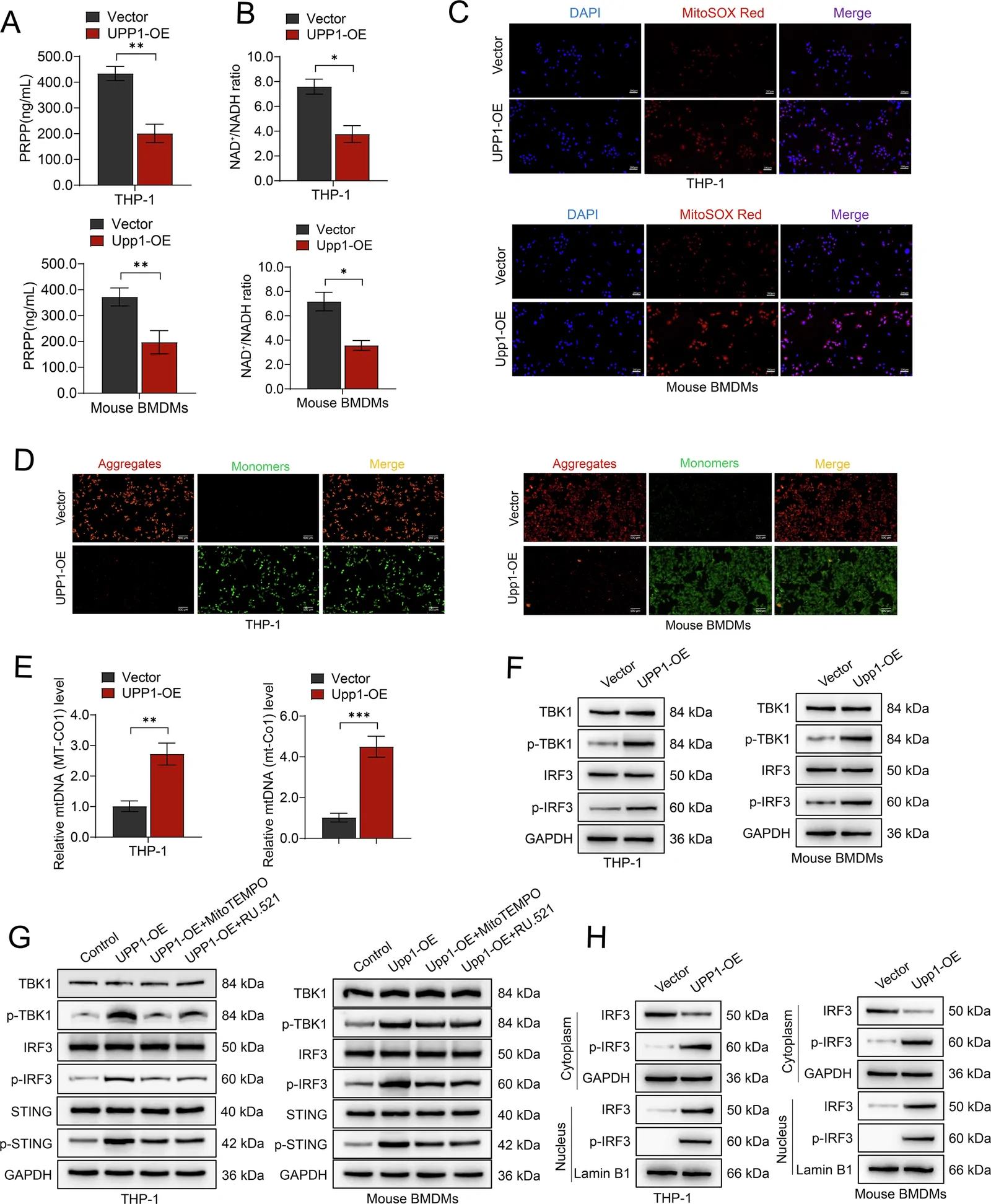
UPP1-associated metabolic alterations are accompanied by mitochondrial stress and cGAS–STING pathway activation. **A**, **B** Quantitative analysis of intracellular metabolic parameters in human THP-1 cells and BMDMs. UPP1-OE significantly reduced intracellular PRPP concentrations (**A**) and decreased the NAD + /NADH redox ratio (**B**) compared with Vector controls. **C** Representative fluorescence microscopy images showing increased mitochondrial reactive oxygen species (mtROS) accumulation in UPP1-OE macrophages, as detected by MitoSOX Red staining. Nuclei were counterstained with DAPI (blue). Scale bar = 200 µm. **D** Assessment of mitochondrial membrane potential (ΔΨm) using the JC-1 fluorescent probe. UPP1-OE induced a shift from red fluorescence (J-aggregates, polarized mitochondria) to green fluorescence (monomers, depolarized mitochondria). Scale bar = 500 µm. **E** qRT-PCR analysis revealing increased leakage of mitochondrial DNA (mtDNA) into the cytosol in UPP1-OE cells. Cytosolic levels of MT-CO1 (human) or mt-Co1 (mouse) were normalized to nuclear DNA. **F** Western blot analysis demonstrating that UPP1-OE enhances the phosphorylation of TBK1 (p-TBK1) and IRF3 (p-IRF3) in both human and mouse macrophages. **G** Immunoblot analysis showing that treatment with the mtROS scavenger MitoTEMPO (200 µM) or the cGAS inhibitor RU.521 (1 µM) for 24 h effectively abrogates the UPP1-induced activation of the STING-TBK1-IRF3 signaling axis. **H** Subcellular fractionation and Western blot analysis confirming the promoted nuclear translocation of phosphorylated IRF3 upon UPP1 overexpression. GAPDH and Lamin B1 served as loading controls for the cytoplasmic and nuclear fractions, respectively. Data are presented as mean ± SD from three independent experiments (*n* = 3). Statistical significance was determined by two-tailed Student’s t-test (**A**, **B**, **E**) or one-way ANOVA followed by Tukey’s post-hoc test (**G**). *P < 0.05, **P < 0.01, ***P < 0.001.

To investigate the molecular mechanisms linking UPP1 enzymatic activity to macrophage immunomodulation, we first established stable UPP1-overexpressing (UPP1-OE) models in both human THP-1 cells and mouse bone marrow-derived macrophages (BMDMs). The overexpression efficiency was confirmed at both the mRNA and protein levels (Suppl. Fig. S2A, B). With this validated system, we investigated the metabolic consequences of UPP1 dysregulation. Given that UPP1 catalyzes the reversible phosphorolysis of uridine, we hypothesized that its deregulation might disrupt intracellular nucleotide and energy homeostasis. Indeed, quantitative metabolic analysis revealed a significant reduction in the levels of PRPP, a key intermediate in nucleotide synthesis, in UPP1-OE cells compared to vector controls (Fig. 2A). Concurrently, UPP1 overexpression led to a marked decrease in the NAD + /NADH ratio (Fig. 2B), indicative of a disrupted cellular redox state and potential impairment of the electron transport chain.

Since mitochondria are the primary regulators of cellular redox homeostasis, we hypothesized that this metabolic perturbation may converge on mitochondrial dysfunction. To test this, we used MitoSOX Red staining to detect mitochondrial superoxide, we observed a substantial accumulation of mtROS in UPP1-OE cells (Fig. 2C). This oxidative stress was accompanied by a collapse in mitochondrial membrane potential (ΔΨm). JC-1 staining revealed that while control cells maintained polarized mitochondria rich in J-aggregates, UPP1-OE cells exhibited profound depolarization, evidenced by a prominent shift towards monomeric green fluorescence (Fig. 2D). Collectively, these data indicate that UPP1 overexpression is associated with mitochondrial oxidative stress and membrane potential loss.

Severe mitochondrial dysfunction frequently precipitates the cytosolic leakage of mitochondrial DNA (mtDNA), a potent damage-associated molecular pattern (DAMP). Consistent with the observed membrane depolarization, qPCR analysis of subcellular fractions revealed significantly elevated levels of cytosolic mtDNA (MT-CO1) in UPP1-OE cells compared to controls (Fig. 2E). To verify that these metabolic alterations and the subsequent mtDNA leakage were driven by mitochondrial oxidative stress, we performed rescue experiments using the mitochondrial ROS scavenger MitoTEMPO. As expected, MitoTEMPO treatment effectively attenuated the UPP1-induced accumulation of mtROS (Suppl. Fig. S3A). Notably, scavenging mtROS also significantly restored the intracellular levels of PRPP and the NAD + /NADH ratio (Suppl. Fig. S3B–C), and reversed the accumulation of cytosolic mtDNA (Suppl. Fig. S3D). These data support a model in which oxidative stress contributes to both metabolic alterations and mtDNA release in UPP1-OE cells. Since cytosolic DNA is the specific ligand for cyclic GMP-AMP synthase (cGAS), we hypothesized that this leakage activates the STING signaling axis. Western Blotting analysis showed that UPP1 overexpression significantly increased the phosphorylation levels of key pathway components, specifically TBK1 (p-TBK1) and IRF3 (p-IRF3), in both THP-1 cells and BMDMs (Fig. 2F). To further assess the relationship between mtROS accumulation, cGAS activation, and this signaling cascade, we performed rescue experiments using specific inhibitors. Treatment with either MitoTEMPO (a mitochondria-targeted antioxidant) or RU.521 (a specific cGAS inhibitor) effectively reversed the UPP1-induced phosphorylation of STING, TBK1, and IRF3 (Fig. 2G). These data confirm that UPP1 activates the STING pathway via a mtROS-cGAS-dependent mechanism. Finally, since the functional execution of this pathway requires the nuclear translocation of transcription factors, we examined the subcellular localization of IRF3. Nuclear/cytoplasmic fractionation assays demonstrated that UPP1 overexpression promoted the robust translocation of phosphorylated IRF3 from the cytoplasm to the nucleus (Fig. 2H). Collectively, these findings support a model in which UPP1 overexpression is associated with metabolic alterations, mitochondrial stress, mtDNA leakage, and activation of the cGAS-STING-IRF3 signaling pathway.

### UPP1 overexpression induces macrophage pyroptosis through the activation of the NLRP3 inflammasome via the mtROS-cGAS-STING Axis

**Fig. 3:**
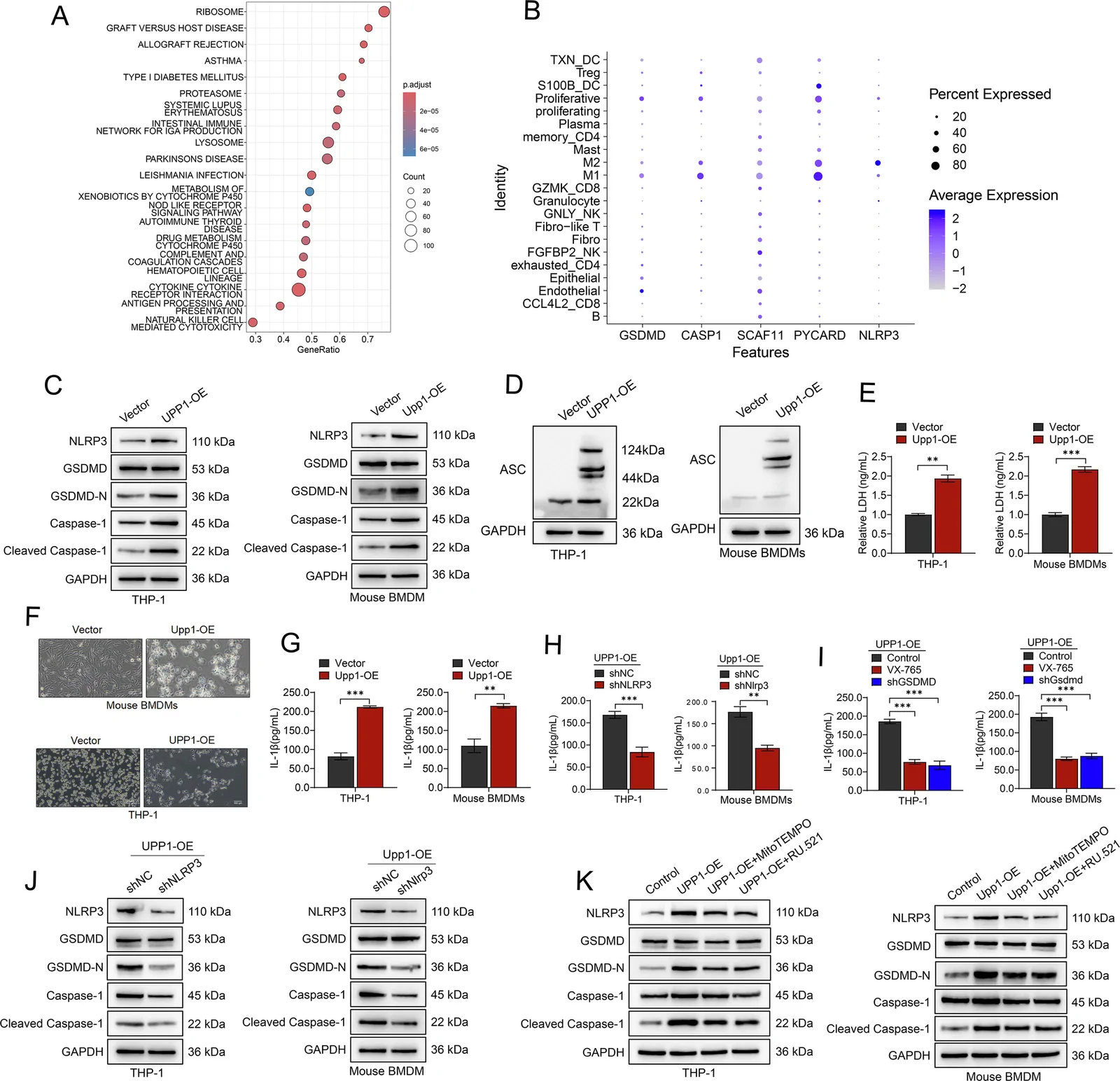
UPP1 overexpression is associated with macrophage pyroptosis and NLRP3 inflammasome activation in the context of the mtROS-cGAS-STING axis. **A** KEGG pathway enrichment analysis of differentially expressed genes (DEGs) from the TCGA cohort, highlighting immune-related pathways. **B** Dot plot from scRNA-seq data visualizing that pyroptosis-related genes (GSDMD, CASP1, PYCARD, NLRP3) are preferentially expressed in macrophage subsets (M1 and M2). **C** Western blot analysis in THP-1 cells and BMDMs showing that UPP1 overexpression (UPP1-OE) triggers the cleavage of GSDMD (full-length: 53 kDa; active N-terminal fragment: 36 kDa) and Caspase-1 (pro: 45 kDa; cleaved: 22 kDa). **D** Immunoblot detection of ASC oligomerization in DSS-crosslinked lysates, demonstrating inflammasome assembly in UPP1-OE cells. **E** LDH release assay quantifying plasma membrane rupture, indicating increased pyroptotic cell death in UPP1-OE macrophages. **F** Representative phase-contrast microscopy images showing characteristic pyroptotic morphology (cellular swelling and large bubbles). Scale bar = 500 µm. **G** ELISA quantification of mature IL-1β secretion in culture supernatants. **H**, **I** ELISA showing that the UPP1-induced IL-1β release is significantly abrogated by shRNA-mediated knockdown of NLRP3 (**H**) or GSDMD (**I**), and pharmacological inhibition of Caspase-1 by VX-765 (**I**). **J** Western blot validating that NLRP3 knockdown prevents the cleavage of Caspase-1 and GSDMD. **K** Western blot demonstrating that the activation of the NLRP3-Caspase-1-GSDMD axis is dependent on mitochondrial ROS and cGAS, as evidenced by the inhibitory effects of MitoTEMPO and RU.521.Data are mean ± SD (*n* = 3). Significance assessed by Student’s t-test or one-way ANOVA (**P < 0.01,***P < 0.001).

To elucidate the specific signaling pathways modulated by UPP1, we first performed KEGG pathway enrichment analysis using transcriptomic data from the TCGA cohort. The results highlighted that immune-related pathways, specifically the “NOD-like receptor signaling pathway, were significantly enriched, suggesting a potential involvement of inflammasome activation (Fig. 3A). To contextualize this within the TME, we analyzed scRNA-seq data. Dot plot analysis revealed that key pyroptosis-related genes—including GSDMD, CASP1, PYCARD, and NLRP3—were prominently expressed within macrophage populations (M1 and M2) (Fig. 3B), highlighting macrophages as a key population associated with this pathway.

Based on these predictions, we validated the activation of the pyroptotic cascade at the protein level. Western Blotting analysis showed that UPP1-OE in both THP-1 cells and mouse BMDMs led to a marked upregulation of NLRP3 and significantly increased the cleavage of Caspase-1 and Gasdermin D (GSDMD), generating their active N-terminal forms (~36 kDa, Fig. 3C). Since inflammasome activation requires the assembly of the ASC adaptor, we assessed ASC oligomerization. Immunoblotting of cross-linked lysates revealed that UPP1 overexpression promoted the formation of ASC oligomers, a hallmark of inflammasome assembly (Fig. 3D). Given that ASC oligomerization is the prelude to cell death, we next assessed whether this signaling cascade culminated in pyroptosis. UPP1-OE cells exhibited significantly higher levels of LDH release compared to vector controls, indicating a loss of plasma membrane integrity (Fig. 3E). Consistent with this compromised membrane integrity, microscopic examination revealed that UPP1-OE cells displayed characteristic pyroptotic morphology, including cellular swelling and large bubble-like herniations (Fig. 3F). Furthermore, ELISA analysis indicated that UPP1 overexpression significantly promoted the secretion of mature IL-1β, a key downstream pro-inflammatory cytokine (Fig. 3G). Collectively, these in vitro findings suggest that enhanced UPP1 expression can promote the transduction of mitochondrial stress into a robust, NLRP3-dependent inflammatory response.

To further assess whether the observed pyroptosis and cytokine release were specifically dependent on the NLRP3 inflammasome, we performed rescue experiments using shRNA. Knockdown of NLRP3 (shNLRP3) in UPP1-OE cells significantly abrogated the elevated IL-1β secretion observed in the UPP1-OE group (Fig. 3H). Similarly, pharmacological inhibition of Caspase-1 (using VX-765) or genetic knockdown of GSDMD (shGSDMD) attenuated the UPP1-induced release of IL-1β, supporting the involvement of the Caspase-1/GSDMD axis (Fig. 3I). We further validated these findings at the molecular level. Western Blotting analysis demonstrated that silencing NLRP3 blocked the UPP1-induced cleavage of Caspase-1 and GSDMD, supporting the placement of UPP1 upstream of the NLRP3 inflammasome (Fig. 3J). Finally, to further examine the contribution of mitochondrial dysfunction and cGAS signaling to this phenotype, we employed specific inhibitors: MitoTEMPO (to scavenge mtROS) and RU.521 (to target cGAS). Both treatments significantly inhibited the UPP1-induced upregulation of NLRP3 and the subsequent cleavage of Caspase-1 and GSDMD (Fig. 3K). Collectively, these in vitro data demonstrate that UPP1 overexpression triggers mitochondrial damage and cGAS-STING activation, which subsequently primes and activates the NLRP3 inflammasome, promoting macrophage pyroptosis.

### UPP1-overexpressing macrophages promote pro-tumor phenotypes and EMT in tumor cells via paracrine signaling

**Fig. 4:**
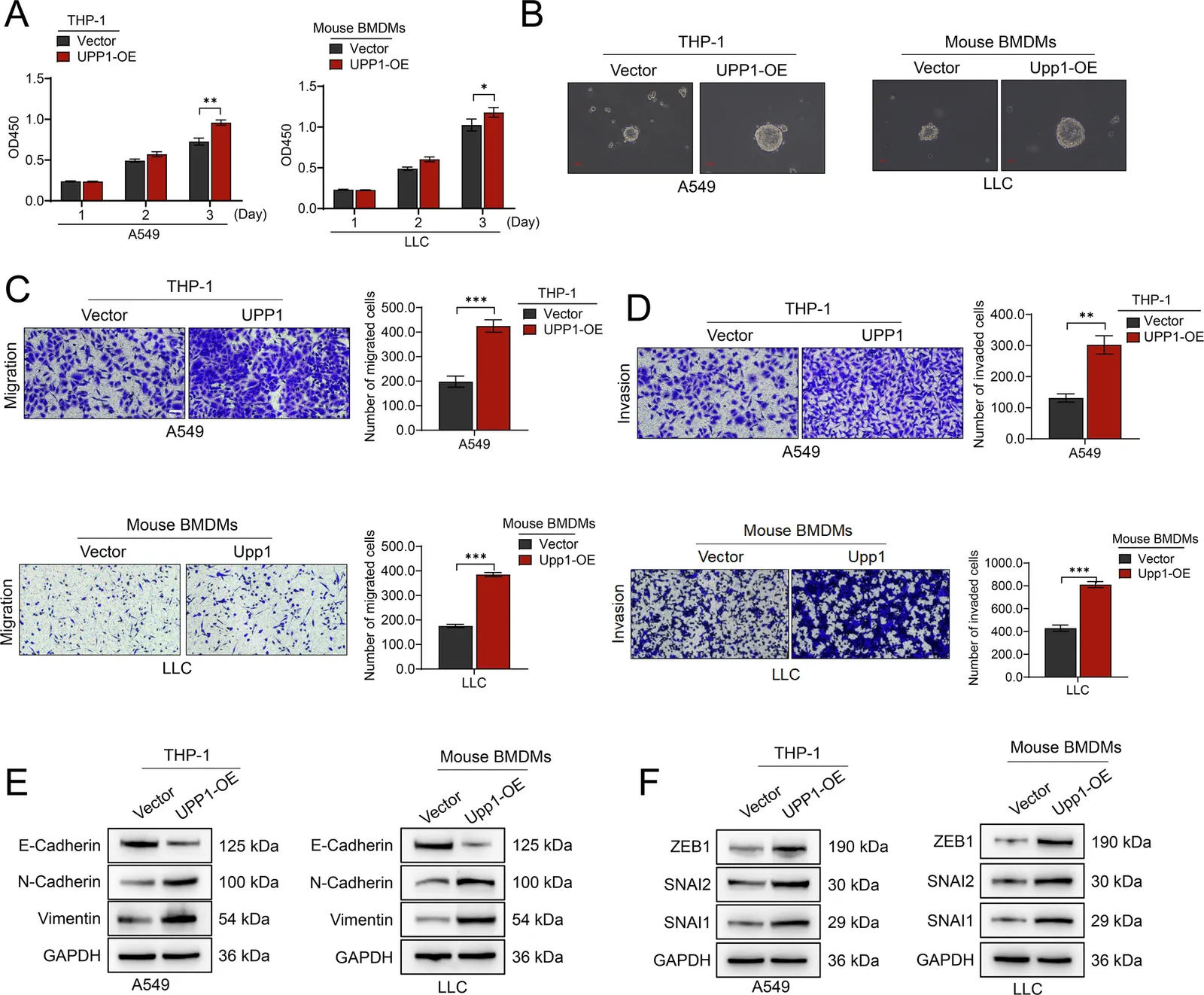
UPP1-overexpressing macrophages are associated with enhanced tumor cell proliferation and EMT-like phenotypes in vitro. **A** Proliferation analysis of human A549 and mouse LLC cells. Co-culture with UPP1-OE macrophages (THP-1 or BMDMs) significantly accelerated the growth of tumor cells starting from day 3, as determined by CCK-8 assay. **B** Representative images of tumor spheres. A549 and LLC cells exposed to UPP1-OE macrophages exhibited enhanced anchorage-independent growth and increased sphere size, indicating promoted cancer stem-like properties. Scale bar = 50 µm. **C**, **D** Assessment of tumor cell motility using Transwell migration.Scale bar = 500 µm. (**C**) and Matrigel-coated invasion (**D**) assays. UPP1-OE macrophages significantly increased the number of migrated and invaded tumor cells compared to the Vector control group. **E** Western blot analysis of EMT markers. Treatment with UPP1-OE macrophage-derived CM induced a mesenchymal-like switch in A549 and LLC cells, characterized by the downregulation of E-Cadherin and upregulation of N-Cadherin and Vimentin. **F** Immunoblotting analysis of EMT-inducing transcription factors (ZEB1, SNAI2, SNAI1). UPP1-OE macrophage CM significantly promoted the protein expression of these key transcription factors, driving the EMT process in tumor cells. Data are mean ± SD (*n* = 3. Significance determined by two-way ANOVA (proliferation) or Student’s t-test (bar graphs) (*P < 0.05, **P < 0.01, ***P < 0.001).

Having observed that UPP1 overexpression is associated with pyroptosis and cytokine release, we next interrogated whether this UPP1-induced secretome could function in a paracrine manner to alter tumor cell behavior. To simulate these interactions in vitro, we established co-culture systems pairing human A549 cells with UPP1-overexpressing (UPP1-OE) THP-1 macrophages, and mouse LLC cells with UPP1-OE BMDMs. We first evaluated tumor cell proliferation using the CCK-8 assay. The results showed that A549 and LLC cells co-cultured with UPP1-overexpressing (UPP1-OE) macrophages exhibited significantly higher proliferation rates compared to those co-cultured with vector-control macrophages, particularly evident by day 3 (Fig. 4A). To further evaluate tumorigenic potential and stemness, we performed a sphere-formation assay. Tumor cells exposed to UPP1-OE macrophages formed larger and more numerous spheres compared to controls, indicating an enhanced capacity for anchorage-independent growth (Fig. 4B).

We next evaluated the effect of UPP1-overexpressing macrophages on the migratory and invasive capabilities of tumor cells. Transwell migration assays revealed that the number of migrated A549 and LLC cells was significantly increased following co-culture with UPP1-OE macrophages (Fig. 4C). Similarly, Matrigel invasion assays demonstrated that UPP1-OE macrophages significantly promoted the invasive potential of both tumor cell lines compared to the vector control group (Fig. 4D). Together, these assays suggest that UPP1 overexpression in macrophages is associated with enhanced pro-tumor phenotypes in vitro.

To explore the molecular features associated with this invasive phenotype, we assessed EMT markers in tumor cells exposed to macrophage-conditioned medium (CM). Western blot analysis revealed a distinct EMT signature in both A549 and LLC cells treated with UPP1-OE supernatant: a downregulation of the epithelial marker E-Cadherin and a concurrent upregulation of mesenchymal markers N-Cadherin and Vimentin (Fig. 4E). Furthermore, we examined the upstream transcriptional regulators of EMT. The expression levels of key EMT-inducing transcription factors—ZEB1, SNAI2 (Slug), and SNAI1 (Snail)—were markedly upregulated in tumor cells exposed to UPP1-OE macrophage conditioned medium (Fig. 4F). Consistent with the protein data, qPCR analysis confirmed that the mRNA levels of Snai1, Snai2, and Zeb1 were also significantly elevated in these tumor cells (Suppl. Fig. S4), suggesting that factors released from UPP1-OE macrophages induce EMT-related transcriptional programs in tumor cells. Collectively, these data indicate that UPP1-overexpressing macrophages secrete soluble factors that induce EMT-like changes in tumor cells in vitro, highlighting their potential to drive a pro-metastatic phenotype.

### Macrophage-derived IL-1β contributes to UPP1-associated pro-tumor phenotypes in tumor cells via paracrine signaling

**Fig. 5:**
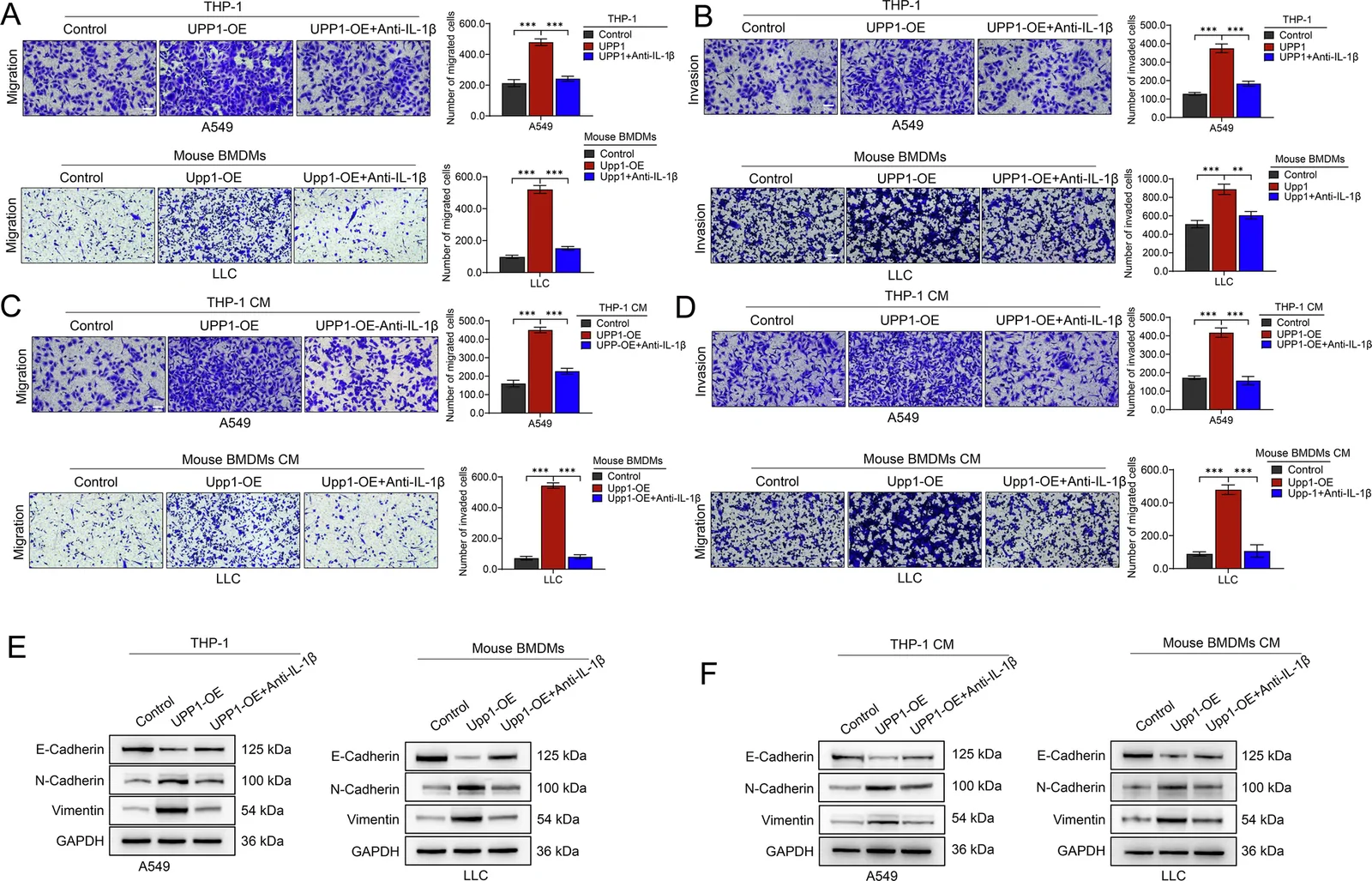
Macrophage-derived IL-1β is associated with tumor cell motility and EMT-like changes induced by UPP1-overexpressing macrophages in vitro. **A**, **B** Rescue experiments in co-culture systems assessing the contribution of IL-1β. The enhanced migration (**A**) and invasion (**B**) of A549 and LLC cells observed in the presence of UPP1-OE macrophages were significantly reduced by the addition of an IL-1β neutralizing antibody. Scale bar = 500 µm. **C**, **D** Validation of the paracrine mechanism using CM. CM derived from UPP1-OE macrophages increased tumor cell motility, whereas the inclusion of anti-IL-1β in the CM significantly attenuated these pro-migratory and pro-invasive effects in vitro in both migration (**C**) and invasion (**D**) assays. Scale bar = 500 µm. **E**, **F** Western blot analysis of EMT markers. Blocking IL-1β signaling, either in the co-culture system (**E**) or via CM stimulation (**F**), prevented the UPP1-induced loss of E-Cadherin and the upregulation of mesenchymal markers (N-Cadherin and Vimentin) in tumor cells. Data are mean ± SD (*n* = 3). Statistical significance determined by one-way ANOVA followed by Tukey’s post hoc test (**P < 0.01, ***P < 0.001).

Having identified IL-1β as a prominently elevated cytokine in the secretome of UPP1-overexpressing macrophages, we next examined whether it might function as a paracrine mediator of tumor cell behavior. To test this, we introduced a neutralizing antibody against IL-1β (Anti-IL-1β) into the co-culture systems. In co-culture systems, Transwell migration assays showed that the enhanced migration of A549 and LLC cells observed in the presence of UPP1-OE macrophages was significantly reduced by IL-1β neutralization (Fig. 5A). Similarly, Matrigel invasion assays showed that IL-1β blockade attenuated the invasive phenotype associated with UPP1-OE macrophages (Fig. 5B). To confirm that this effect was driven by soluble factors released into the extracellular environment rather than direct cell-to-cell contact, we utilized CM from macrophages. Consistent with the co-culture results, treatment of tumor cells with CM from UPP1-OE macrophages significantly increased cell migration (Fig. 5C) and invasion (Fig. 5D). Importantly, the addition of the IL-1β neutralizing antibody to the CM markedly reduced these pro-migratory and pro-invasive effects in vitro, supporting a role for secreted IL-1β as a paracrine mediator in this system. To further assess whether this paracrine effect was associated with GSDMD-dependent pyroptotic signaling, we performed genetic knockdown of GSDMD in UPP1-OE macrophages. Consistent with the antibody neutralization results, silencing GSDMD significantly reduced the enhanced migration and invasion of co-cultured tumor cells (Suppl. Fig. S5), supporting the involvement of GSDMD-dependent cytokine release in these tumor cell phenotypes.

Since we previously observed that UPP1-OE macrophages induce EMT in tumor cells, we sought to determine if this process was IL-1β-dependent. Western blot analysis of tumor cells from the co-culture system showed that IL-1β neutralization prevented the loss of the epithelial marker E-Cadherin and blocked the upregulation of the mesenchymal markers N-Cadherin and Vimentin (Fig. 5E). Notably, we also examined the effect of IL-1β neutralization on the macrophages themselves. Western blot analysis revealed that blocking extracellular IL-1β partially attenuated the upregulation of NLRP3 and the cleavage of Caspase-1 and GSDMD in UPP1-OE macrophages (Suppl. Fig. S6). This finding raises the possibility that secreted IL-1β may contribute to sustaining macrophage pyroptotic signaling through an autocrine feedback mechanism. Furthermore, a similar rescue effect was observed when tumor cells were treated with conditioned medium: addition of α-IL-1β to UPP1-OE CM partially restored the epithelial expression profile (Fig. 5F). Collectively, these findings support a model in which UPP1-associated macrophage pyroptotic signaling is accompanied by IL-1β release, which may act in a paracrine manner to promote EMT-like changes and invasive phenotypes in tumor cells in vitro.

### Co-injection of UPP1-overexpressing macrophages is associated with increased tumor growth and pulmonary metastatic burden in vivo

**Fig. 6:**
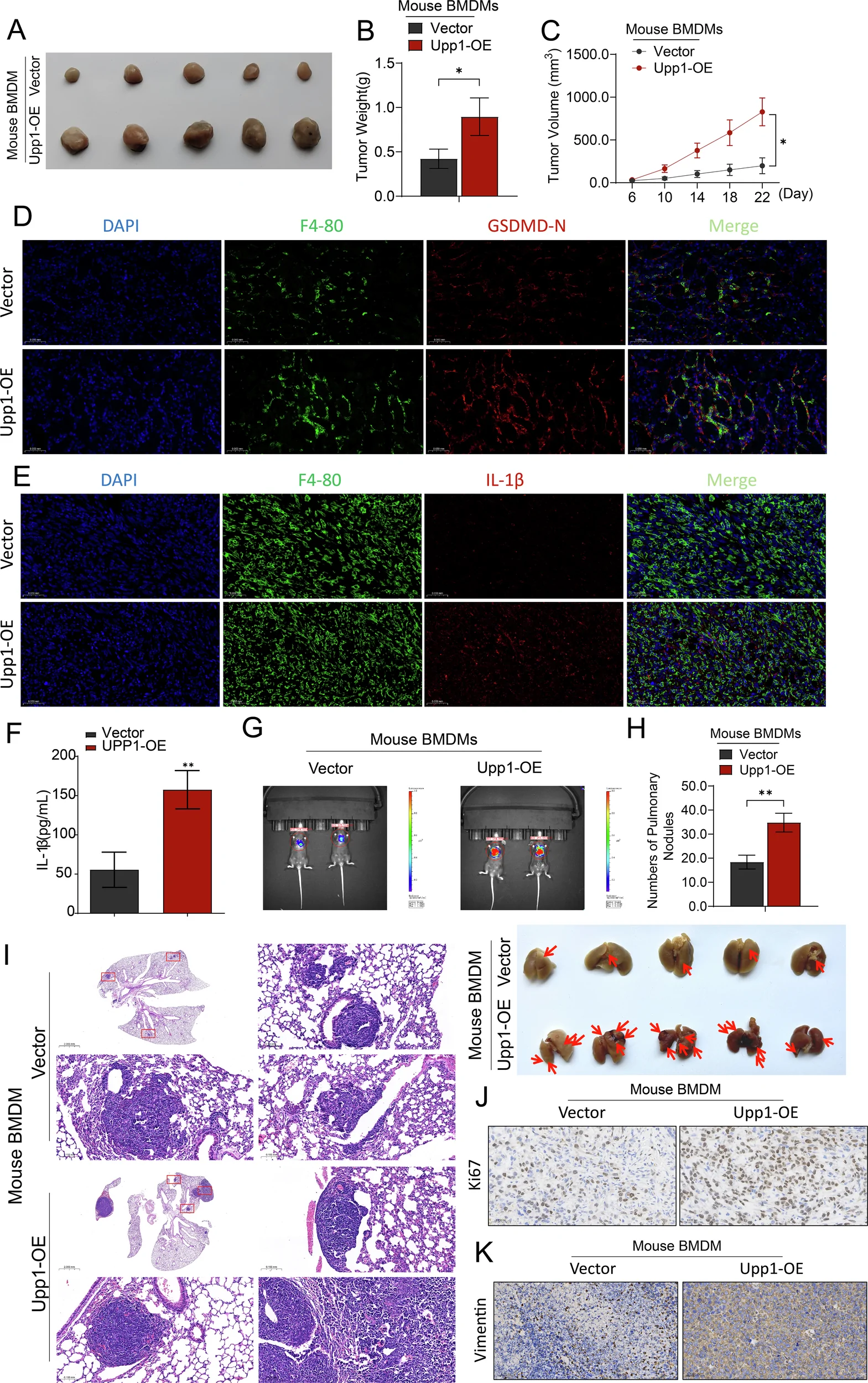
Co-injection of UPP1-overexpressing macrophages is associated with increased tumor growth and pulmonary metastatic burden in vivo. **A**–**C** Evaluation of primary tumor growth in a C57BL/6 subcutaneous co-injection model. LLC cells (5 × 10⁵) were mixed with either Vector-control or Upp1-OE BMDMs (1 × 10⁵) and injected into the right flank of mice (*n* = 5 per group). Shown are representative images of excised tumors (**A**), final tumor weights at day 28 (**B**), and longitudinal tumor volume curves (**C**). **D** Representative immunofluorescence images of tumor sections stained for F4/80 (green) and GSDMD-N (red). Increased GSDMD-N signals were observed in regions enriched with F4/80⁺ macrophages in the Upp1-OE group. Nuclei were counterstained with DAPI (blue). Scale bar = 50 µm. **E** Representative immunofluorescence images of tumor sections stained for F4/80 (green) and IL-1β (red). Increased IL-1β signals were observed in regions enriched with F4/80⁺ macrophages in the Upp1-OE group. Nuclei were counterstained with DAPI (blue). Scale bar = 50 µm. **F** ELISA quantification of IL-1β levels in tumor tissues from the Vector and Upp1-OE co-injection groups. **G** Representative thoracic bioluminescence images (IVIS) of mice from the Vector and Upp1-OE co-injection groups. **H** Representative gross anatomical photographs of excised lungs (Down; red arrows indicate visible nodules) and quantification of pulmonary surface nodules (Up). Surface nodules were independently counted by two researchers blinded to group allocation. **I** Representative hematoxylin and eosin (H&E) staining of lung sections showing hypercellular lesions consistent with metastatic tumor foci in the Vector and Upp1-OE groups. **J**, **K** Representative immunohistochemical staining of lung metastatic lesions for Ki67 (**J**) and Vimentin (**K**), supporting increased proliferative and mesenchymal features in the Upp1-OE group. Scale bar = 50 µm. Ki67 and Vimentin staining were performed on independent sections and are presented as representative assessments of proliferative and mesenchymal features of metastatic lesions. Data are presented as mean ± SD. Statistical significance was determined by two-tailed Student’s t-test (**B**, **F**, **H**) or two-way ANOVA (**C**). *P < 0.05, **P < 0.01.

To extend our in vitro findings to an in vivo context, we established a subcutaneous tumor model by co-injecting LLC cells with either vector-control or UPP1-OE mouse BMDMs into the flanks of mice. We monitored tumor progression to determine the impact of these engineered macrophages on tumorigenesis. At the study endpoint, morphological examination revealed that tumors in the UPP1-OE group were visibly larger than those in the vector control group (Fig. 6A). Quantitative analysis confirmed that the mean tumor weight was significantly higher in the UPP1-OE group (Fig. 6B). Furthermore, measurements of tumor volume demonstrated a significantly accelerated growth rate in mice co-injected with UPP1-OE macrophages compared to controls (Fig. 6C). Collectively, these data indicate that tumors co-injected with UPP1-overexpressing macrophages exhibit enhanced growth in this model.

We examined the occurrence of pyroptosis and the resulting inflammatory response within the tumor microenvironment. Immunofluorescence (IF) analysis showed increased GSDMD-N signals in regions enriched with the macrophage marker F4/80 in UPP1-OE tumors, supporting enhanced macrophage-associated pyroptotic signaling in this group (Fig. 6D). Furthermore, to assess the functional consequence of this pyroptosis, we evaluated IL-1β expression. IF staining showed increased IL-1β signals in regions enriched with F4/80⁺ macrophages in the UPP1-OE group (Fig. 6E). Consistent with these histological findings, ELISA analysis confirmed significantly elevated levels of IL-1β in the UPP1-overexpressing group compared to the vector control (Fig. 6F). Together, these data are consistent with our in vitro findings and support the presence of an IL-1β-rich inflammatory microenvironment in tumors formed with UPP1-overexpressing macrophages.

Given the pronounced pro-metastatic phenotype observed in vitro, we next assessed the impact of UPP1-overexpressing macrophages on lung metastasis. In vivo bioluminescence imaging (IVIS) showed an increased luminescent signal in the thoracic region of mice bearing UPP1-OE tumors compared with control mice, consistent with elevated pulmonary metastatic burden (Fig. 6G). Gross anatomical examination of the lungs corroborated these imaging findings; while control lungs appeared relatively healthy, the lungs from the UPP1-OE group were covered with numerous visible metastatic nodules (indicated by red arrows), and quantification confirmed a marked increase in pulmonary nodule number (Fig. 6H). Furthermore, histological H&E staining revealed larger and more frequent metastatic foci in the lungs of the UPP1-overexpressing group (Fig. 6I).

Finally, to assess the proliferative and invasive characteristics associated with these tumors, immunohistochemistry (IHC) analysis was performed. Ki67 staining, performed on independent sections, showed a higher proportion of proliferating cells in the UPP1-overexpression group than in controls (Fig. 6J). Additionally, staining for the mesenchymal marker Vimentin showed stronger expression in the UPP1-OE group (Fig. 6K), consistent with the EMT-like changes observed in vitro. Collectively, these in vivo findings indicate that, in this co-injection model, UPP1-overexpressing macrophages are associated with enhanced primary tumor growth, macrophage-associated pyroptotic and inflammatory signals, and increased pulmonary metastatic burden.

**Fig. 7:**
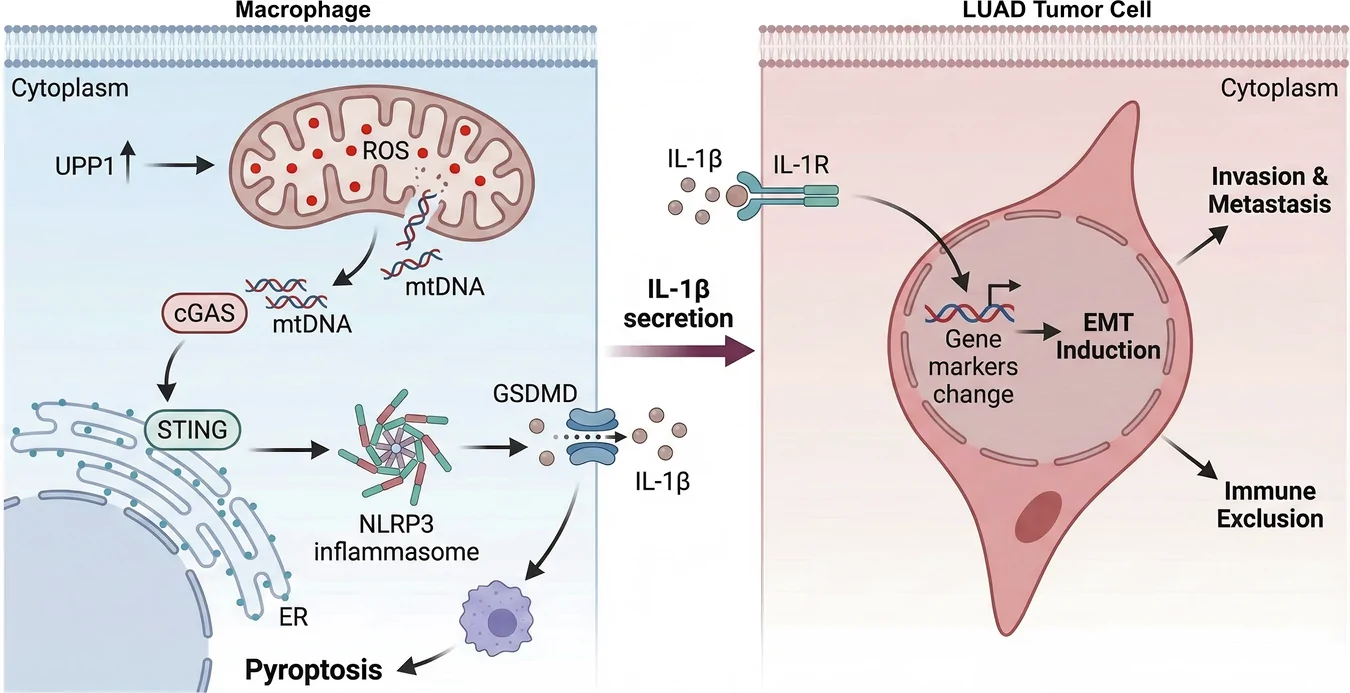
Schematic illustration of the proposed immunometabolic mechanism. Left panel: In macrophages, UPP1 ultimately associated with increased invasive behavior and metastatic burden in LUAD, ROS accumulation, and cytosolic mtDNA leakage. This stress activates the ER-localized cGAS-STING pathway, which primes the NLRP3 inflammasome, leading to GSDMD cleavage, pyroptosis, and IL-1β release. Right panel: Paracrine IL-1β binds to IL-1R on tumor cells, triggering transcriptional reprogramming and EMT, ultimately promoting invasion, metastasis, and immune exclusion in LUAD.

Based on these findings, we propose a working model (Fig. 7) in which UPP1 overexpression in macrophages induces mitochondrial stress and inflammatory signaling. The resulting cytokine release promotes EMT-like changes in tumor cells in vitro and is associated with increased metastatic progression in vivo.

## Discussion

LUAD remains the leading cause of cancer-related mortality worldwide, with metastatic progression representing the primary bottleneck in improving patient survival [26–28]. Despite the paradigm shift introduced by ICB, a significant fraction of patients exhibit primary or acquired resistance, leading to dismal clinical outcomes [29–31]. Increasing evidence implicates the TME as a critical determinant of this therapeutic failure [32, 33]. Among the stromal components, TAMs constitute the dominant fraction of the immune infiltrate and are increasingly recognized not merely as bystanders, but as active orchestrators of an immunosuppressive and pro-metastatic niche [32, 34, 35]. However, the specific metabolic regulators facilitating the functional reprogramming of TAMs remain incompletely understood.

Historically, UPP1 has been characterized as a tumor-intrinsic enzyme regulating fluoropyrimidine sensitivity or proliferation [36, 37]. Our study expands the current understanding of UPP1 by identifying it as a key metabolic feature specifically enriched in the myeloid compartment of LUAD. Unlike bulk-sequencing studies that mask cellular heterogeneity [37], our single-cell approach reveals that UPP1 expression correlates with an immune-excluded phenotype (low CD8 + T cells, high PD-L1), suggesting that UPP1+ macrophages contribute to a hostile niche that dampens adaptive immunity. While these bioinformatic associations provide valuable hypothesis-generating insights, they are inherently influenced by tumor purity and warrant further mechanistic validation.

Mechanistically, we propose that UPP1 acts as a metabolic driver associated with activation of the cGAS-STING innate immune sensor. While canonical cGAS-STING activation typically responds to viral or nuclear DNA damage [38–40], our findings highlight a distinct metabolic-driven mechanism. We show that UPP1 overexpression induces mtROS accumulation and mtDNA leakage, serving as a sterile DAMP. Although UPP1 functions in the cytosol [41], our data suggest that its dysregulation depletes the intracellular pool of PRPP and disrupts the NAD + /NADH ratio, which may contribute to metabolic stress and impaired mitochondrial homeostasis. This addresses a significant gap in understanding how sterile inflammation is sustained in the TME, suggesting metabolic enzymes can act as endogenous agonists for cytosolic DNA sensing.

Downstream of DNA sensing, our data suggest that UPP1-high macrophages exhibit enhanced coupling of STING signaling to NLRP3 inflammasome activation. While we functionally linked STING to NLRP3 using specific inhibitors, the precise molecular intermediaries connecting these pathways are complex. Based on established studies, we propose that STING-induced NF-κB activation may provide the essential transcriptional priming for NLRP3, while the marked mitochondrial depolarization and mtROS accumulation observed in our model likely trigger secondary signals, such as potassium (K + ) efflux, to drive full inflammasome assembly. While STING is often viewed as anti-tumorigenic via Type I IFN production. [42, 43] Our data support a pro-tumorigenic role in which STING signaling in UPP1-high macrophages is associated with enhanced NLRP3 inflammasome activation and chronic IL-1β release, rather than a predominantly antiviral response. This offers a plausible explanation for the potential failure of STING agonists, where broad activation may inadvertently fuel inflammasome-dependent progression [44].

The functional consequence of this axis is the paracrine induction of EMT in tumor cells, mediated by mature IL-1β. While TGF-β is regarded as a master regulator of EMT [45, 46], our study highlights the critical role of inflammatory cytokines. We provide evidence that macrophage-derived IL-1β independently sustains the mesenchymal phenotype (ZEB1/SNAI1 upregulation). These findings support a model in which the “UPP1–pyroptosis–IL-1β“ axis constitutes a distinct inflammatory source that facilitates metastasis, offering a more precise target than broad anti-cytokine therapies.

The translational relevance is underscored by our in vivo and clinical analyses. Our murine co-injection models showed that enhanced myeloid UPP1 expression is sufficient to promote tumor growth and is associated with increased pulmonary metastatic burden in this co-injection model. Clinically, high UPP1 expression was associated with poorer survival. The concordance between hyper-proliferative, mesenchymal metastatic nodules in mice and poor patient survival suggests that UPP1 may have potential as a biomarker to stratify high-risk patients for adjuvant therapies.

Targeting the UPP1-mtROS-cGAS/NLRP3-IL-1β axis opens avenues for therapeutic intervention. Developing specific UPP1 inhibitors could attenuate the pathological cascade, while repurposing drugs like mitochondrial antioxidants, cGAS inhibitors, or IL-1β antagonists (e.g., Canakinumab [47])—could disrupt downstream nodes. Furthermore, combining these anti-inflammatory strategies with ICB represents a logical path to sensitize “cold” tumors.

While our study provides a comprehensive mechanistic framework, we acknowledge several limitations that warrant future investigation. First, our mechanistic studies primarily utilized THP-1 cell lines and murine BMDMs. While these are well-established models, they may not fully recapitulate the ontogeny, plasticity, and complex microenvironmental education of primary human TAMs in vivo. Second, our in vivo conclusions rely on gain-of-function co-injection models; future studies using myeloid-specific Upp1 conditional knockout mice are necessary to definitively establish its endogenous necessity. Additionally, while our current findings highlight the pro-metastatic role of the UPP1-IL-1β axis through in vitro blockade and in vivo correlation, future studies utilizing in vivo pharmacological inhibitors or therapeutic neutralizing antibodies will be essential to further validate the clinical translational potential of this circuit. Third, although we link UPP1 to mitochondrial stress, the precise metabolic flux perturbations require further elucidation via stable isotope tracing. Fourth, while the malignant identity of lung nodules was pathologically confirmed via IHC (Ki67/Vimentin), our quantification relied on macroscopic surface counting, which might not capture deep micro-metastases. Thus, our data may represent a conservative estimate of the total metastatic burden. Finally, our clinical validation relied on bulk transcriptomics and localized immunofluorescence; integrating large-scale tissue microarrays (TMA) with multiplex immunofluorescence (mIF) or spatial transcriptomics would better resolve the spatial neighborhood of UPP1+ TAMs and their clinical relevance. Despite these limitations, our work supports a functional contribution of UPP1 upregulation to macrophage-associated metastatic progression, offering a fresh perspective on immunometabolic targeting.

In summary, our study conceptualizes UPP1 as a functional regulator of macrophage-mediated metastasis in LUAD. By delineating the “UPP1-mtROS-cGAS-NLRP3-IL-1β“ cascade, we provide a mechanistic framework that links metabolic dysregulation in the stroma to tumor aggressiveness, advocating for UPP1 as both a prognostic marker and a therapeutic target.

## Materials and Methods

### Public datasets and single-cell RNA-seq analysis

The scRNA-seq dataset GSE189357 was downloaded from the Gene Expression Omnibus (GEO). Raw gene expression matrices were processed using the Seurat R package (v4.0) [48]. Cells with low gene counts (< 200), high gene counts (> 6000, potential doublets), or high mitochondrial gene percentage (> 10%) were filtered out. The data were normalized using the “LogNormalize” method. The top 2000 variable features were identified using the “vst” selection method [49]. Principal Component Analysis (PCA) was performed, and the top 20 PCs were used for Uniform Manifold Approximation and Projection (UMAP) non-linear dimensional reduction [50]. Cell clusters were identified using the FindClusters function with a resolution of 0.5. Cell clusters were annotated based on the expression of canonical lineage markers (EPCAM for epithelial cells, PTPRC/CD45 for immune cells, CD68/CD163 for myeloid cells). Differentially expressed genes (DEGs) between groups were identified using the FindMarkers function (Wilcoxon rank-sum test). Kyoto Encyclopedia of Genes and Genomes (KEGG) pathway enrichment were performed using the clusterProfiler R package to investigate biological functions.

### TCGA data and bioinformatics analysis

RNA-seq data and clinical data for the LUAD cohort were retrieved from TCGA via the UCSC Xena browser. Expression values were converted to Transcripts Per Million (TPM) and log2-transformed [log2(TPM + 1)] for downstream analysis. The CIBERSORT algorithm with the LM22 signature matrix was employed to quantify tumor-infiltrating immune cells. Only samples with a CIBERSORT P-value < 0.05 were included. Functional enrichment, including KEGG and GSEA, was performed using the clusterProfiler R package. An adjusted P-value (FDR) < 0.05 was considered statistically significant. Correlations between gene expression and immune features were assessed using Pearson’s correlation. Patients were stratified into high- and low-expression groups based on optimal cutoffs determined by the survminer package. Survival outcomes (OS and PFS) were evaluated using Kaplan-Meier curves with the log-rank test. Univariate and multivariate Cox proportional hazards models were constructed to determine independent prognostic factors. All analyses were conducted using R software (v4.1.0).

### Specimen collection and Immunofluorescence (IF) staining

Paired primary and metastatic LUAD tissues were obtained from Fudan University Affiliated Cancer Hospital with written informed consent. The study was approved by the Institutional Ethics Committee (Approval No. 050432-4-2307E). FFPE sections (4 µm) were deparaffinized, rehydrated, and subjected to antigen retrieval in citrate buffer (pH 6.0). To eliminate tissue autofluorescence—a critical step for lung imaging—sections were treated with TrueBlack® Lipofuscin Autofluorescence Quencher (Cat# 23007, Biotium). Following blocking with 5% normal goat serum (CST), sections were incubated overnight at 4 °C with rabbit anti-UPP1 (1:200, Proteintech, Cat# 14186-1-AP) and mouse anti-CD68 (1:200, Abcam, Cat# ab955). Target proteins were visualized using Alexa Fluor 488- (for UPP1) and Alexa Fluor 594- (for CD68) conjugated secondary antibodies (1:500, Invitrogen). Nuclei were counterstained with DAPI. Images were acquired using a Leica SP8 Confocal Laser Scanning Microscope. Co-localization was analyzed using ImageJ software.

### Establishment of brain-tropic sublines and transcriptomic profiling

To generate brain-tropic lung cancer sublines with high metastatic potential, we employed an iterative in vivo selection strategy. Parental cells were injected into the left ventricle of BALB/c nude mice, and brain metastasis formation was monitored via 7.0 T MRI (Bruker). Brain lesions were harvested, cultured, and re-injected for four consecutive rounds to establish the high-metastatic subline (designated as -BrM4).

For transcriptomic characterization, total RNA was extracted from parental and BrM4 cells using the RNeasy Mini Kit (Cat# 74104, Qiagen) and quality-controlled via an Agilent 2100 Bioanalyzer. Sequencing libraries constructed with the NEBNext® Ultra™ II RNA Library Prep Kit (Cat# E7770, NEB) were sequenced on an Illumina NovaSeq 6000 platform (150-bp paired-end). Raw reads were aligned to the human reference genome (GRCh38) using STAR. DEGs were identified using DESeq2 (adjusted *P* < 0.05, |log2FoldChange | > 1). Hierarchical clustering heatmaps of Z-score normalized expression values were visualized using the ComplexHeatmap R package based on Euclidean distance and Ward’s linkage method.

### Cell lines and cell culture

Human LUAD cell line A549, human monocyte cell line THP-1, and mouse Lewis Lung Carcinoma (LLC) cells were obtained from the American Type Culture Collection (ATCC, Manassas, VA, USA). A549 and LLC cells were cultured in DMEM (Gibco, Thermo Fisher Scientific, Waltham, MA, USA) supplemented with 10% Fetal Bovine Serum (FBS; Gibco) and 1% penicillin-streptomycin (P/S; HyClone, Logan, UT, USA). THP-1 cells were maintained in RPMI-1640 medium (Gibco) containing 10% FBS and 0.05 mM 2-mercaptoethanol.

Bone Marrow-Derived Macrophages (BMDMs): Bone marrow cells were isolated from the femurs and tibias of 6–8 week-old C57BL/6 mice. Cells were differentiated into macrophages in DMEM supplemented with 10% FBS and 20 ng/mL recombinant mouse M-CSF (Cat# 315-02, PeproTech, Cranbury, NJ, USA) for 7 days.

### Reagents and antibodies

#### Inhibitors and agonists

MitoTEMPO (Mitochondrial ROS scavenger, Cat# SML0737) was purchased from Sigma-Aldrich. The cGAS inhibitor RU.521 (Cat# S6841) and Caspase-1 inhibitor VX-765 (Cat# S2228) were purchased from Selleck Chemicals (Houston, TX, USA). Recombinant Human IL-1β (Cat# 200-01B) was obtained from PeproTech.

#### Antibodies

Primary antibodies used for Western Blotting are listed in Supplementary Table S1. All primary antibodies were diluted in 5% BSA or specific signal enhancer solutions. HRP-linked anti-rabbit IgG (#7074, CST) and anti-mouse IgG (#7076, CST) secondary antibodies were used at a 1:3000 dilution for Western Blotting detection.

### Plasmids, lentiviral transduction, and RNA interference

Full-length human UPP1 and mouse Upp1 cDNAs were cloned into the pCDH-CMV-MCS-EF1-Puro lentiviral vector (System Biosciences, Palo Alto, CA, USA). For knockdown experiments, Short hairpin RNAs (shRNAs) targeting NLRP3, GSDMD, and a scrambled control (shNC) were synthesized by GeneChem (Shanghai, China) and cloned into the pLKO.1 vector.

Lentiviral particles were generated by co-transfecting HEK293T cells with the target plasmid and packaging plasmids (psPAX2 and pMD2.G) using Lipofectamine 3000 (Invitrogen, Carlsbad, CA, USA). Viral supernatants were collected at 48 h and 72 h, filtered, and used to infect macrophages in the presence of 8 µg/mL polybrene (Sigma). Stable cell lines were selected using 2 µg/mL Puromycin (Gibco) for 7 days. The specific target sequences for all shRNAs used in this study are detailed in Supplementary Table S2.

### Cell viability and proliferation assay

Cell proliferation was monitored using the Cell Counting Kit-8 (CCK-8) assay (Cat# CK04, Dojindo, Kumamoto, Japan). Briefly, tumor cells (pre-treated or co-cultured) were seeded into 96-well plates at a density of 2 × 10^3^ cells per well. At indicated time points (0, 24, 48, and 72 h), 10 µL of CCK-8 reagent was added to each well, and cells were incubated at 37 °C for 2 h. The absorbance at 450 nm was measured using a microplate reader.

### Tumor sphere formation assay

To evaluate cancer stem-like properties, a sphere formation assay was performed in Ultra-Low Attachment (ULA) 6-well plates (Cat# 3471, Corning, NY, USA). Single-cell suspensions were seeded at a density of 1×10^3^ cells/well and cultured in serum-free DMEM/F12 medium (Gibco) supplemented with 2% B27 Supplement (Cat# 17504044, Gibco), 20 ng/mL EGF (Cat# AF-100-15, PeproTech), and 20 ng/mL bFGF (Cat# 100-18B, PeproTech). Fresh medium was supplemented every 3 days. After 10–14 days, spheres with a diameter > 50 µm were counted and imaged under an inverted microscope.

### Mitochondrial ROS (mtROS)

Cells were incubated with 5 µM MitoSOX™ Red Mitochondrial Superoxide Indicator (Cat# M36008, Invitrogen) for 15 min at 37 °C. Cells were then washed with PBS and analyzed by flow cytometry (BD FACSCanto II, BD Biosciences). Mean fluorescence intensity (MFI) was quantified using FlowJo software.

### Mitochondrial Membrane Potential (MMP)

MMP was assessed using the JC-1 Mitochondrial Membrane Potential Assay Kit (Cat# C2006, Beyotime, Shanghai, China). Cells were stained with JC-1 working solution for 20 min. The ratio of red (aggregates) to green (monomers) fluorescence was determined using a fluorescence microplate reader (BioTek) or flow cytometry to assess mitochondrial depolarization.

### Measurement of intracellular PRPP levels

The intracellular concentration of Phosphoribosyl pyrophosphate (PRPP) was quantified using a specific PRPP ELISA Kit (Cat# MBS702739, MyBioSource, San Diego, CA, USA) according to the manufacturer’s instructions. Briefly, 5 × 10^6^ cells were harvested, washed with PBS, and lysed by repeated freeze-thaw cycles. The supernatants were collected after centrifugation, and PRPP levels were determined by measuring absorbance at 450 nm using a microplate reader. The concentration of PRPP (ng/mL) in the samples was calculated based on a standard curve.

### NAD + /NADH ratio assay

The intracellular NAD + /NADH redox state was assessed using the NAD/NADH Quantitation Colorimetric Kit (Cat# ab65348, Abcam, Cambridge, UK). Cells were lysed in NADH/NAD Extraction Buffer and centrifuged at 10,000 × g for 5 min. To distinguish between NADH and NAD + , the total NAD (NADt) was measured directly, while a portion of the lysate was heated at 60 °C for 30 min to decompose NAD+ for the specific quantification of NADH. The NAD+ level was calculated as (Total NAD − NADH). The ratio of NAD + /NADH was determined by measuring absorbance at 450 nm.

### Cytosolic mtDNA extraction and analysis

To quantify cytosolic mtDNA, subcellular fractionation was performed as previously described. Briefly, cells were resuspended in mild lysis buffer containing 150 mM NaCl, 50 mM HEPES (pH 7.4), and 25 µg/mL digitonin (Sigma) to selectively permeabilize the plasma membrane without disrupting mitochondria. The lysates were centrifuged at 1000 × g for 5 min to pellet nuclei and intact mitochondria. The supernatant (cytosolic fraction) was collected, and DNA was extracted using the QIAamp DNA Mini Kit (Qiagen). The abundance of mitochondrial DNA (MT-CO1) was measured by qPCR and normalized to nuclear DNA control, 18S for human samples and Tert for mouse samples.

### RNa isolation and quantitative real-time PCR (qRT-PCR)

Total RNA was extracted using TRIzol reagent (Invitrogen) according to the manufacturer’s protocol. 1 µg of total RNA was reverse-transcribed into cDNA using the PrimeScript™ RT Reagent Kit (Cat# RR037A, Takara, Shiga, Japan). Quantitative PCR was performed using TB Green® Premix Ex Taq™ II (Cat# RR820A, Takara) on an ABI 7500 Real-Time PCR System (Applied Biosystems). Relative gene expression was calculated using the 2 − ΔΔCt method, normalized to GAPDH. The specific primer sequences are listed in Supplementary Table S3.

### Western blotting and ASC oligomerization

Cells were lysed in RIPA buffer supplemented with protease and phosphatase inhibitor cocktails (Roche). Protein concentration was determined using the BCA Protein Assay Kit (Thermo Fisher). 20–40 µg of protein was separated by SDS-PAGE and transferred to PVDF membranes (Millipore). For ASC oligomerization detection, cells were lysed, and the pellets were cross-linked with disuccinimidyl suberate (DSS, 2 mM, Sigma) for 30 min at room temperature before electrophoresis. Membranes were blocked with 5% non-fat milk and incubated with primary antibodies overnight at 4 °C, followed by HRP-conjugated secondary antibodies. Bands were visualized using an ECL detection system (Bio-Rad).

### LDH release and ELISA

Cell death (Pyroptosis) was evaluated by measuring lactate dehydrogenase (LDH) release in the culture supernatant using the CytoTox 96® Non-Radioactive Cytotoxicity Assay (Cat# G1780, Promega, Madison, WI, USA).

The concentration of IL-1β in the cell culture supernatants or mouse serum was quantified using the Human IL-1β Quantikine ELISA Kit (Cat# DLB50, R&D Systems, Minneapolis, MN, USA) or Mouse IL-1β ELISA Kit (Cat# MLB00C, R&D Systems) according to the manufacturer’s instructions.

### Immunohistochemistry (IHC)

Formalin-fixed, paraffin-embedded (FFPE) tissue sections (4 µm) were deparaffinized and rehydrated. Antigen retrieval was performed by boiling in sodium citrate buffer (pH 6.0). Sections were blocked with 3% H2O2 and 5% goat serum, then incubated with primary antibodies against Ki67 or Vimentin overnight at 4 °C (Supplementary Table S1). Detection was carried out using the EnVision™+ HRP-DAB System (Dako). Sections were counterstained with hematoxylin. Staining intensity was quantified using the H-score method based on the intensity and percentage of positive cells. All histological and IHC evaluations were performed by two independent pathologists who were blinded to the experimental groupings.

### Tumor cell migration and invasion assays

#### Co-culture system

Tumor cells (A549/LLC) were seeded in the lower chamber of a Transwell plate (0.4 µm pore size; Corning), while macrophages (Control or UPP1-OE) were seeded in the upper chamber inserts.

#### Migration/Invasion

For direct migration assays, tumor cells pre-treated with macrophage-conditioned medium (CM) were seeded into the upper chamber of Transwell inserts (8.0 µm pore size; Corning). For invasion assays, the inserts were pre-coated with Matrigel (Cat# 356234, BD Biosciences). Medium containing 20% FBS was added to the lower chamber as a chemoattractant. After 24 h (migration) or 48 h (invasion), cells remaining on the upper surface were removed, and migrated/invaded cells on the lower surface were fixed with methanol, stained with 0.1% crystal violet, and imaged.

### IL-1β neutralization

For rescue experiments, an IL-1β neutralizing antibody (1 µg/mL, Cat# MAB201, R&D Systems) or IgG isotype control was added to the co-culture system or conditioned medium.

### In vivo xenograft and metastasis models

All animal experiments were approved by the Institutional Animal Care and Use Committee (IACUC) of Fudan University.

Subcutaneous Co-injection Model: 5 × 10^5^ LLC cells mixed with 1 × 10^5^ BMDMs (Vector or UPP1-OE) were suspended in 100 µL PBS/Matrigel (1:1) and injected subcutaneously into the right flank of 6-week-old male C57BL/6 mice (*n* = 5/group). Tumor volume was measured every 3 days using calipers (V = 0.5 × Length × Width^2^).

#### Spontaneous metastasis

Mice from the subcutaneous model were sacrificed at the endpoint (Day 28). Lungs were excised and fixed in Bouin’s solution for 24 h to enhance the contrast of metastatic nodules. The number of surface metastatic nodules was independently counted by two researchers in a blinded manner under a dissecting microscope. To ensure consistency, only macroscopically visible nodules with a diameter > 0.5 mm were included in the quantification. After visual counting, lung tissues were embedded in paraffin and sectioned at 4 µm. Hematoxylin and eosin (H&E) staining was performed to examine tissue architecture and confirm metastatic adenocarcinoma lesions based on typical histopathological features. Immunohistochemical (IHC) staining for Ki67 and Vimentin was subsequently performed to evaluate proliferative activity and mesenchymal characteristics, respectively, further supporting the identification of metastatic tumor foci and distinguishing them from potential inflammatory or reactive lesions.

#### Bio-luminescence imaging

To monitor metastasis in real-time, LLC cells expressing Luciferase were used. Mice were injected intraperitoneally with D-Luciferin (150 mg/kg, PerkinElmer) and imaged using the IVIS Spectrum System (PerkinElmer). Signal intensity was quantified as Total Flux (photons/s) in the thoracic region of interest (ROI).

### In vivo experimental design and ARRIVE guidelines

#### All animal experiments were performed and reported in accordance with the ARRIVE (Animal Research

Reporting of In Vivo Experiments) guidelines.

#### Sample size justification

The sample size (*n* = 5 per group) was determined based on preliminary experiments and previous experience with the LLC model to ensure adequate statistical power to detect meaningful differences in tumor growth and metastasis.

#### Randomization

Mice were randomly assigned to experimental groups using a simple random allocation method (computer-generated random numbers).

#### Blinding

To minimize bias, researchers responsible for tumor volume measurements and lung nodule counting were blinded to the group assignments throughout the study. Group identities were only unmasked during the final data analysis.

### Statistical Analysis

Statistical analyses were performed using GraphPad Prism 9.0 (GraphPad Software, San Diego, CA, USA). Data are presented as mean ± Standard Deviation (SD) from at least three independent experiments. Differences between two groups were analyzed using the two-tailed Student’s t-test. Multigroup comparisons were performed using One-way or Two-way ANOVA followed by Tukey’s post hoc test. Correlation analysis was performed using Pearson’s or Spearman’s correlation coefficients. A *P*-value < 0.05 was considered statistically significant.

## Supplementary information

Figure S1

Figure S2

Figure S3

Figure S4

Figure S5

Figure S6

Suppl. Fig. legends

Supplementary Table

Supplement WB

## Data Availability

The datasets generated and analysed during the current study are not publicly available due to data ownership and confidentiality considerations but are available from the corresponding author on reasonable request. The original Western blot images can be found in Supplementary WB.
