# Supplementary material for: Upregulation of macrophage UPP1 promotes lung adenocarcinoma metastasis through an mtROS-cGAS-NLRP3 inflammasome axis
Source: Cell Death Discov. 2026 Jul 6;12:385. doi: 10.1038/s41420-026-03226-4 (PMC13620140; doi:10.1038/s41420-026-03226-4)
Supplement: Supplementary file 1 [file 41420_2026_3226_MOESM1_ESM.pdf]

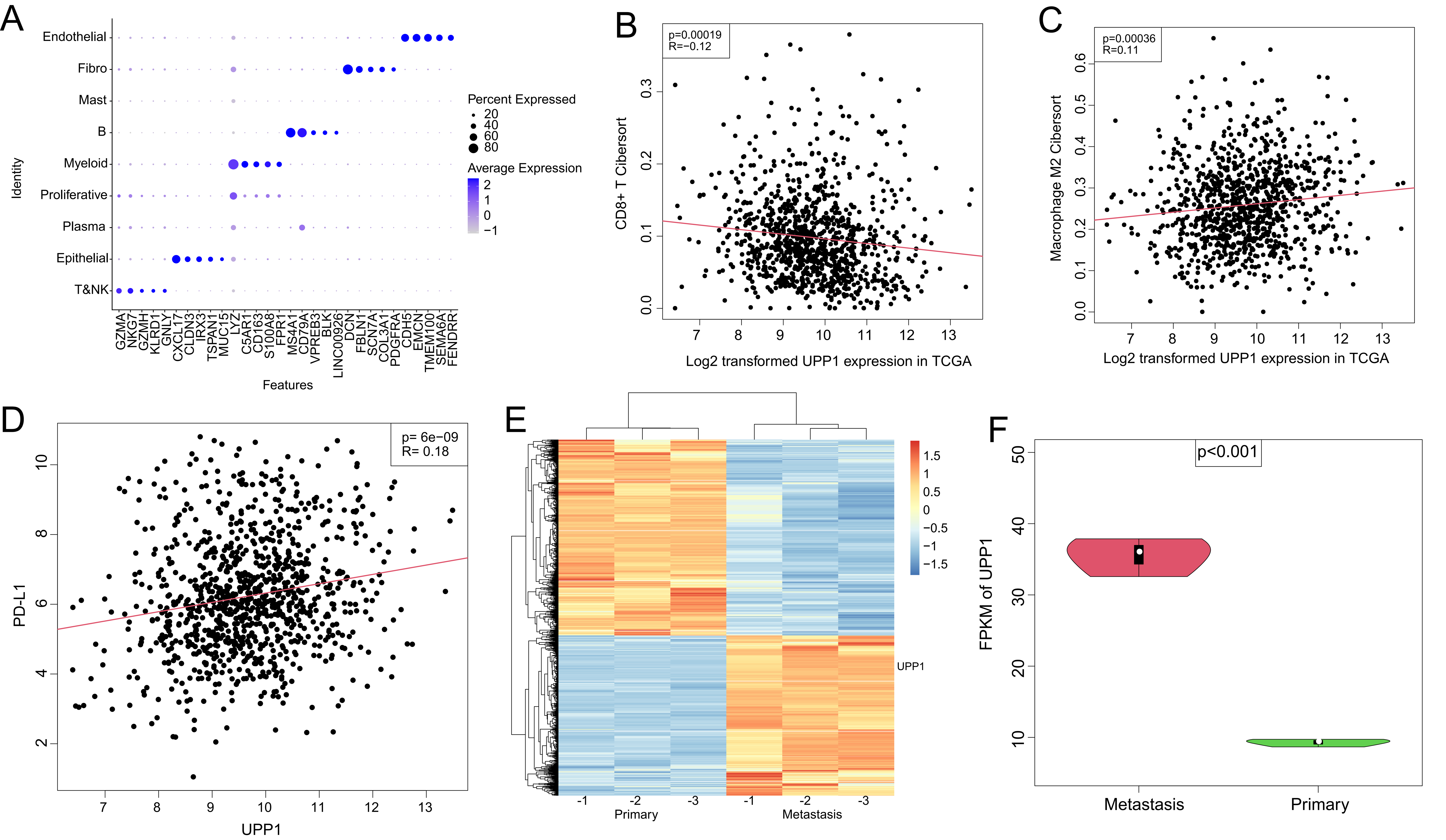
A
Endothelial
Fibro
Mast
B
Myeloid
Proliferative
Plasma
Epithelial
T&NK
Identity
GZMA
NKG7
GZMH
KLRD1
GNLY
CXCL17
CLDN3
IRX3
TSPAN1
MUC15
LYZ
C5AR1
CD163
S100A8
FPR1
MS4A1
CD79A
VPREB3
BLK
LINC00926
DCN
FBLN1
SCN7A
COL3A1
PDGFRA
CDH5
EMCN
TMEM100
SEMA6A
FENDRR
Features
Percent Expressed
20
40
60
80
Average Expression
2
1
0
−1
B
p=0.00019
R=−0.12
CD8+ T Cibersort
Log2 transformed UPP1 expression in TCGA
C
p=0.00036
R=0.11
Macrophage M2 Cibersort
Log2 transformed UPP1 expression in TCGA
D
p= 6e−09
R= 0.18
PD-L1
UPP1
E
1.5
1
0.5
0
−0.5
−1
−1.5
UPP1
-1
-2
-3
Primary
-1
-2
-3
Metastasis
F
p<0.001
FPKM of UPP1
Metastasis
Primary
