# Supplementary material for: Upregulation of macrophage UPP1 promotes lung adenocarcinoma metastasis through an mtROS-cGAS-NLRP3 inflammasome axis
Source: Cell Death Discov. 2026 Jul 6;12:385. doi: 10.1038/s41420-026-03226-4 (PMC13620140; doi:10.1038/s41420-026-03226-4)
Supplement: Supplementary file 2 [file 41420_2026_3226_MOESM2_ESM.pdf]

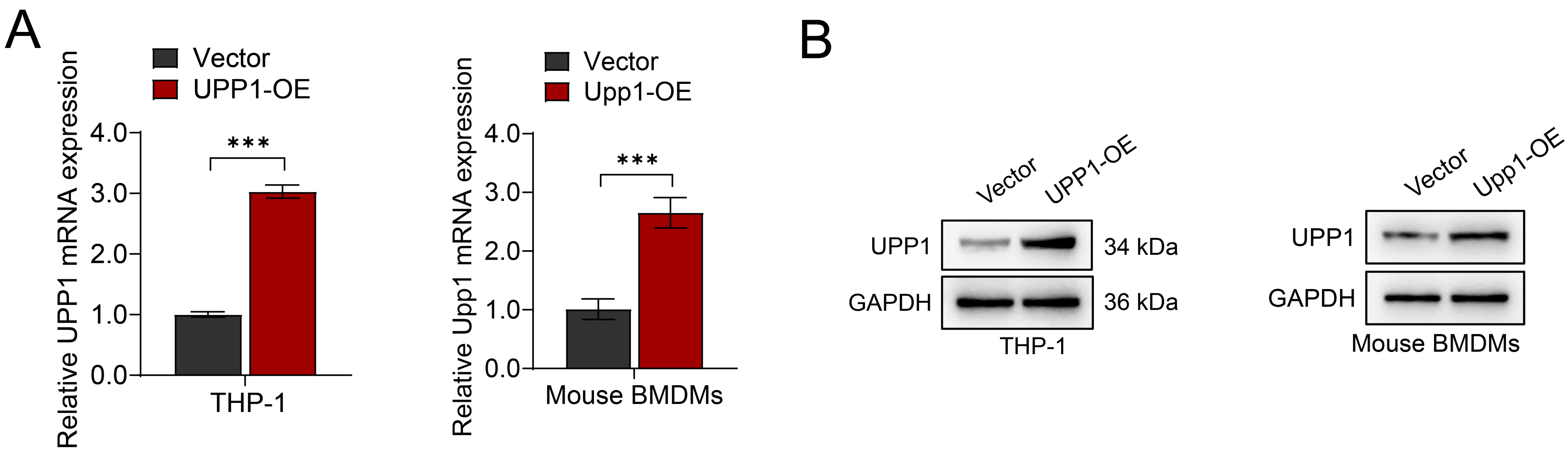
A
Vector
UPP1-OE
Relative UPP1 mRNA expression
4.0
3.0
2.0
1.0
0.0
***
THP-1
Vector
Upp1-OE
Relative Upp1 mRNA expression
4.0
3.0
2.0
1.0
0.0
***
Mouse BMDMs
B
Vector
UPP1-OE
UPP1
34 kDa
GAPDH
36 kDa
THP-1
Vector
Upp1-OE
UPP1
GAPDH
Mouse BMDMs
