# Supplementary material for: Upregulation of macrophage UPP1 promotes lung adenocarcinoma metastasis through an mtROS-cGAS-NLRP3 inflammasome axis
Source: Cell Death Discov. 2026 Jul 6;12:385. doi: 10.1038/s41420-026-03226-4 (PMC13620140; doi:10.1038/s41420-026-03226-4)
Supplement: Supplementary file 3 [file 41420_2026_3226_MOESM3_ESM.pdf]

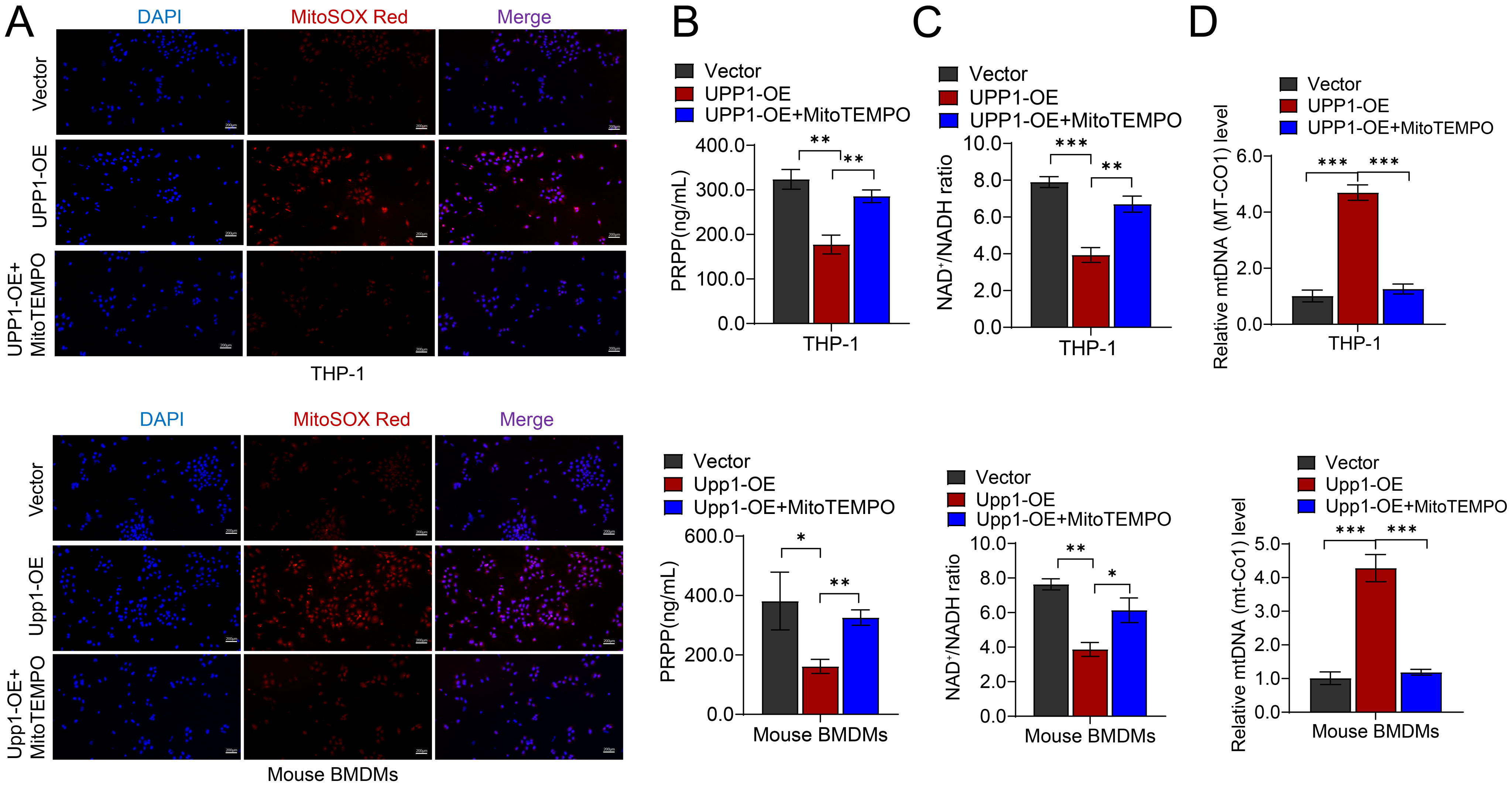
A
B
C
D
DAPI
MitoSOX Red
Merge
Vector
UPP1-OE
UPP1-OE+
MitoTEMPO
THP-1
Vector
UPP1-OE
UPP1-OE+MitoTEMPO
PRPP(ng/mL)
400.0
300.0
200.0
100.0
0.0
THP-1
NAD+/NADH ratio
10.0
8.0
6.0
4.0
2.0
0.0
Relative mtDNA (MT-CO1) level
6.0
4.0
2.0
0.0
DAPI
MitoSOX Red
Merge
Vector
Upp1-OE
Upp1-OE+
MitoTEMPO
Mouse BMDMs
Vector
Upp1-OE
Upp1-OE+MitoTEMPO
600.0
400.0
200.0
0.0
Relative mtDNA (mt-Co1) level
5.0
4.0
3.0
2.0
1.0
0.0
Mouse BMDMs
