# Supplementary material for: Upregulation of macrophage UPP1 promotes lung adenocarcinoma metastasis through an mtROS-cGAS-NLRP3 inflammasome axis
Source: Cell Death Discov. 2026 Jul 6;12:385. doi: 10.1038/s41420-026-03226-4 (PMC13620140; doi:10.1038/s41420-026-03226-4)
Supplement: Supplementary file 4 [file 41420_2026_3226_MOESM4_ESM.pdf]

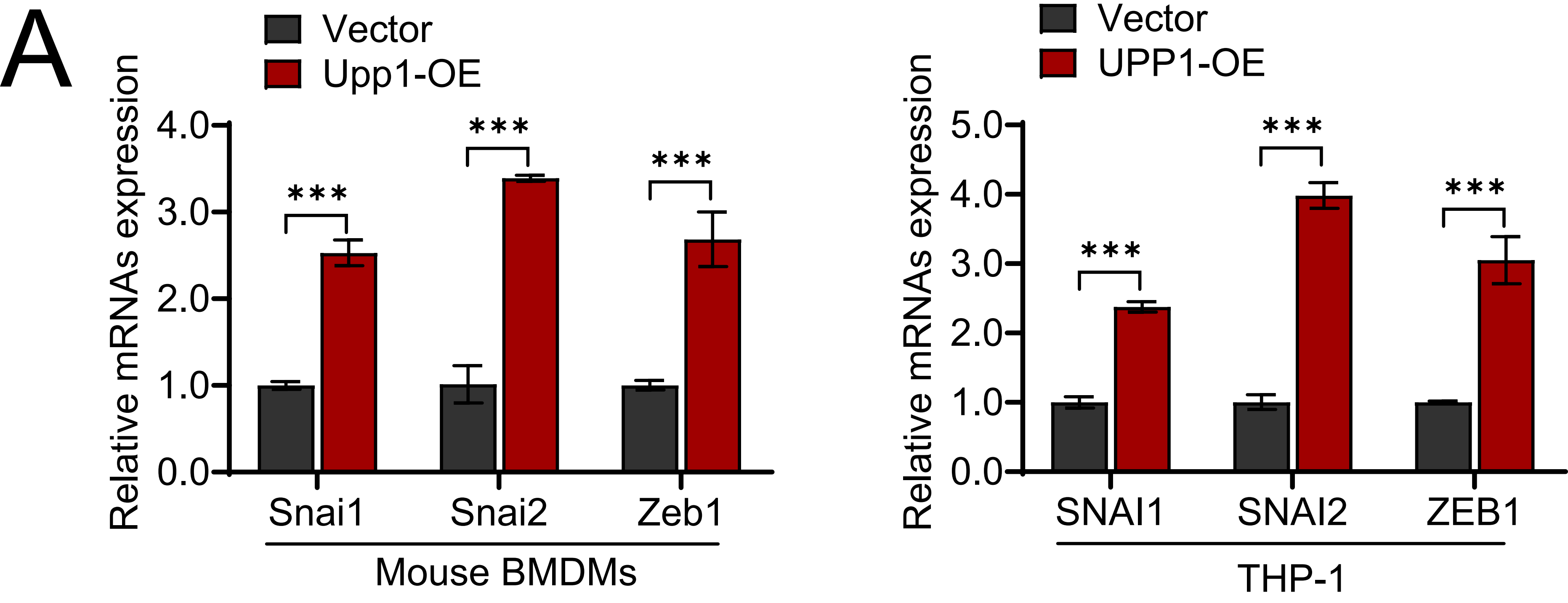

A
Vector
Upp1-OE
Relative mRNAs expression
4.0
3.0
2.0
1.0
0.0
***
***
***
Snai1
Snai2
Zeb1
Mouse BMDMs
Vector
UPP1-OE
Relative mRNAs expression
5.0
4.0
3.0
2.0
1.0
0.0
***
***
***
SNAI1
SNAI2
ZEB1
THP-1
