# Supplementary material for: Upregulation of macrophage UPP1 promotes lung adenocarcinoma metastasis through an mtROS-cGAS-NLRP3 inflammasome axis
Source: Cell Death Discov. 2026 Jul 6;12:385. doi: 10.1038/s41420-026-03226-4 (PMC13620140; doi:10.1038/s41420-026-03226-4)
Supplement: Supplementary file 5 [file 41420_2026_3226_MOESM5_ESM.pdf]

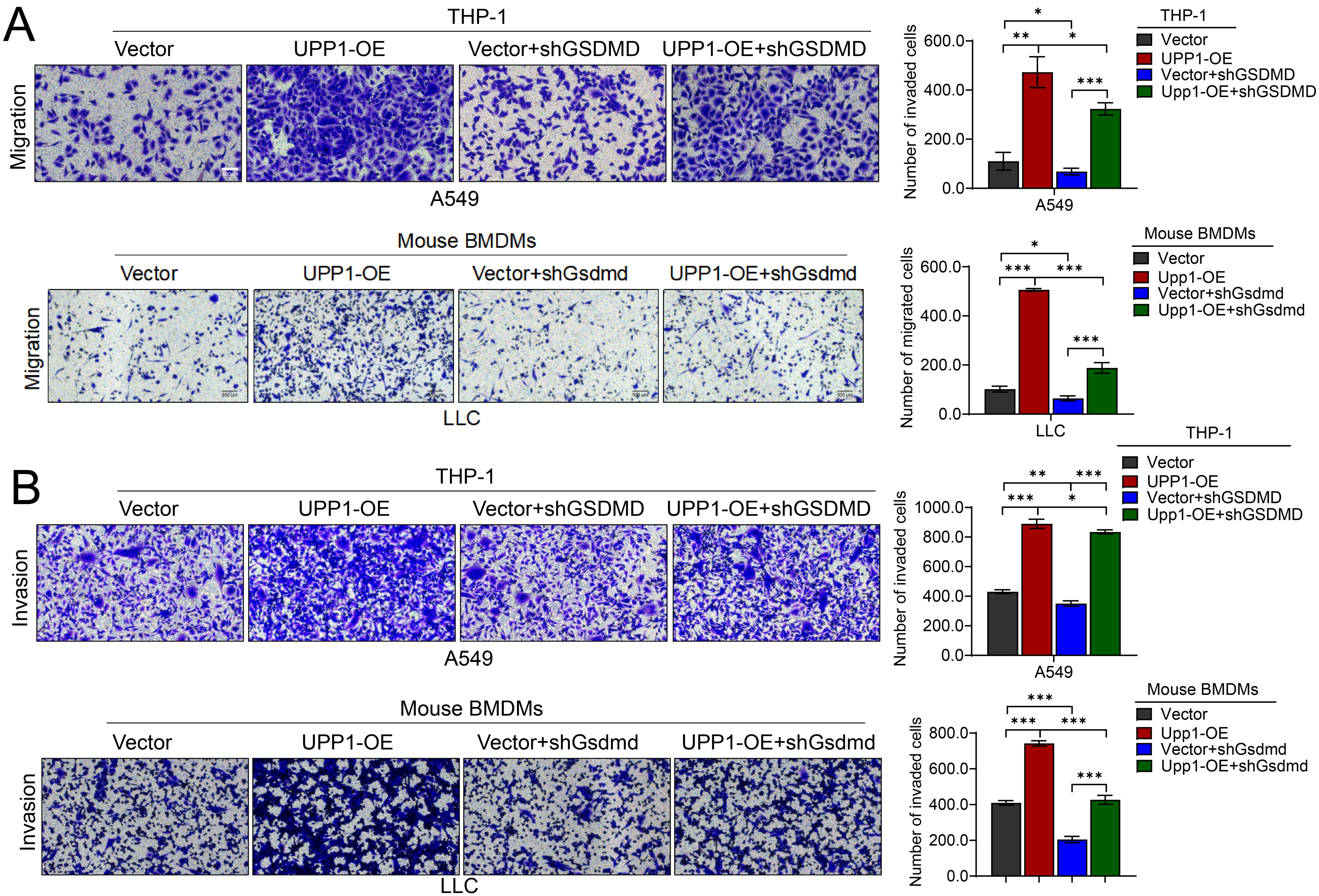
A
THP-1
Vector
UPP1-OE
Vector+shGSDMD
UPP1-OE+shGSDMD
Migration
A549
Number of invaded cells
600.0
400.0
200.0
0.0
A549
THP-1
Vector
UPP1-OE
Vector+shGSDMD
Upp1-OE+shGSDMD
Mouse BMDMs
Vector
UPP1-OE
Vector+shGsdmd
UPP1-OE+shGsdmd
Migration
LLC
Number of migrated cells
600.0
400.0
200.0
0.0
LLC
Mouse BMDMs
Vector
Upp1-OE
Vector+shGsdmd
Upp1-OE+shGsdmd
B
THP-1
Vector
UPP1-OE
Vector+shGSDMD
UPP1-OE+shGSDMD
Invasion
A549
Number of invaded cells
1000.0
800.0
600.0
400.0
200.0
0.0
A549
THP-1
Vector
UPP1-OE
Vector+shGSDMD
Upp1-OE+shGSDMD
Mouse BMDMs
Vector
UPP1-OE
Vector+shGsdmd
UPP1-OE+shGsdmd
Invasion
LLC
Number of invaded cells
800.0
600.0
400.0
200.0
0.0
Mouse BMDMs
Vector
Upp1-OE
Vector+shGsdmd
Upp1-OE+shGsdmd
