# Supplementary material for: Upregulation of macrophage UPP1 promotes lung adenocarcinoma metastasis through an mtROS-cGAS-NLRP3 inflammasome axis
Source: Cell Death Discov. 2026 Jul 6;12:385. doi: 10.1038/s41420-026-03226-4 (PMC13620140; doi:10.1038/s41420-026-03226-4)
Supplement: Supplementary file 6 [file 41420_2026_3226_MOESM6_ESM.pdf]

A

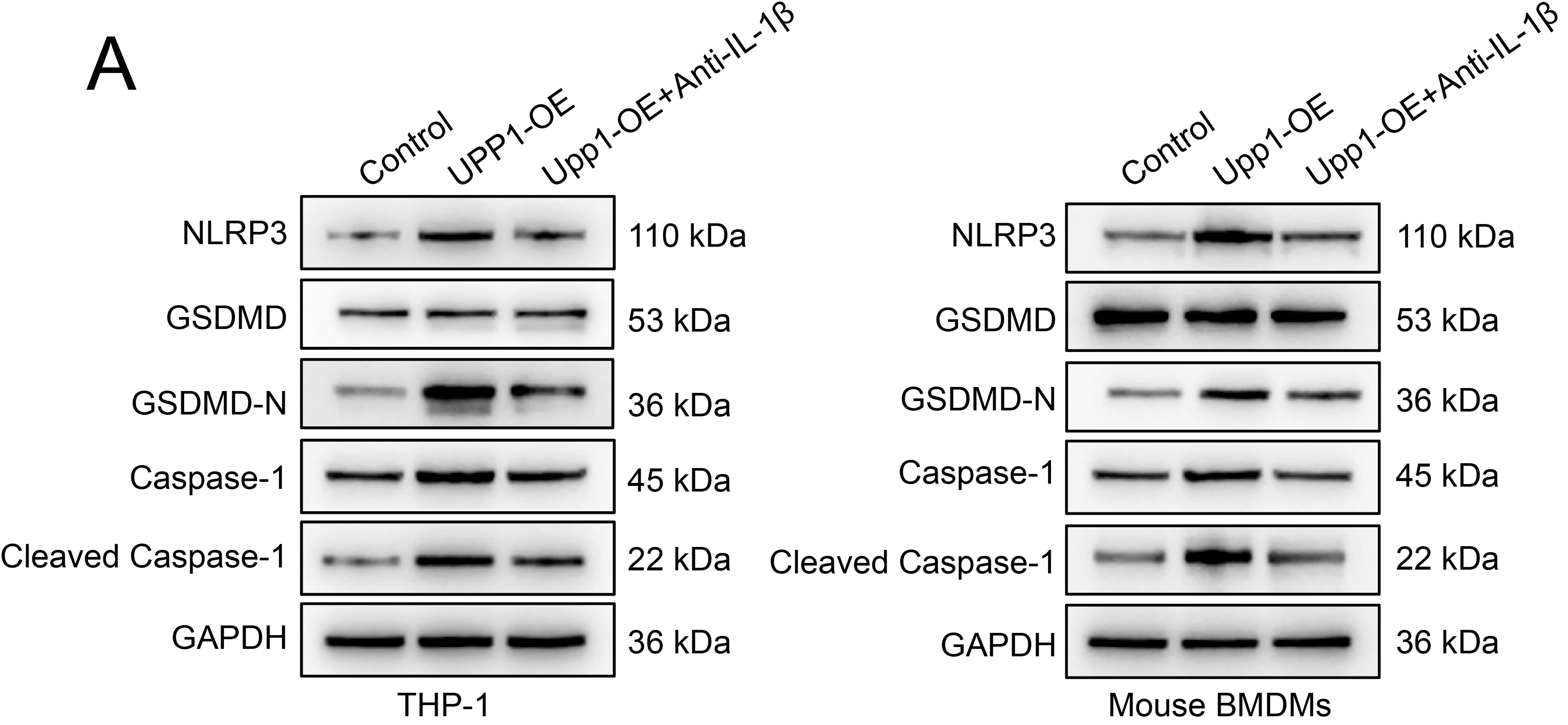
Control
UPP1-OE
Upp1-OE+Anti-IL-1β
NLRP3
110 kDa
GSDMD
53 kDa
GSDMD-N
36 kDa
Caspase-1
45 kDa
Cleaved Caspase-1
22 kDa
GAPDH
36 kDa
THP-1
Control
Upp1-OE
Upp1-OE+Anti-IL-1β
NLRP3
110 kDa
GSDMD
53 kDa
GSDMD-N
36 kDa
Caspase-1
45 kDa
Cleaved Caspase-1
22 kDa
GAPDH
36 kDa
Mouse BMDMs
