# Supplementary material for: Upregulation of macrophage UPP1 promotes lung adenocarcinoma metastasis through an mtROS-cGAS-NLRP3 inflammasome axis
Source: Cell Death Discov. 2026 Jul 6;12:385. doi: 10.1038/s41420-026-03226-4 (PMC13620140; doi:10.1038/s41420-026-03226-4)
Supplement: Supplementary file 7 [file 41420_2026_3226_MOESM9_ESM.pdf]

Marker

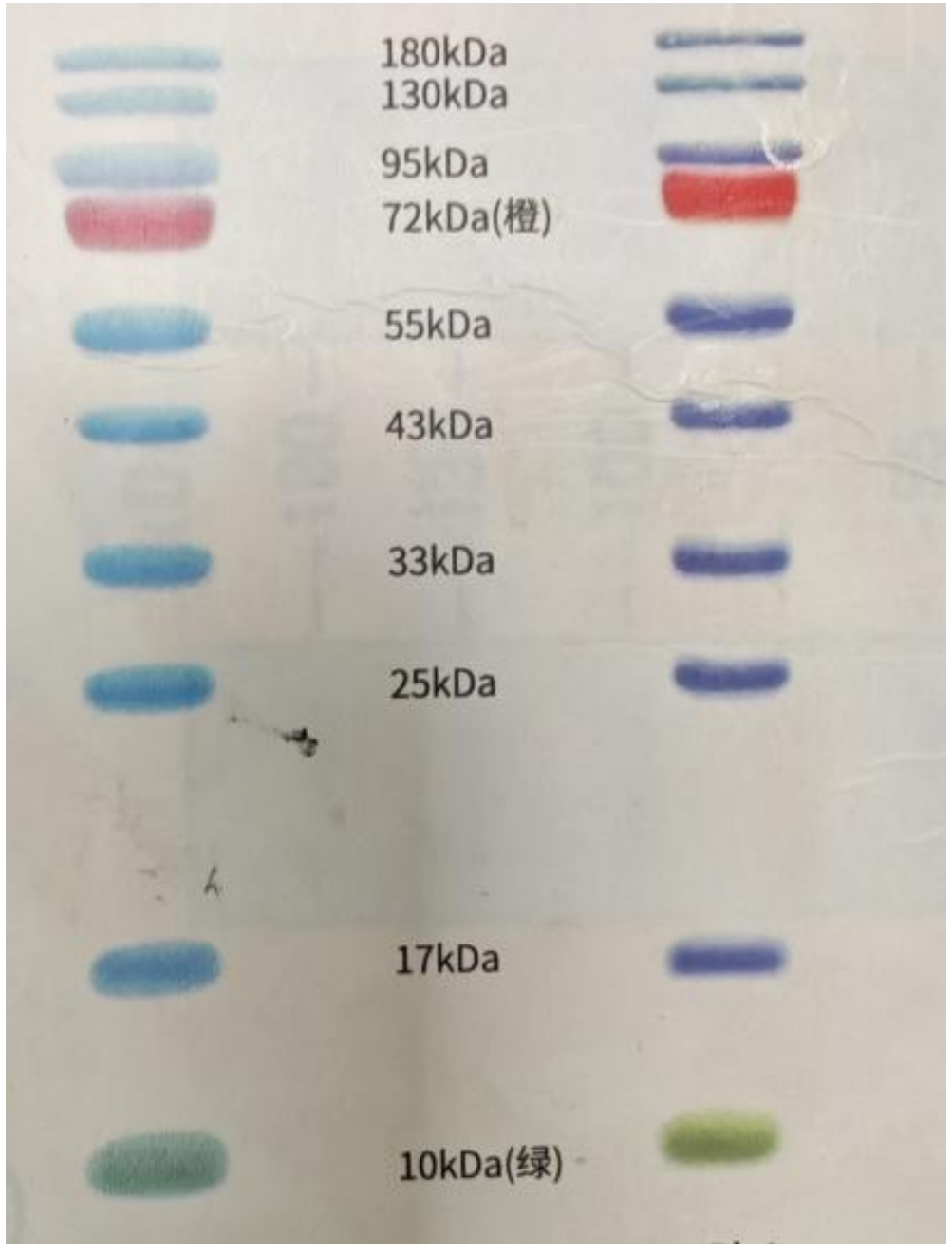

Figure 2F

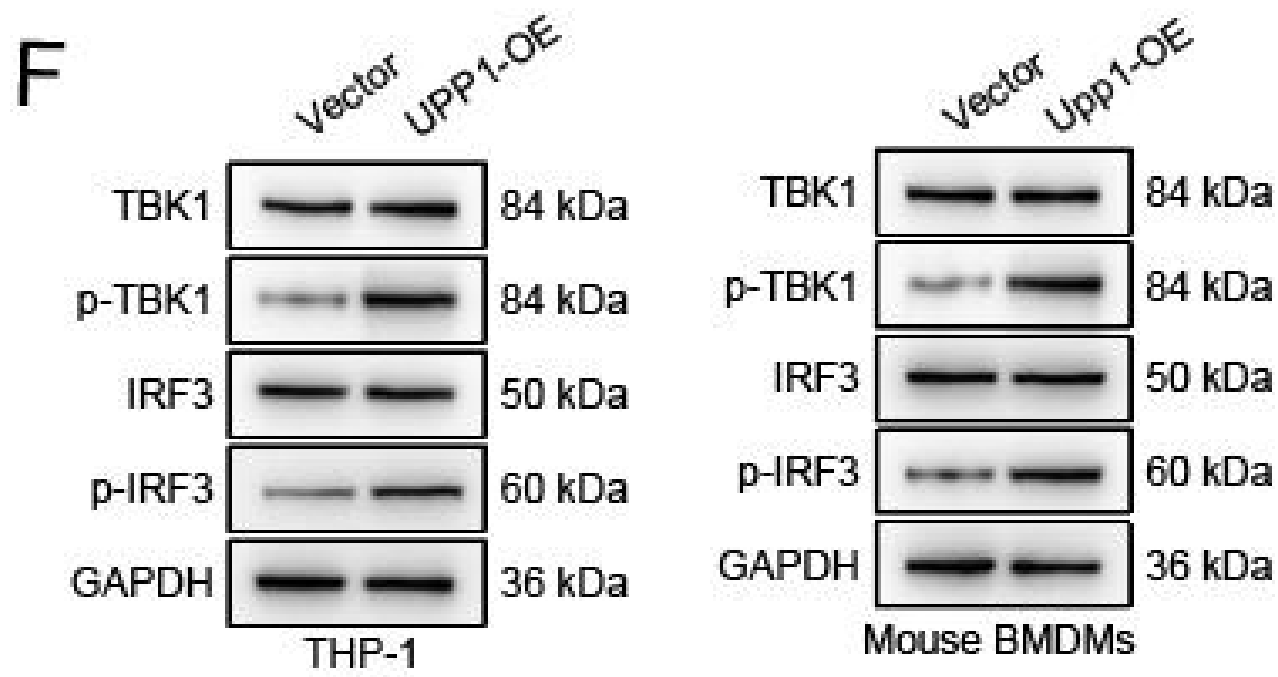

THP-1

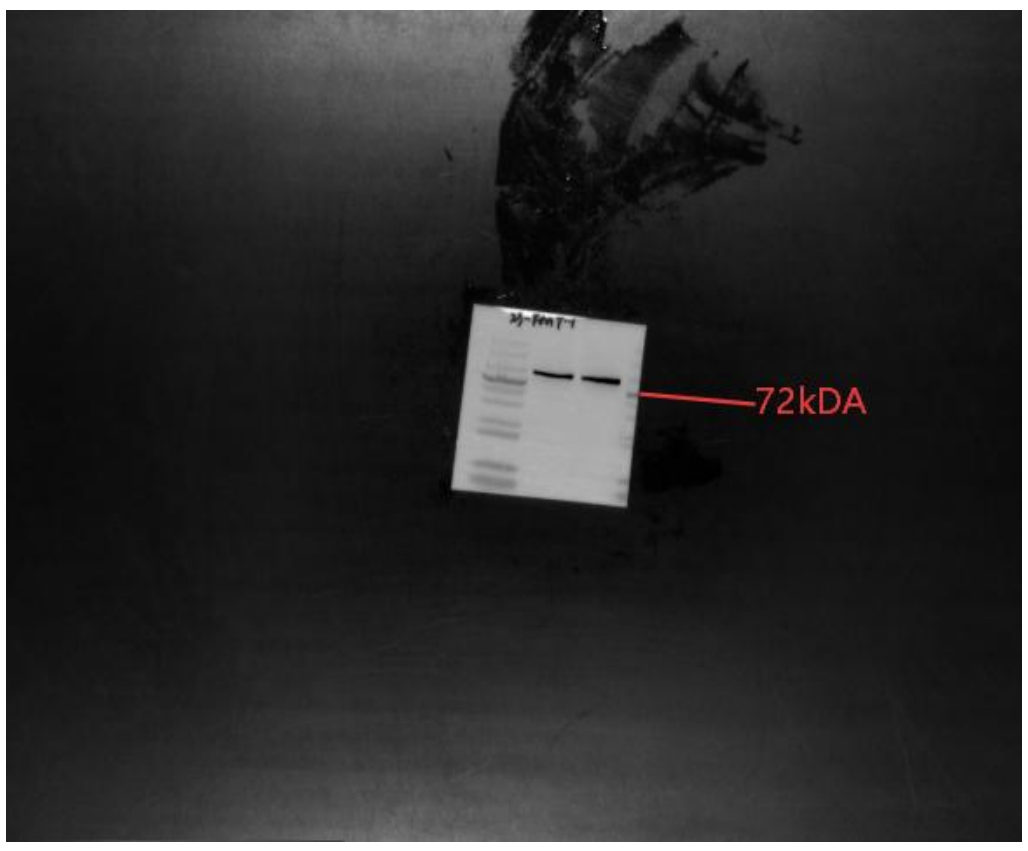

TBK1

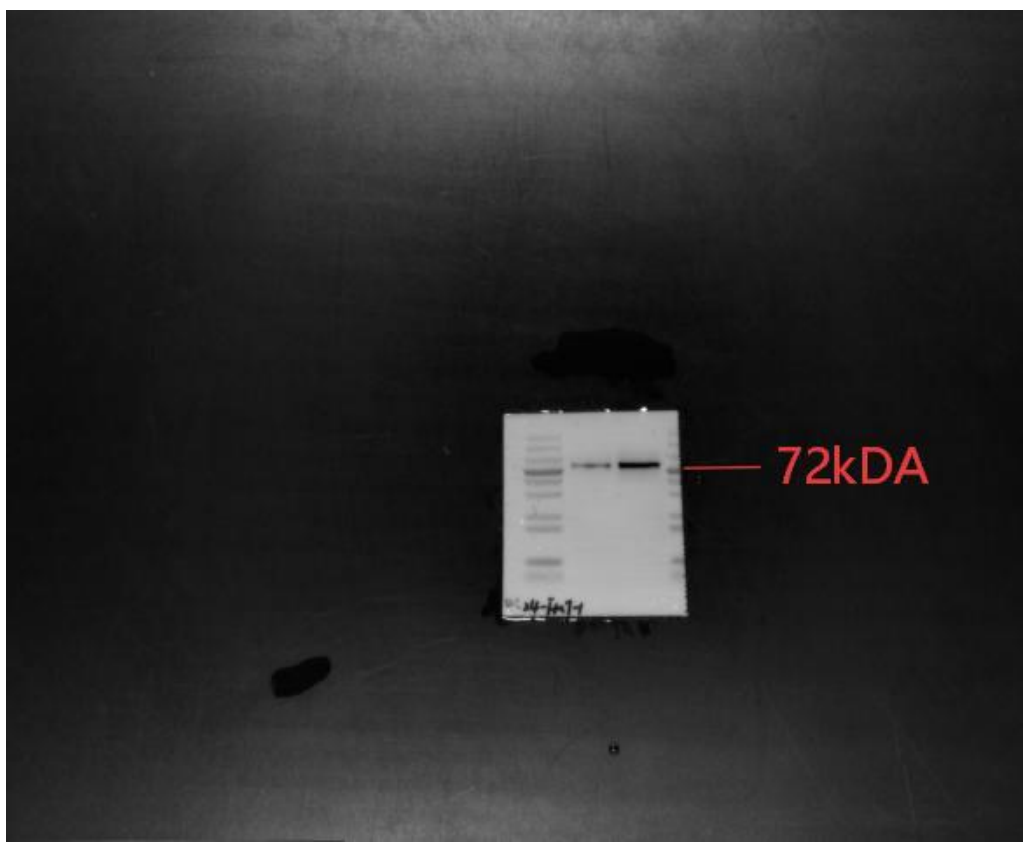

p-TBK1

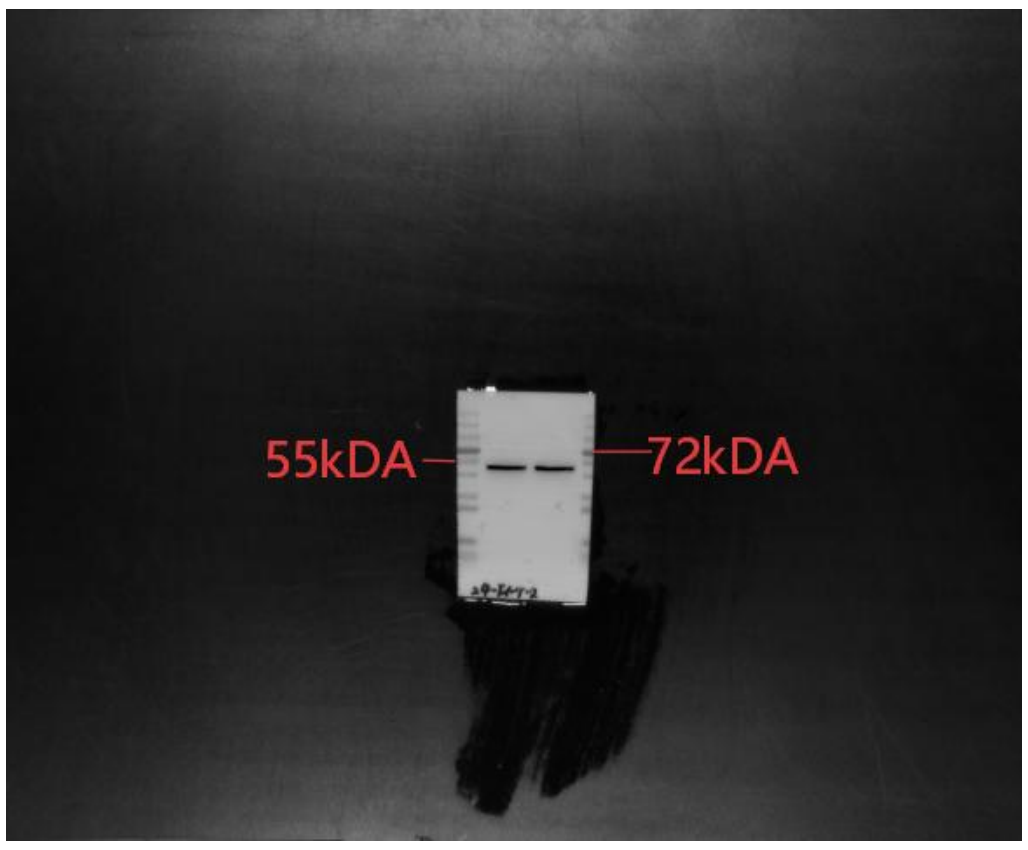

IRF3

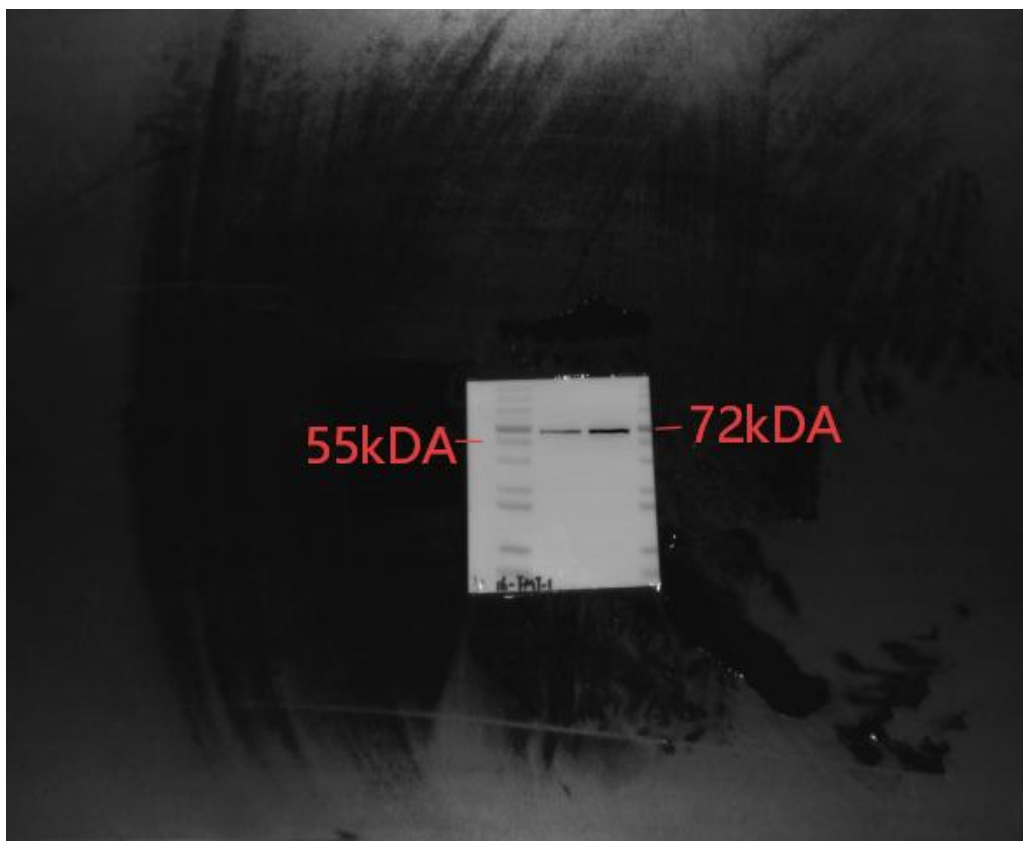

p-IRF3

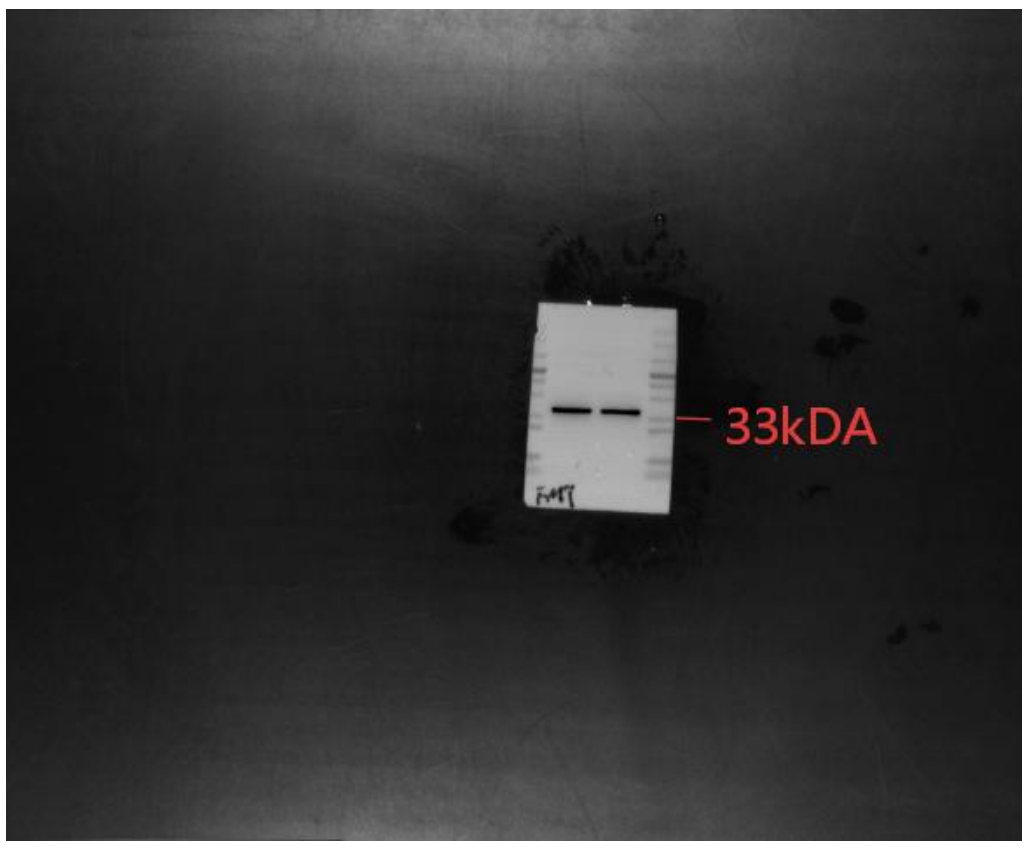

GAPDH

Mouse BMDMs

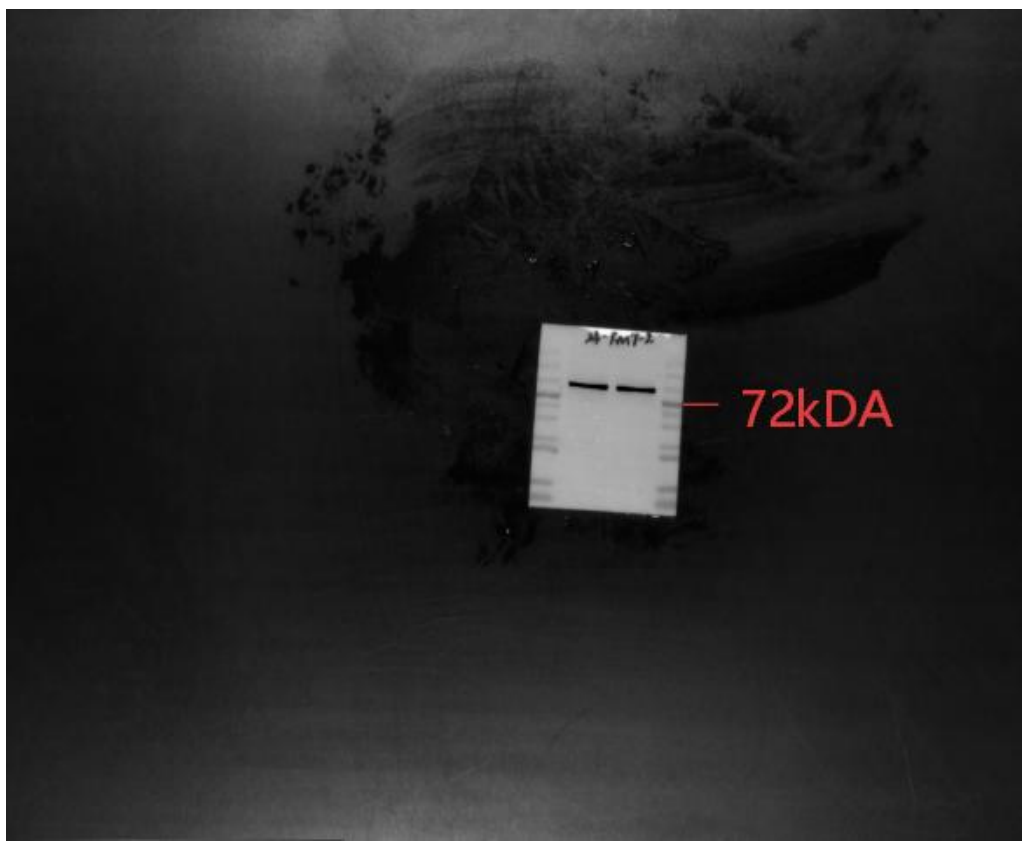

TBK1

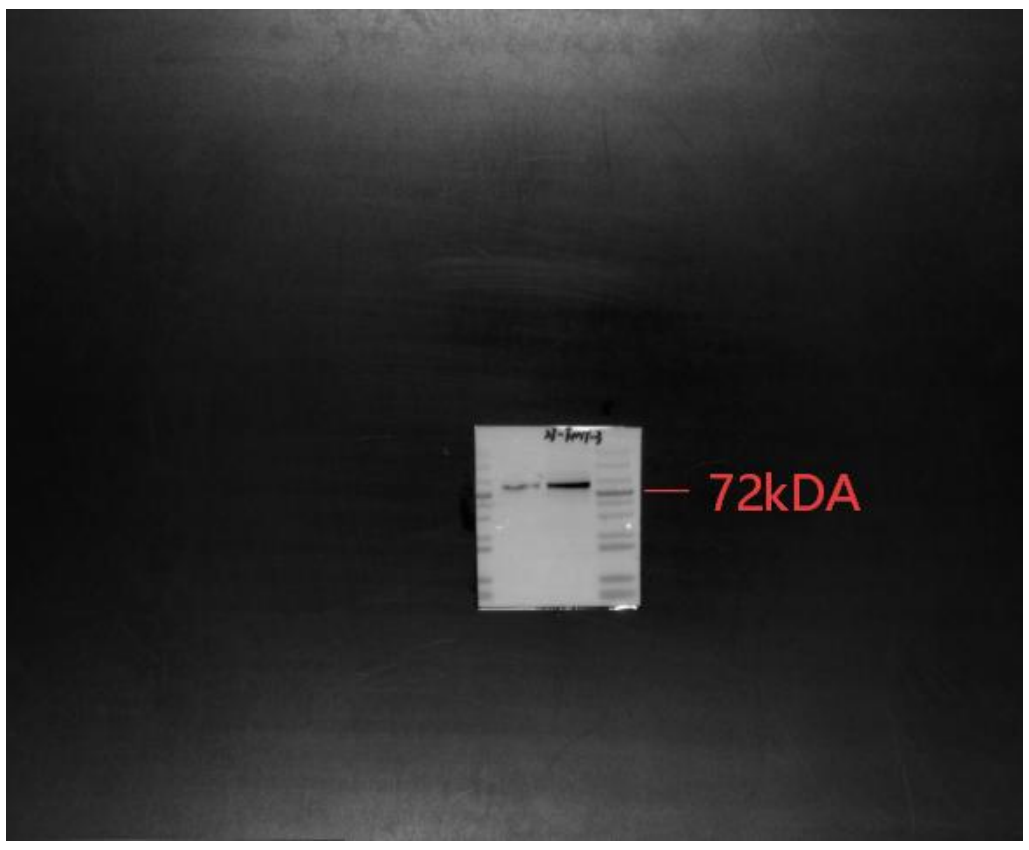

p-TBK1

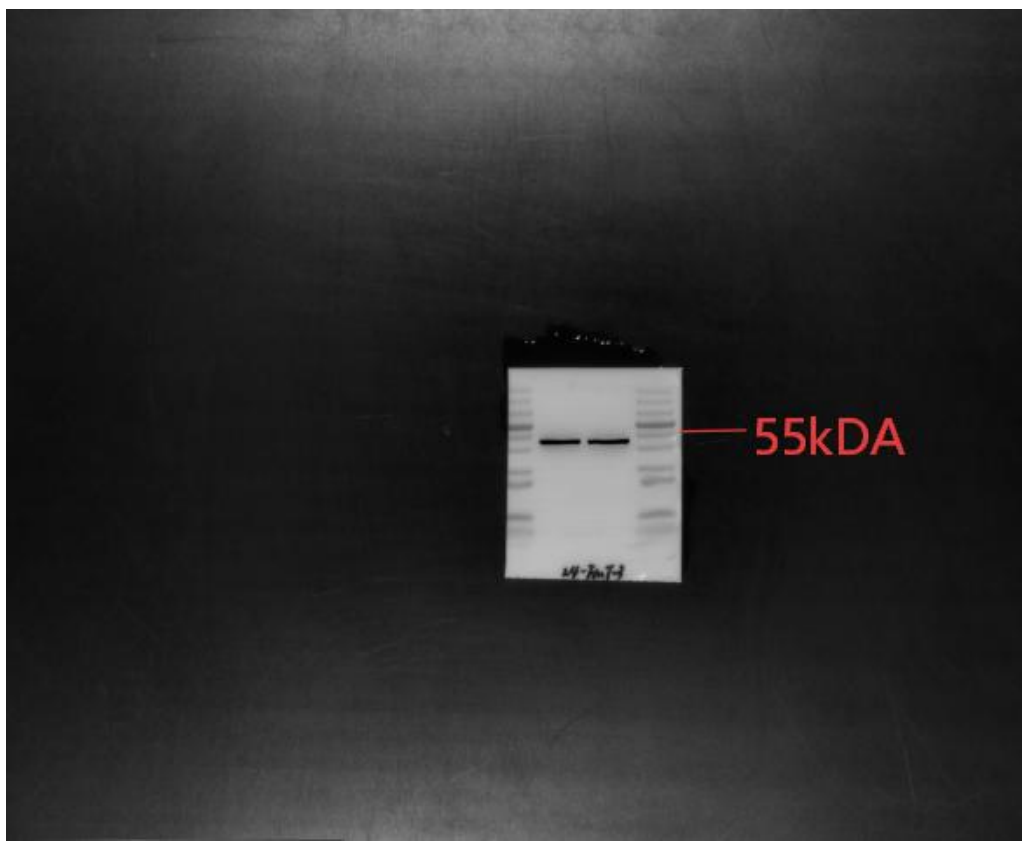

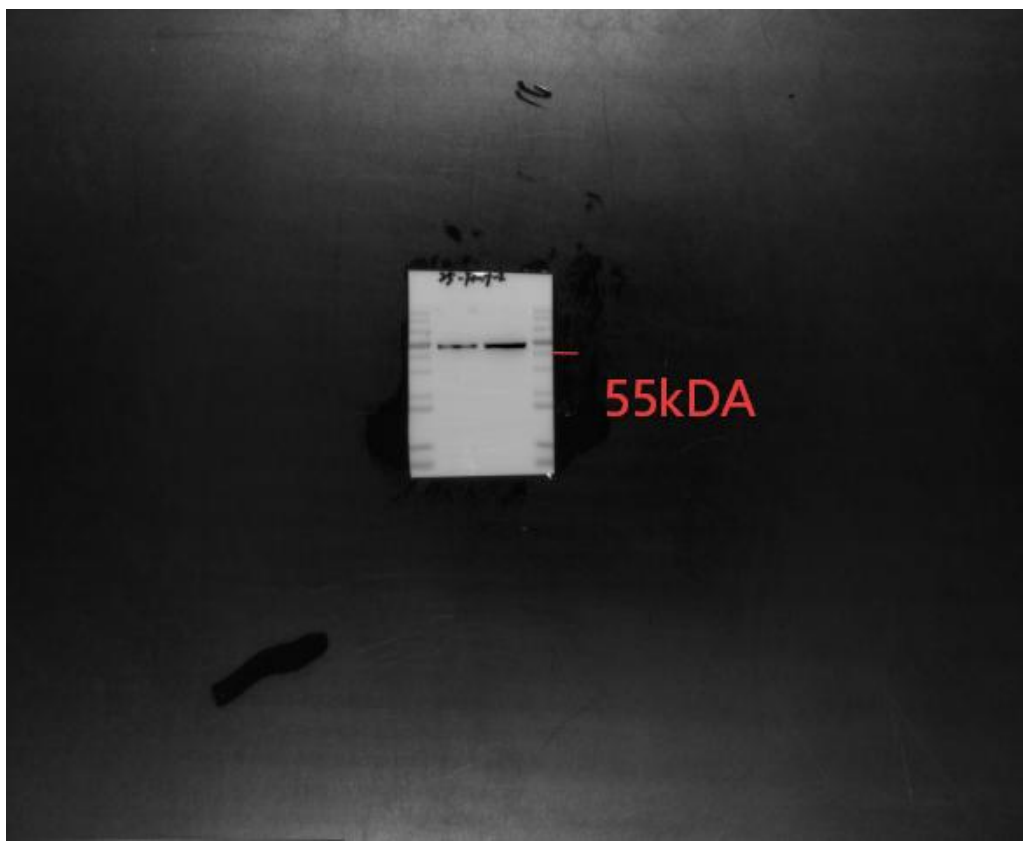

IRF3

p-IRF3

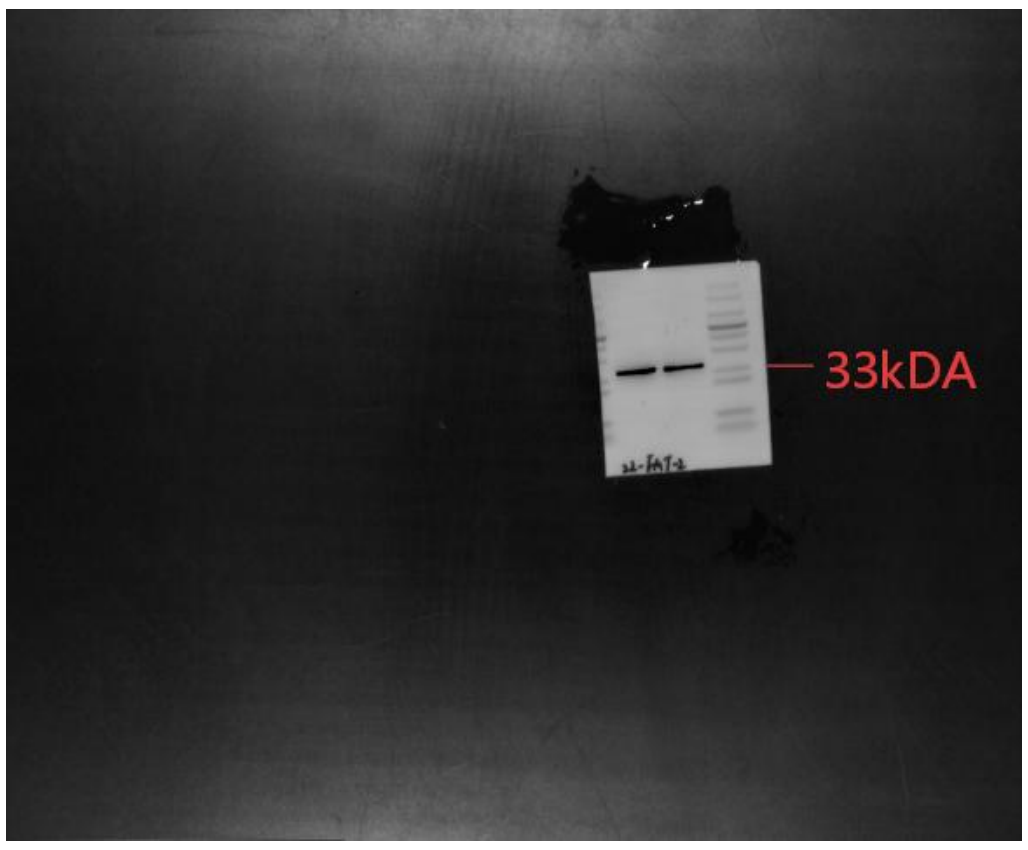

GAPDH

Figure 2G

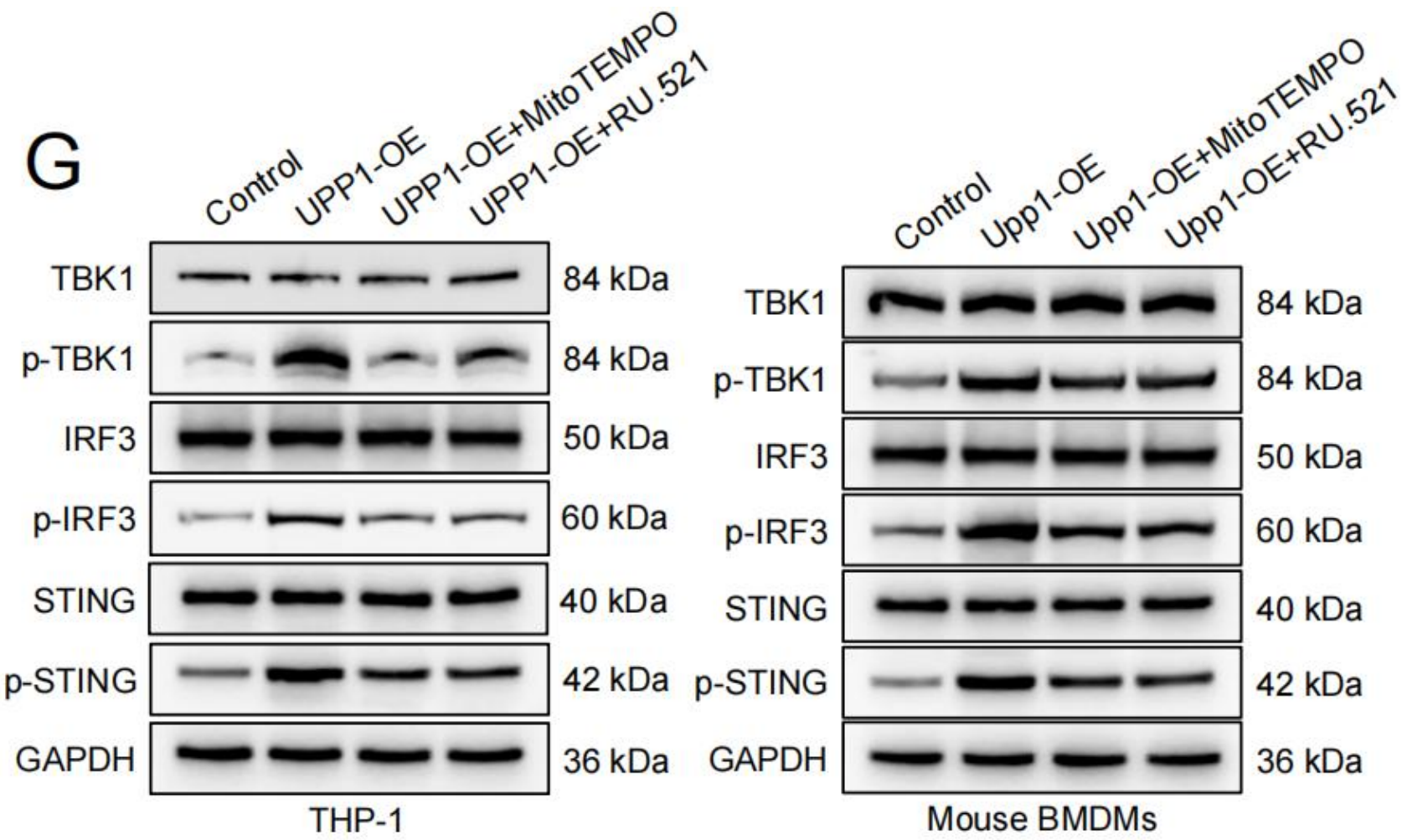

THP-1

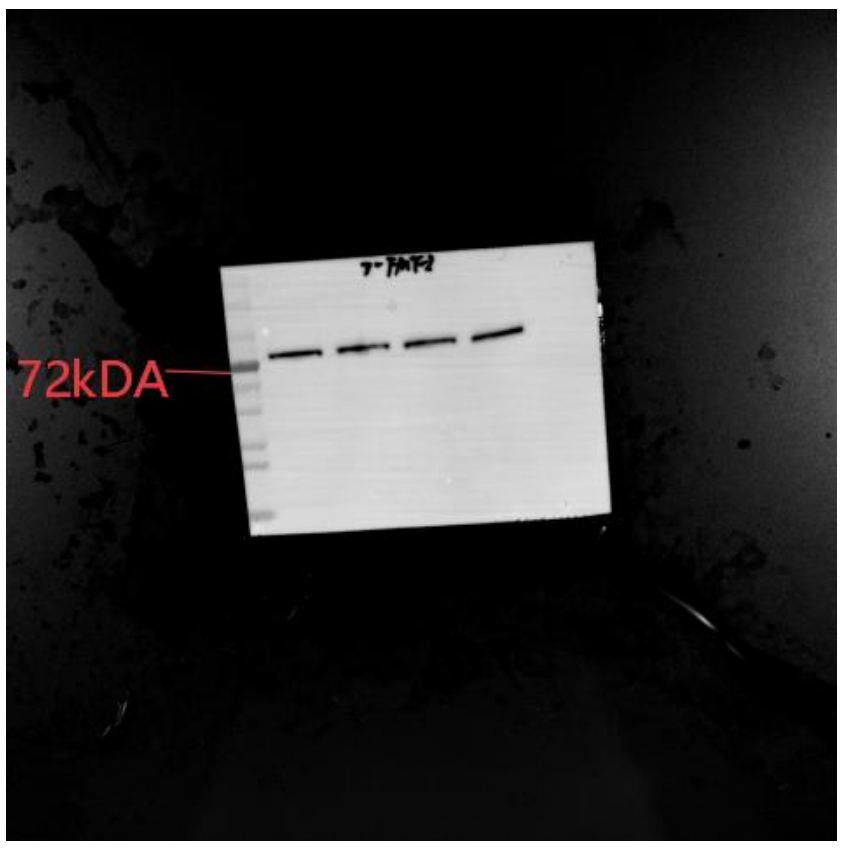

TBK1

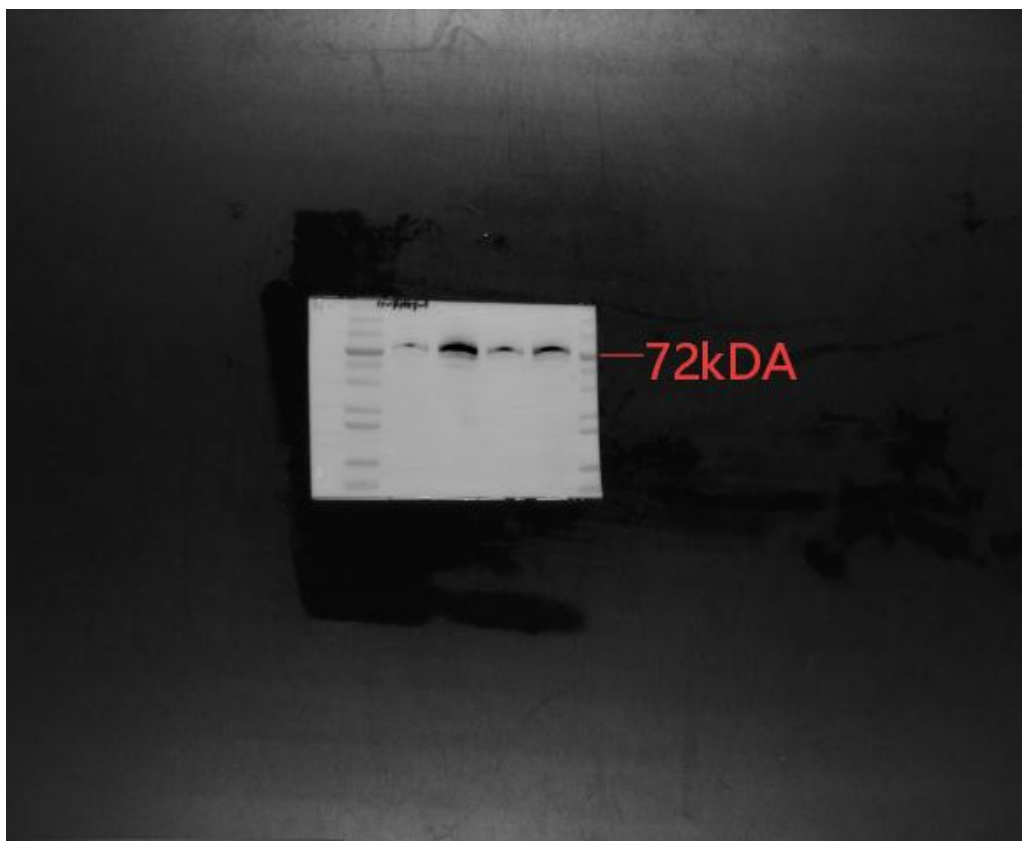

p-TBK1

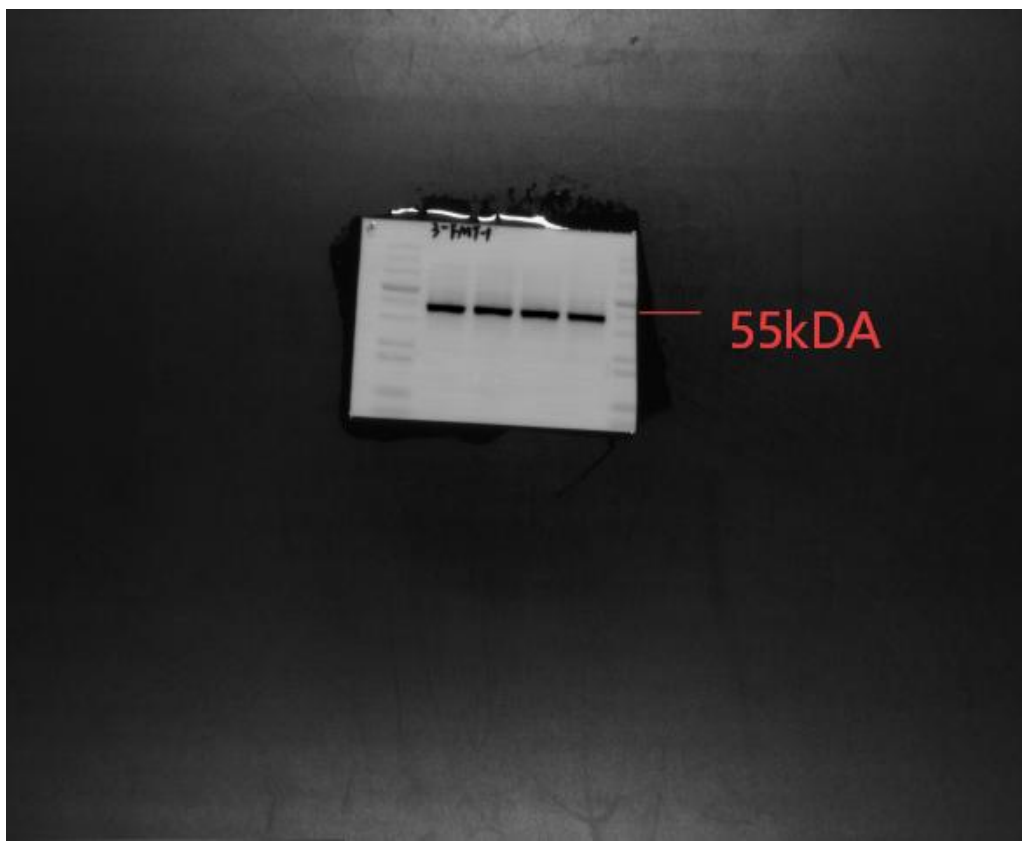

IRF3

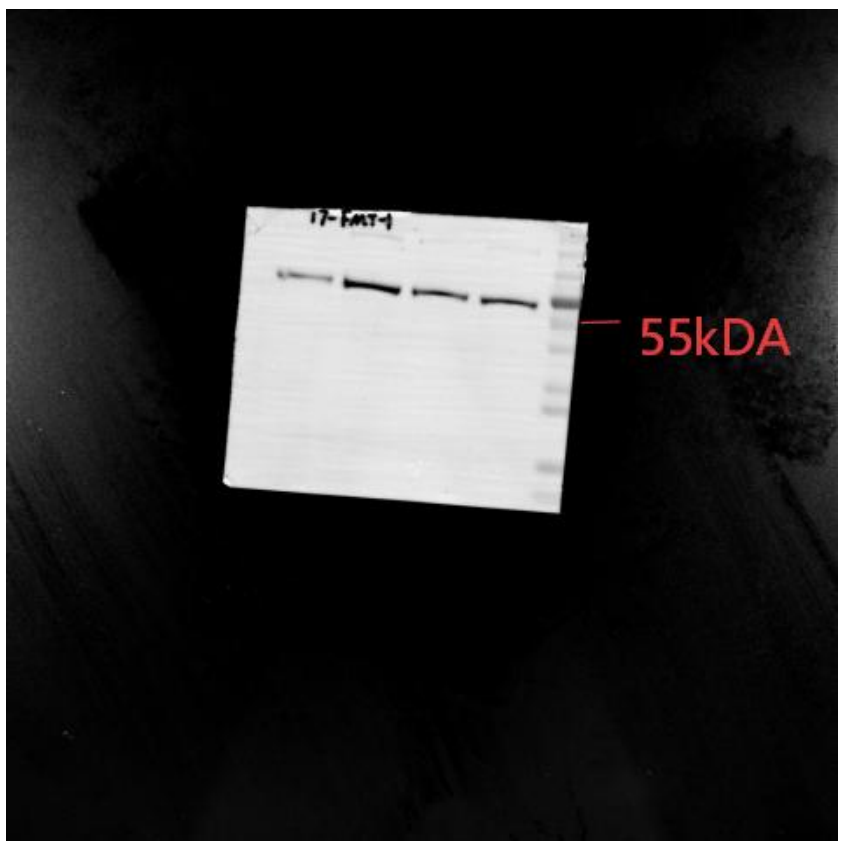

p-IRF3

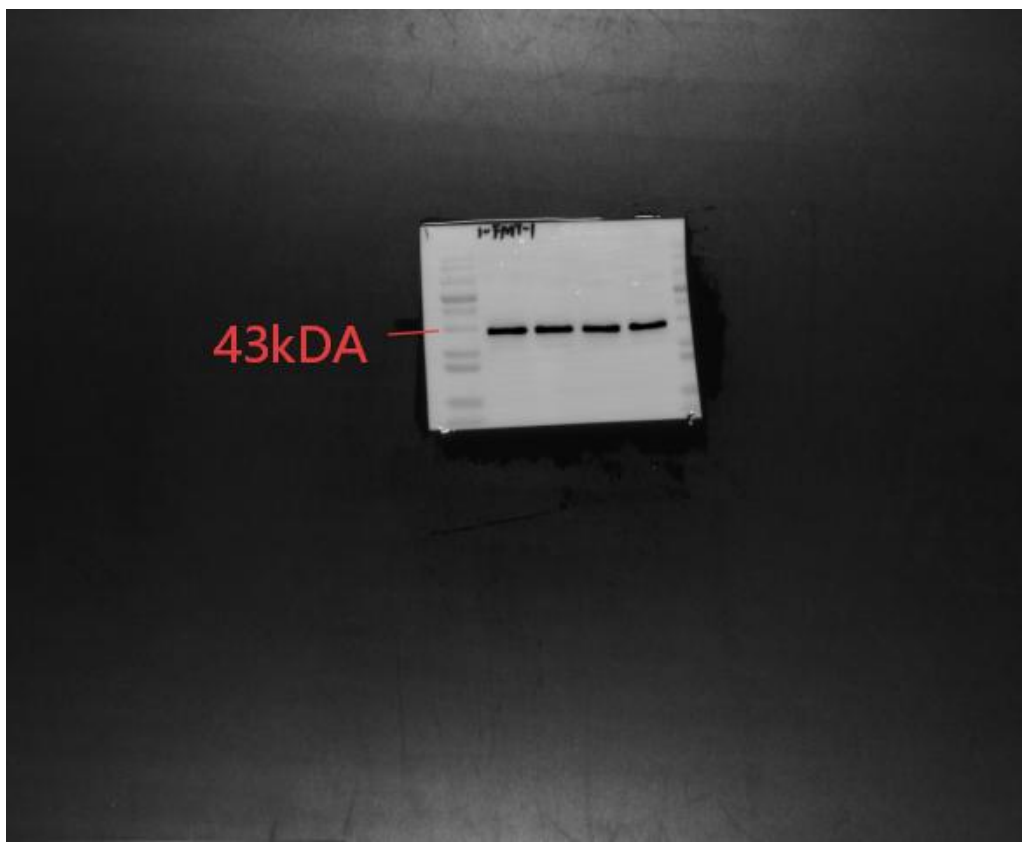

STING

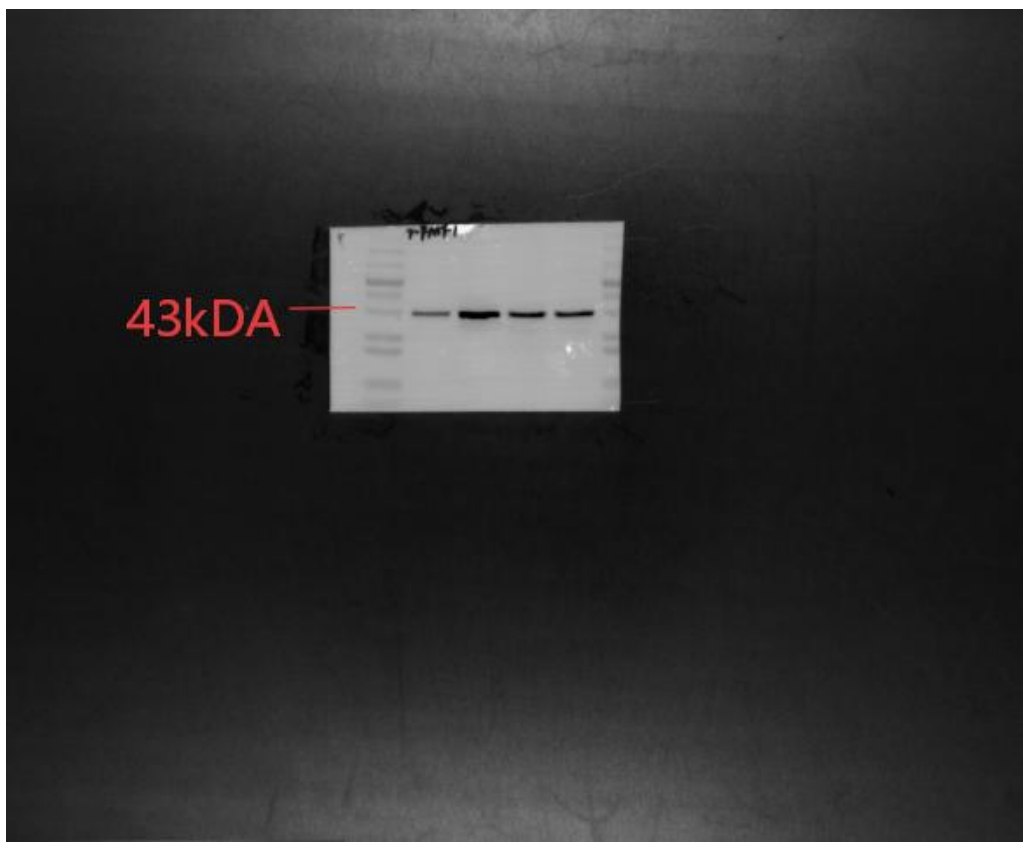

p-STING

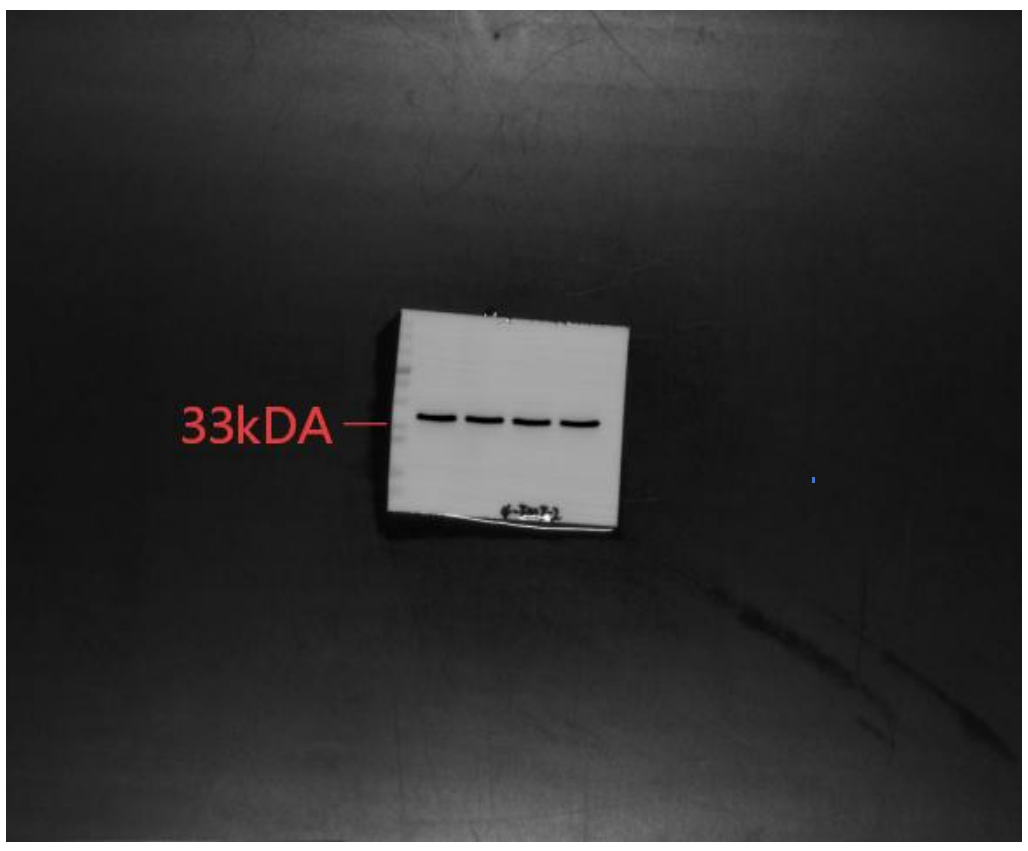

GAPDH

Mouse BMDMs

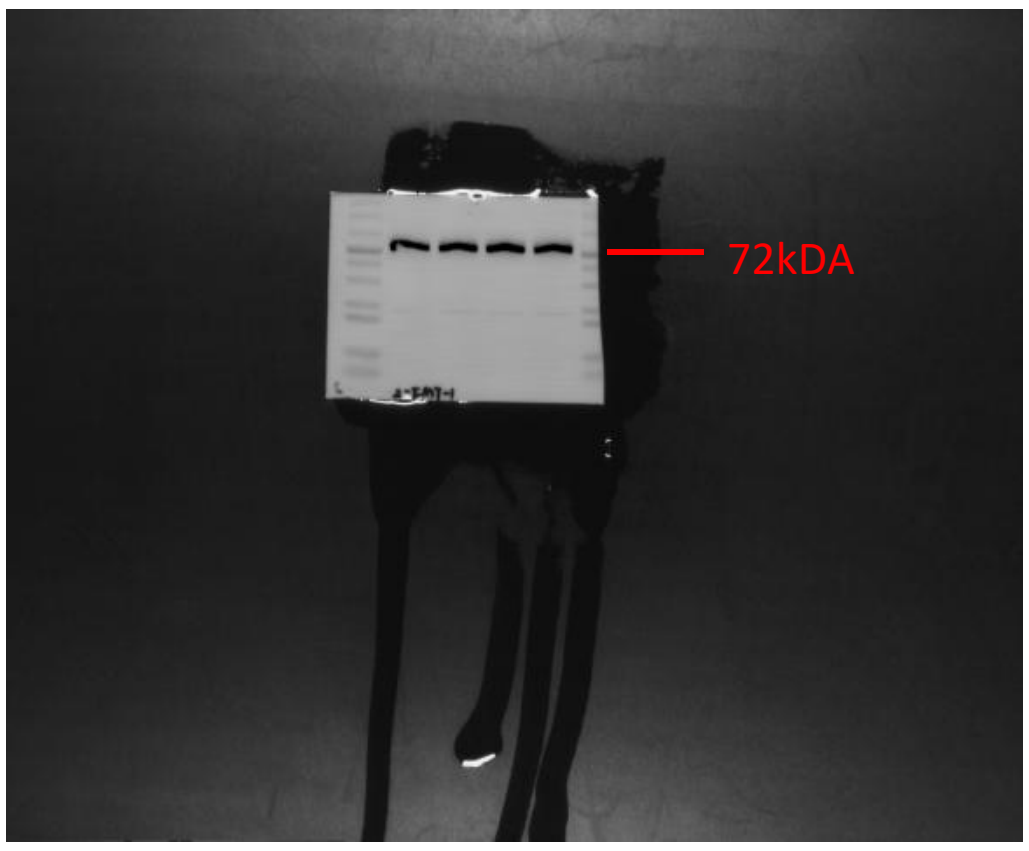

TBK1

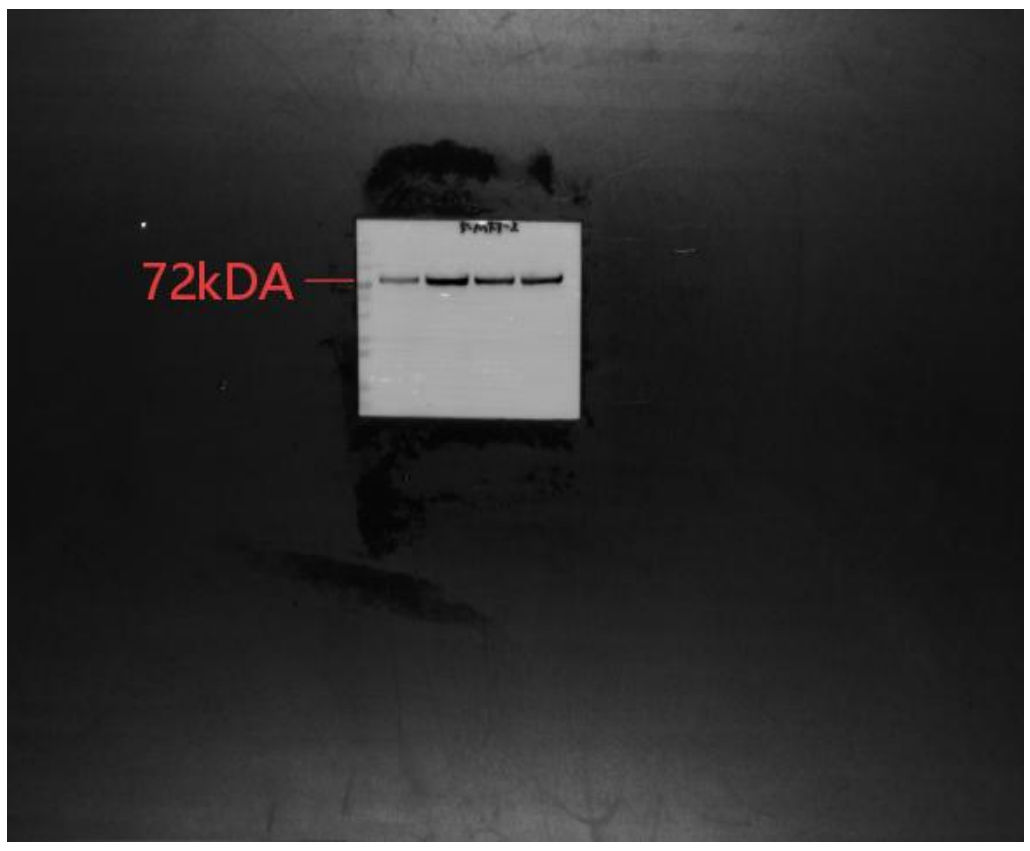

p-TBK1

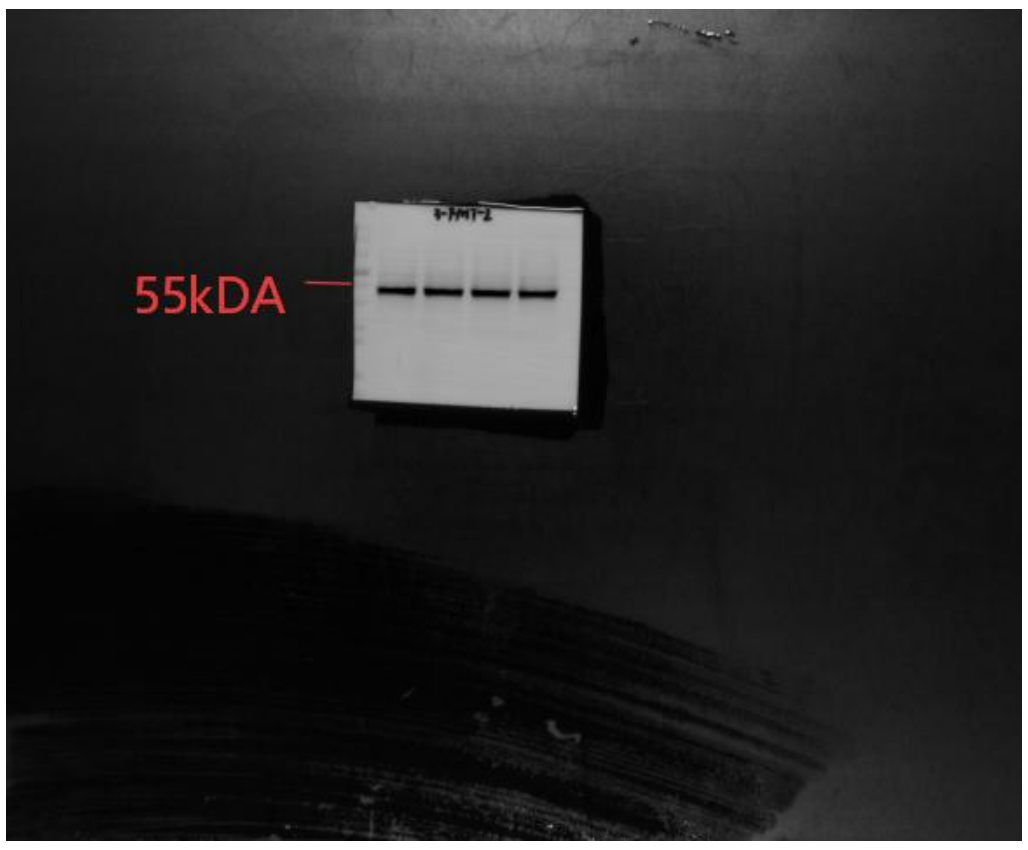

IRF3

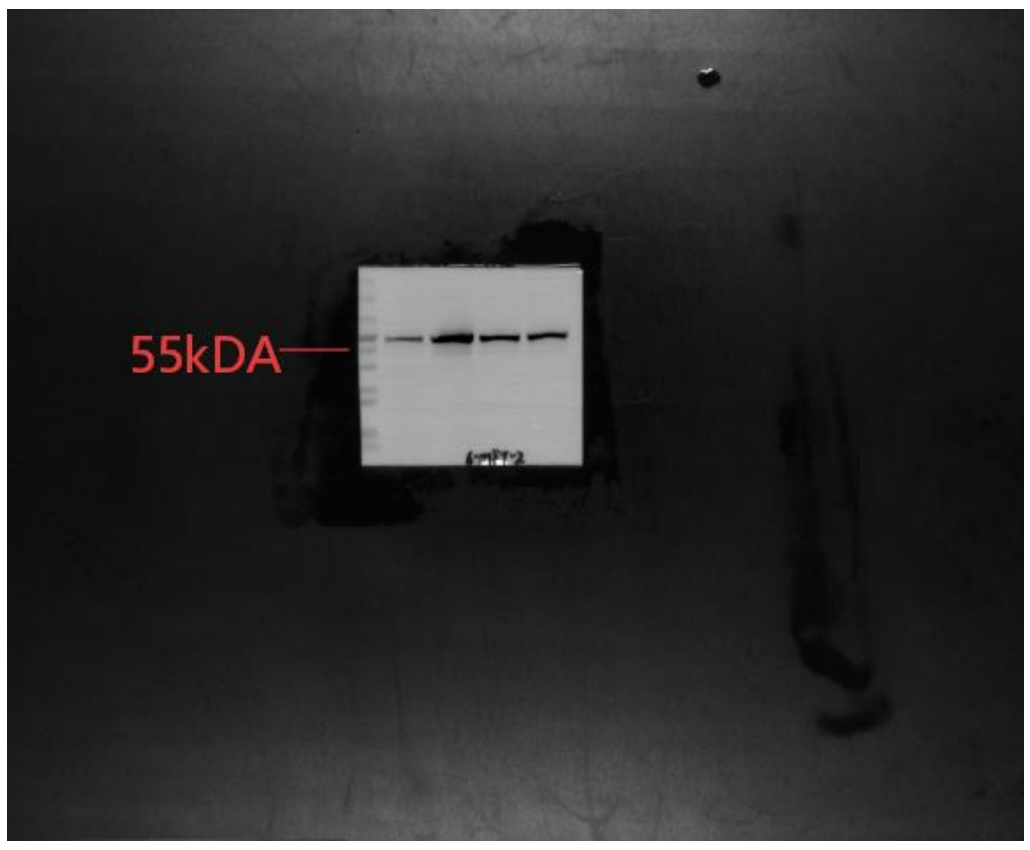

p-IRF3

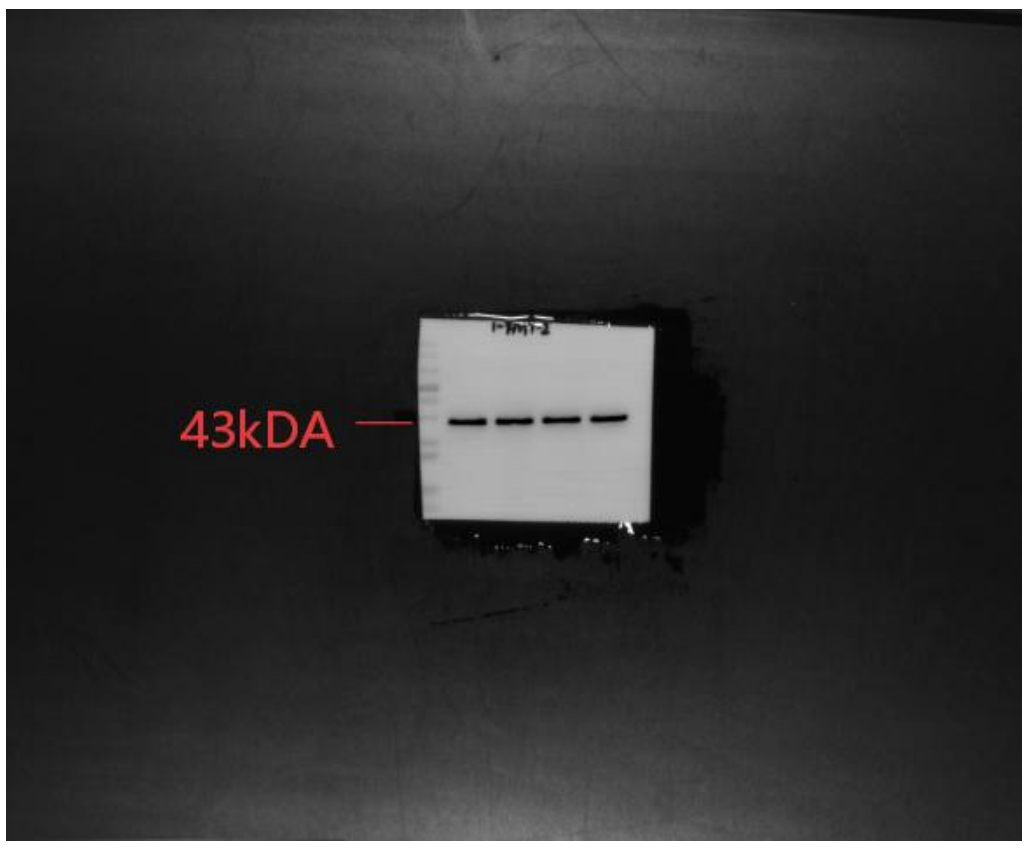

STING

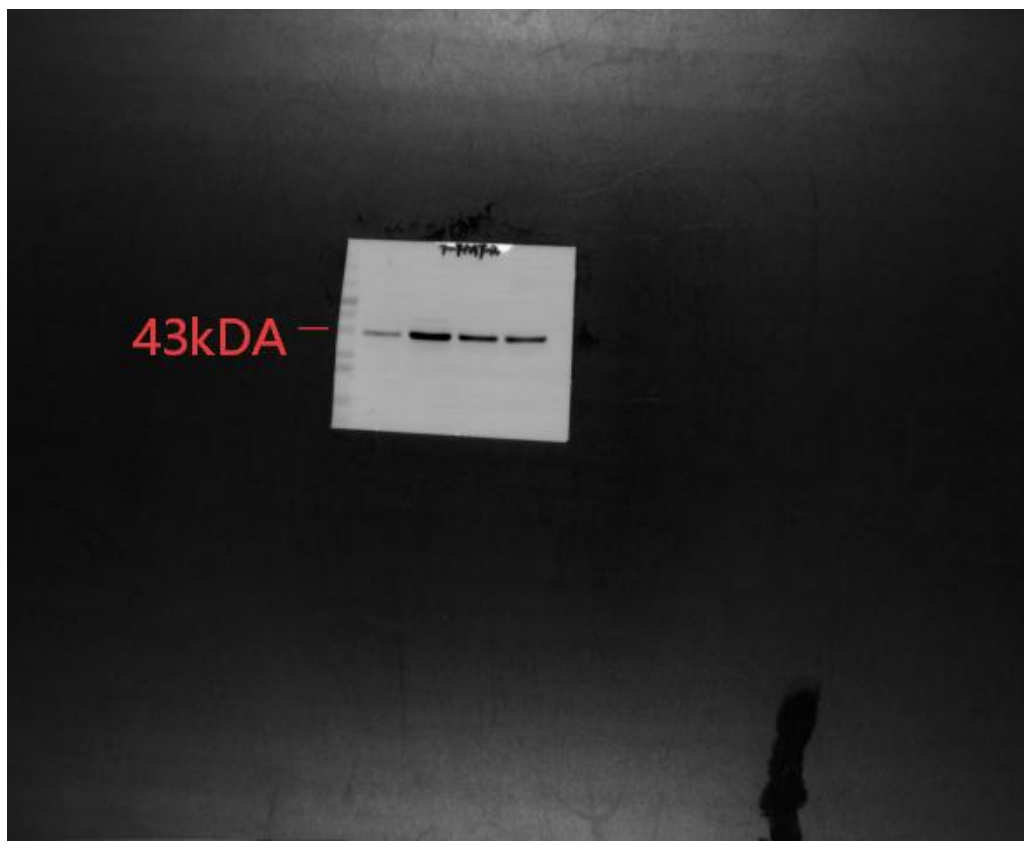

p-STING

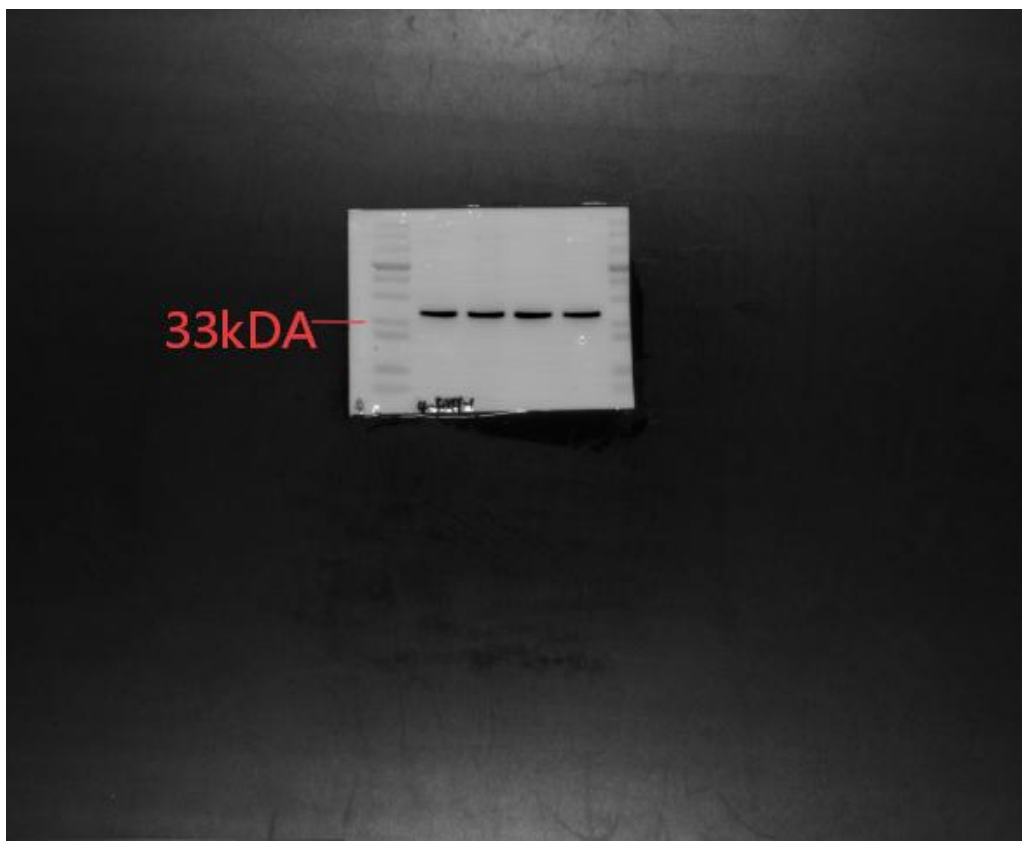

GAPDH

Figure 2H

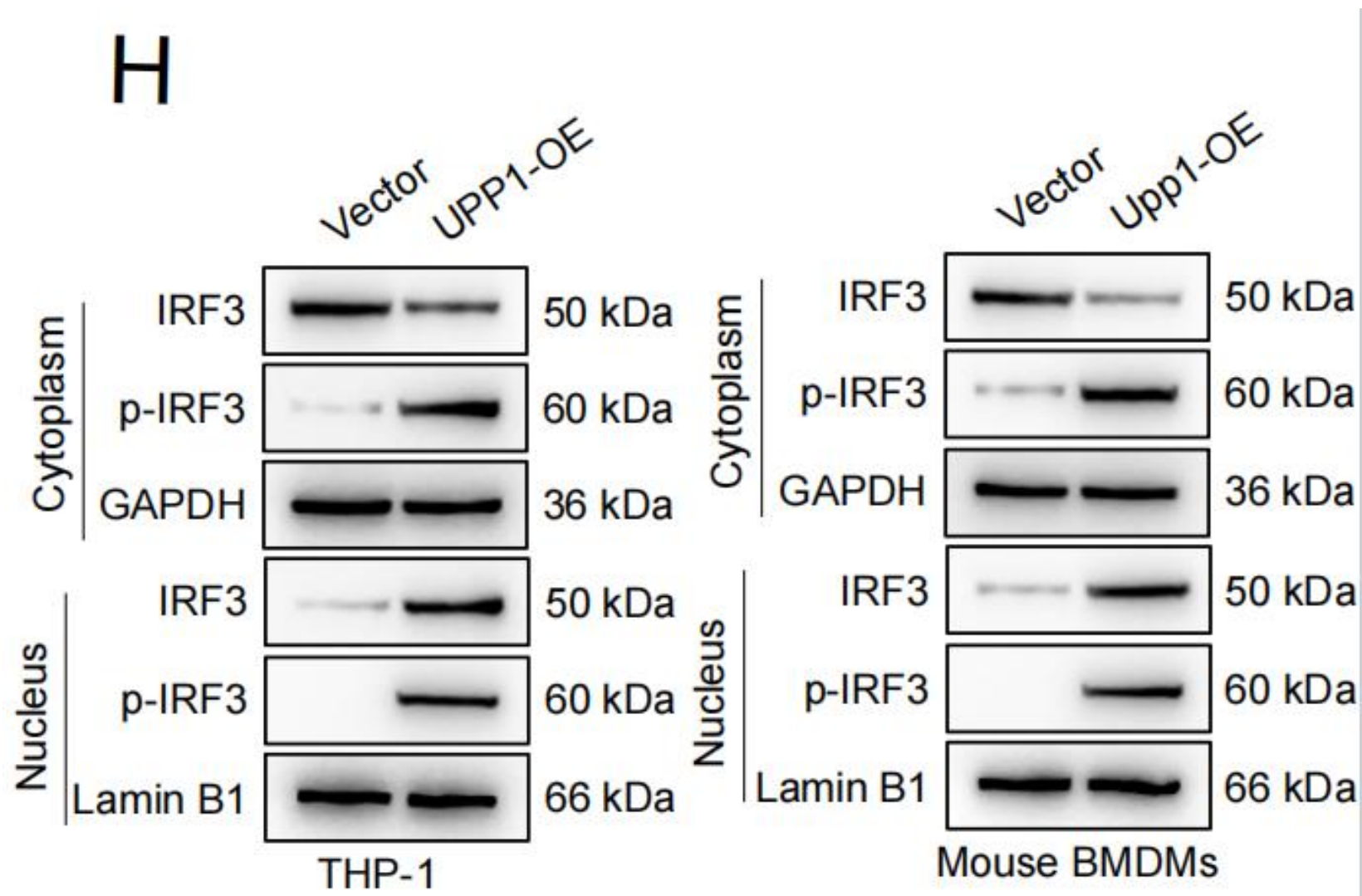

THP-1 Cytoplasm

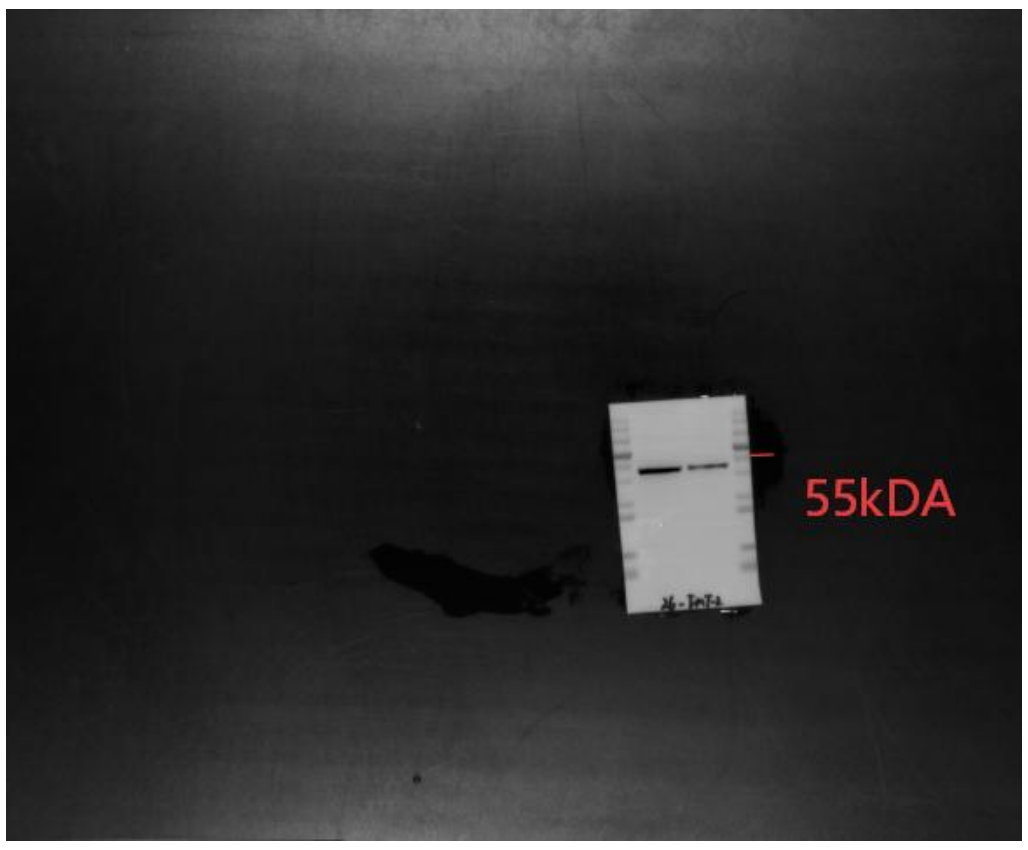

IRF3

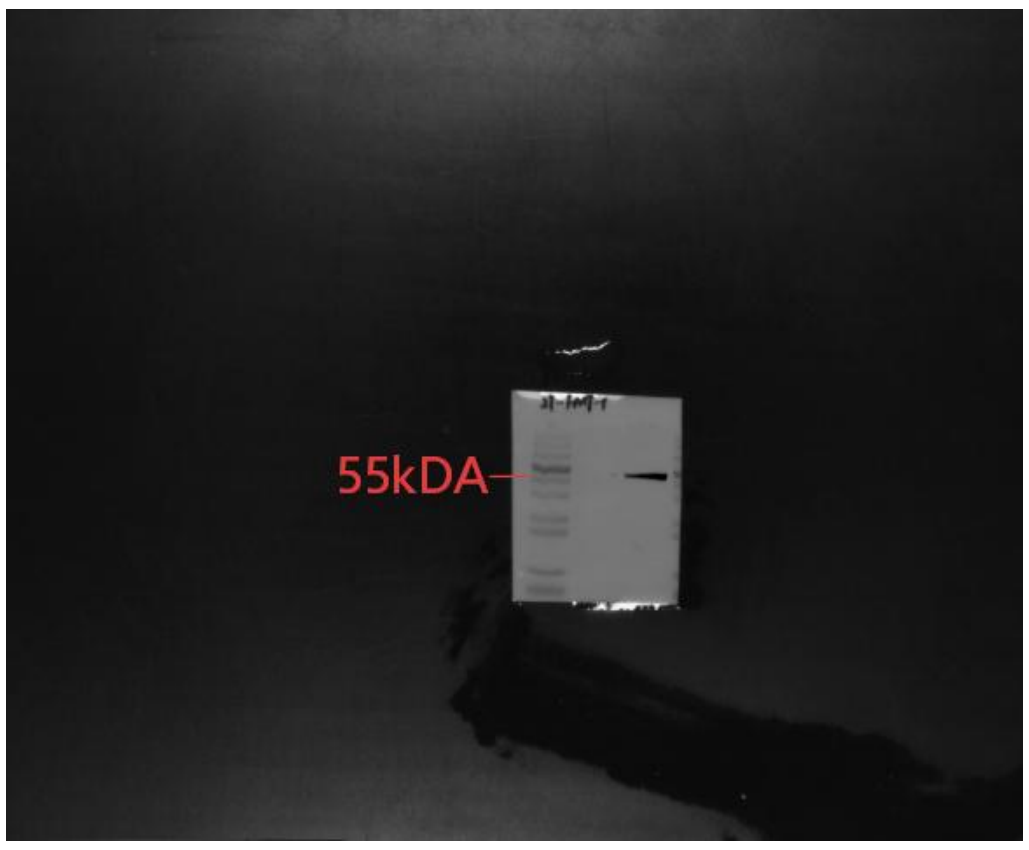

p-IRF3

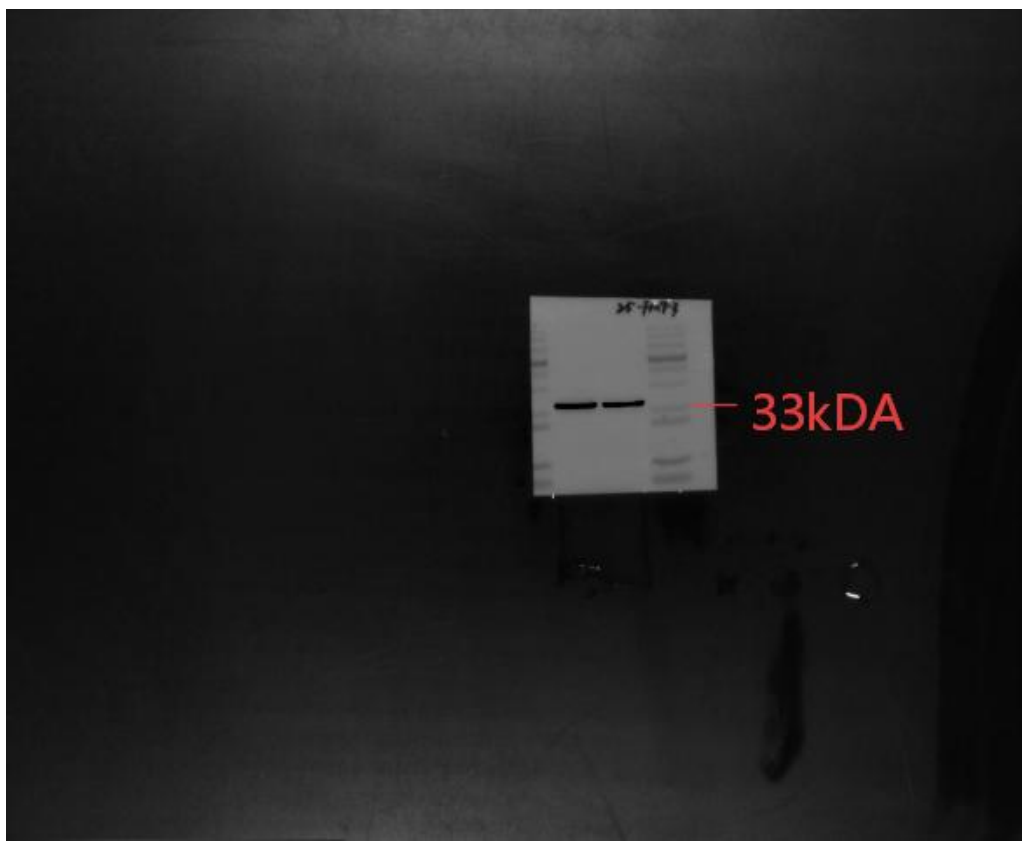

GAPDH

THP-1 Nucleus

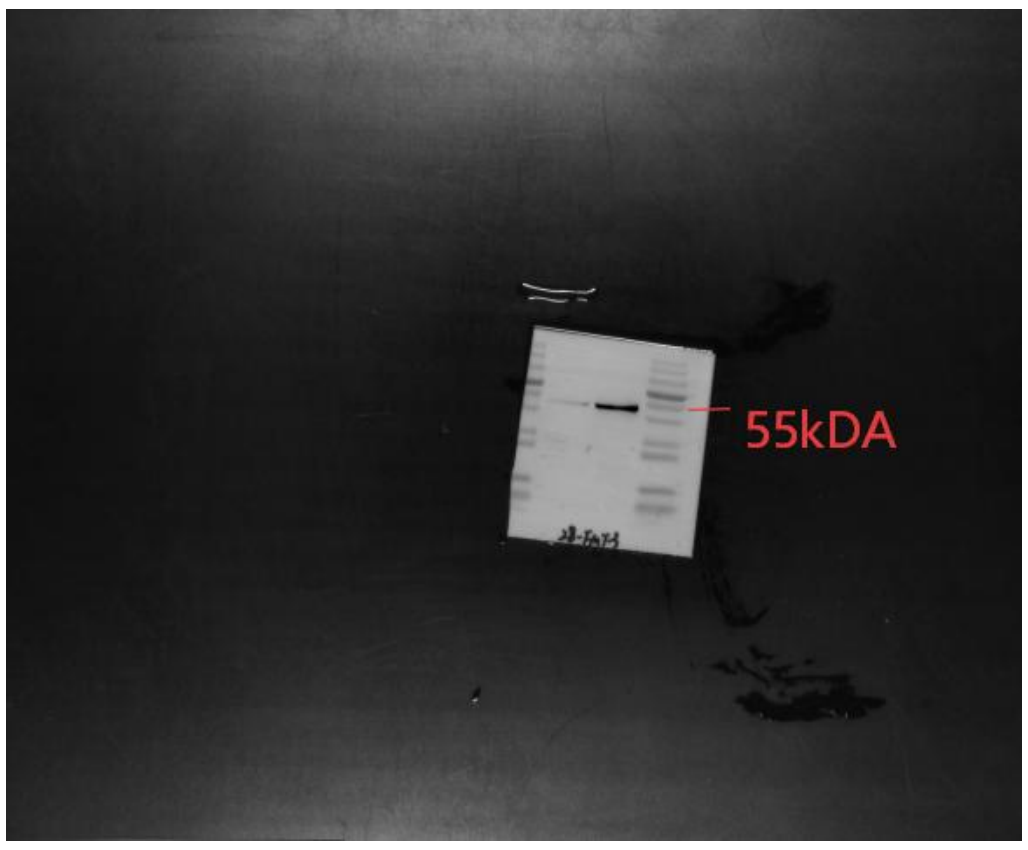

IRF3

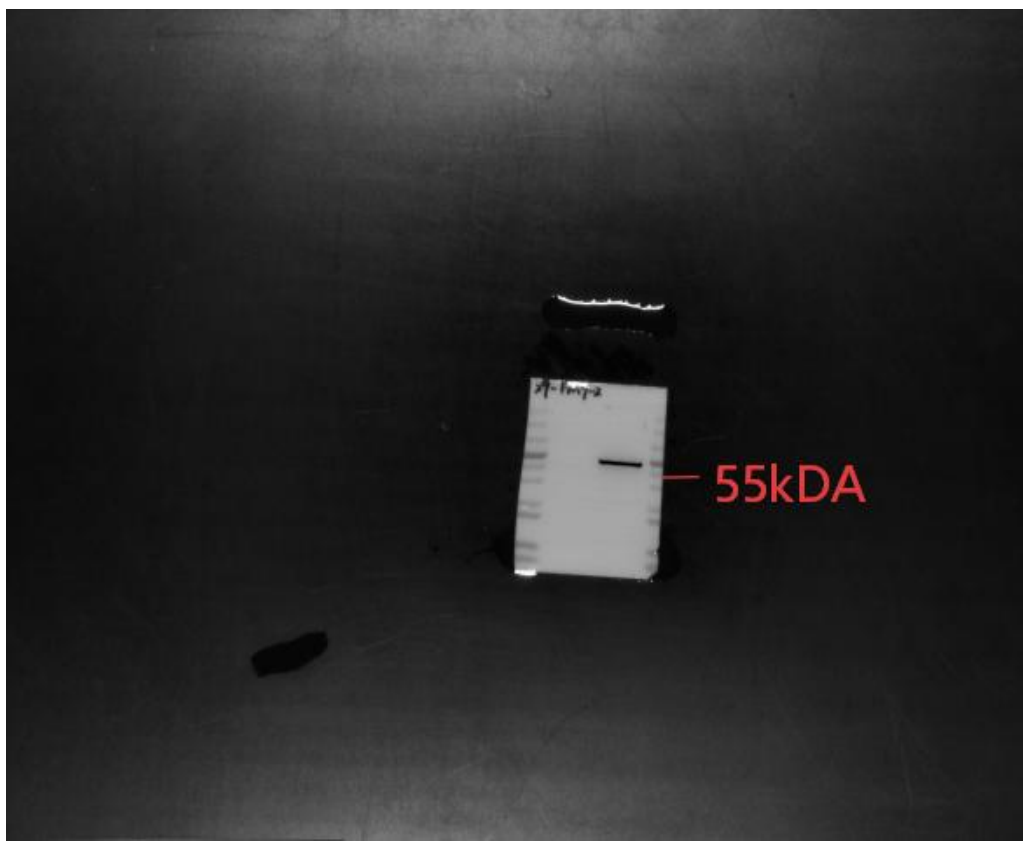

p-IRF3

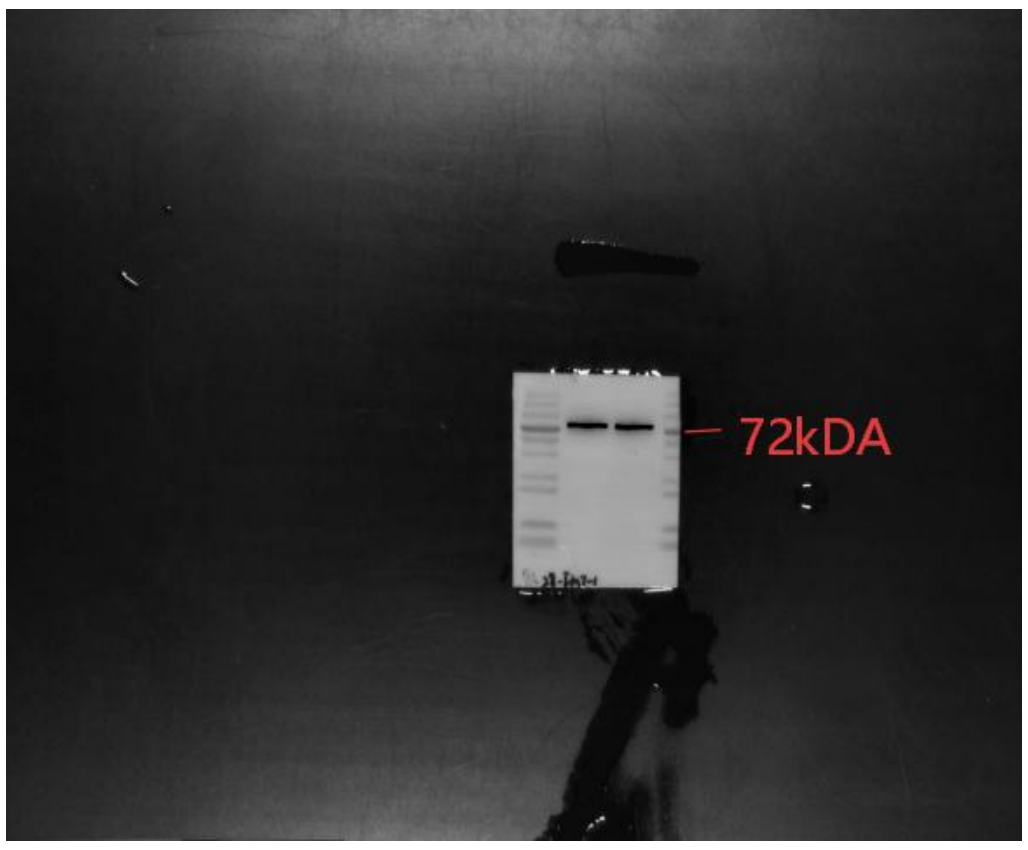

Lamin B1

Mouse BMDMs Cytoplasm

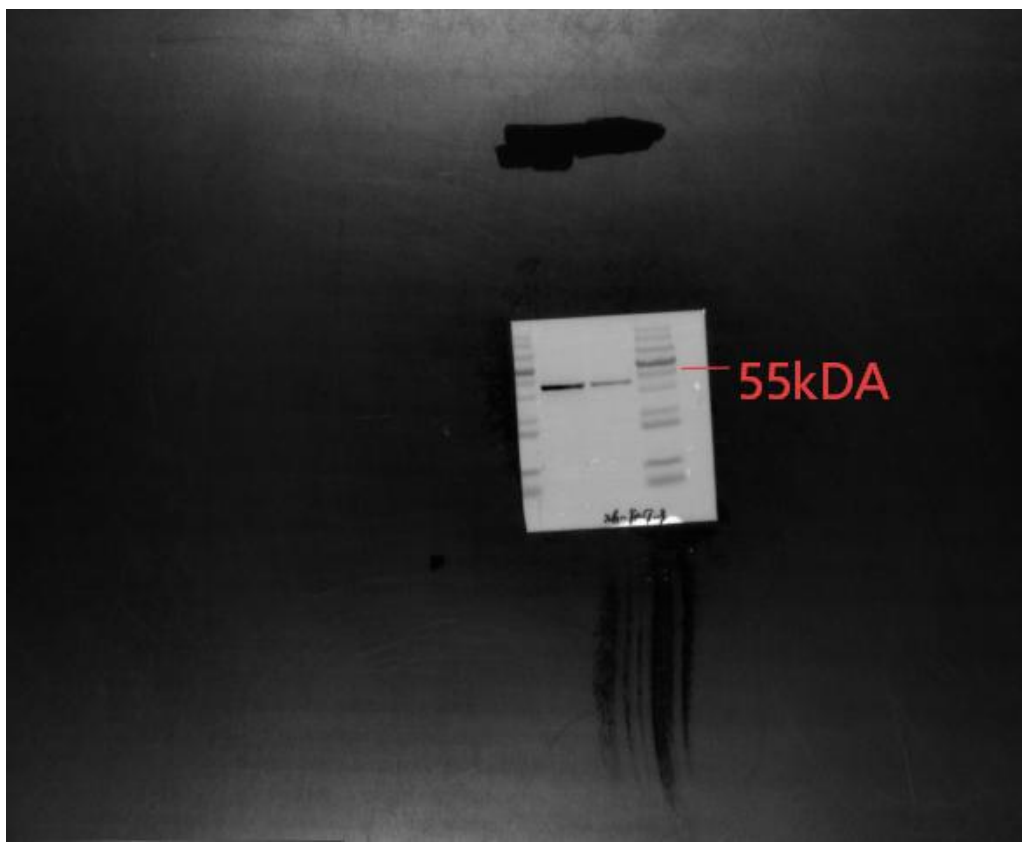

IRF3

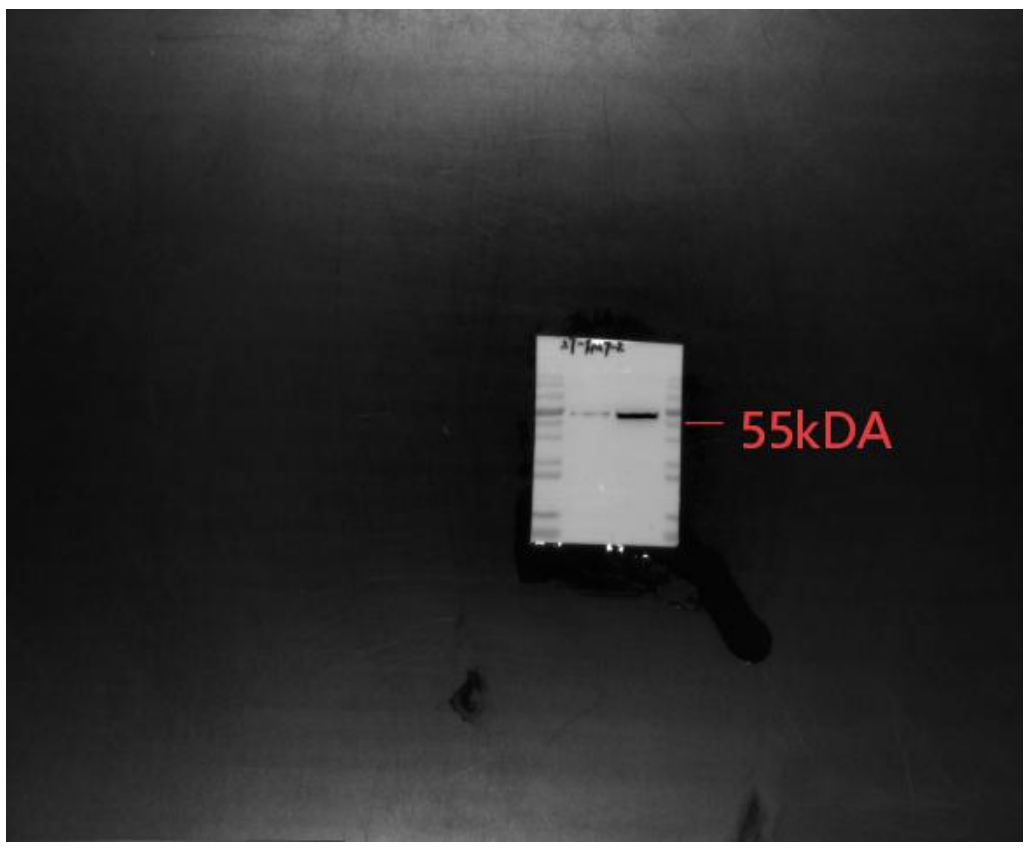

p-IRF3

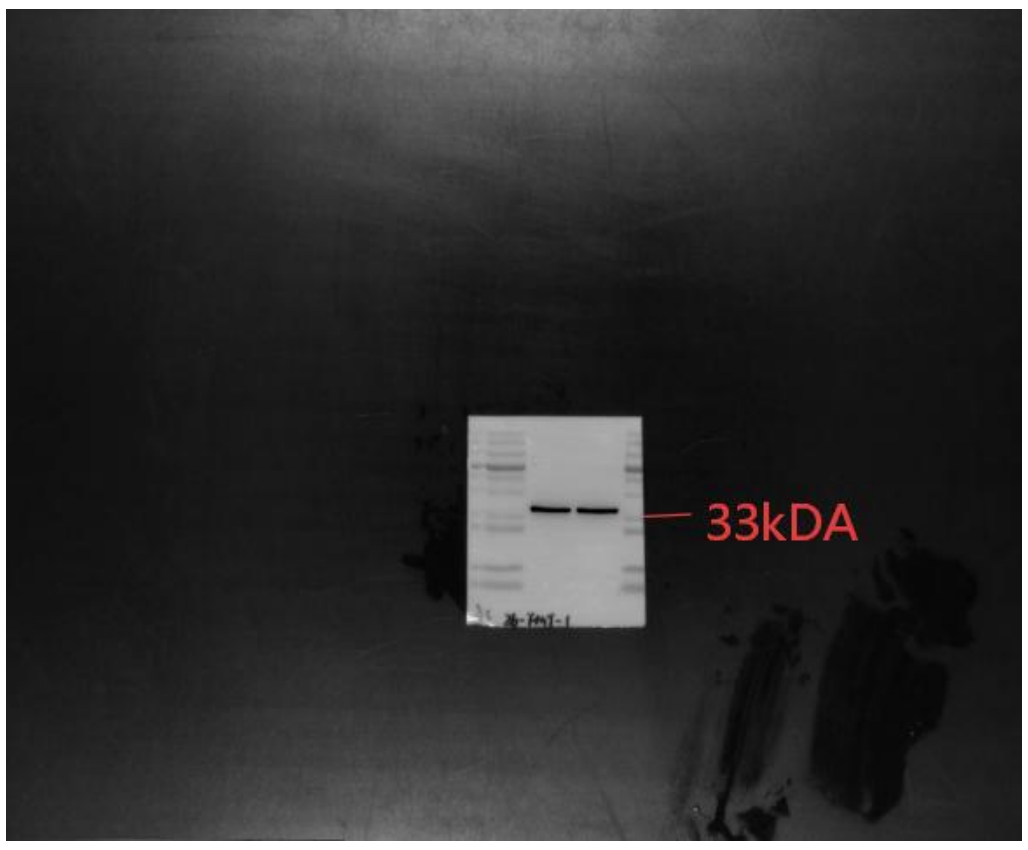

GAPDH

Mouse BMDMs Nucleus

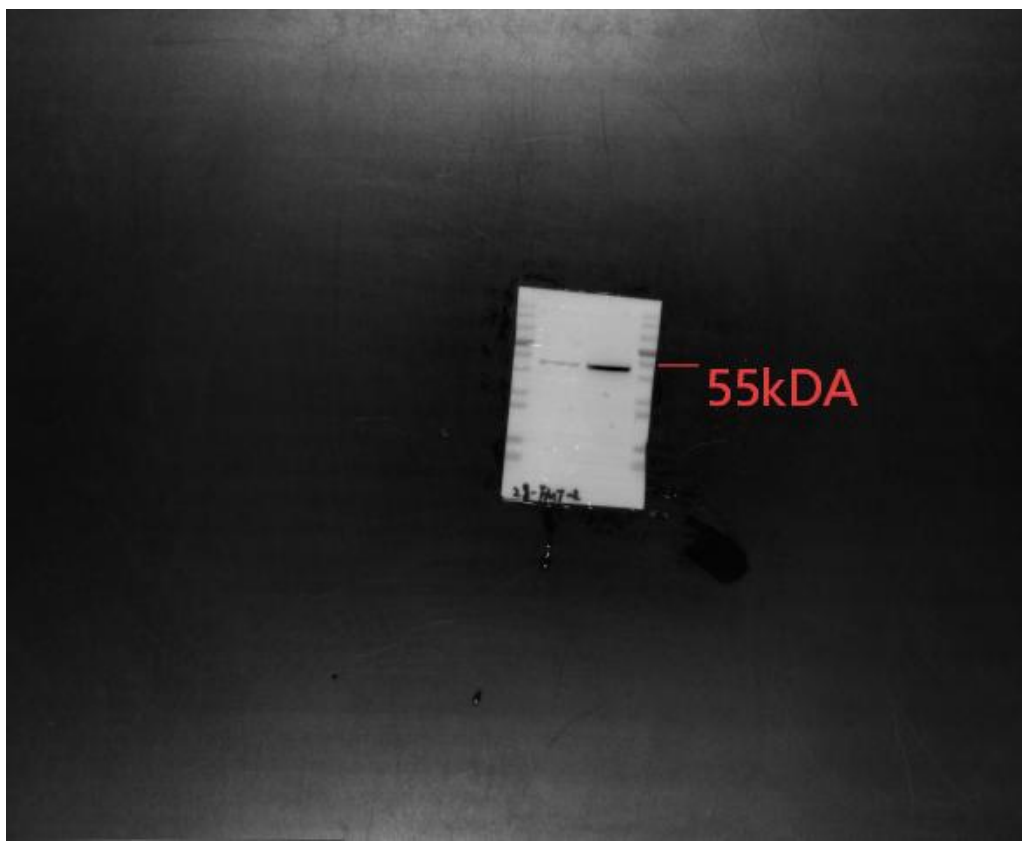

IRF3

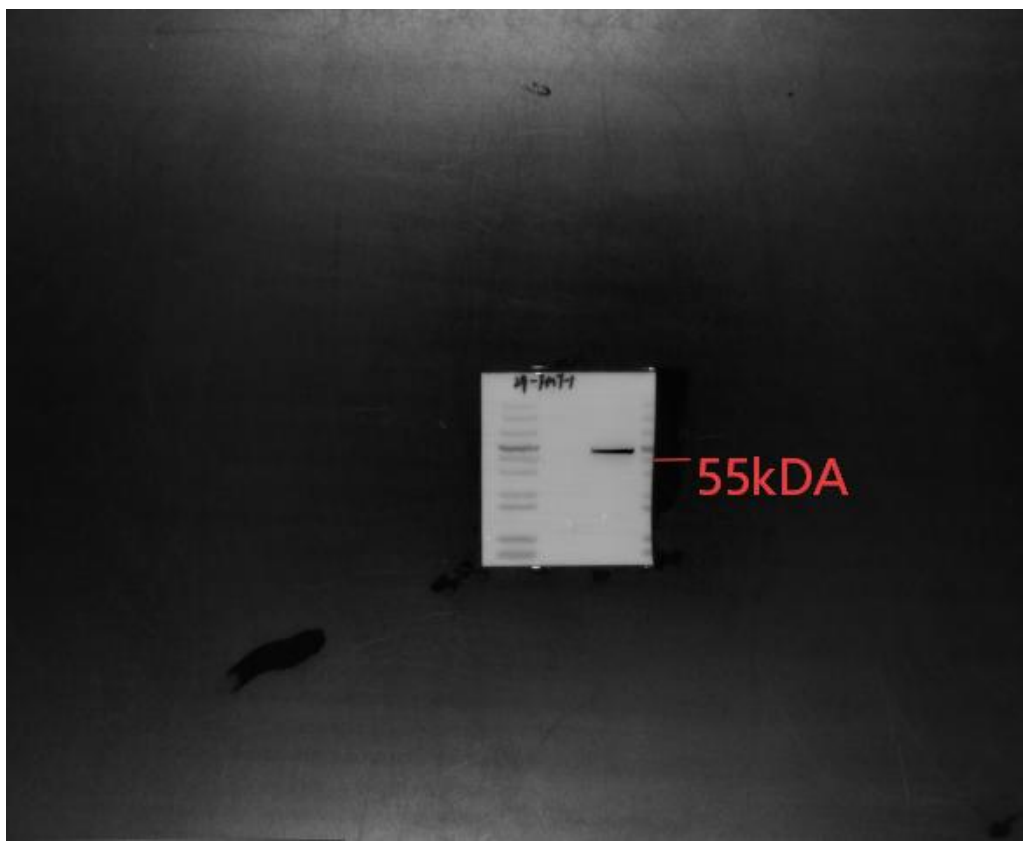

p-IRF3

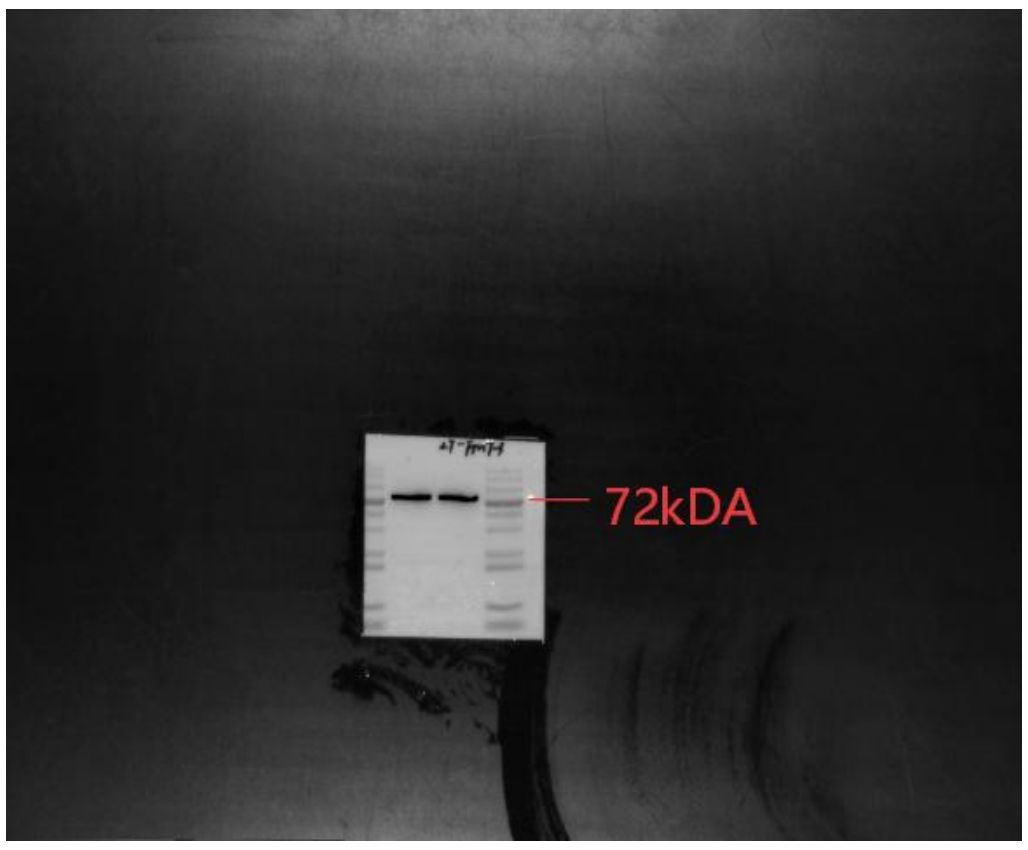

Lamin B1

Figure 3C

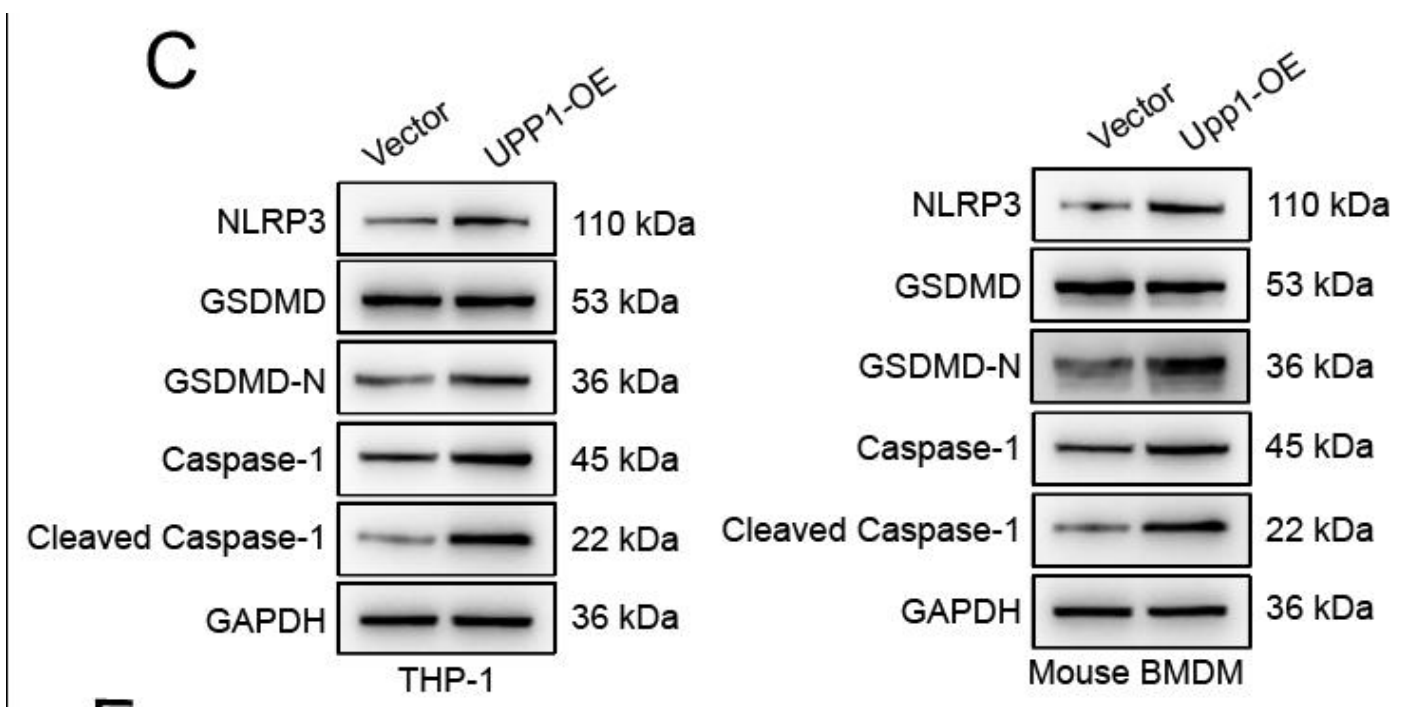

THP-1

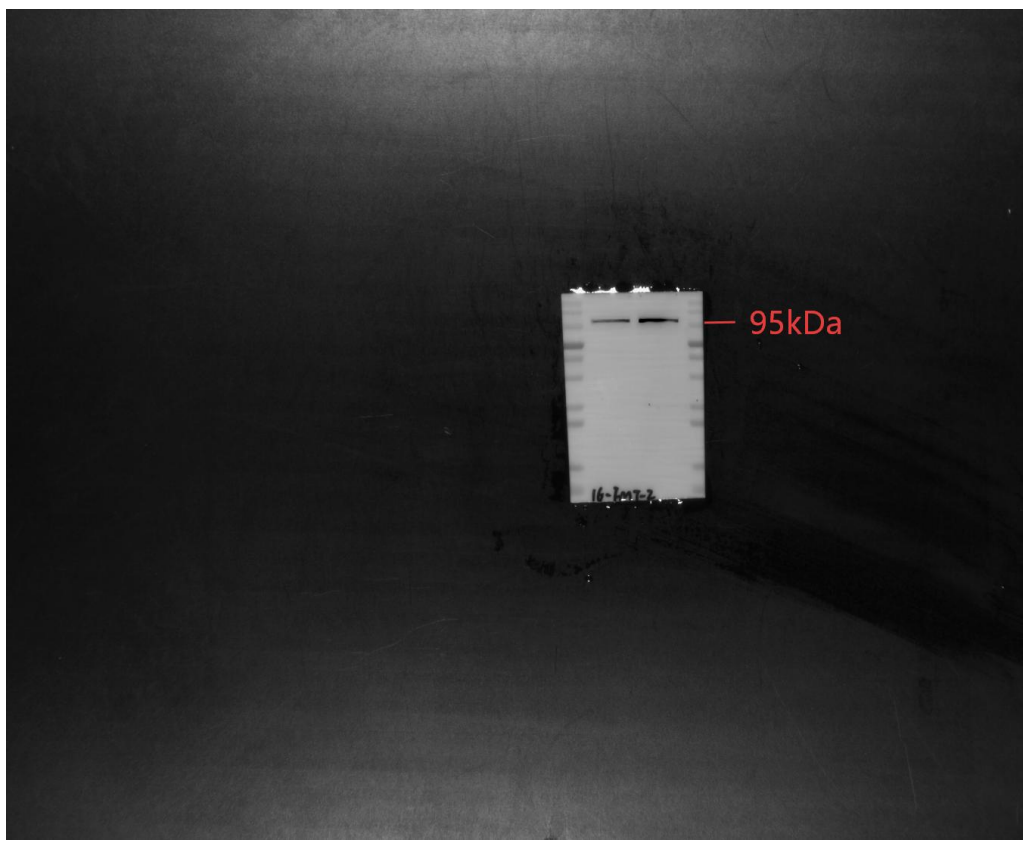

NLRP3

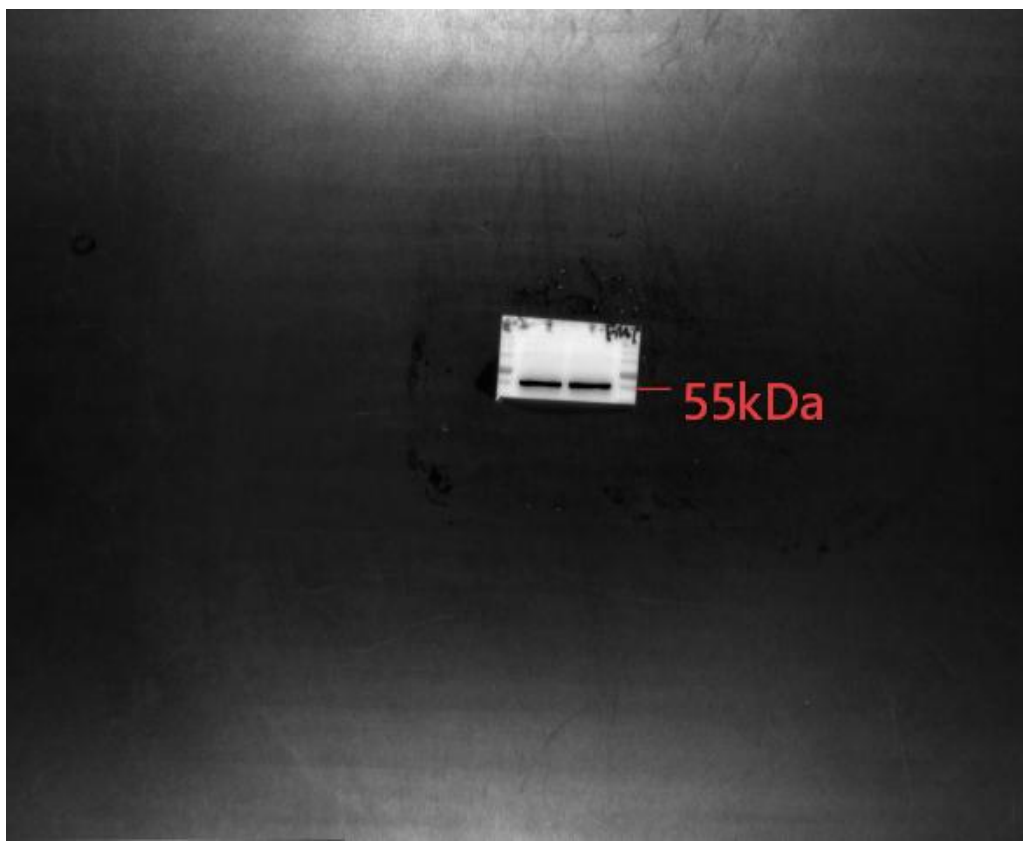

GSDMD

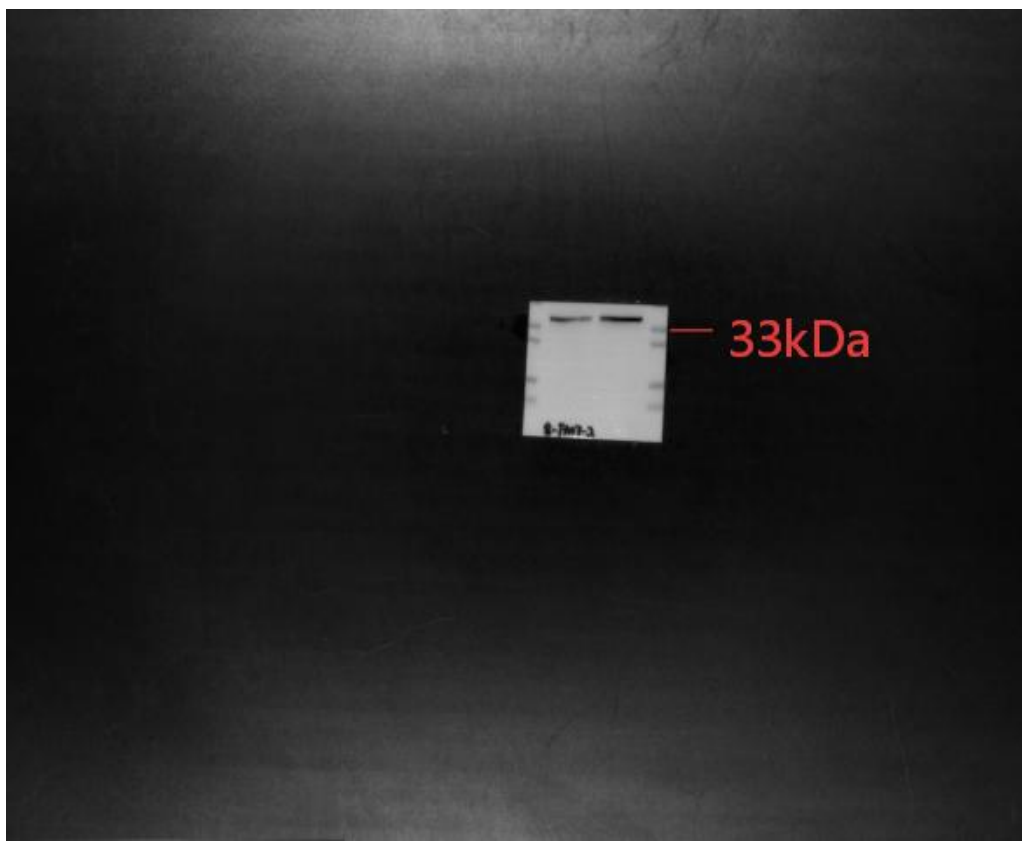

GSDMD-N

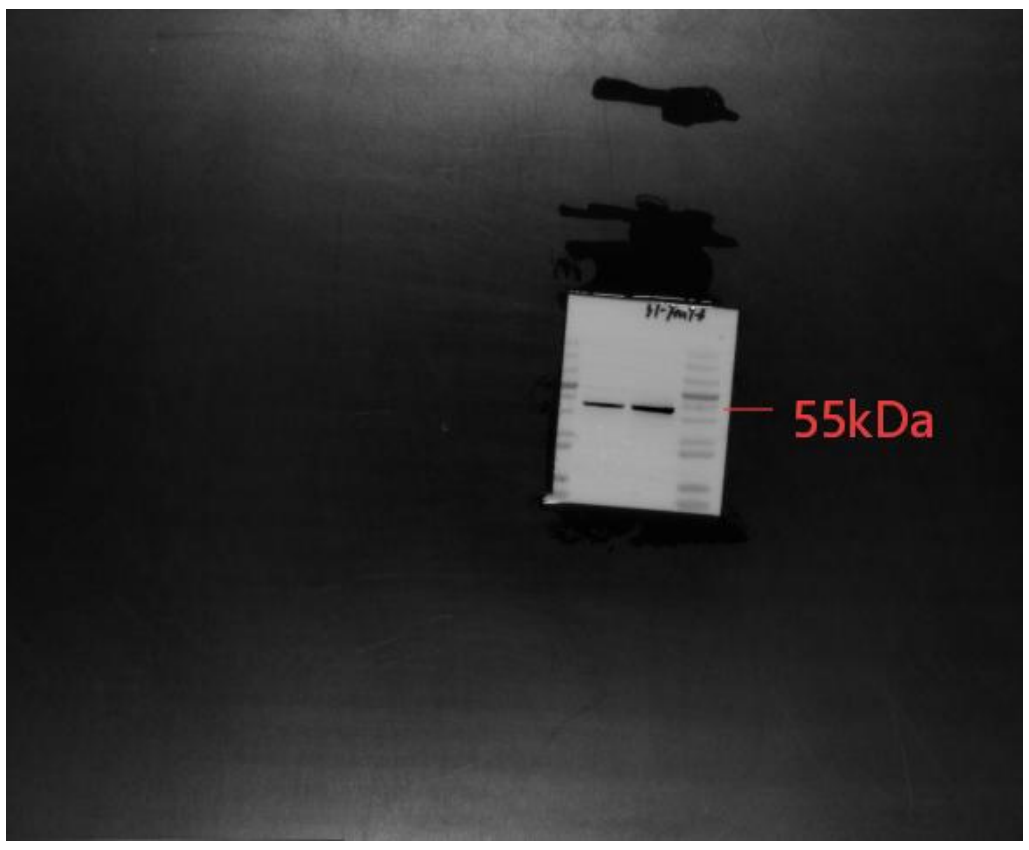

Caspase-1

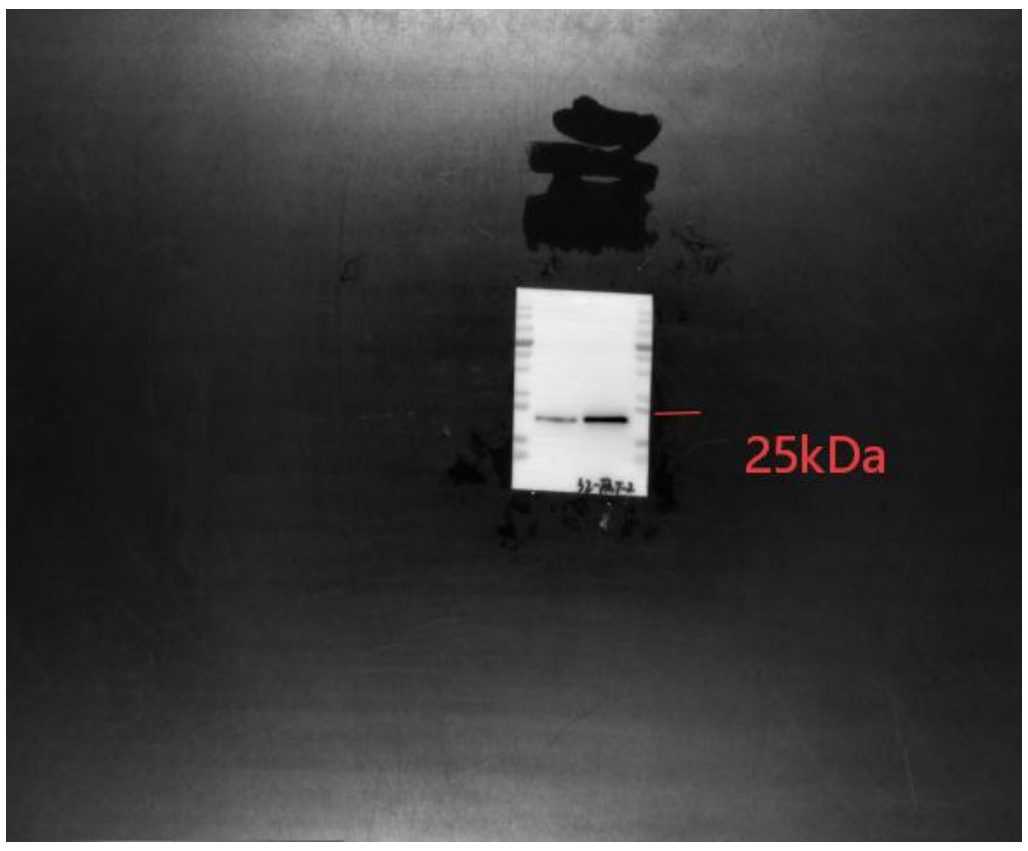

Cleaved Caspase-1

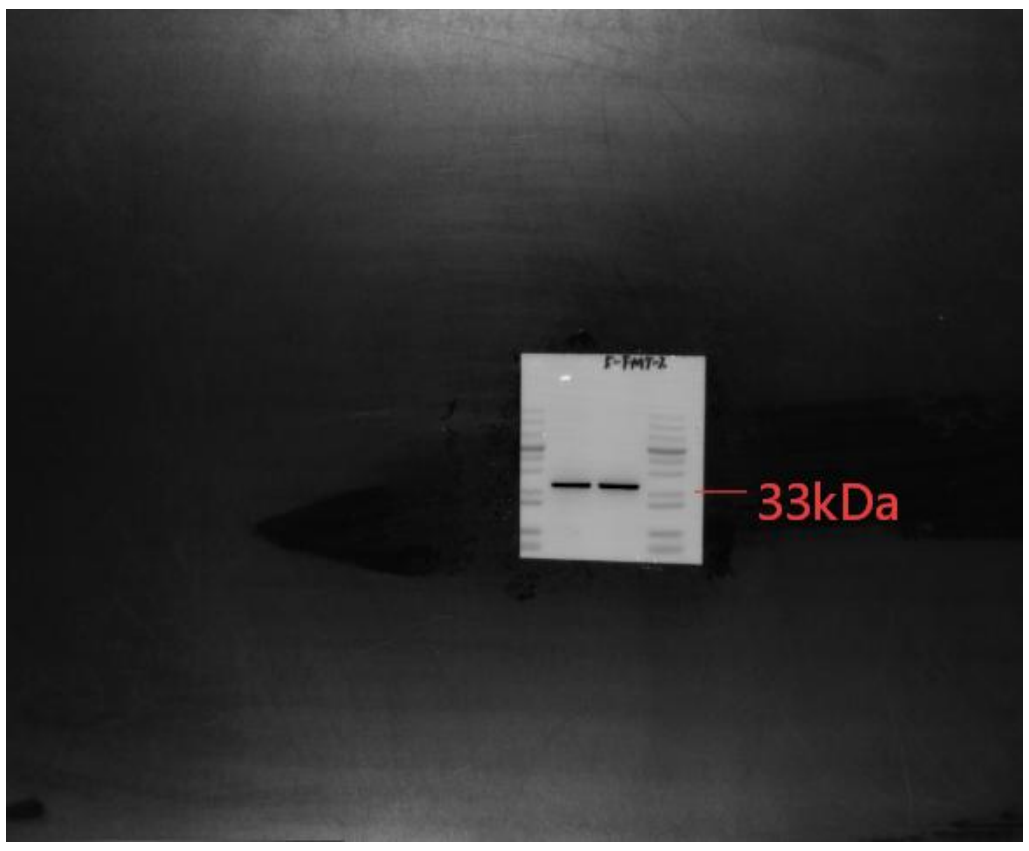

GAPDH

Mouse BMDMs

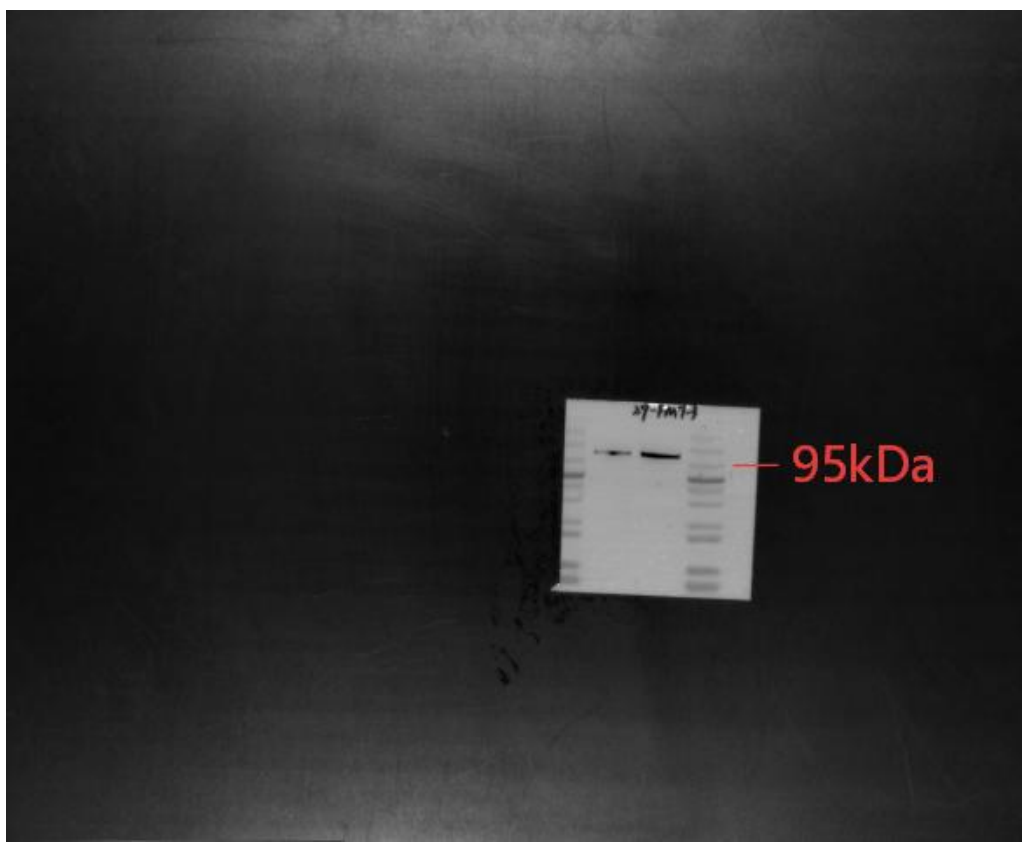

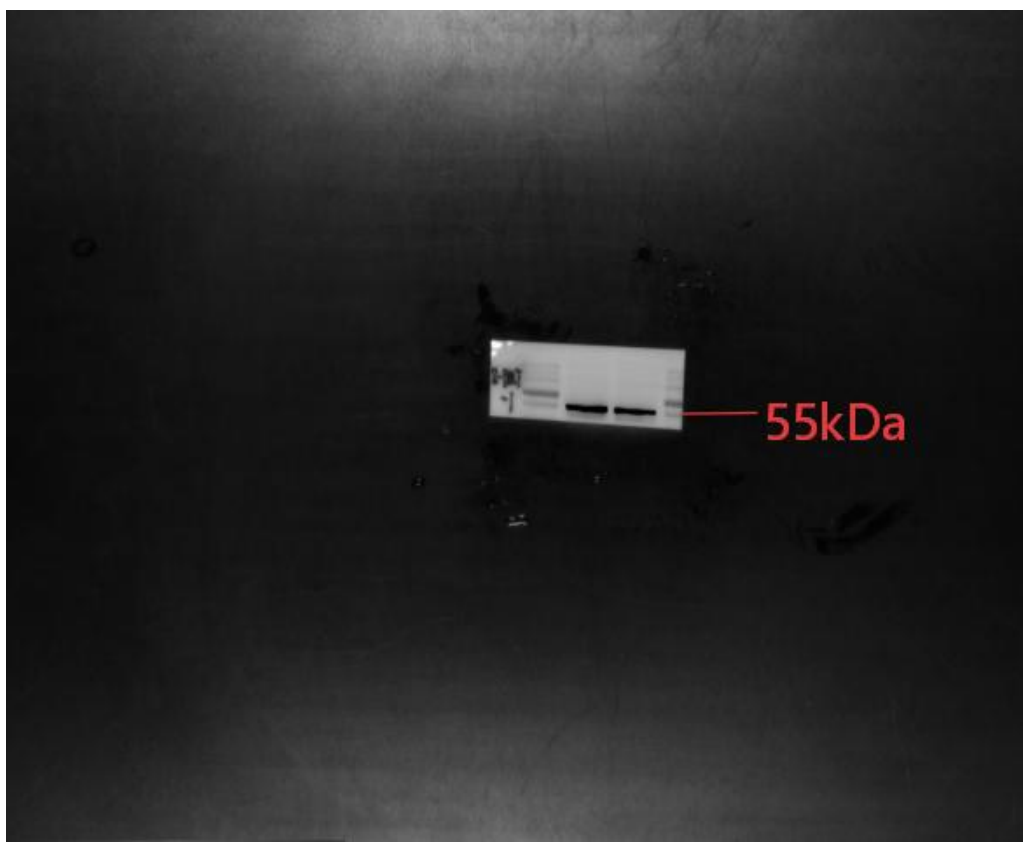

NLRP3 GSDMD

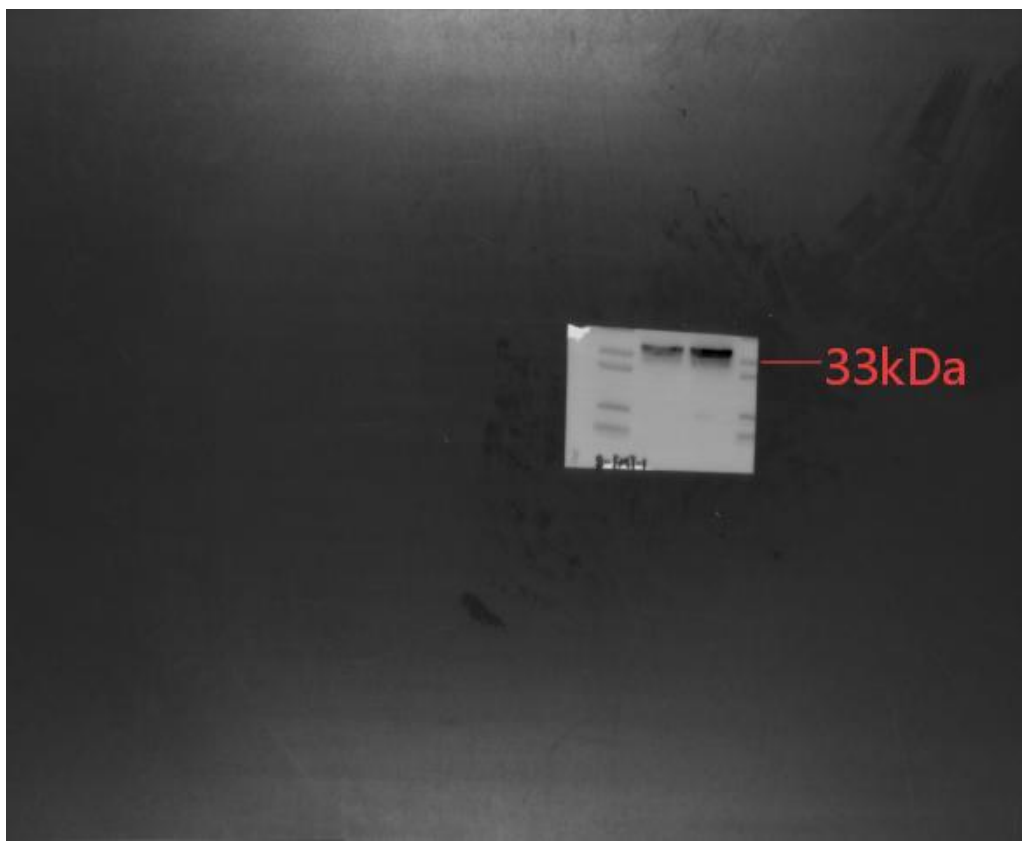

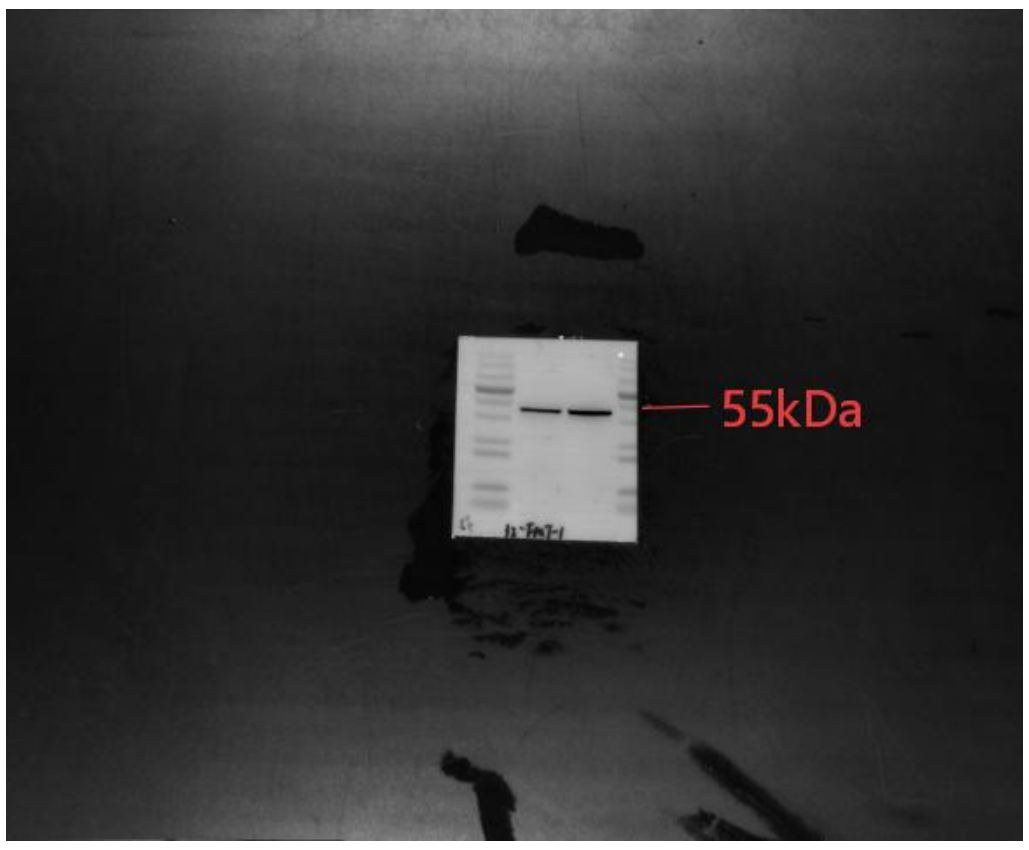

GSDMD-N Caspase-1

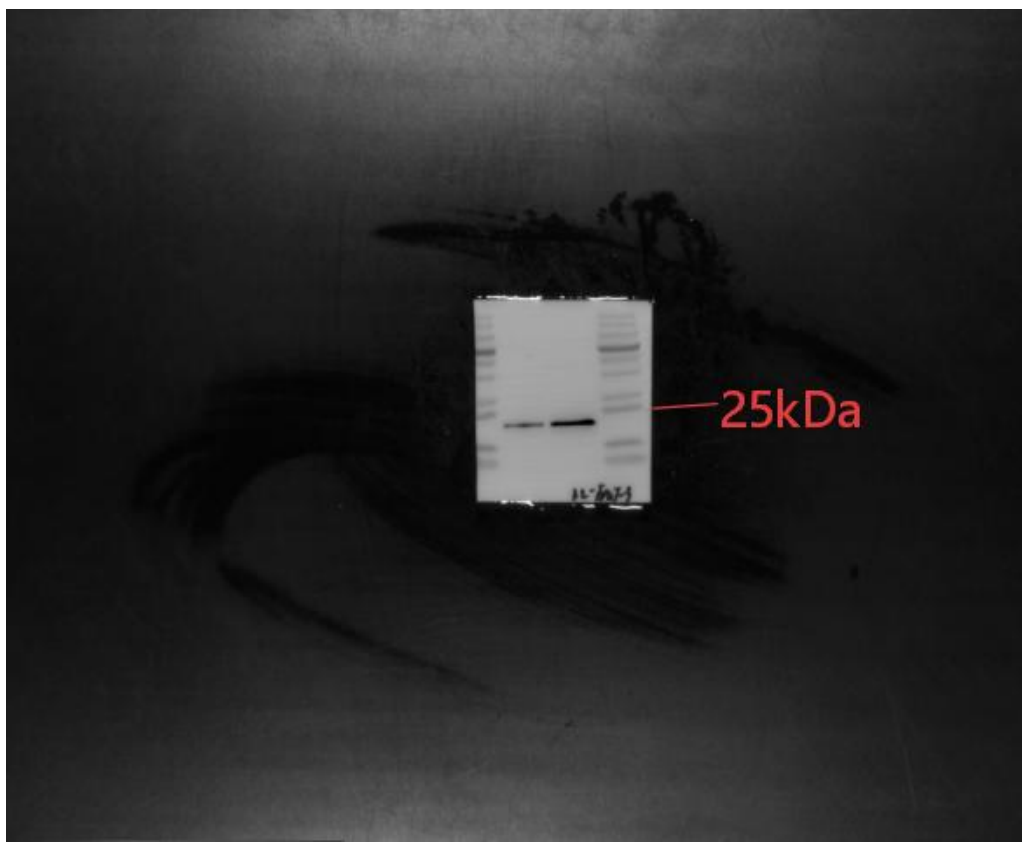

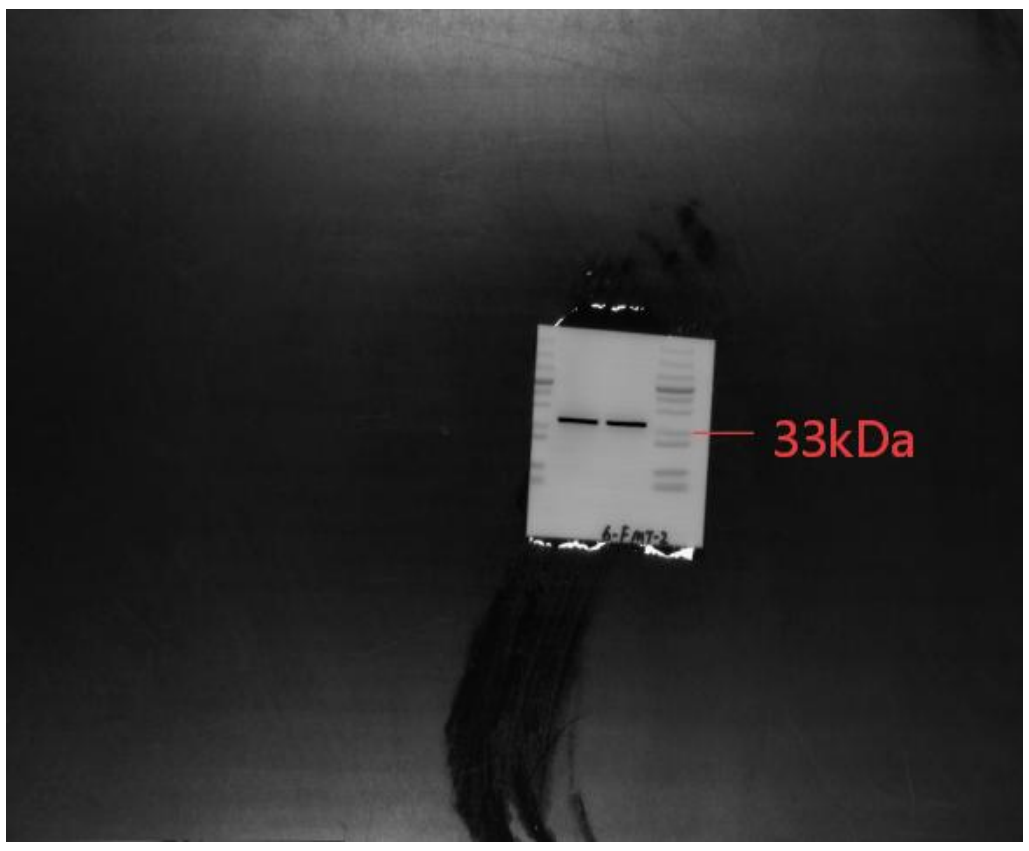

Cleaved Caspase-1 GAPDH

Figure 3D

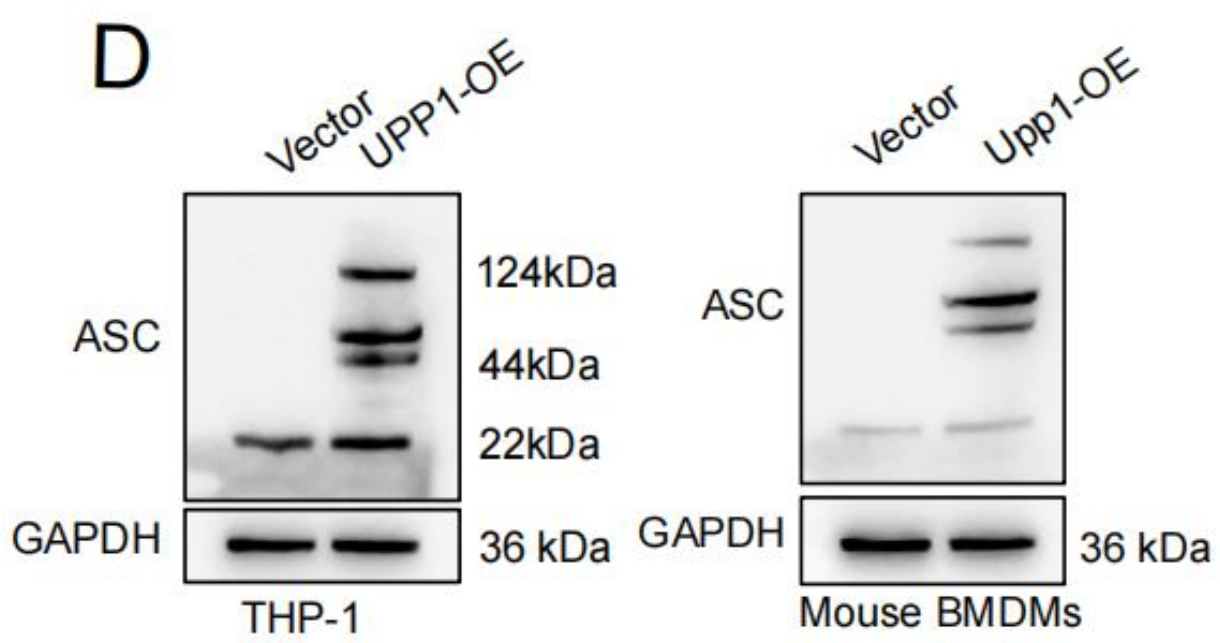

THP-1

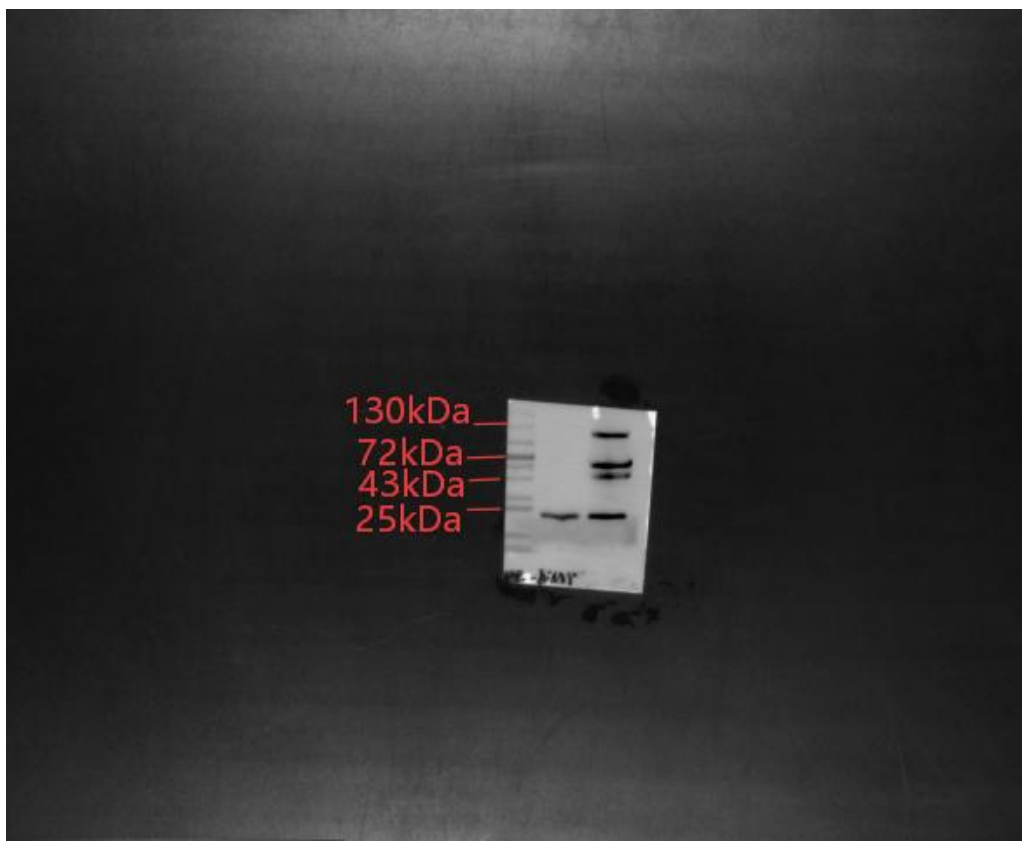

ASC

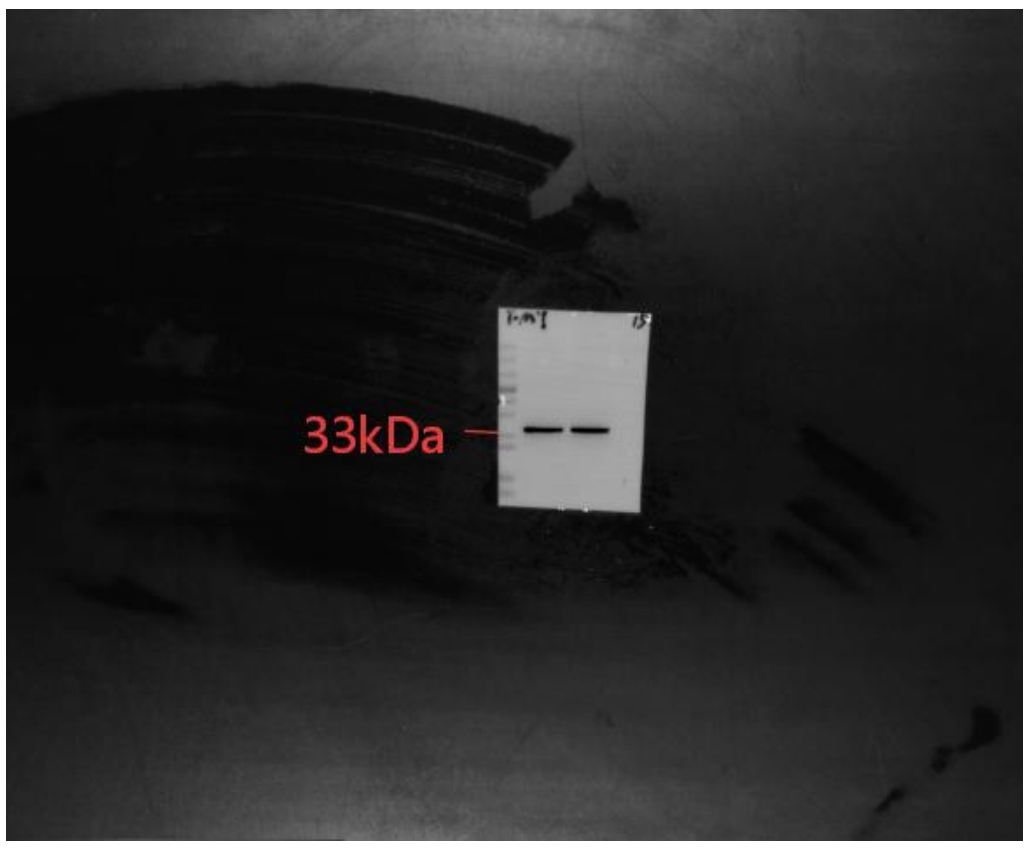

GAPDH

Mouse BMDMs

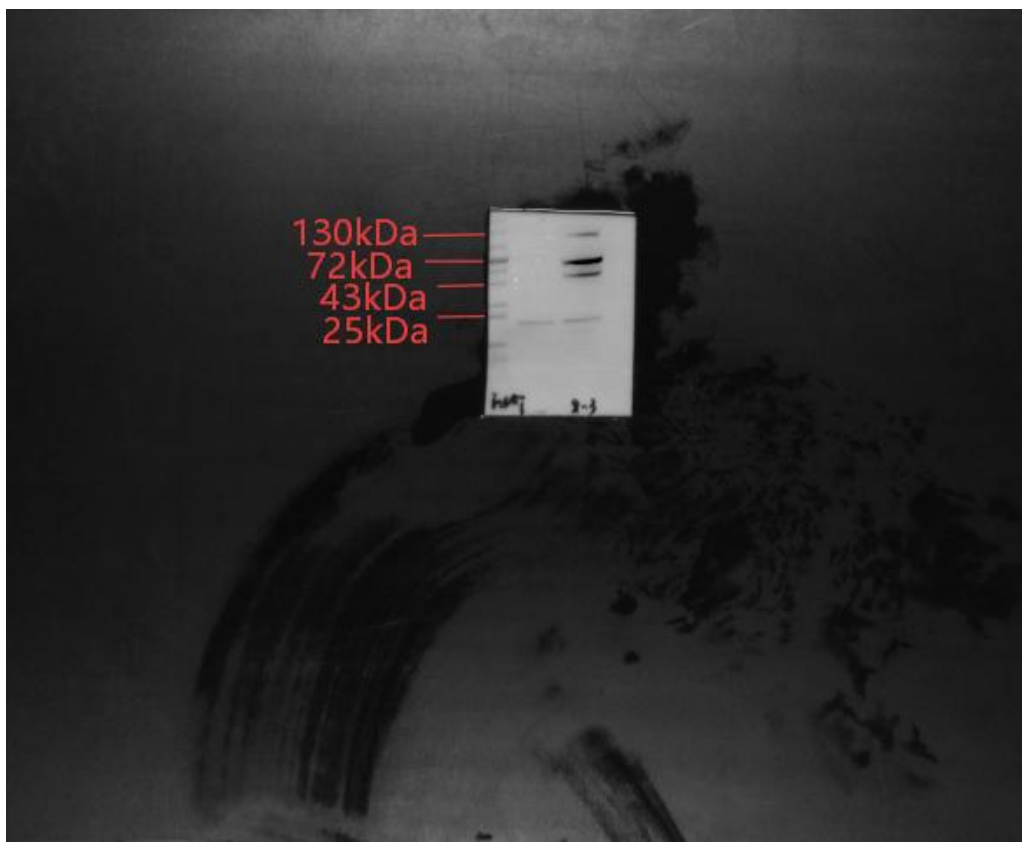

ASC

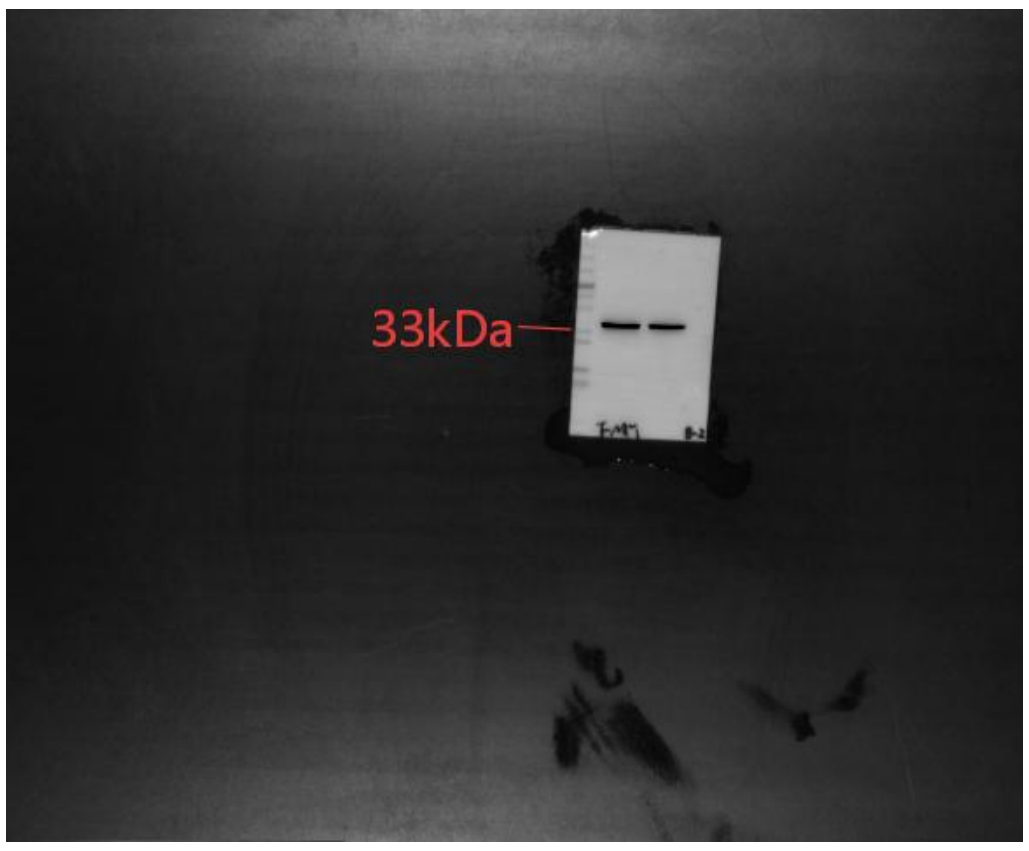

GAPDH

Figure 3J

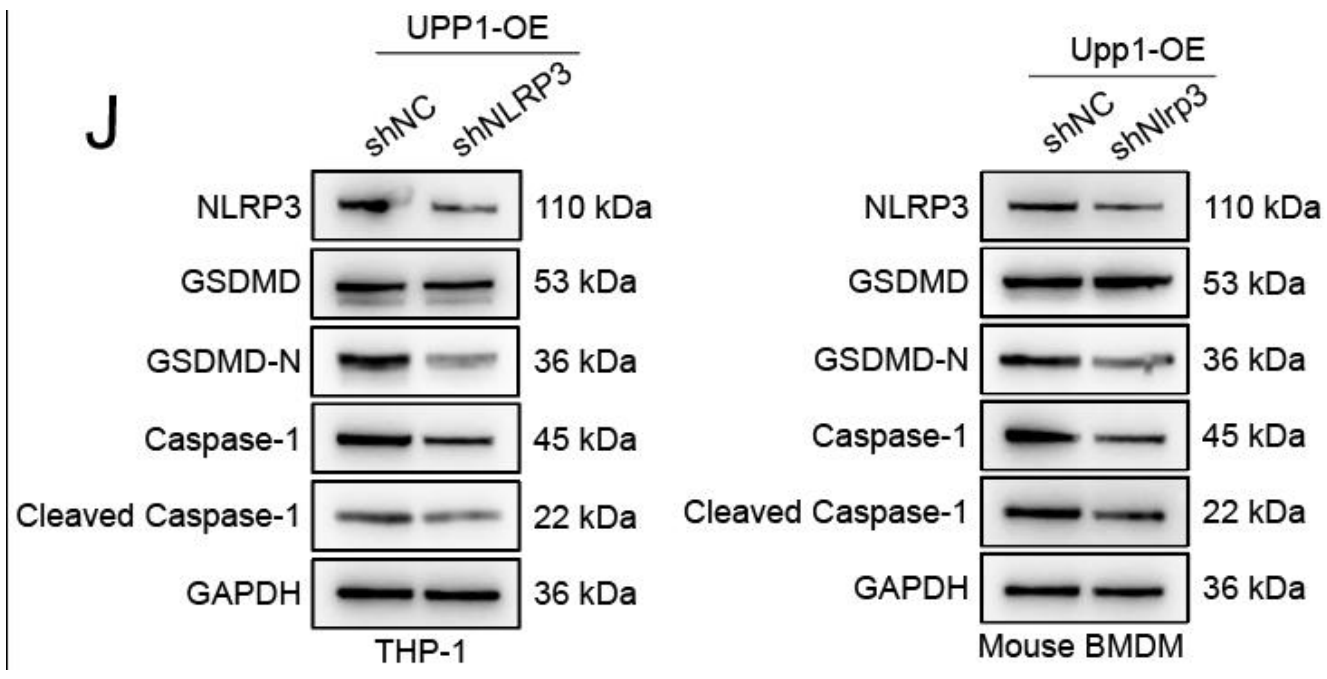

THP-1

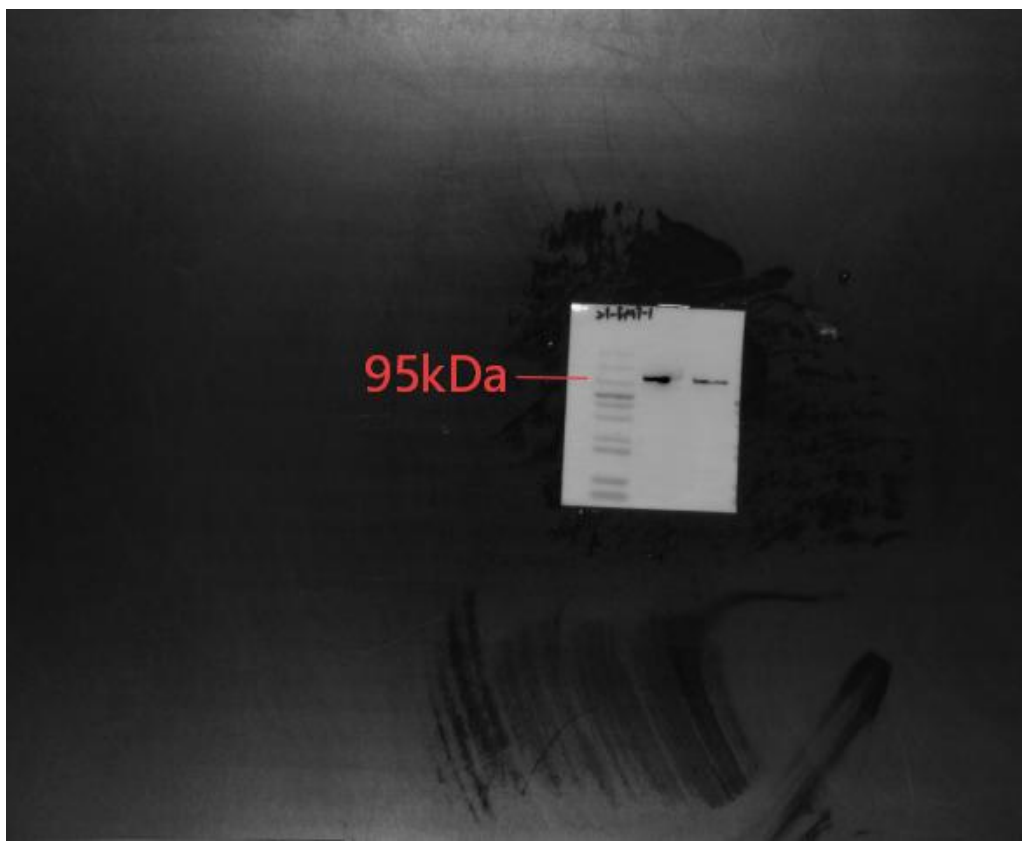

NLRP3

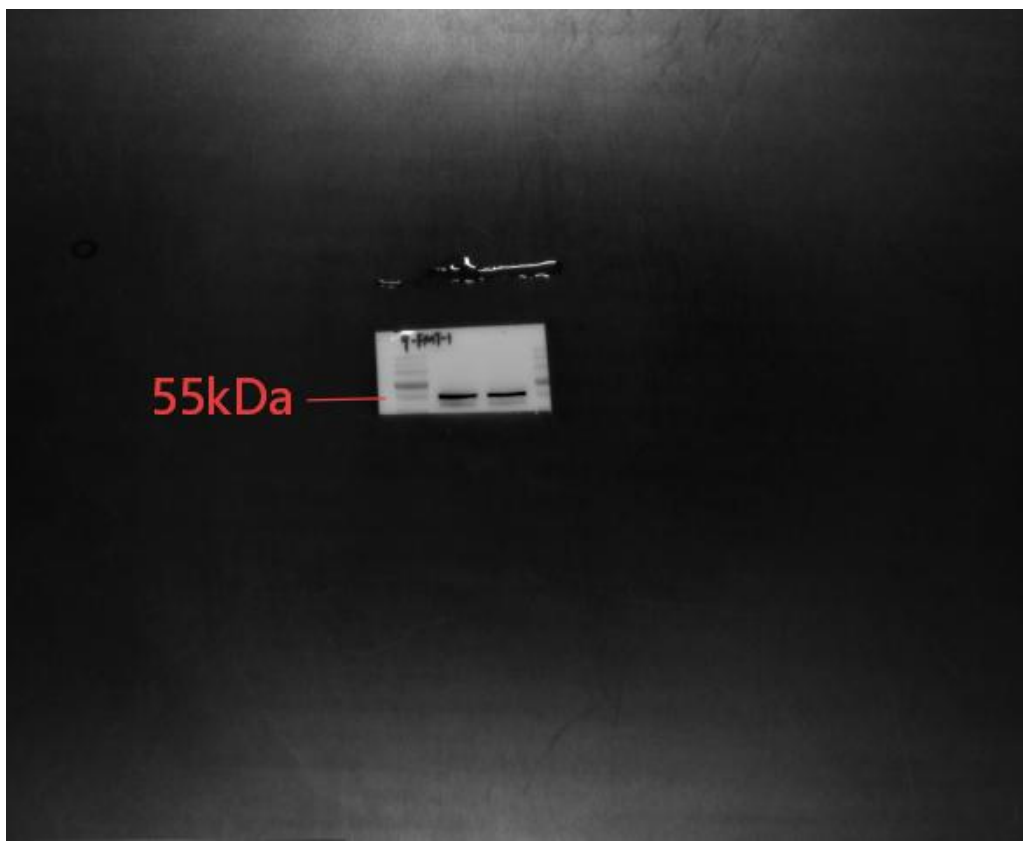

GSDMD

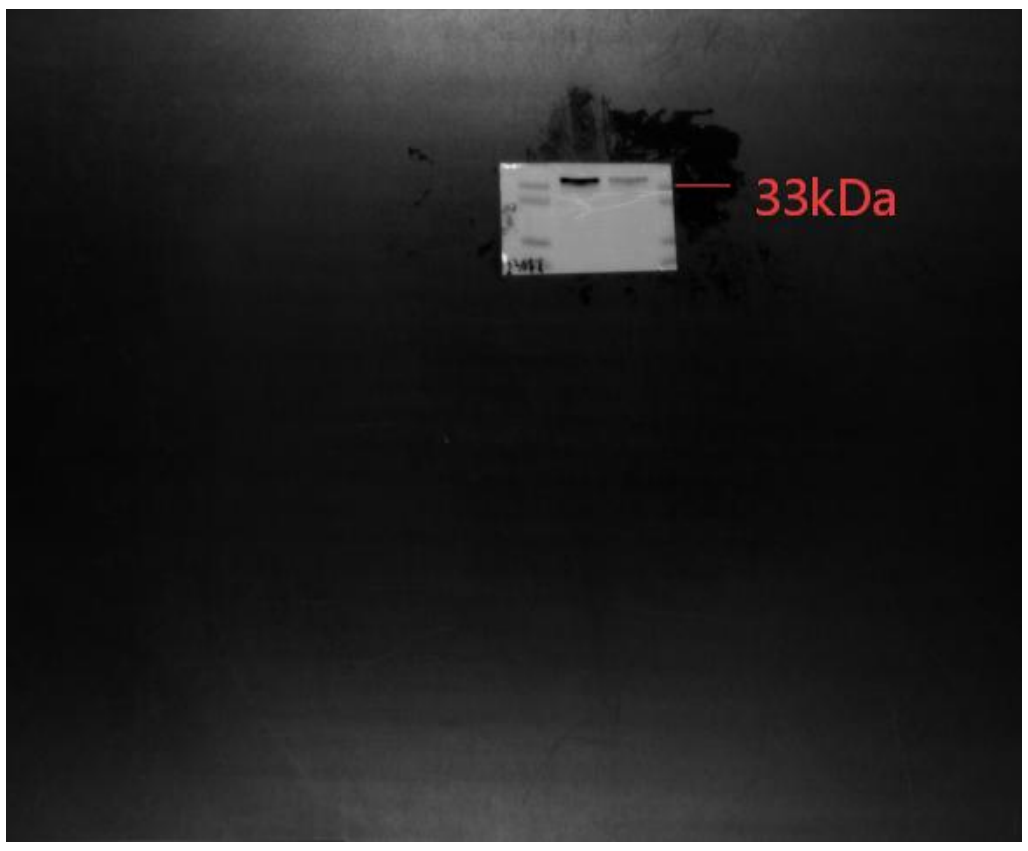

GSDMD-N

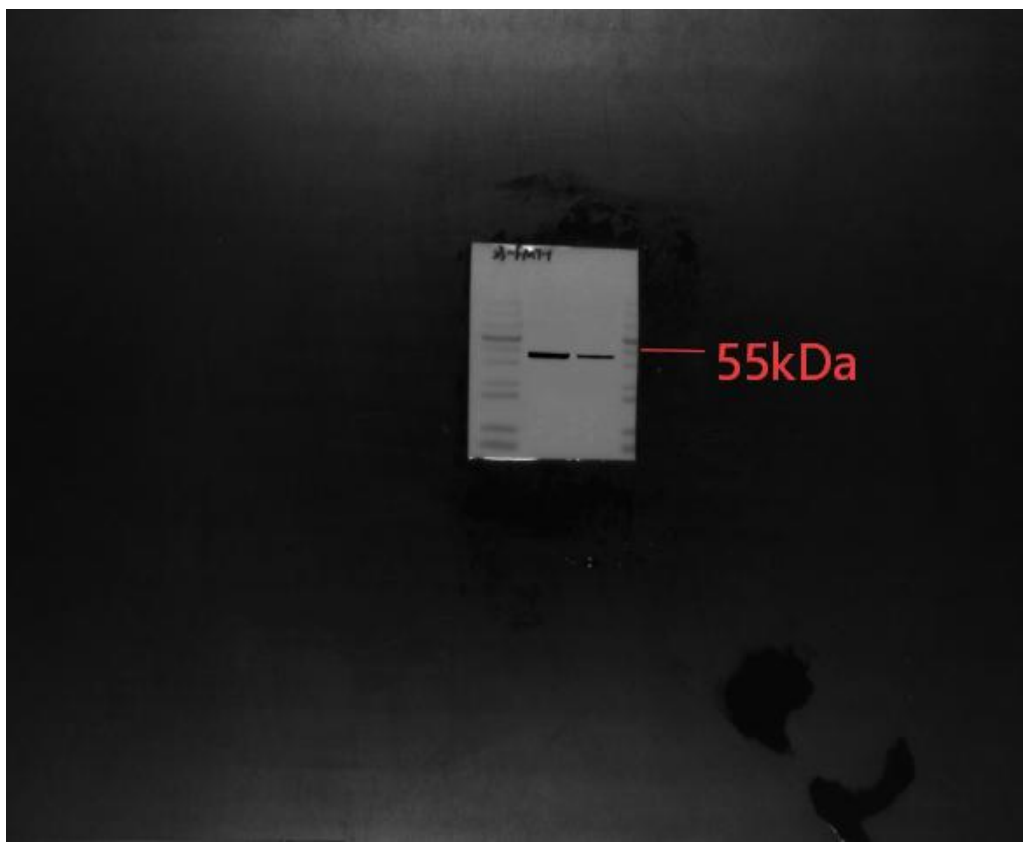

Caspase-1

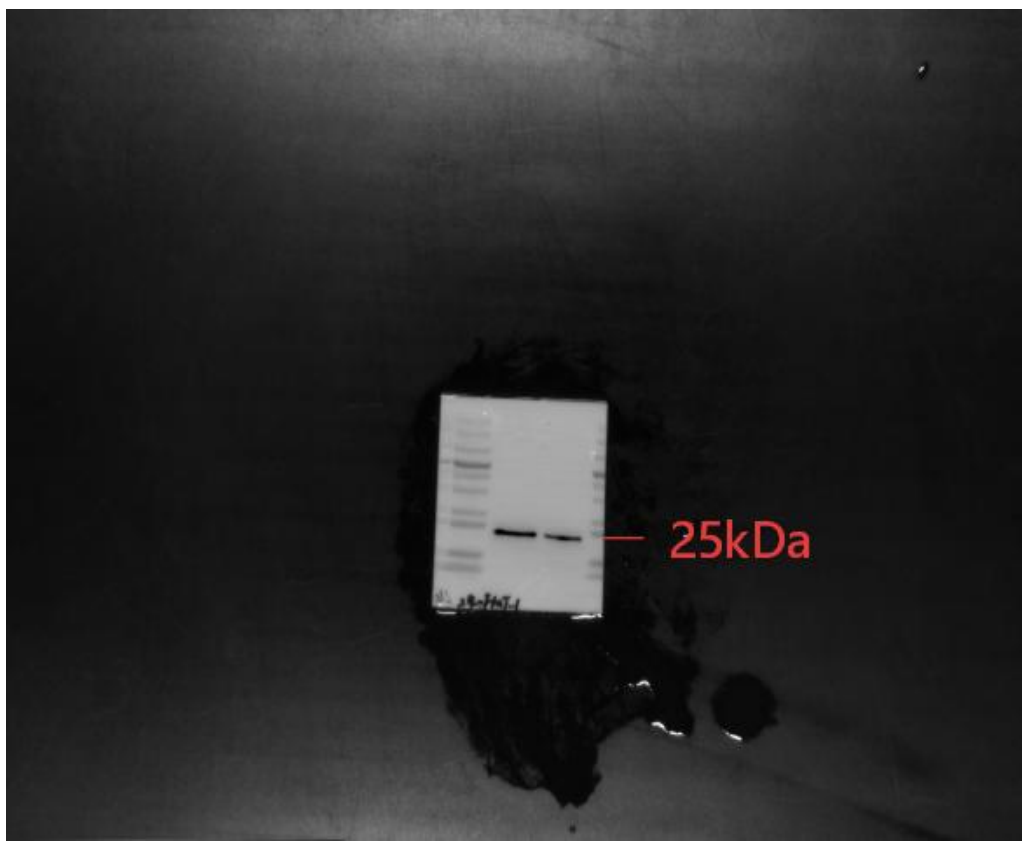

Cleaved Caspase-1

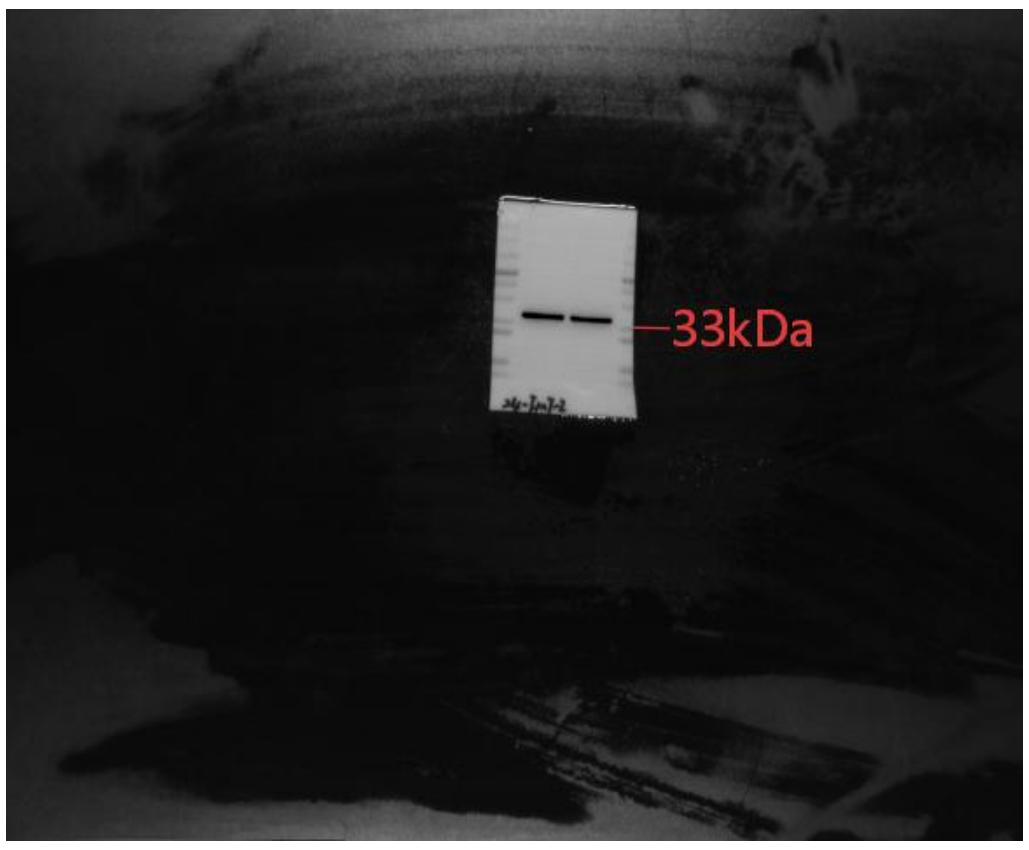

GAPDH

Mouse BMDMs

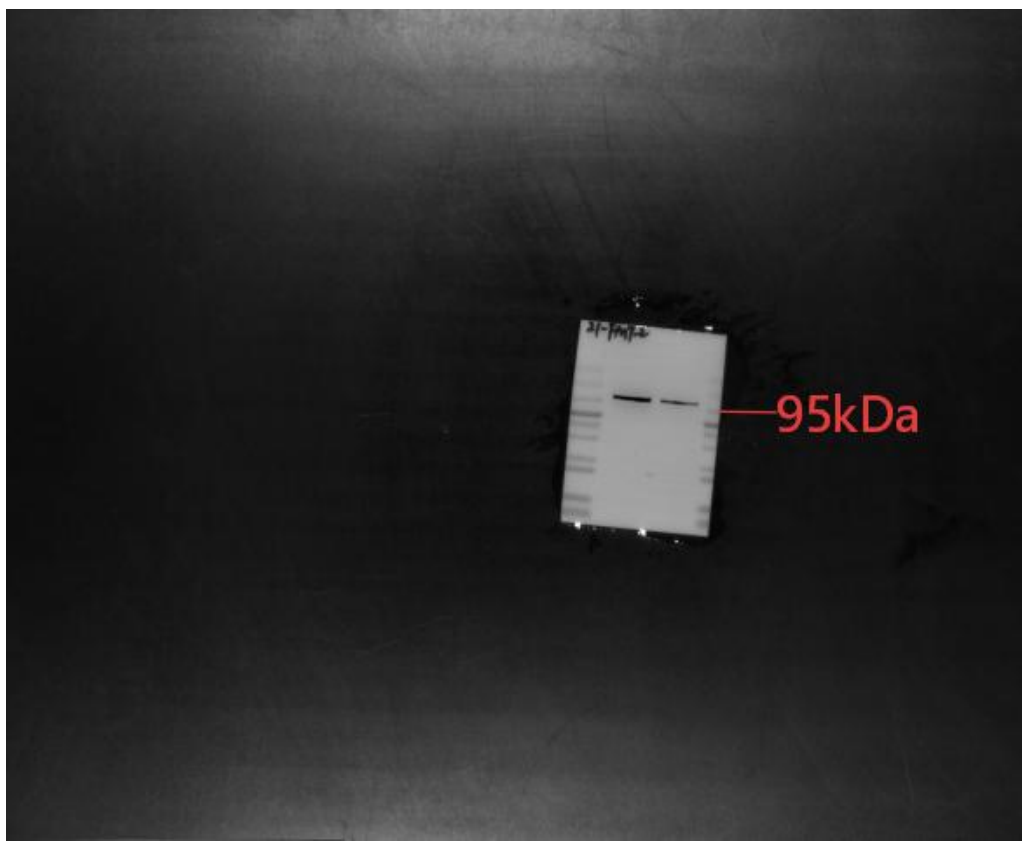

NLRP3

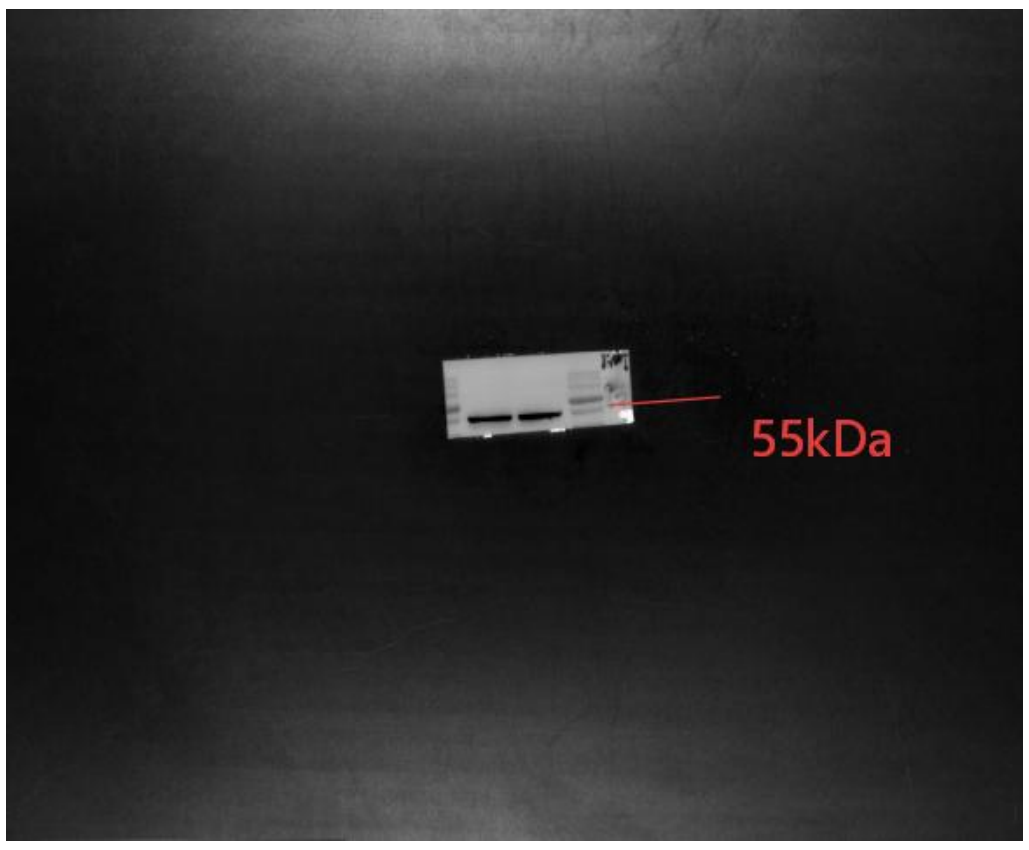

GSDMD

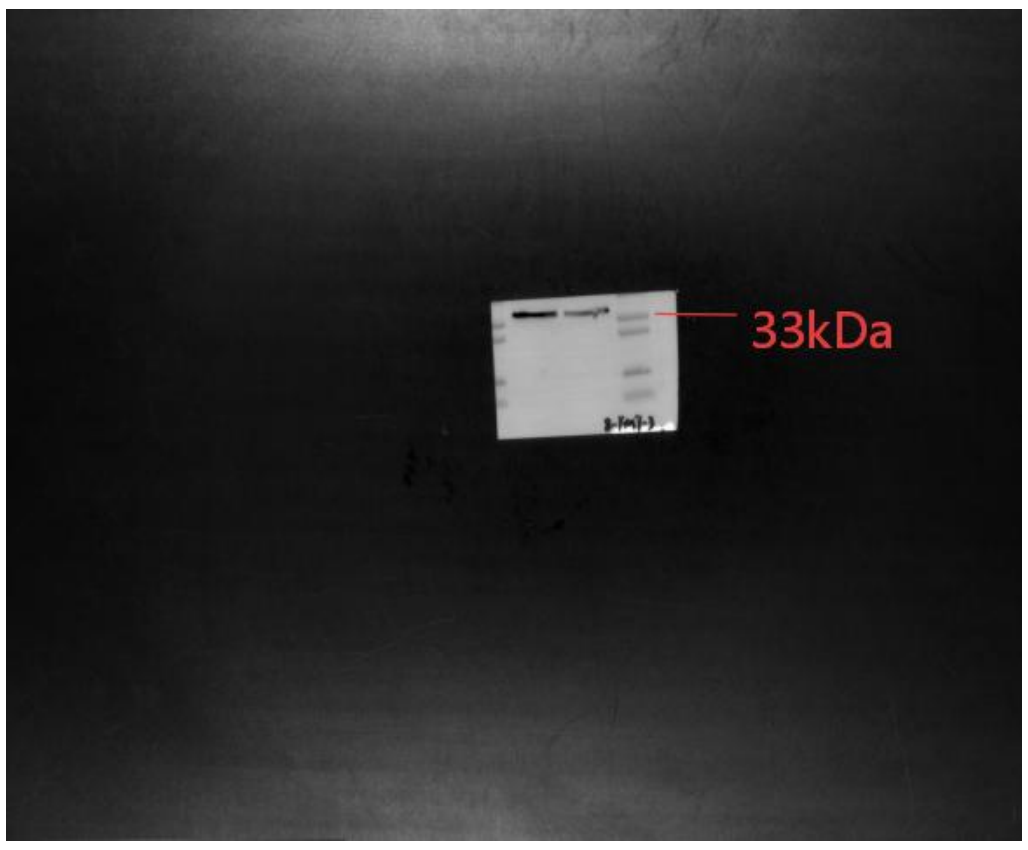

GSDMD-N

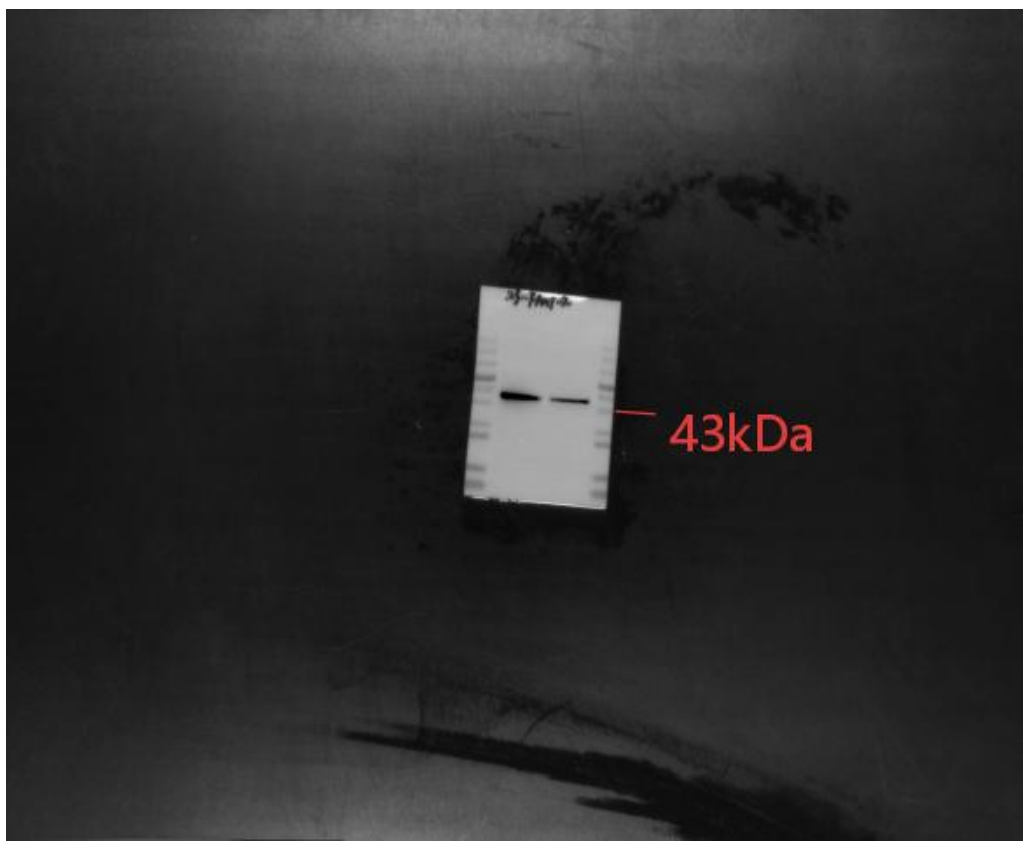

Caspase-1

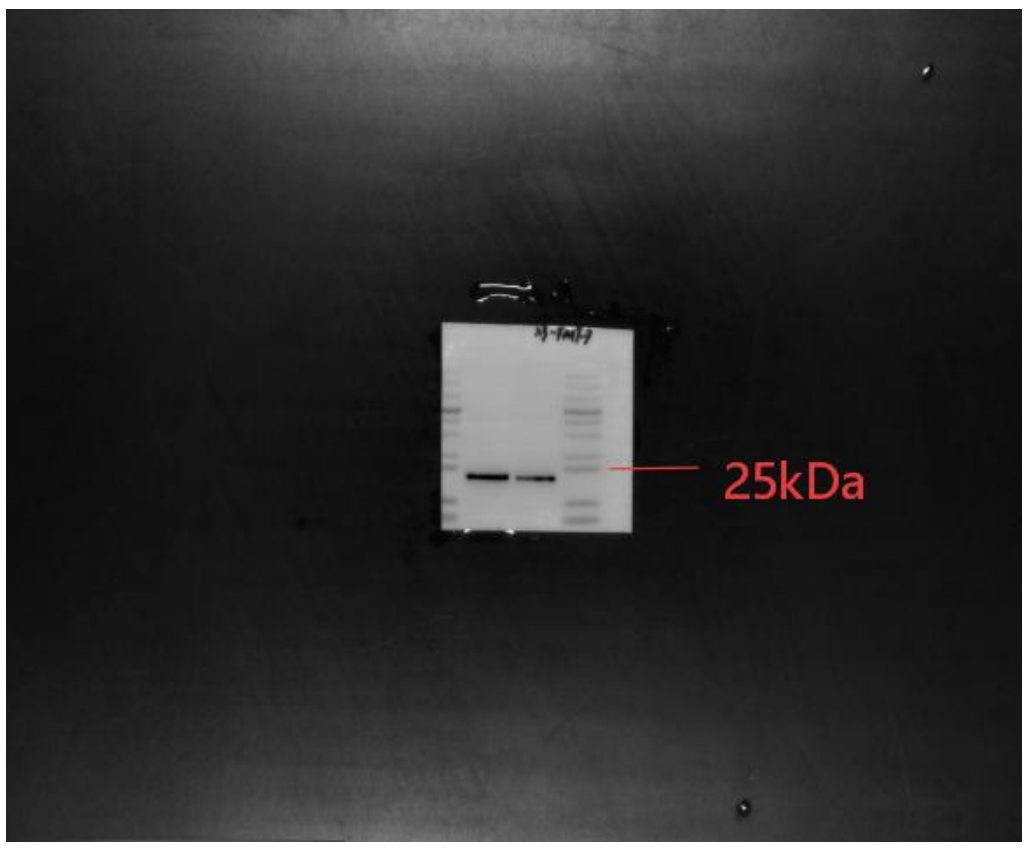

Cleaved Caspase-1

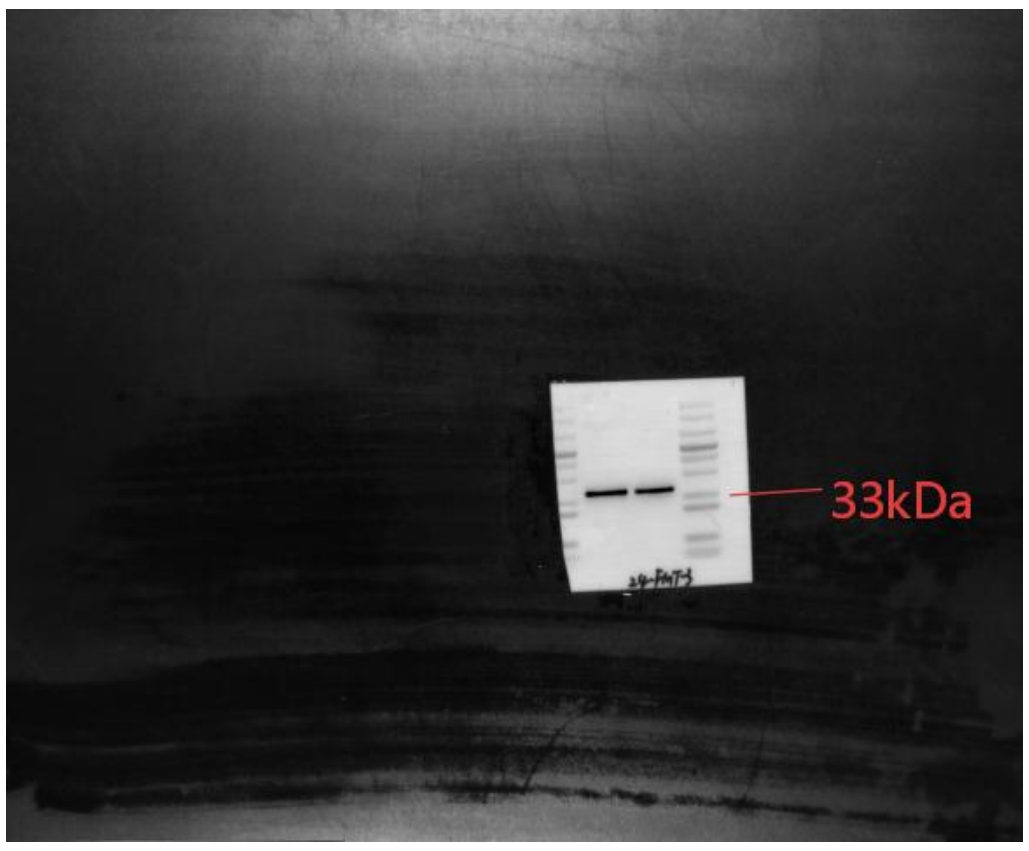

GAPDH

Figure 3K

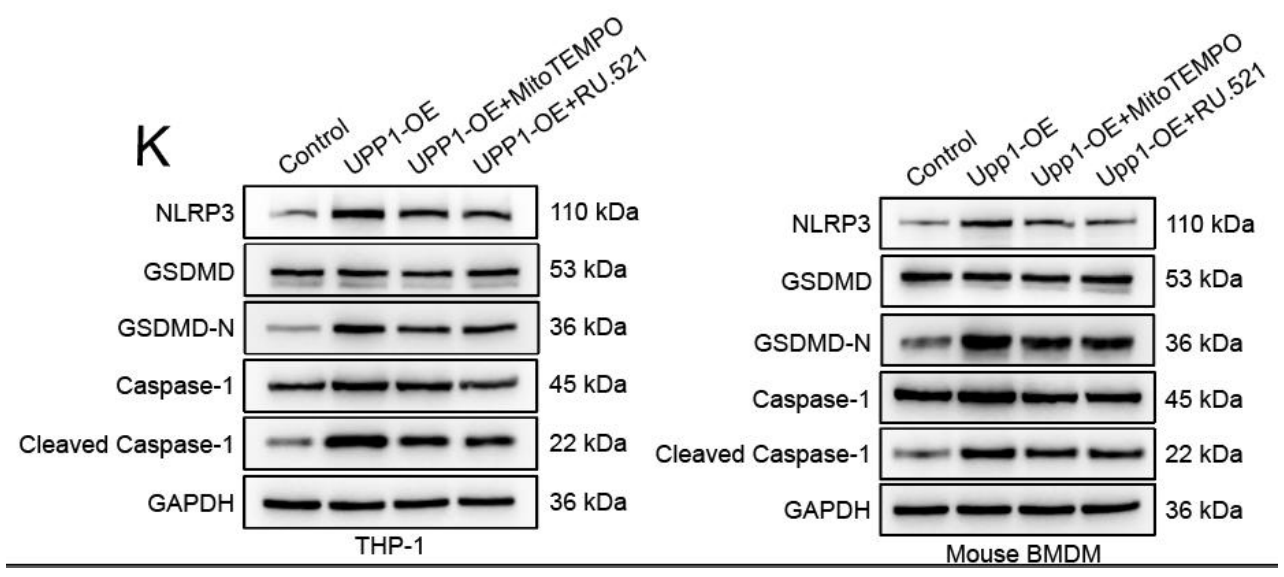

THP-1

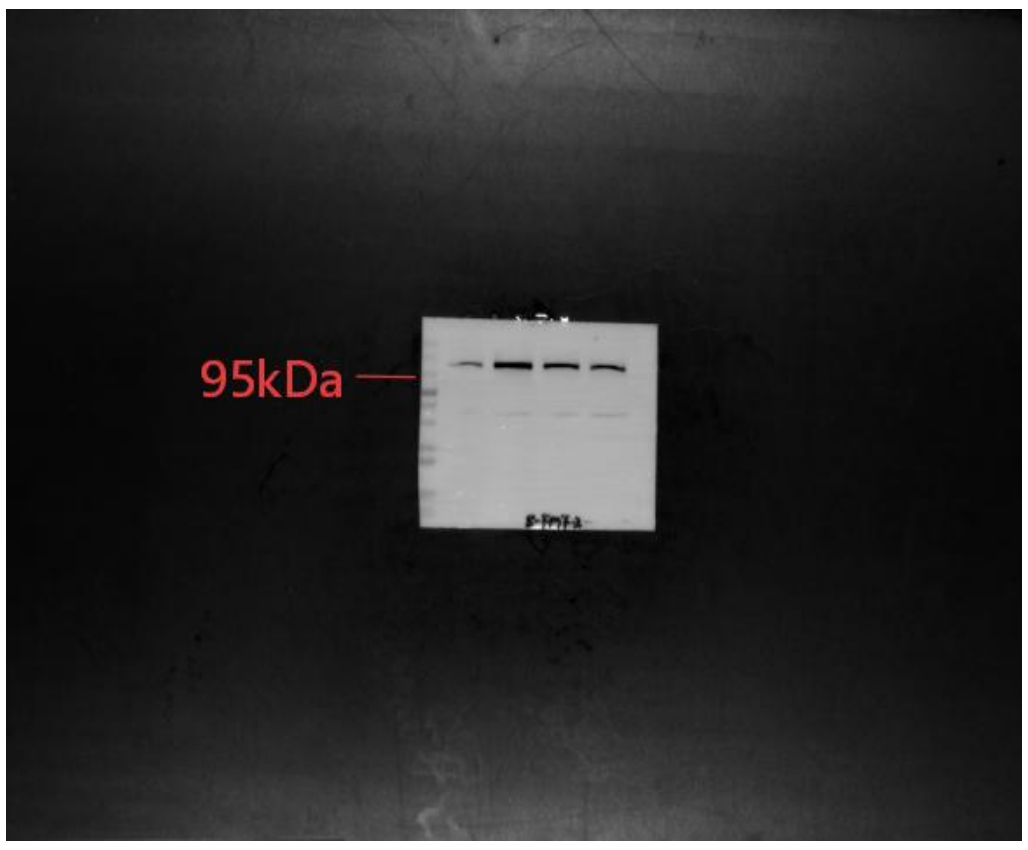

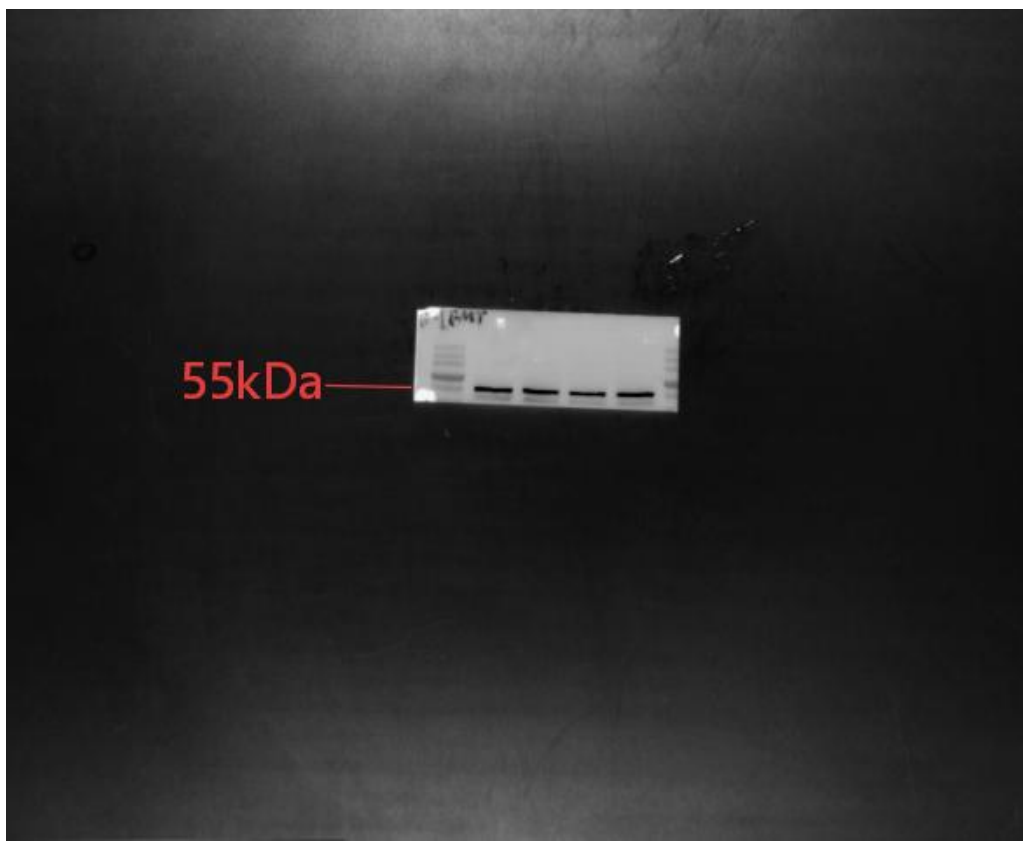

NLRP3

GSDMD

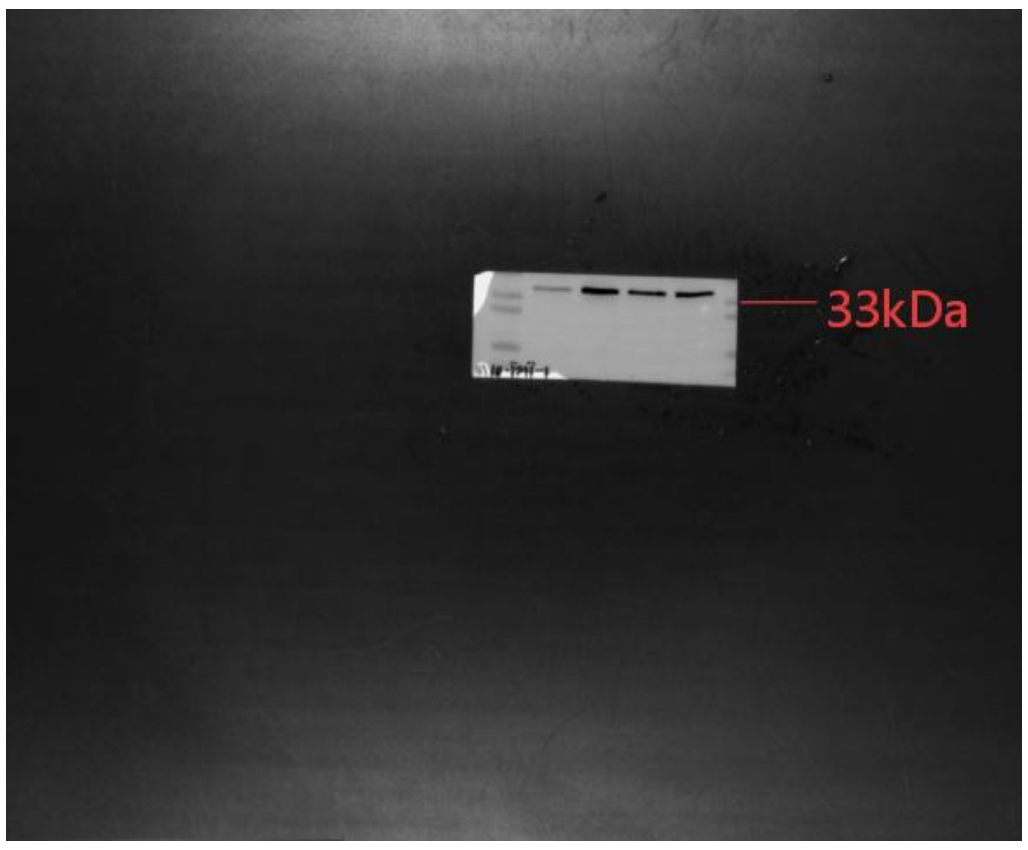

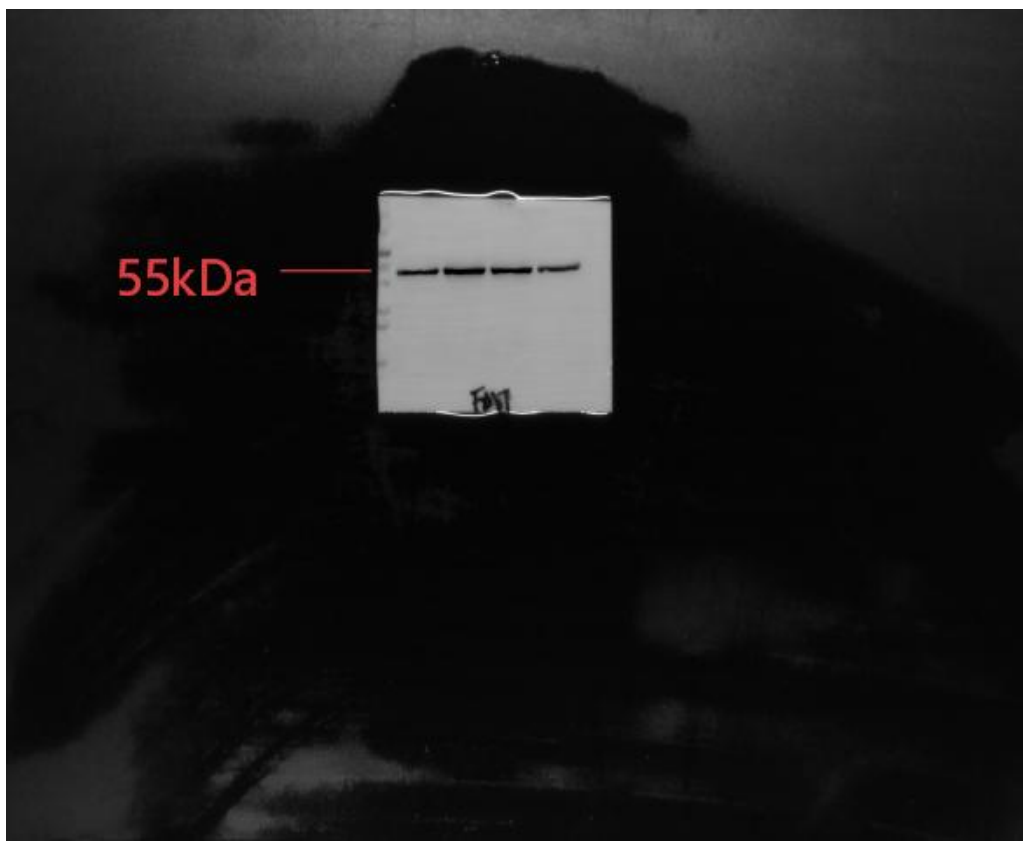

GSDMD-N

Caspase-1

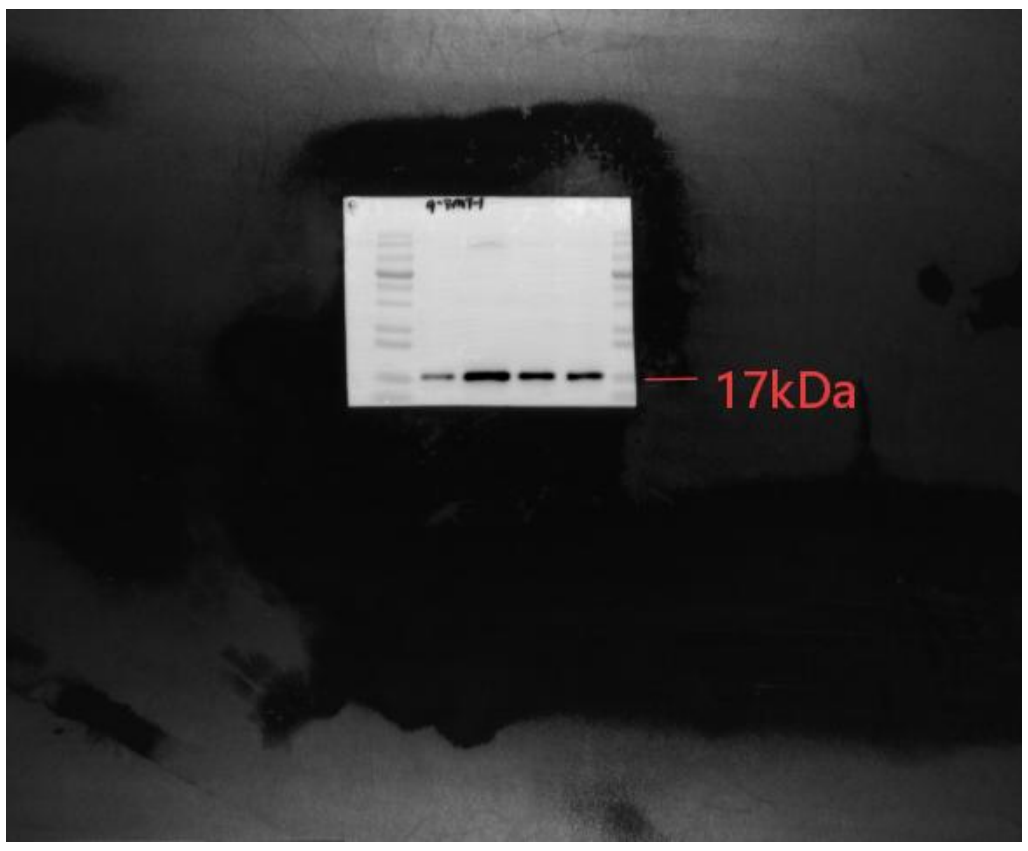

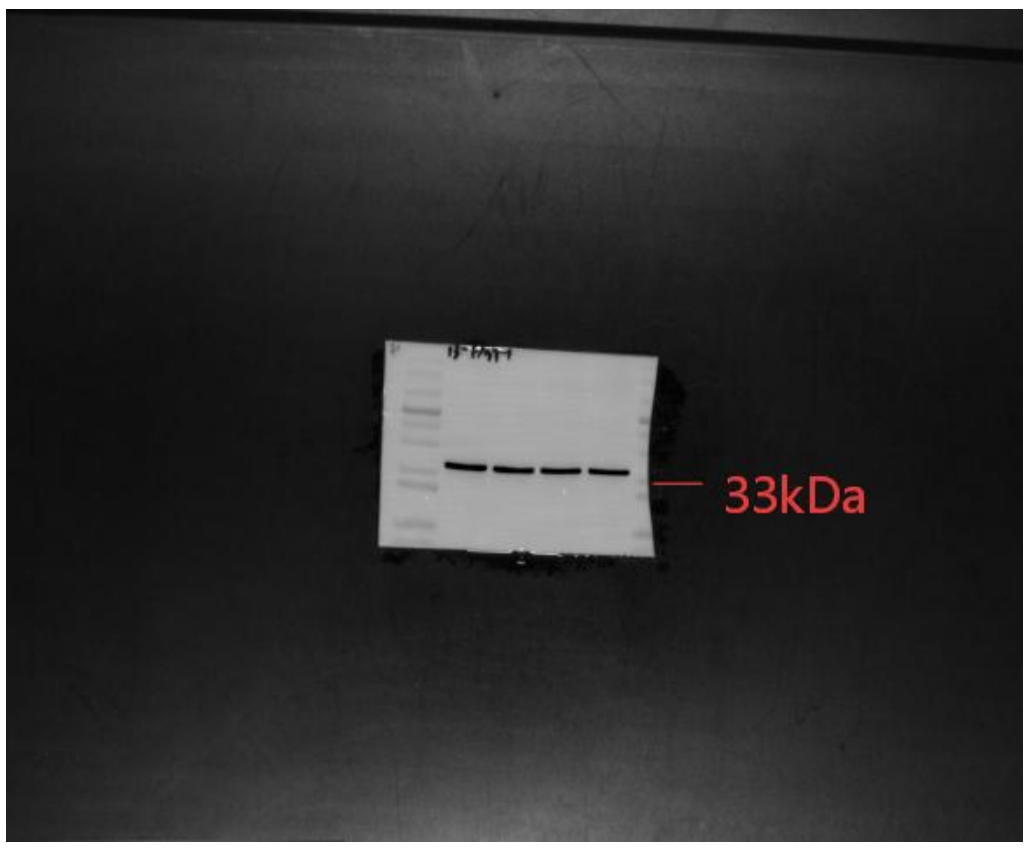

Cleaved Caspase-1

GAPDH

Mouse BMDMs

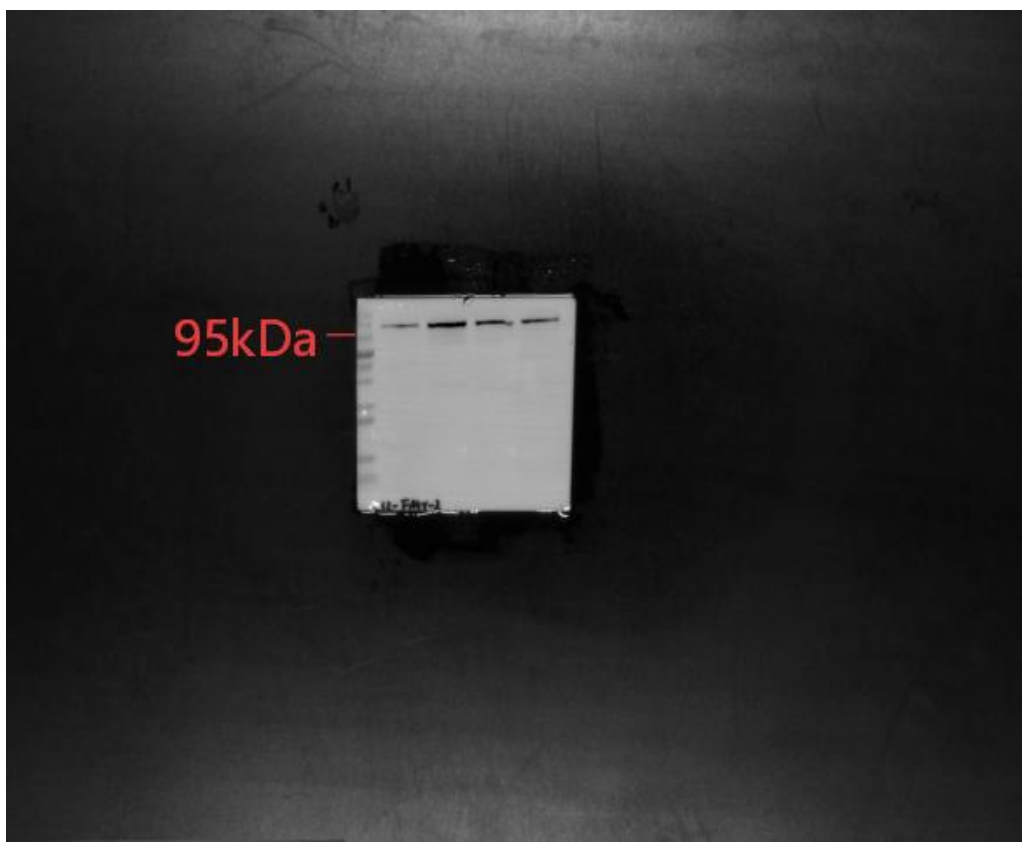

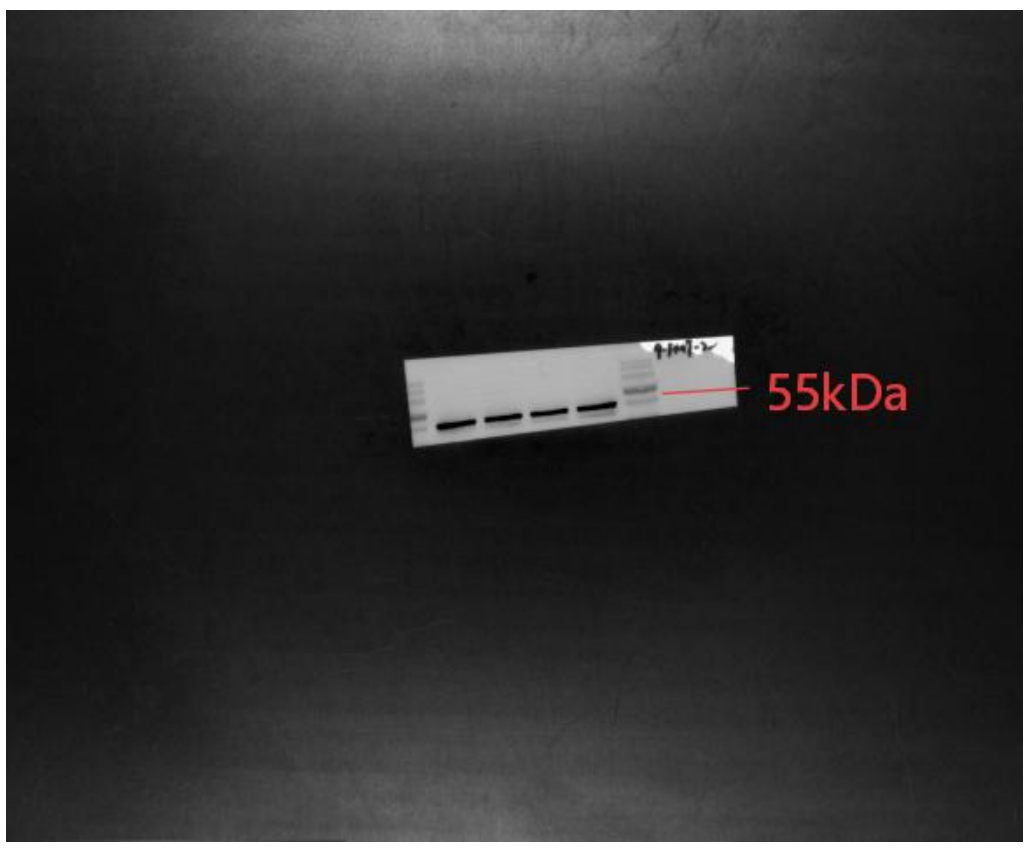

NLRP3

GSDMD

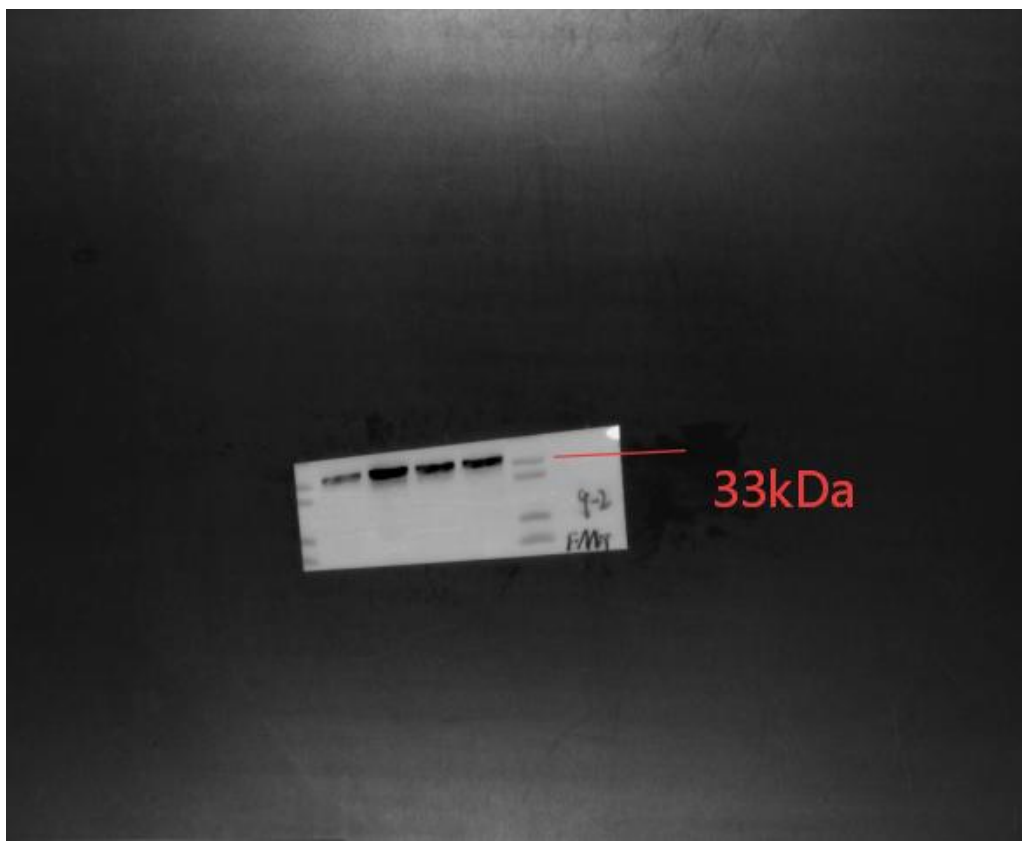

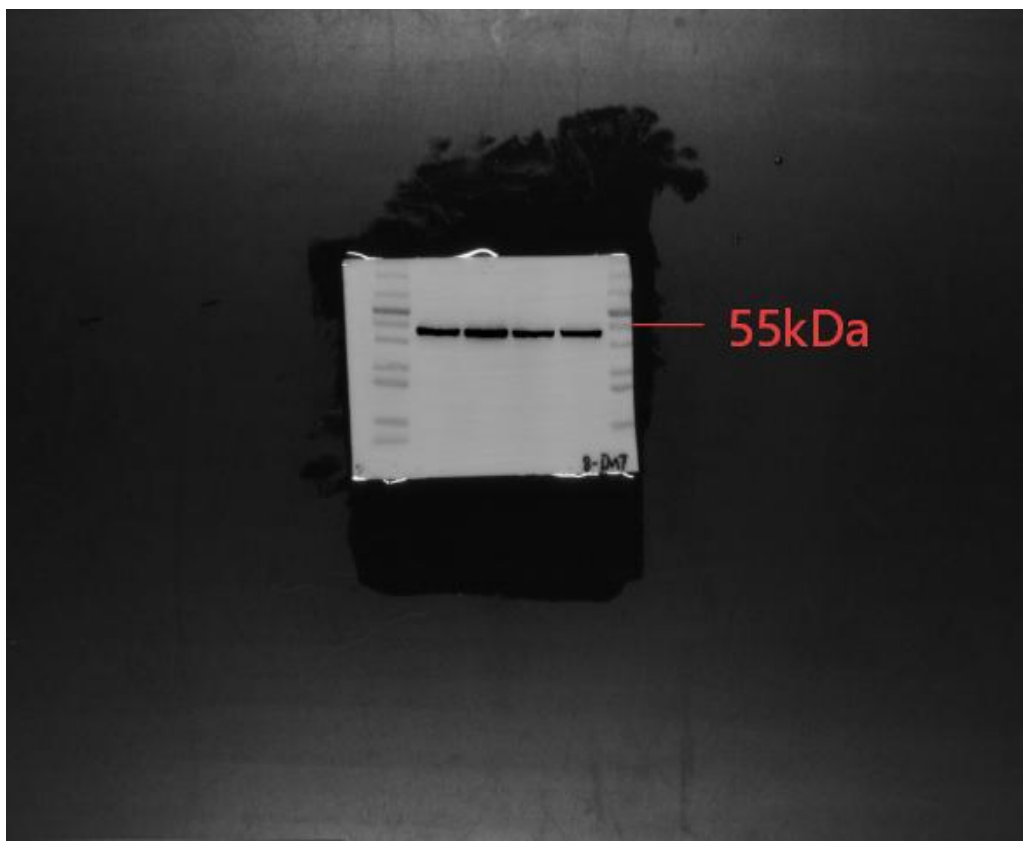

GSDMD-N

Caspase-1

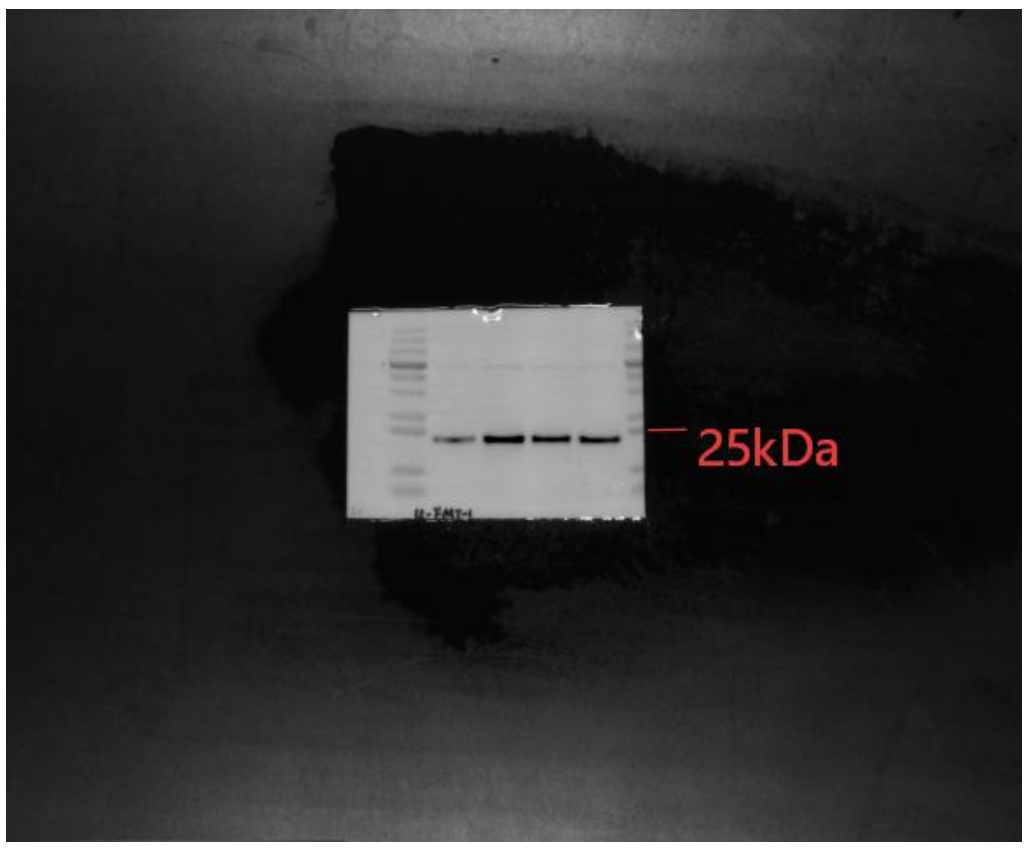

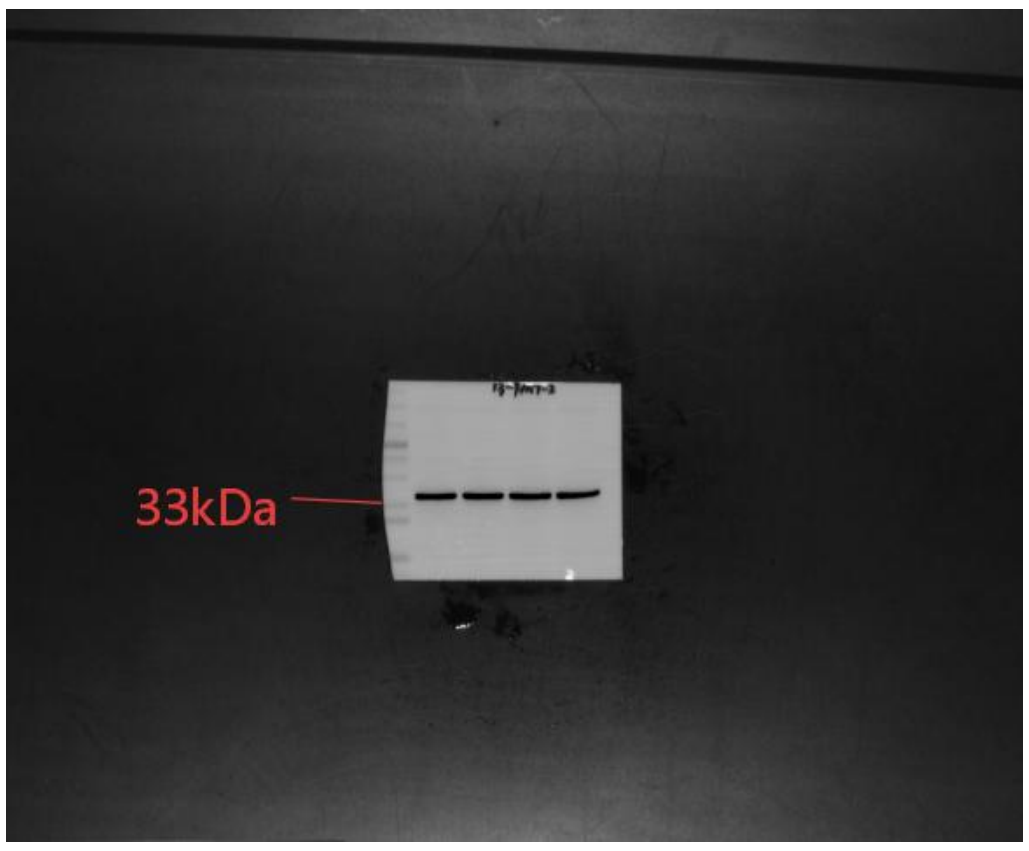

Cleaved Caspase-1

GAPDH

Figure 4E

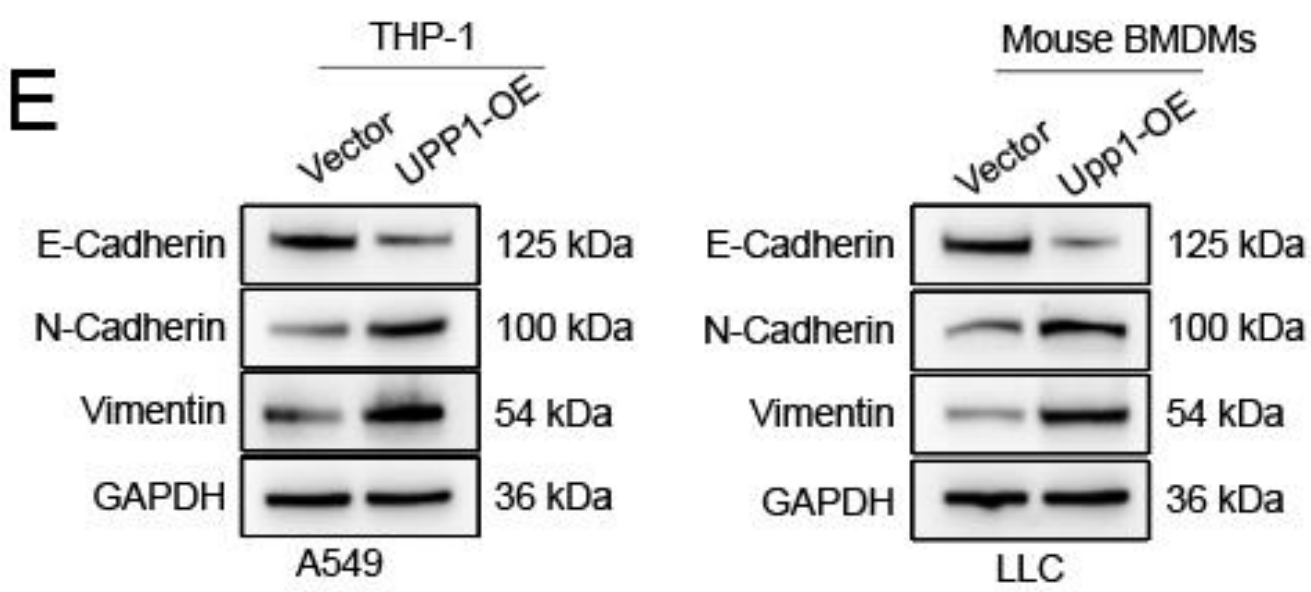

A549

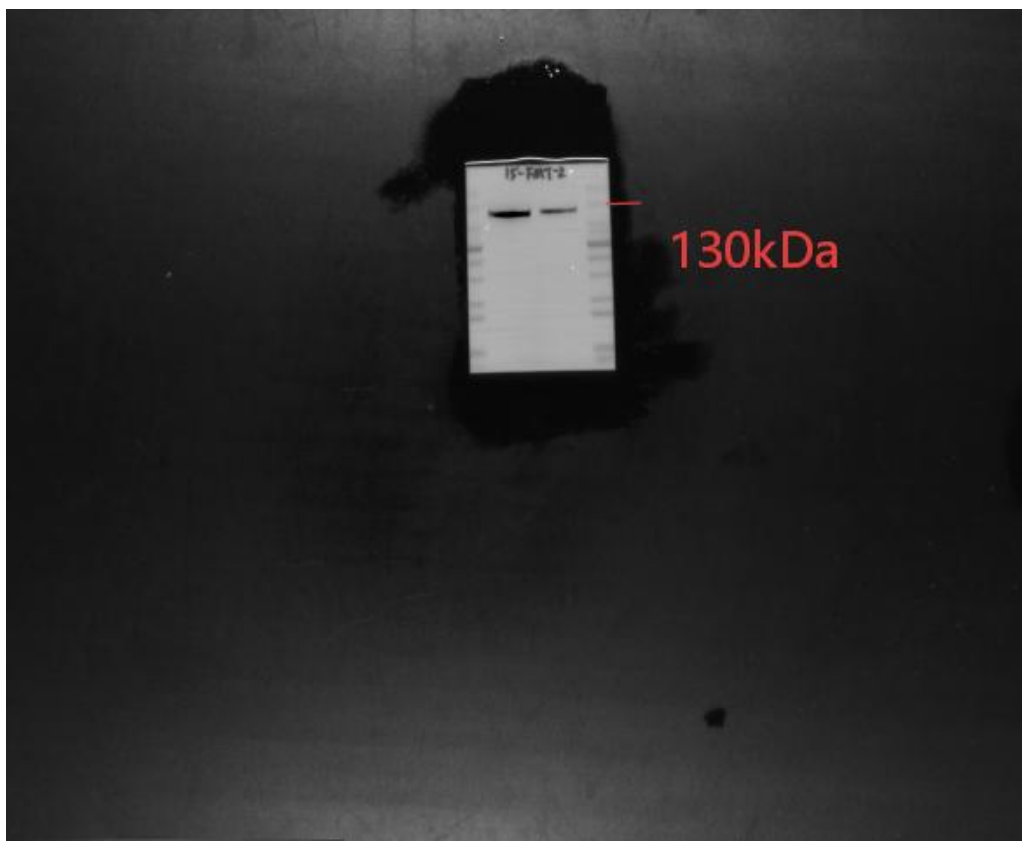

E-Cadherin

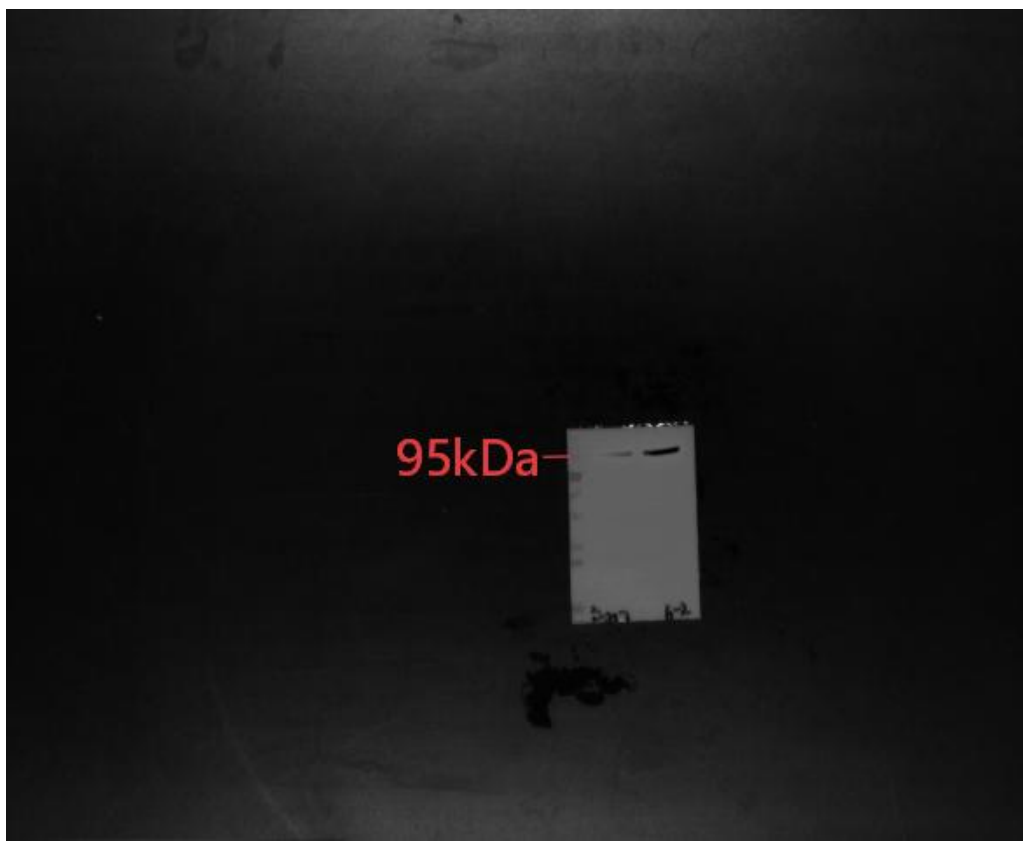

N-Cadherin

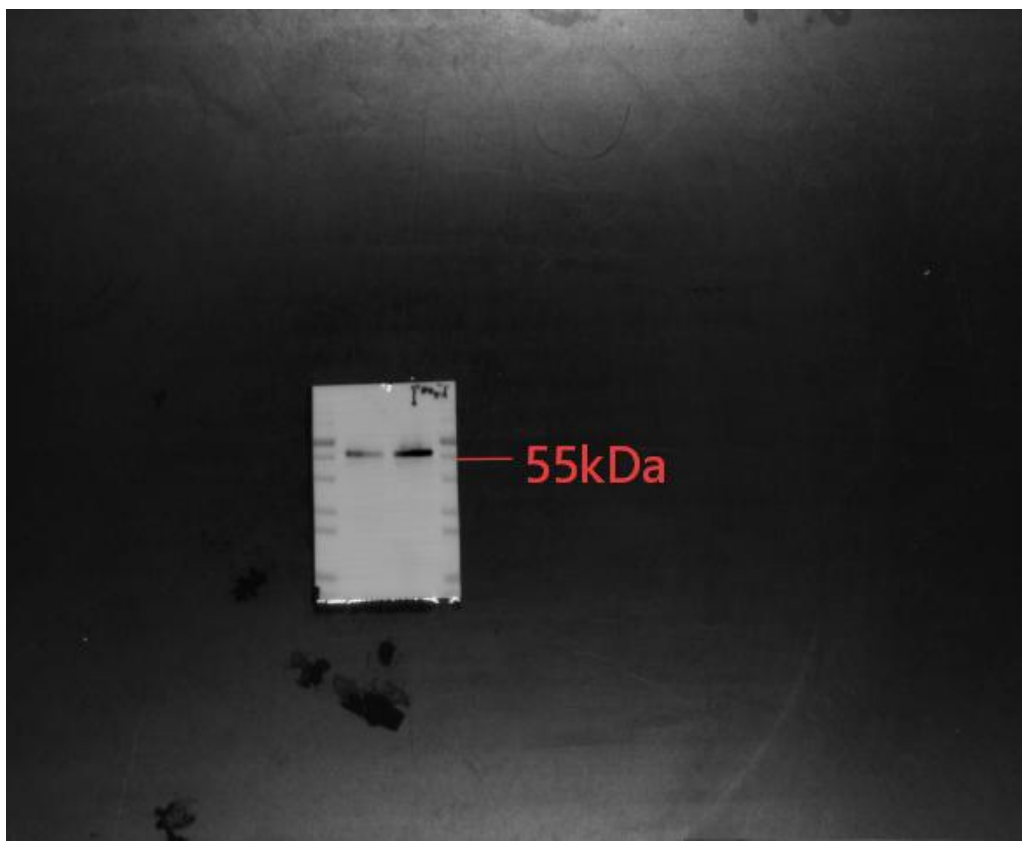

Vimentin

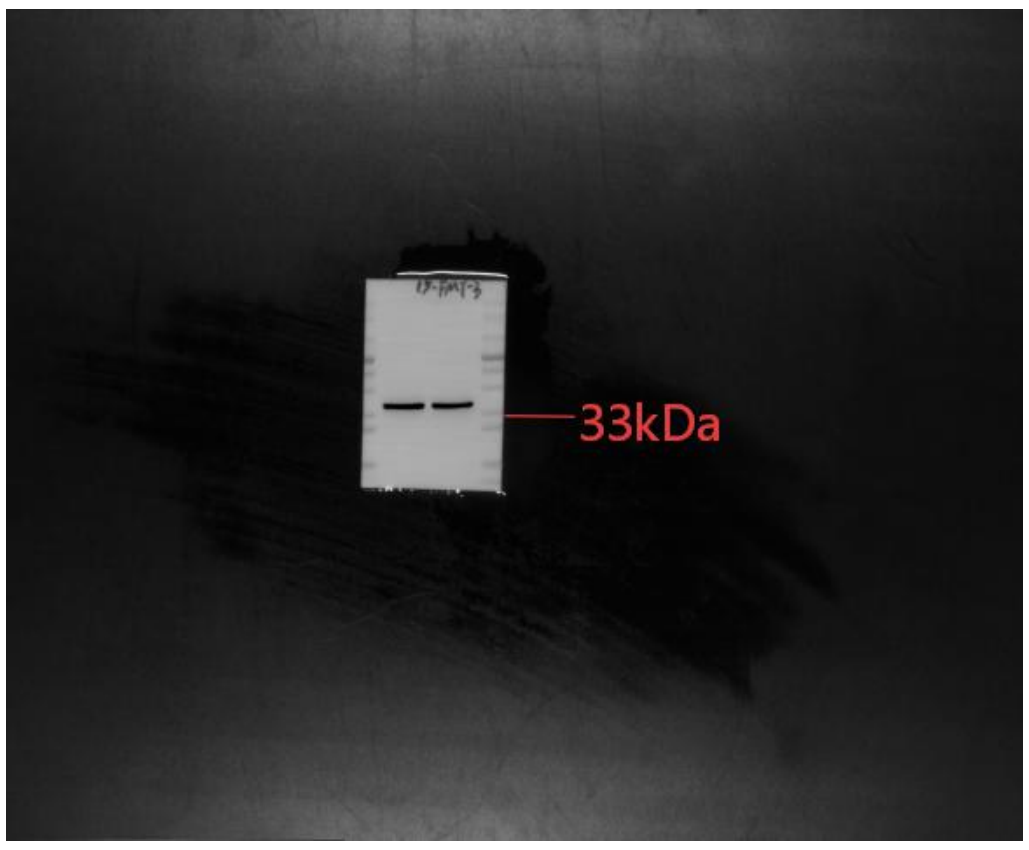

GAPDH

LLC

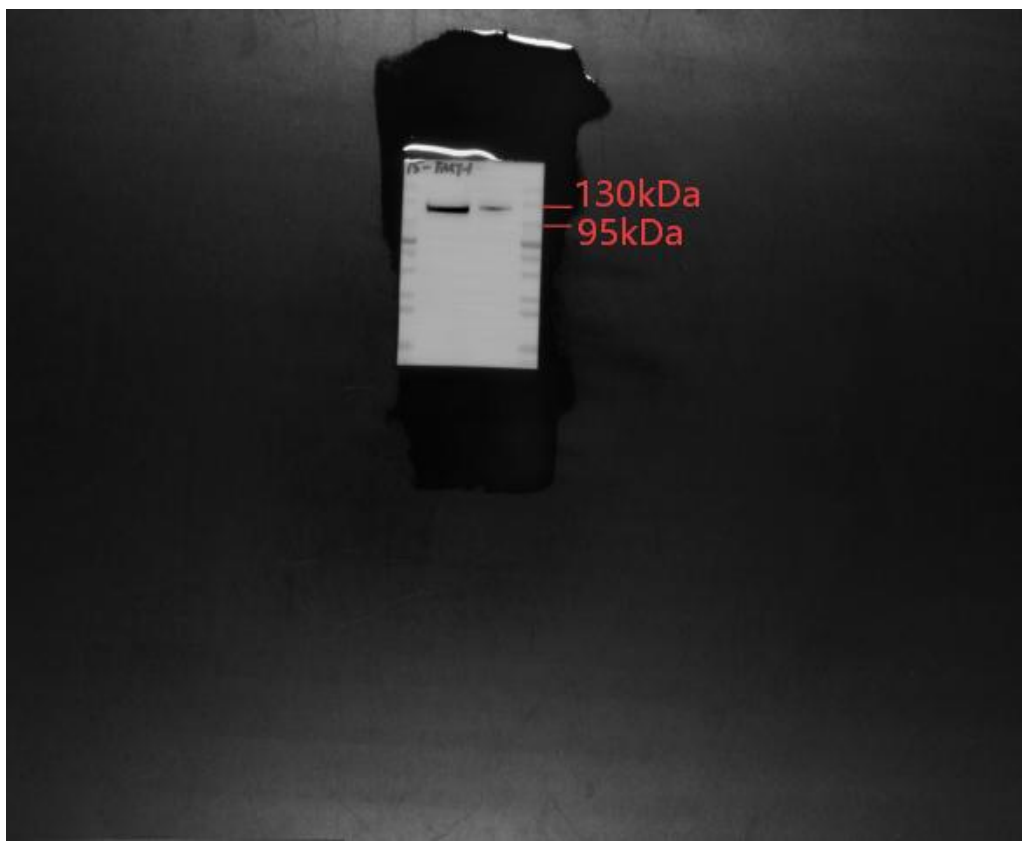

E-Cadherin

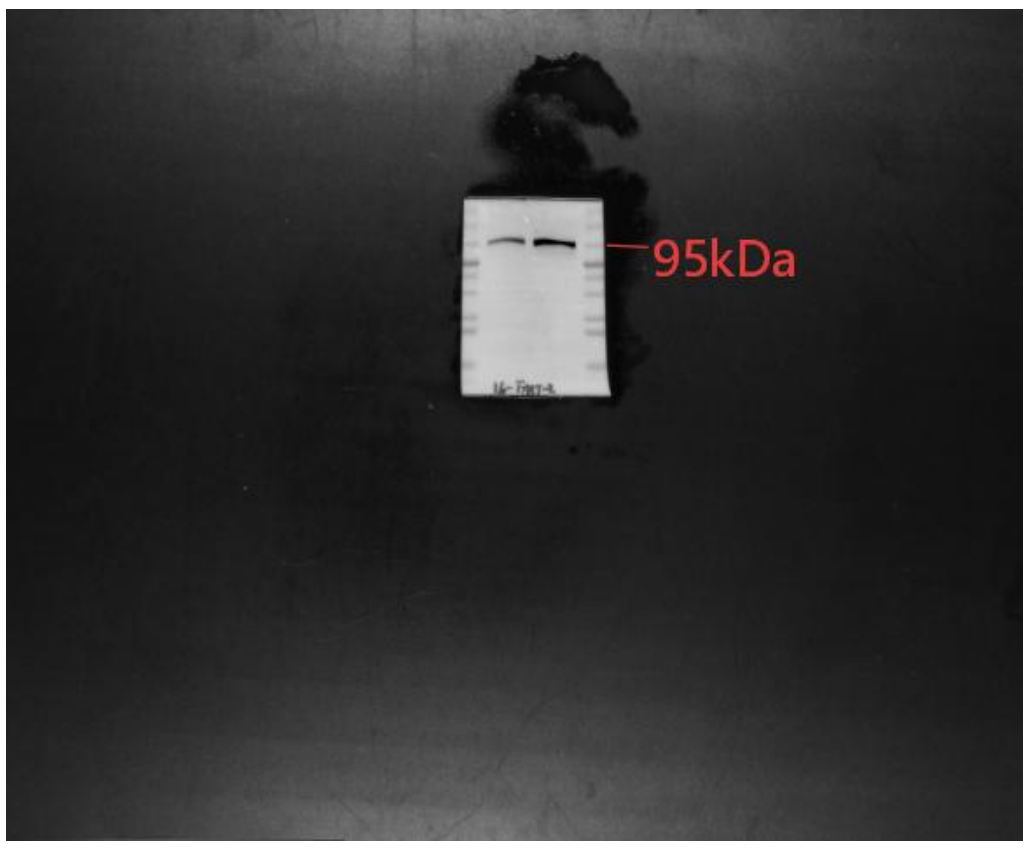

N-Cadherin

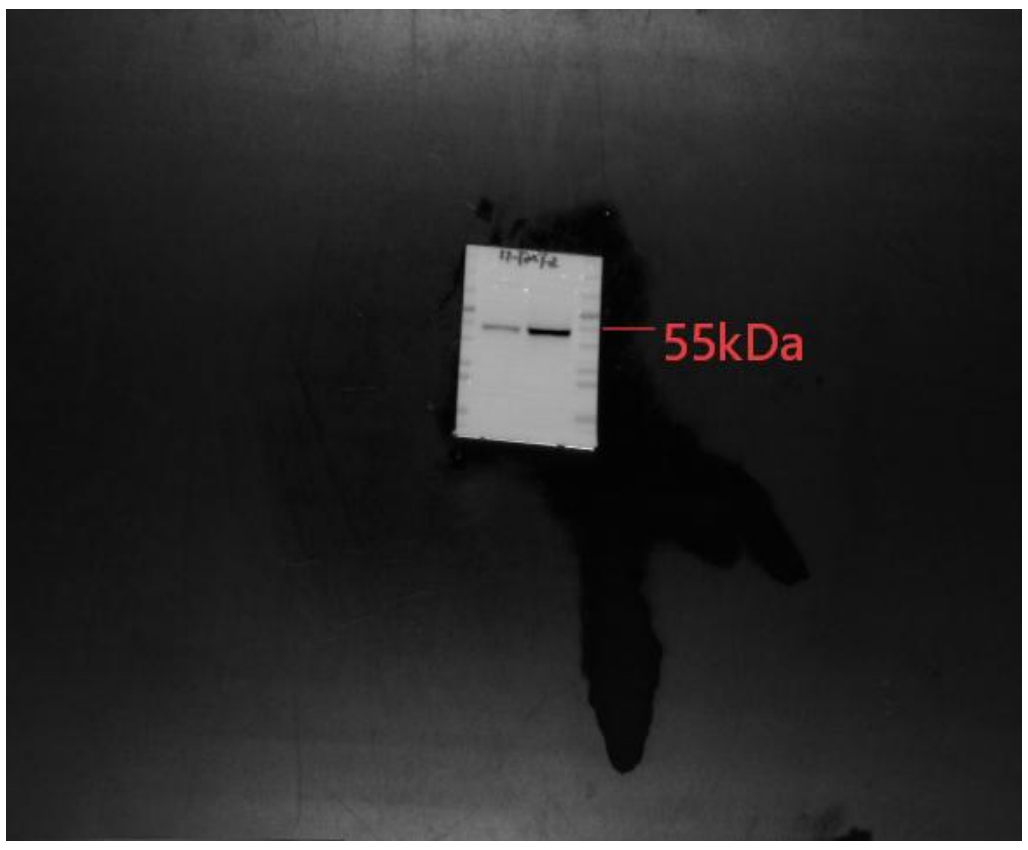

Vimentin

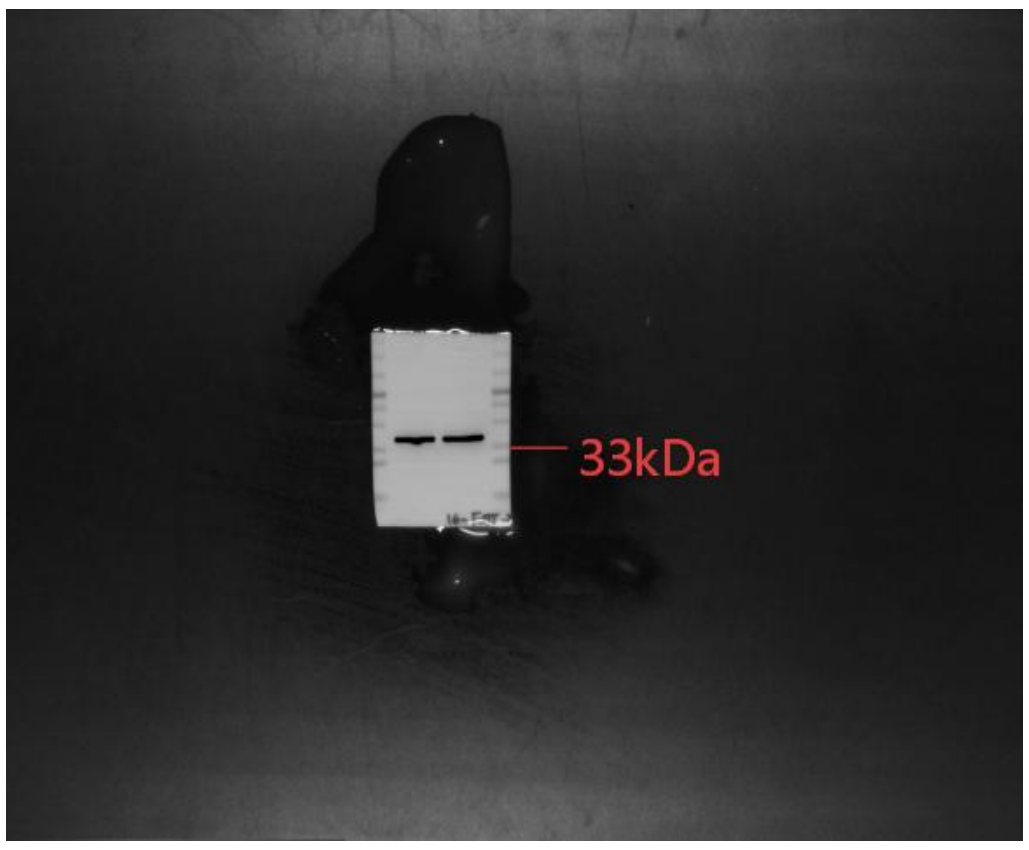

GAPDH

Figure 4F

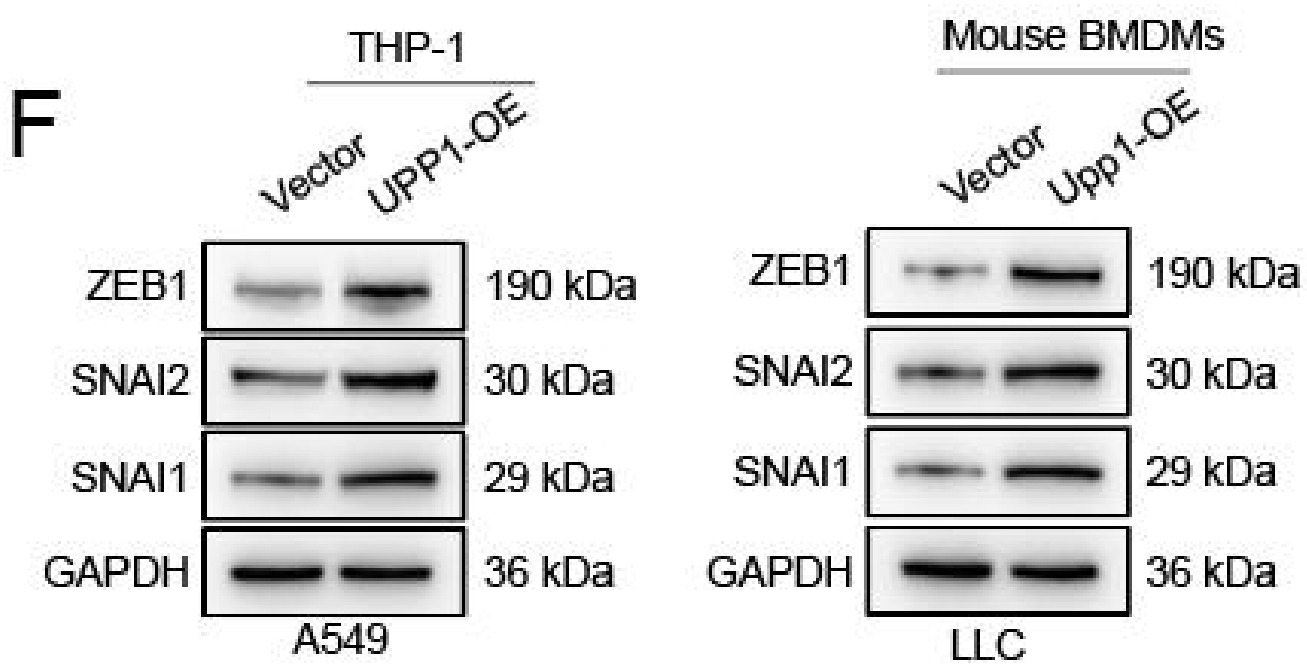

A549

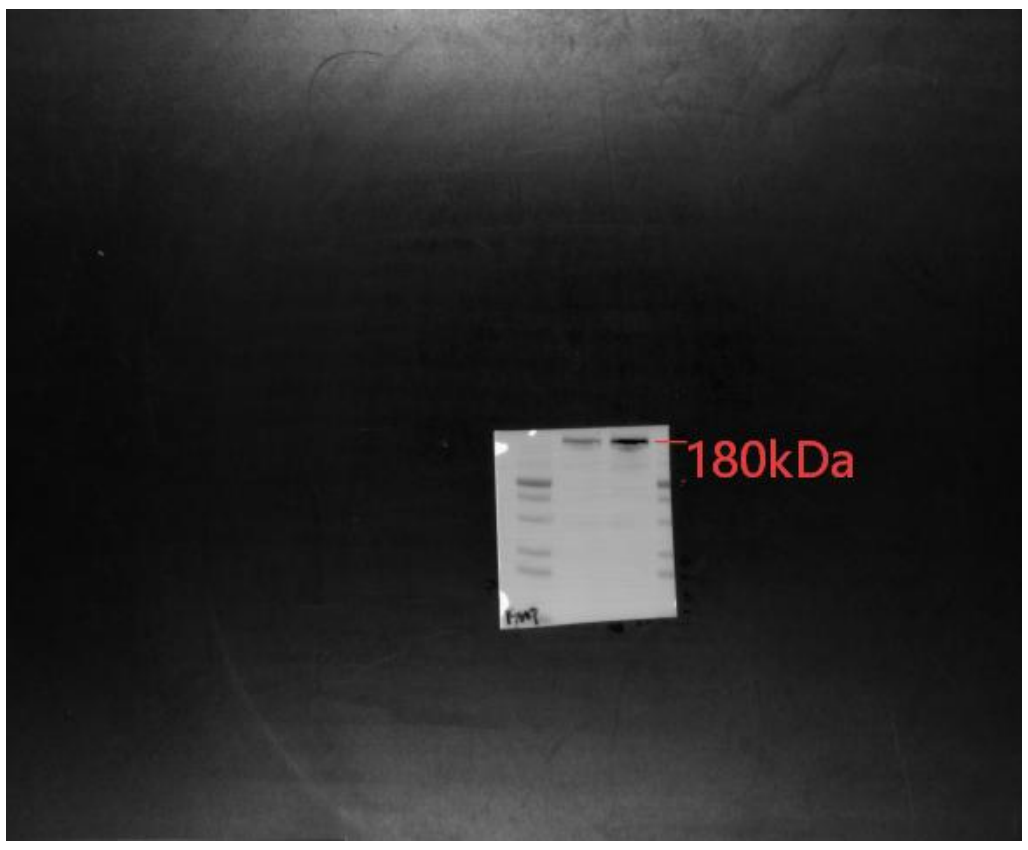

ZEB1

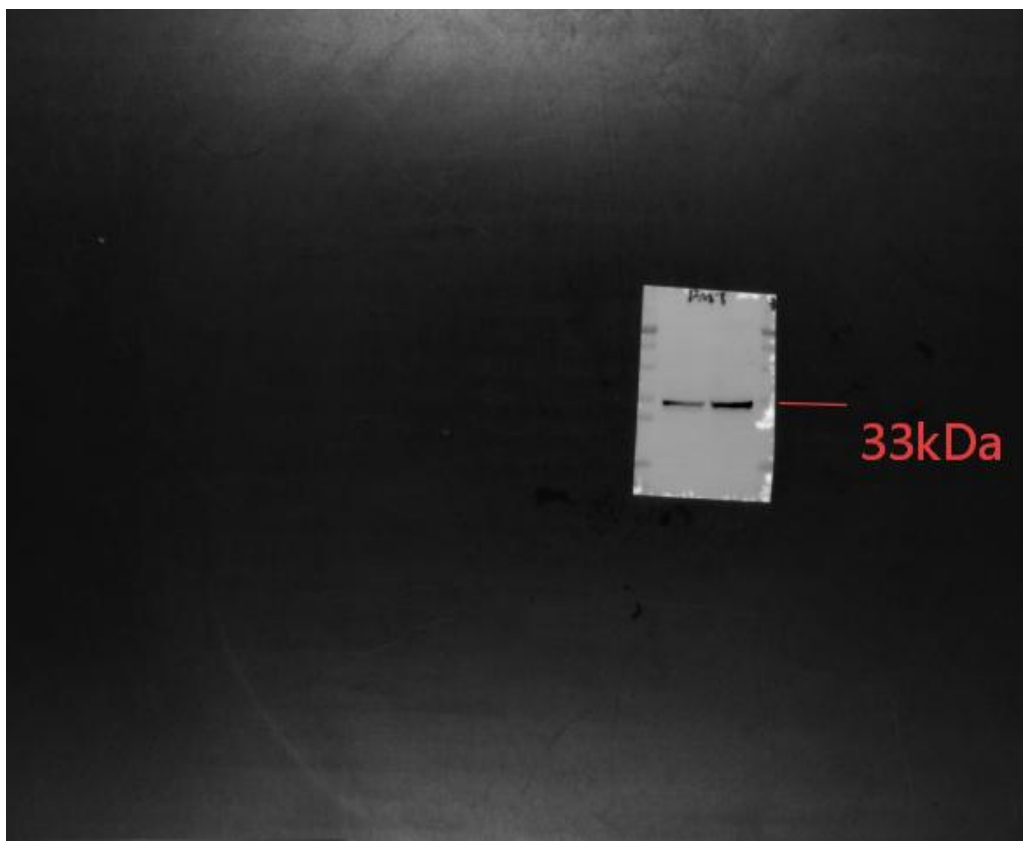

SNAI2

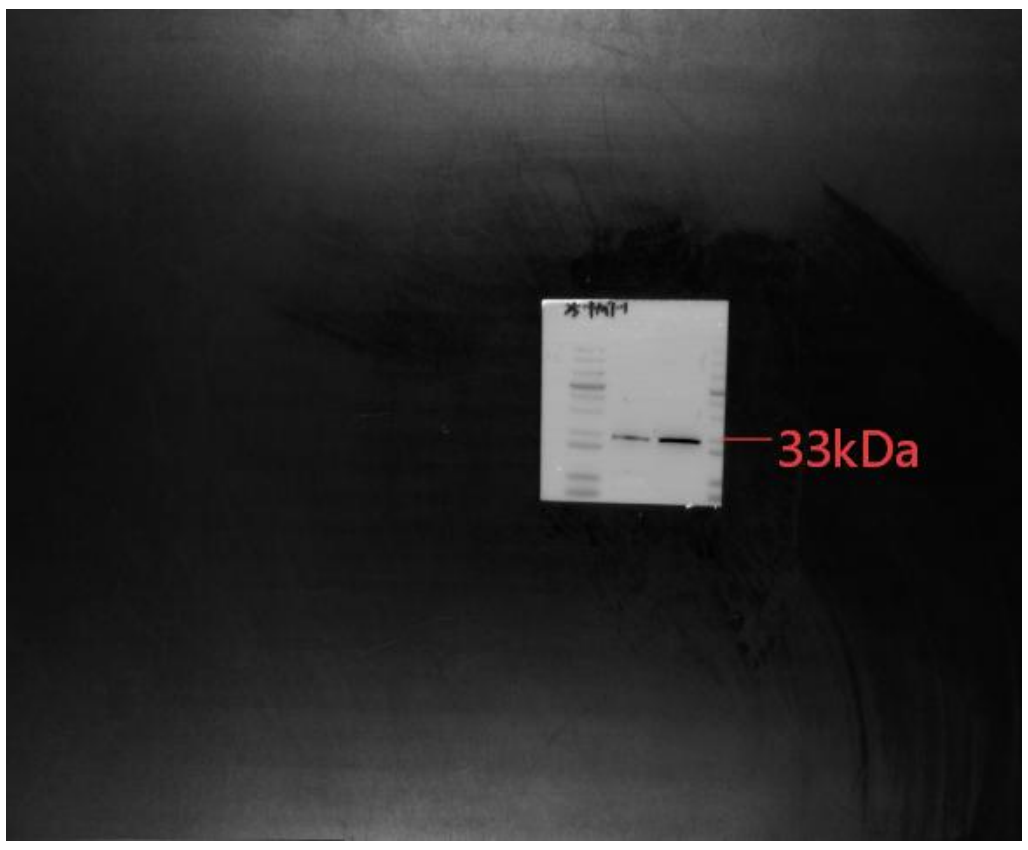

SNAI1

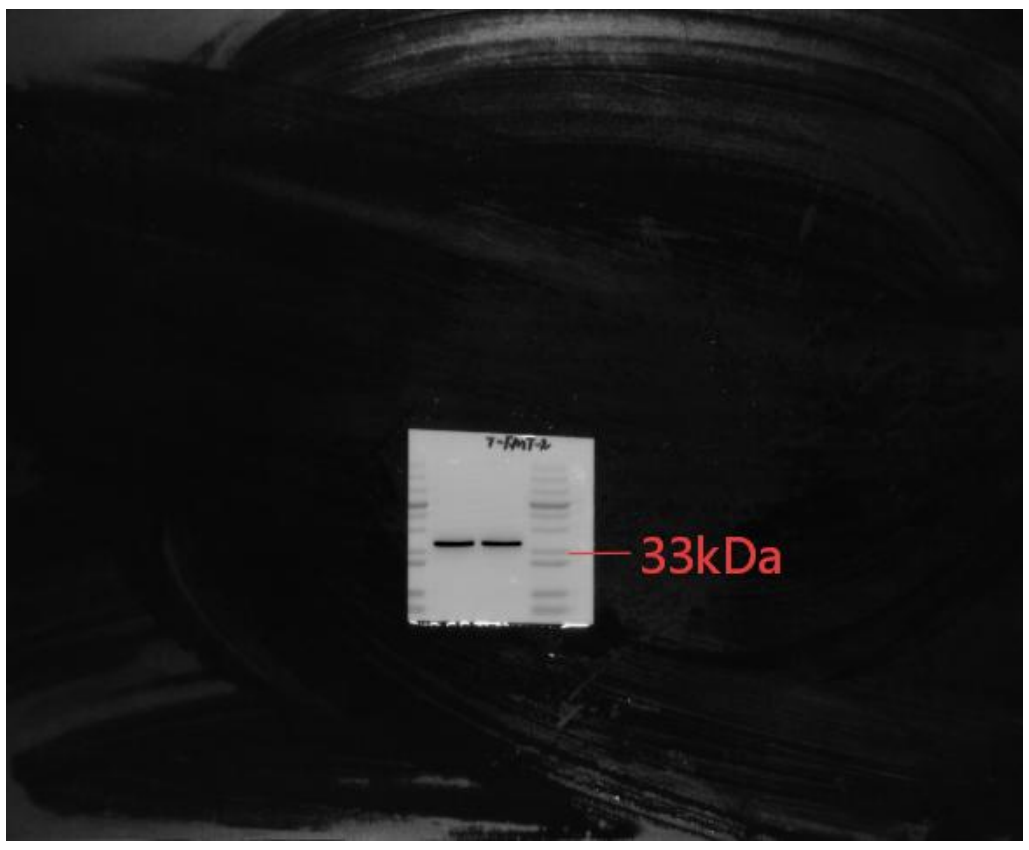

GAPDH

LLC

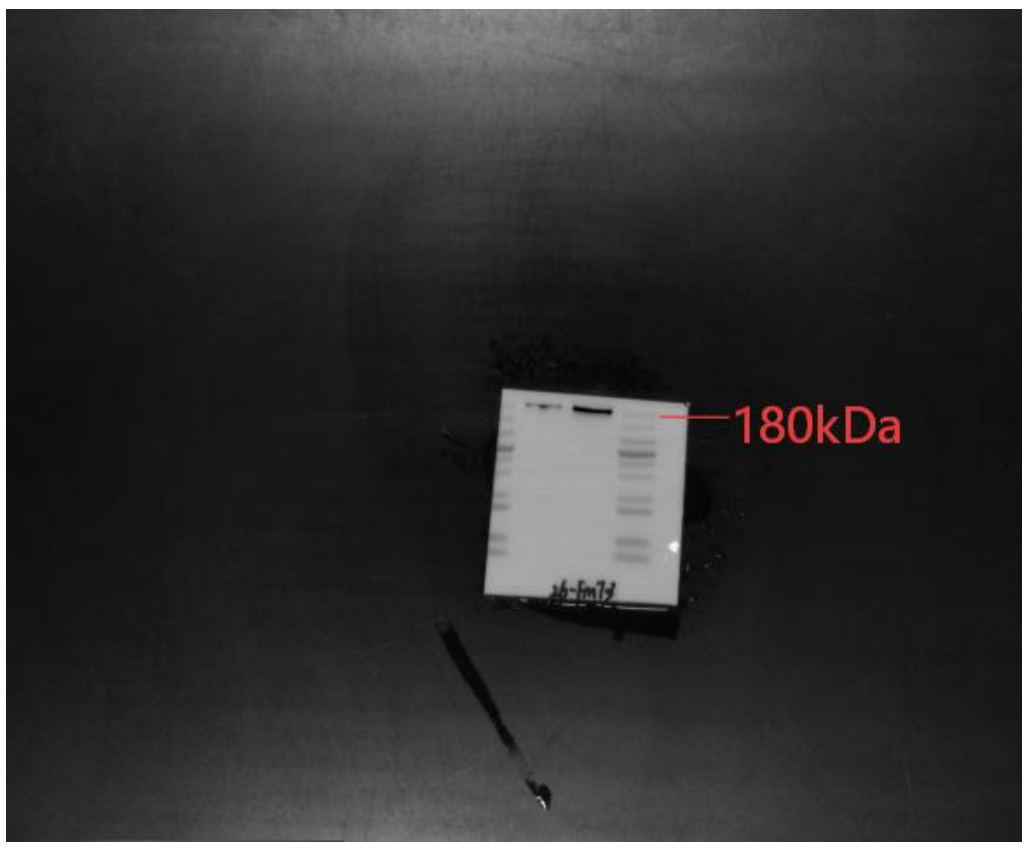

ZEB1

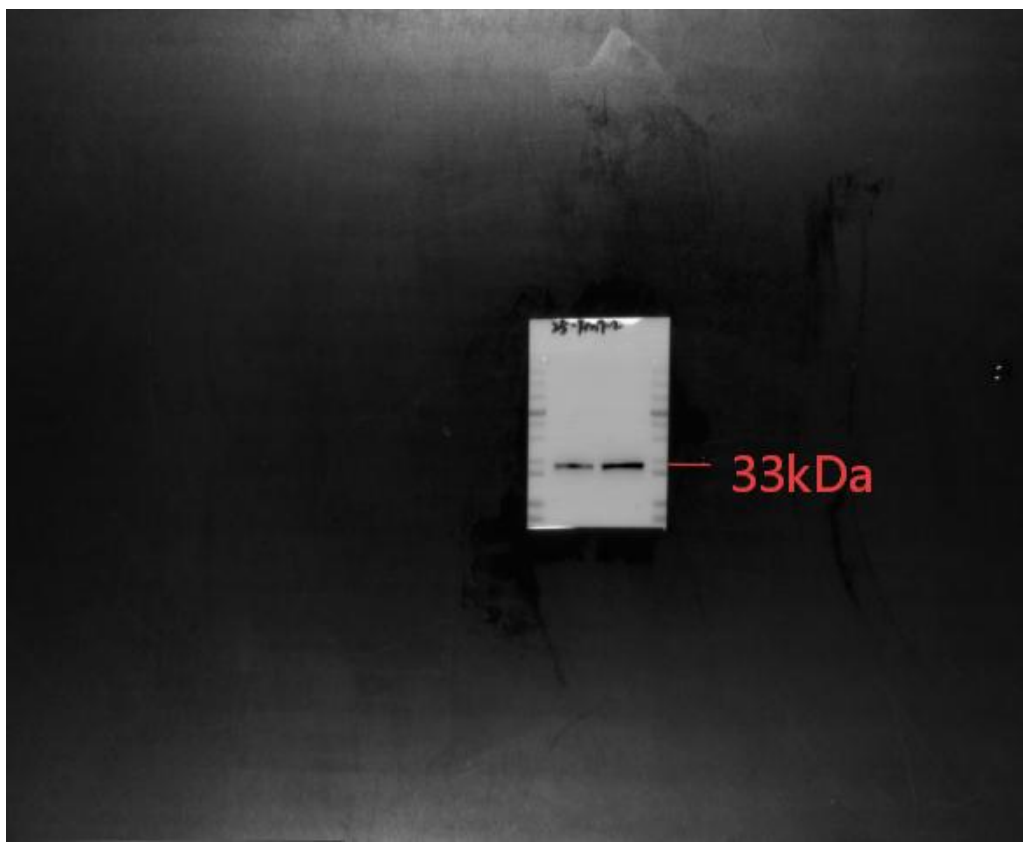

SNAI2

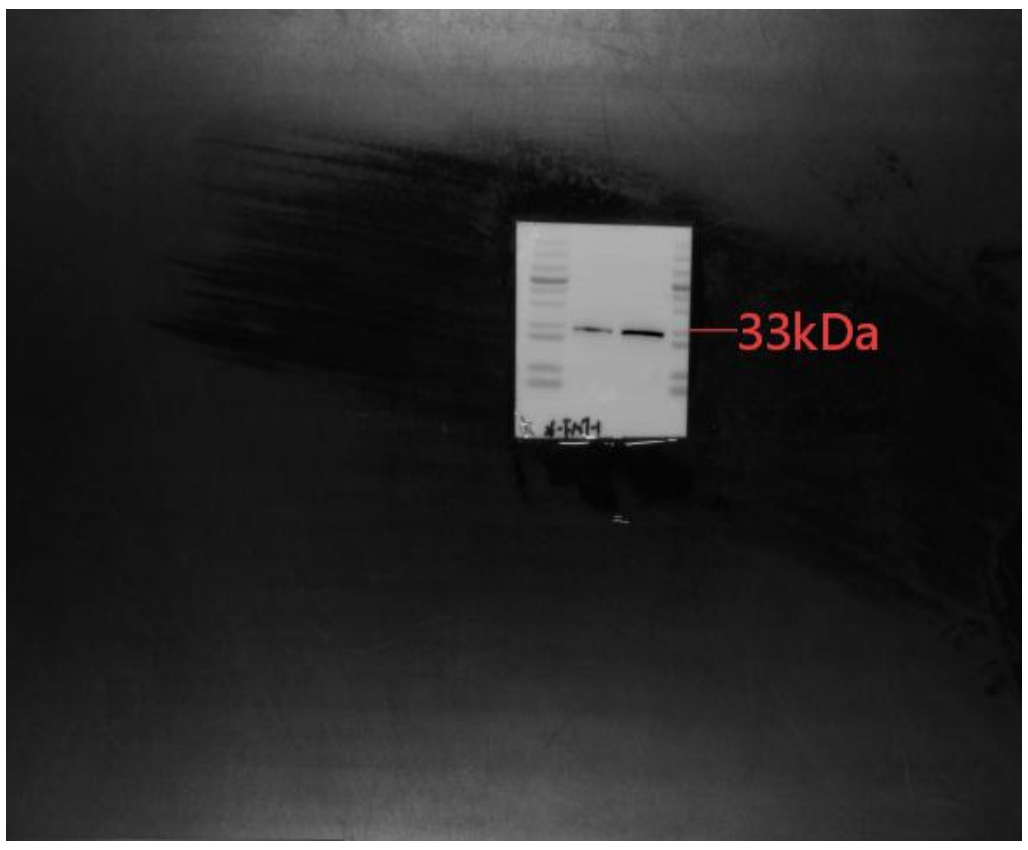

SNAI1

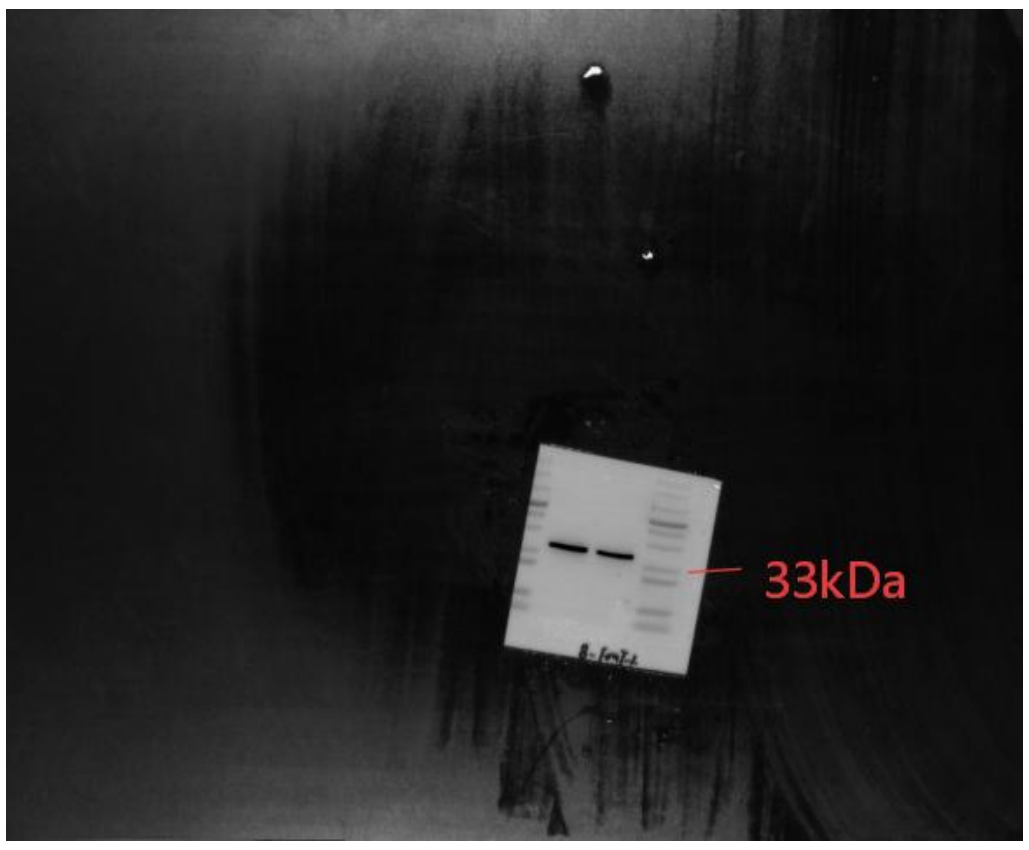

GAPDH

Figure 5E

E

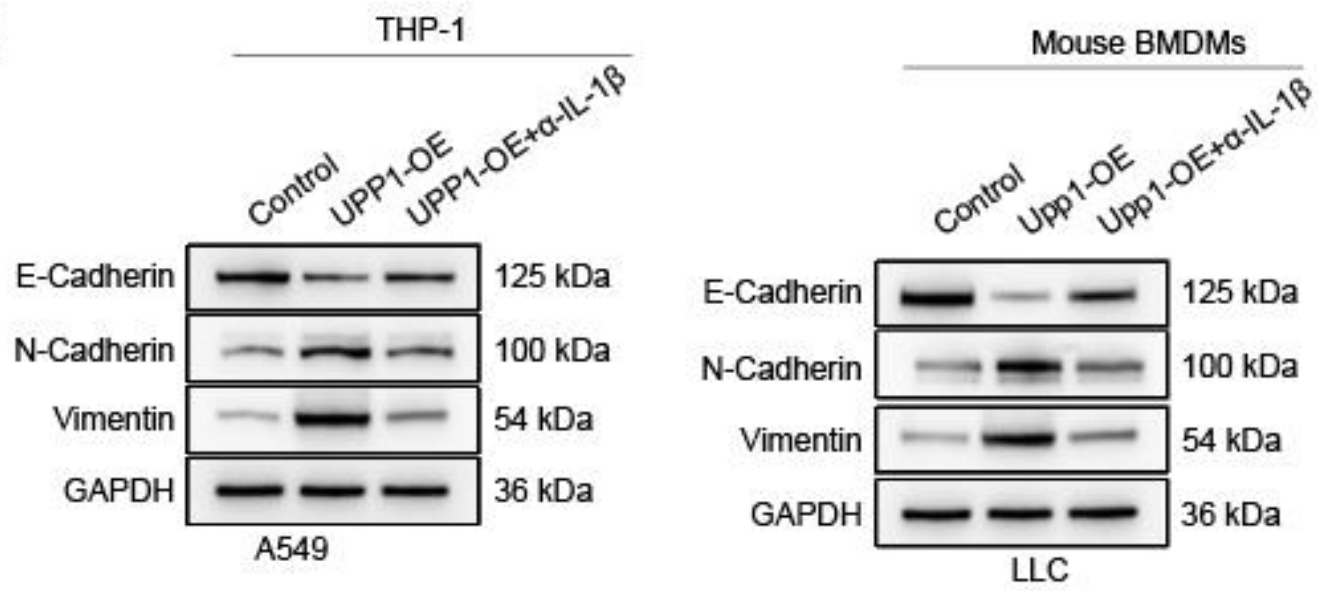

A549

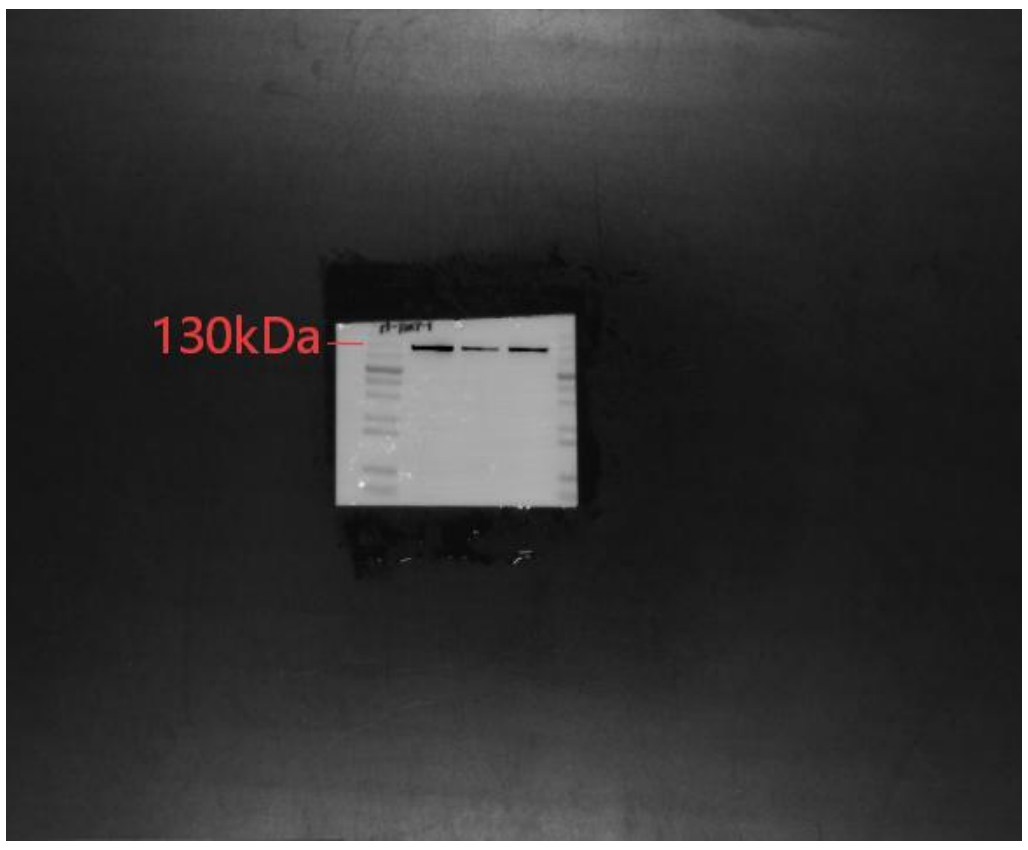

E-Cadherin

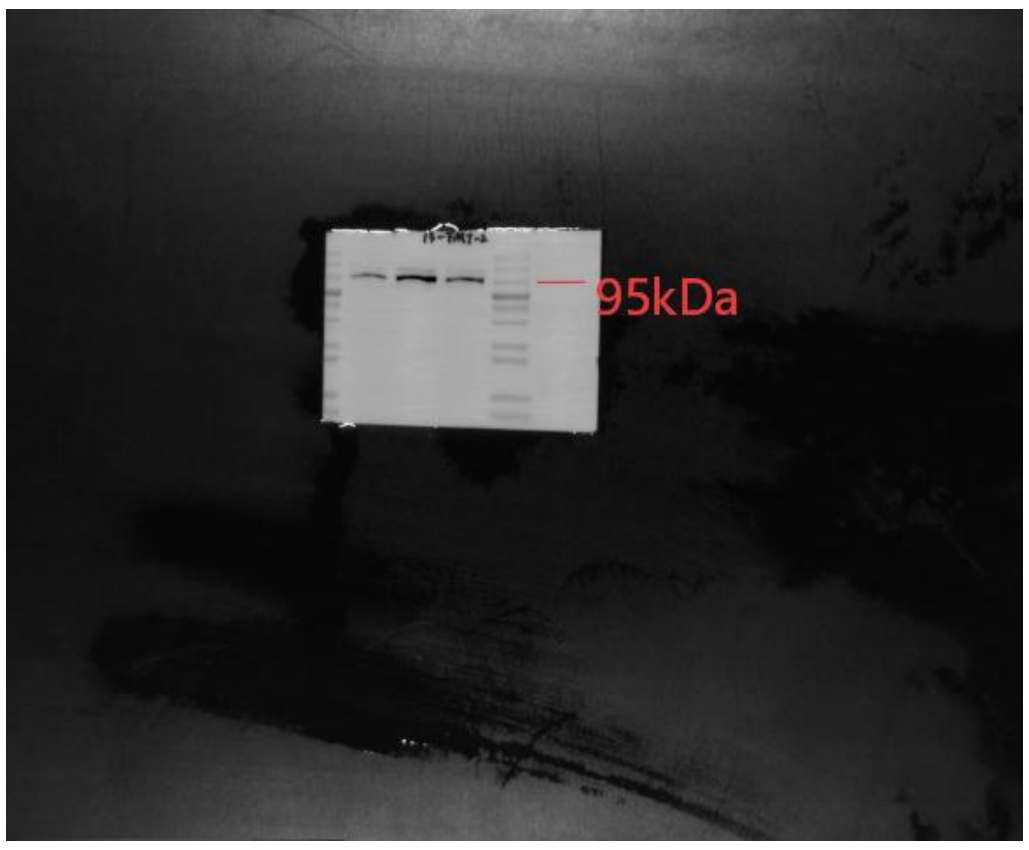

N-Cadherin

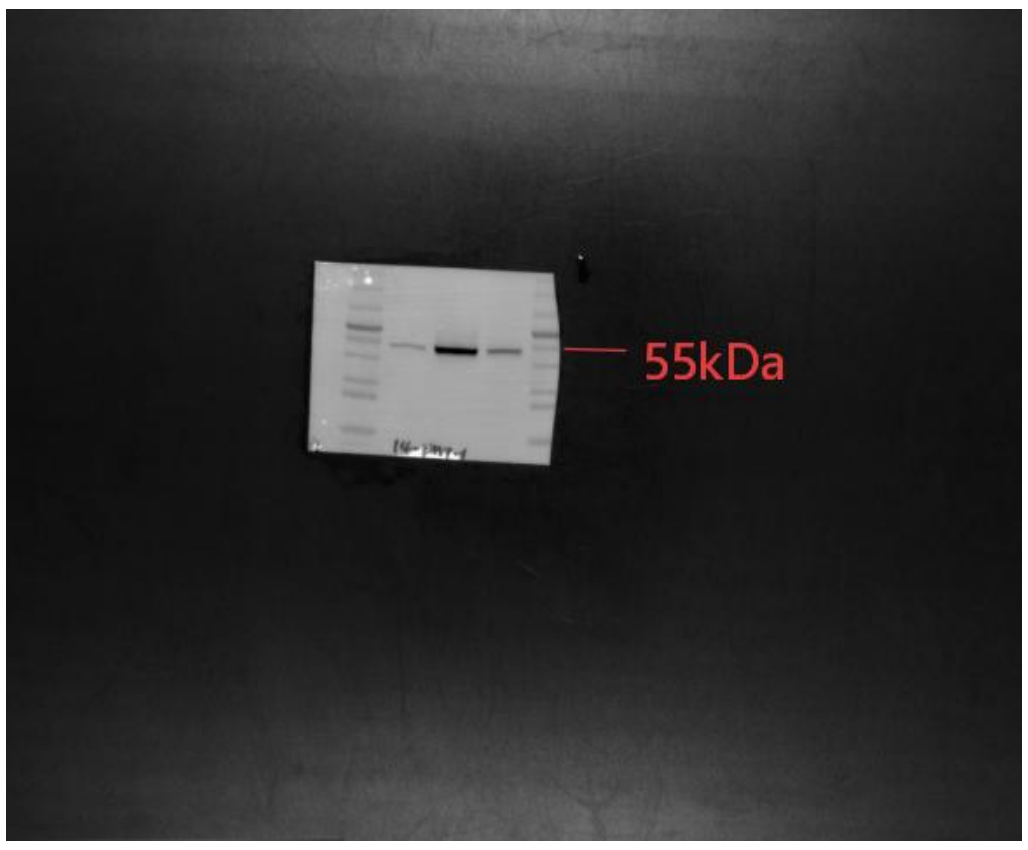

Vimentin

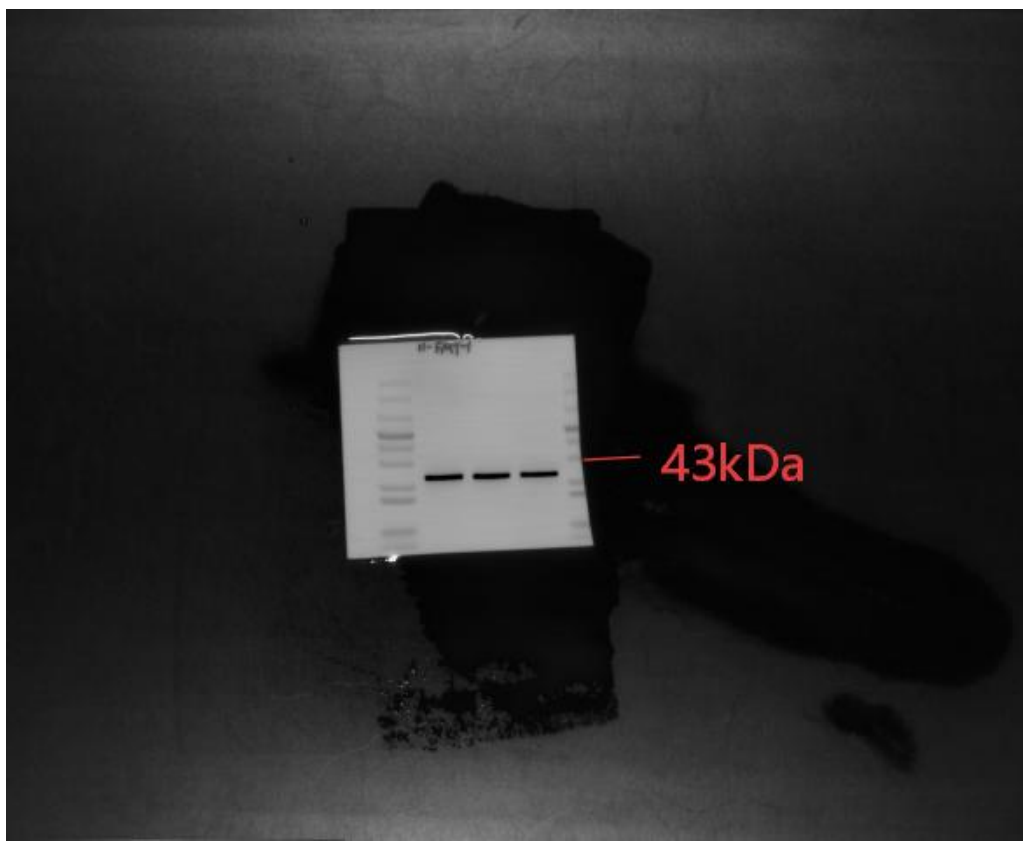

GAPDH

LLC

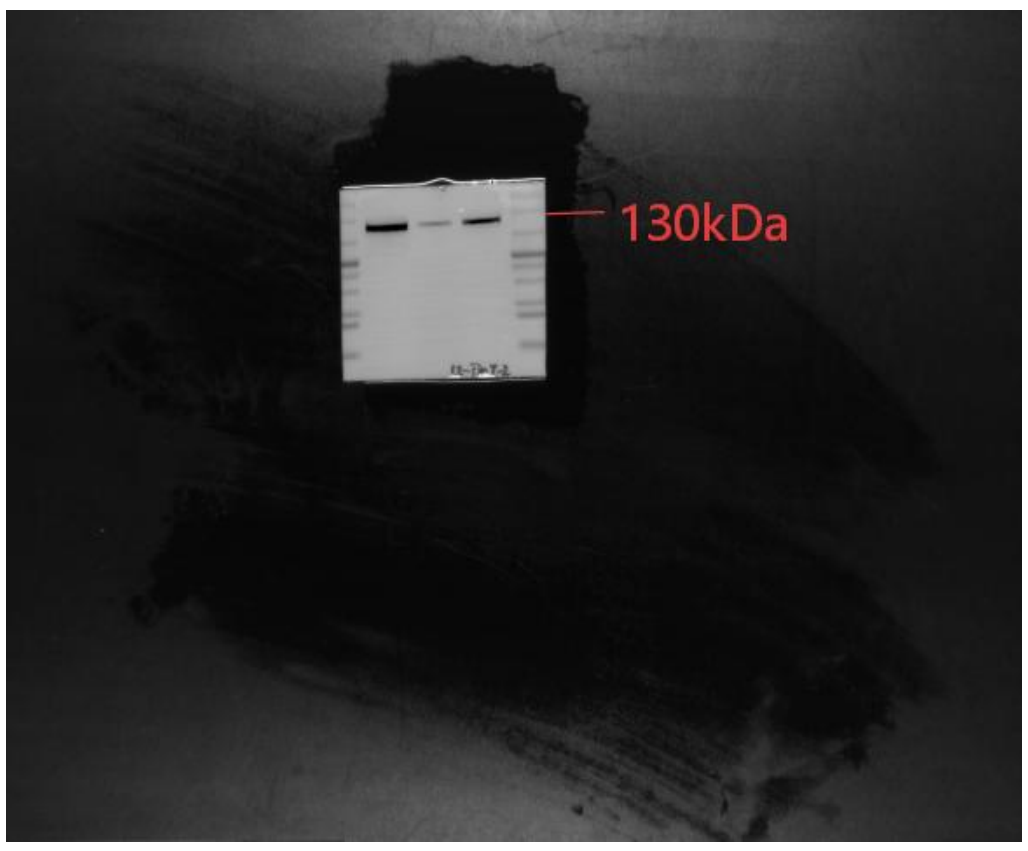

E-Cadherin

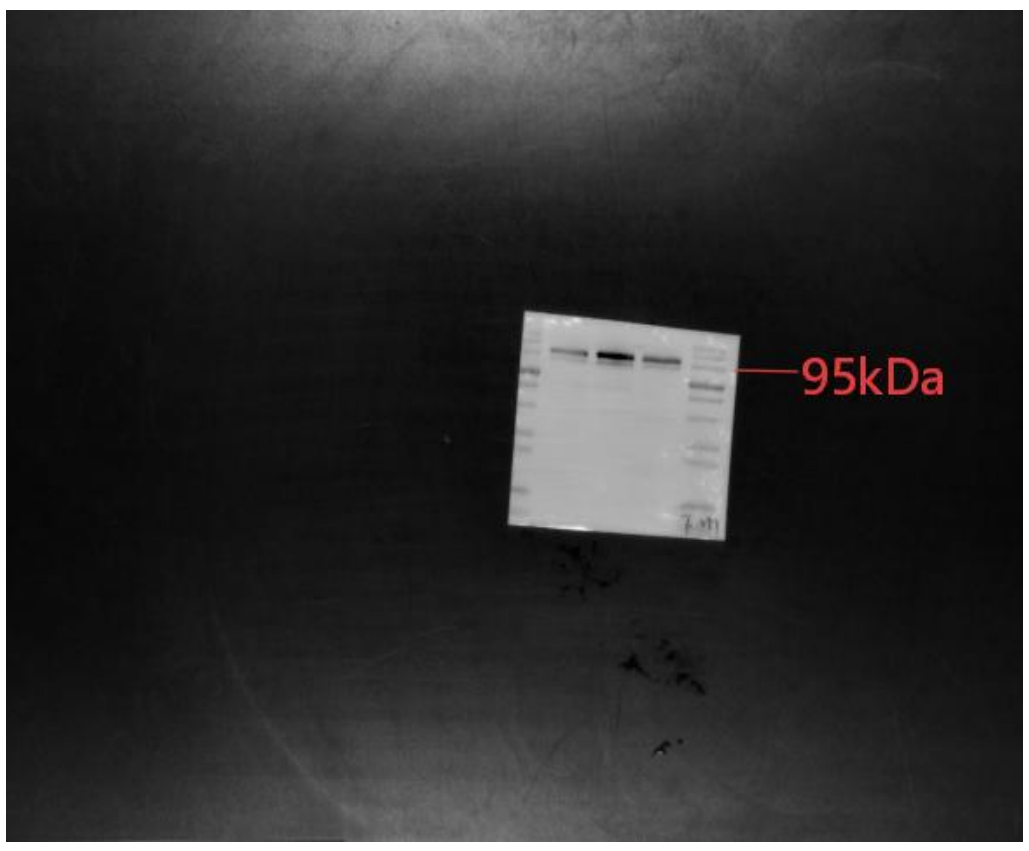

N-Cadherin

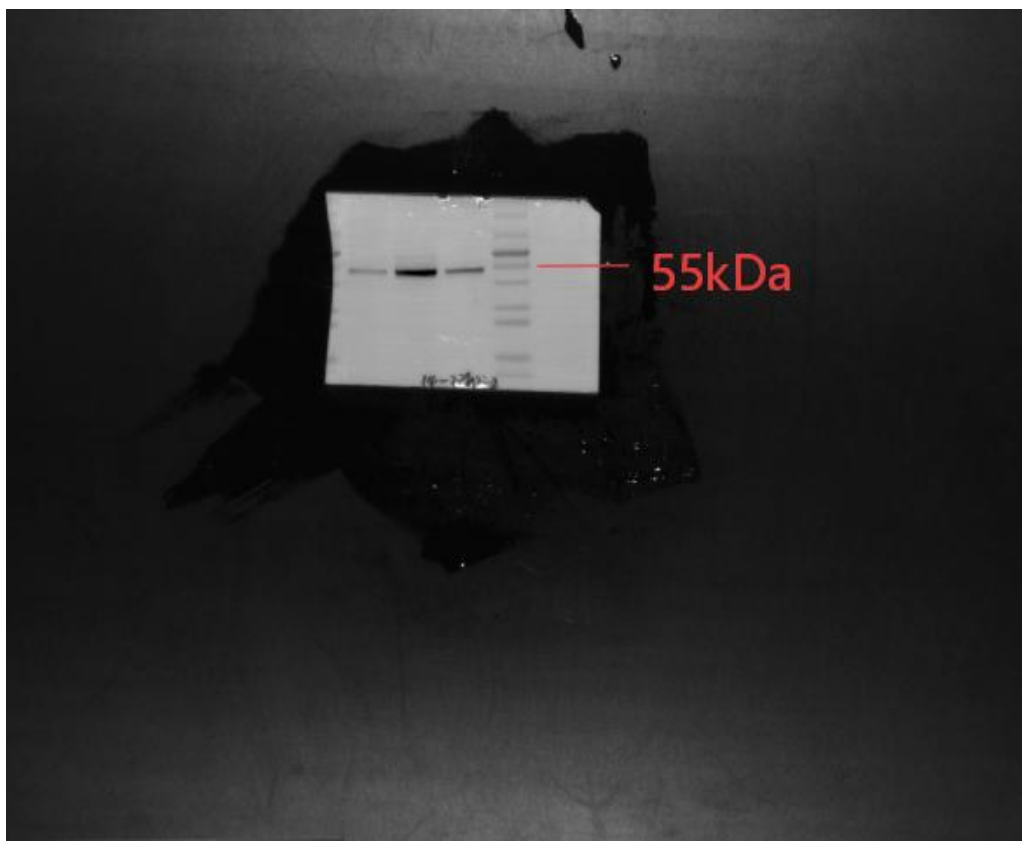

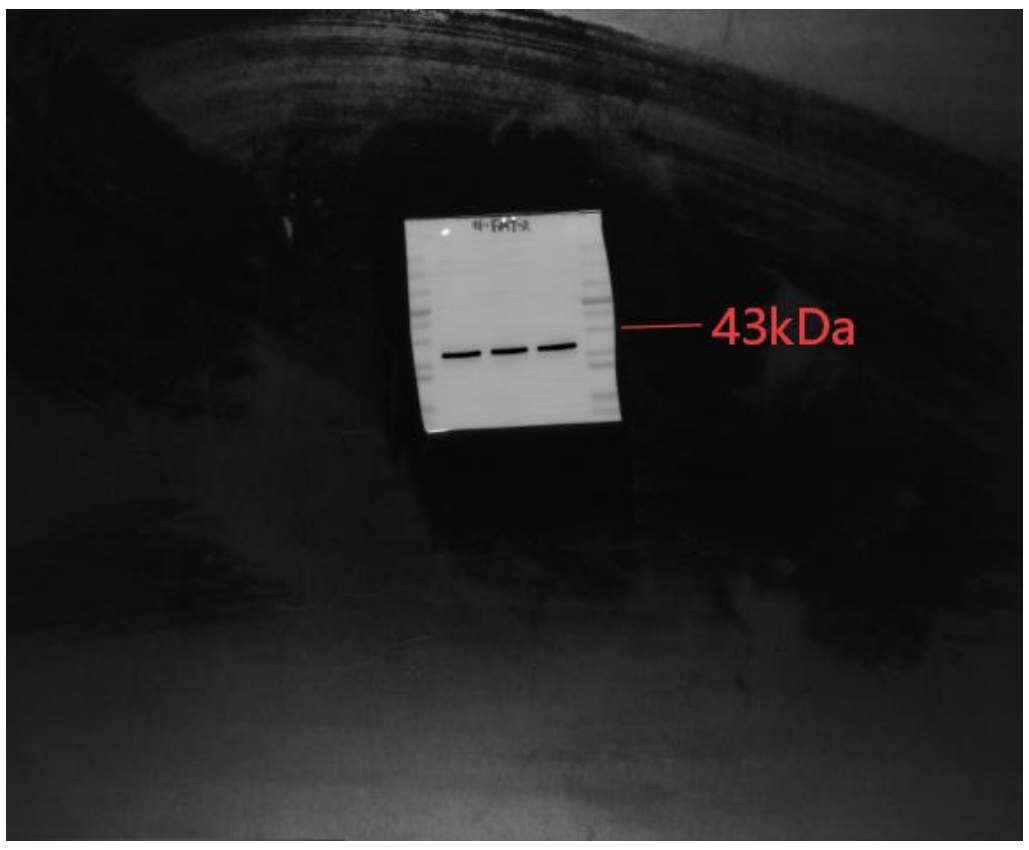

Vimentin GAPDH

Figure 5F

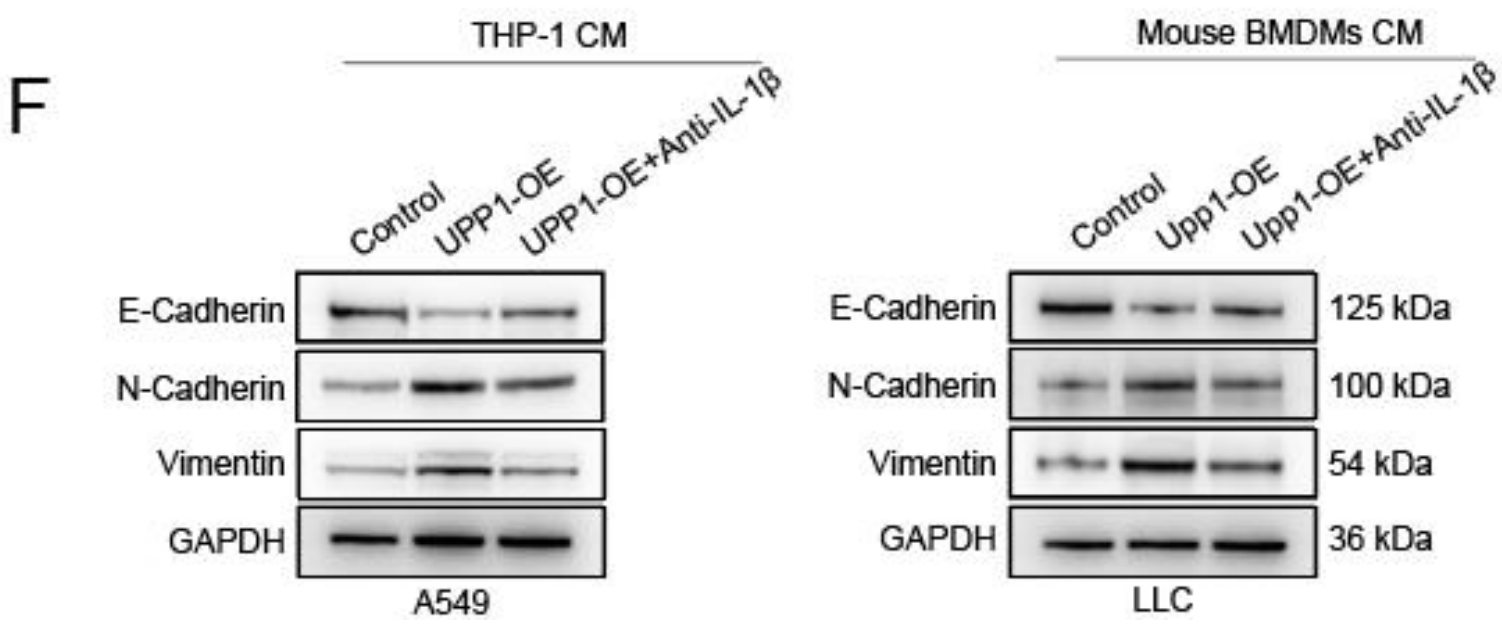

A549

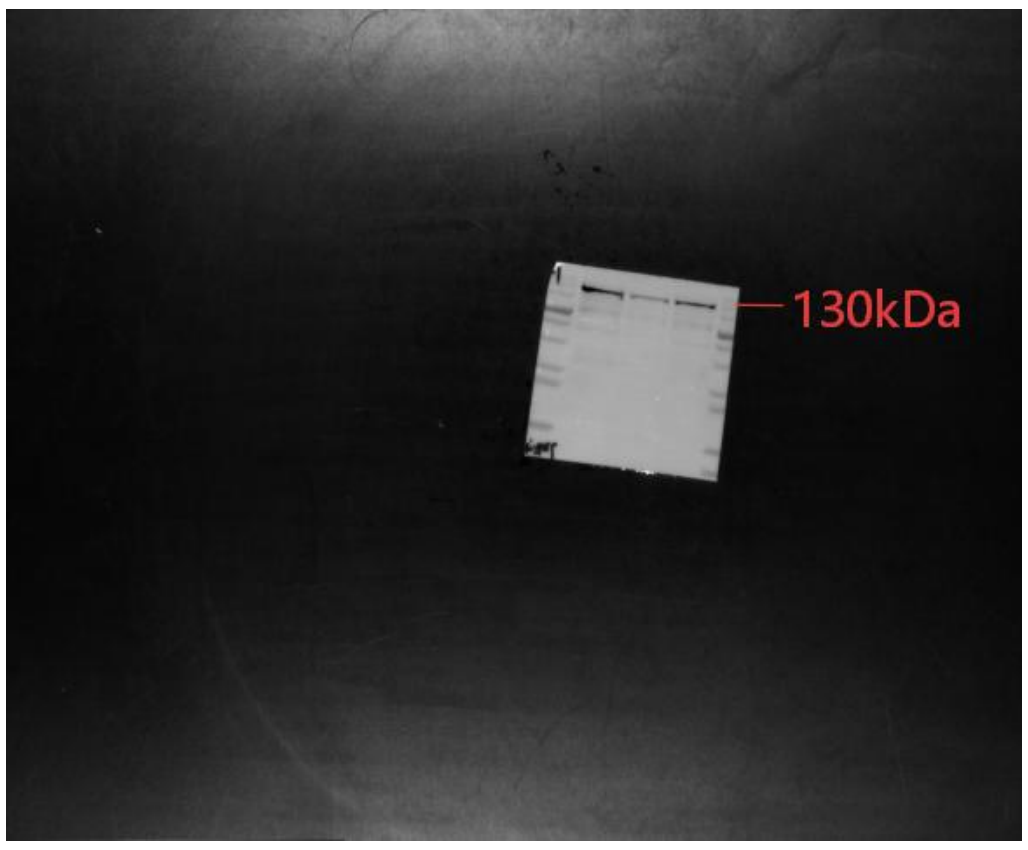

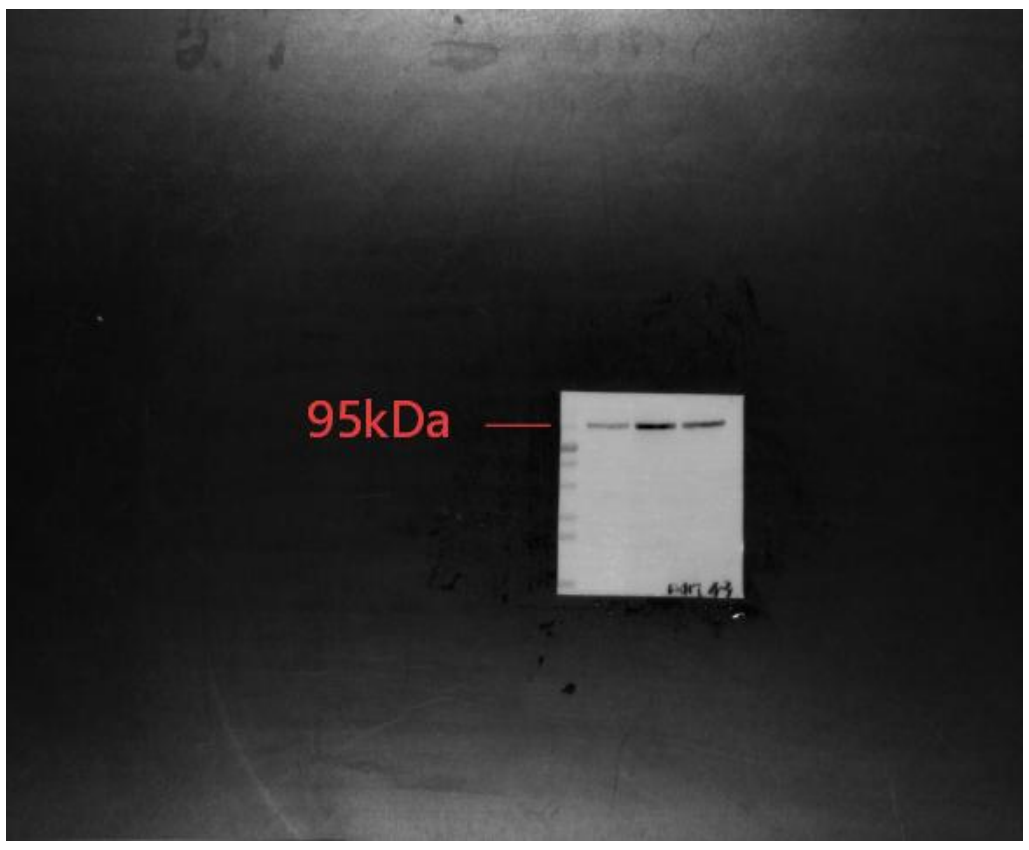

E-Cadherin N-Cadherin

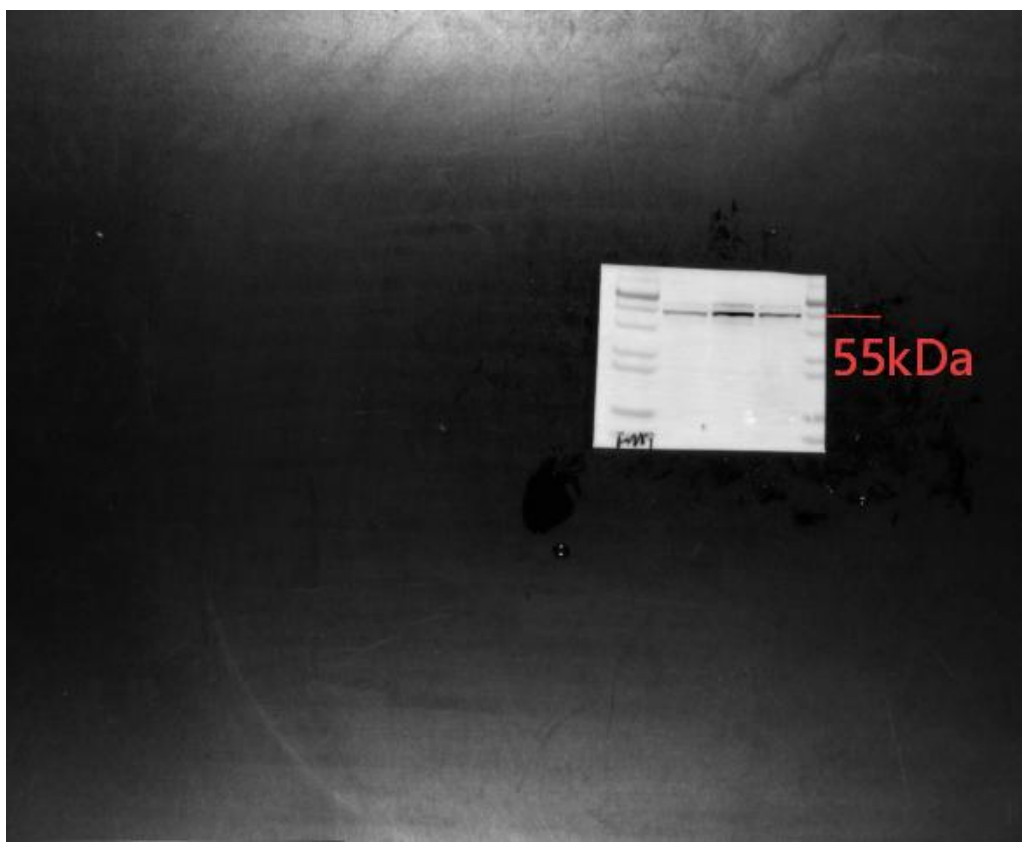

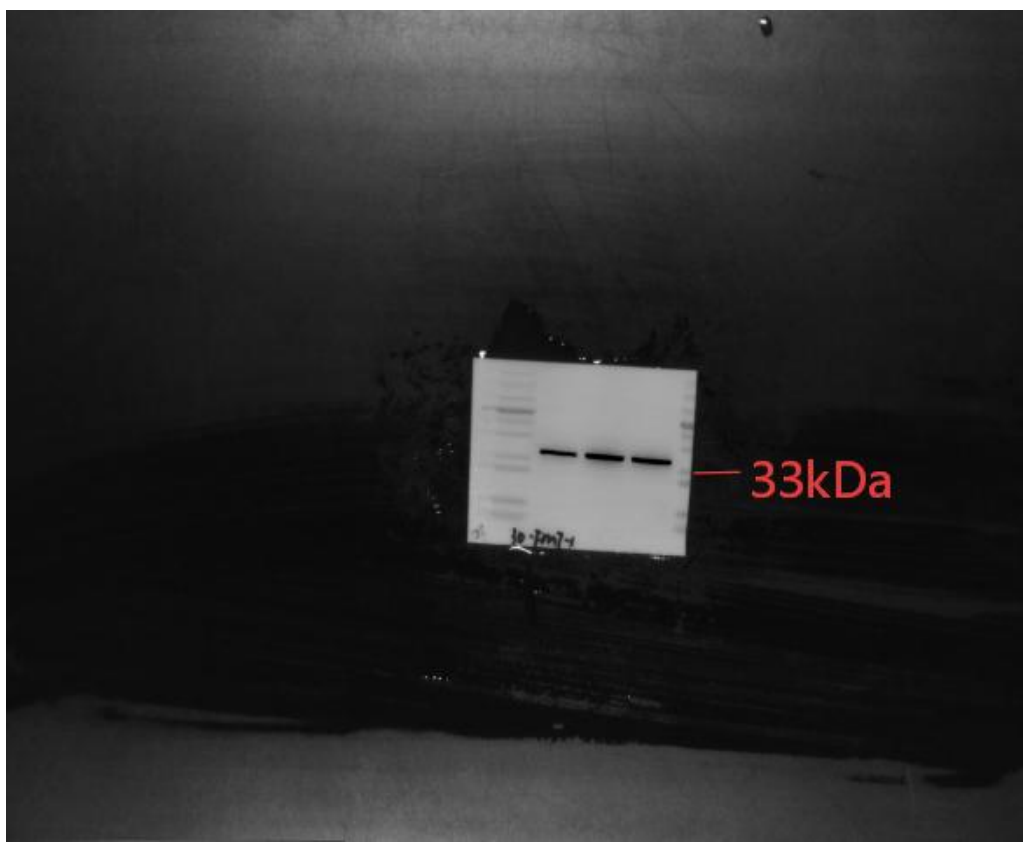

Vimentin GAPDH

LLC

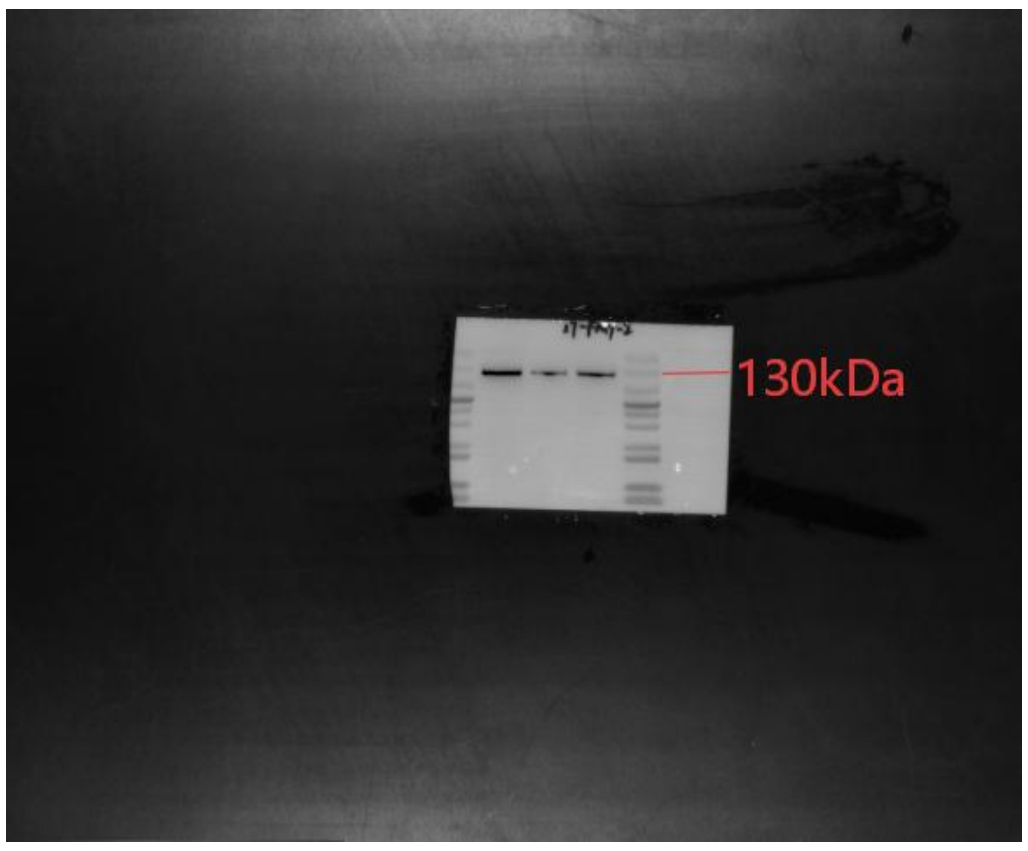

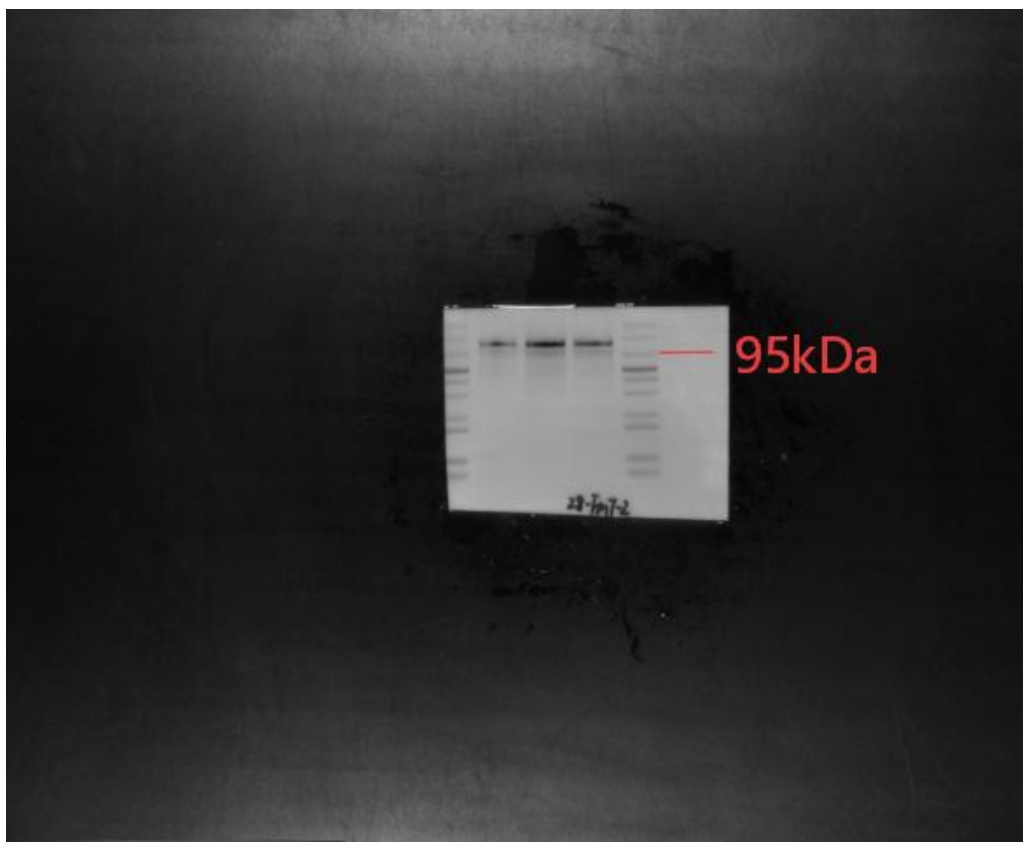

E-Cadherin N-Cadherin

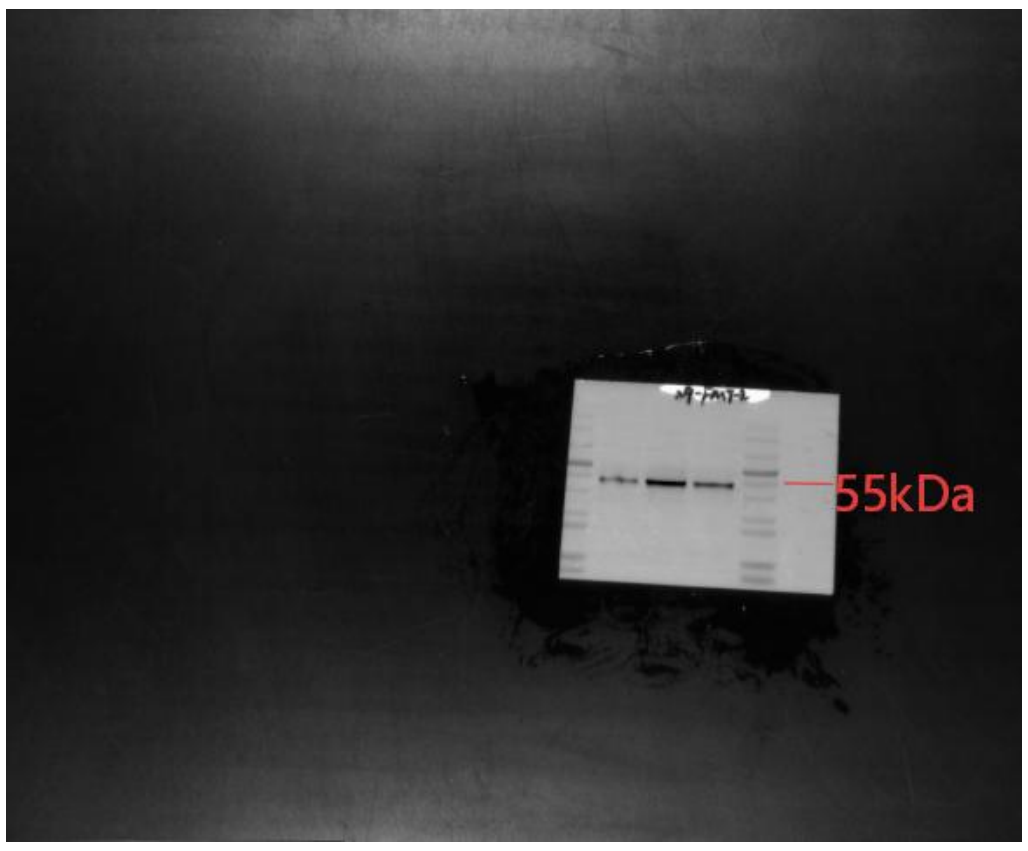

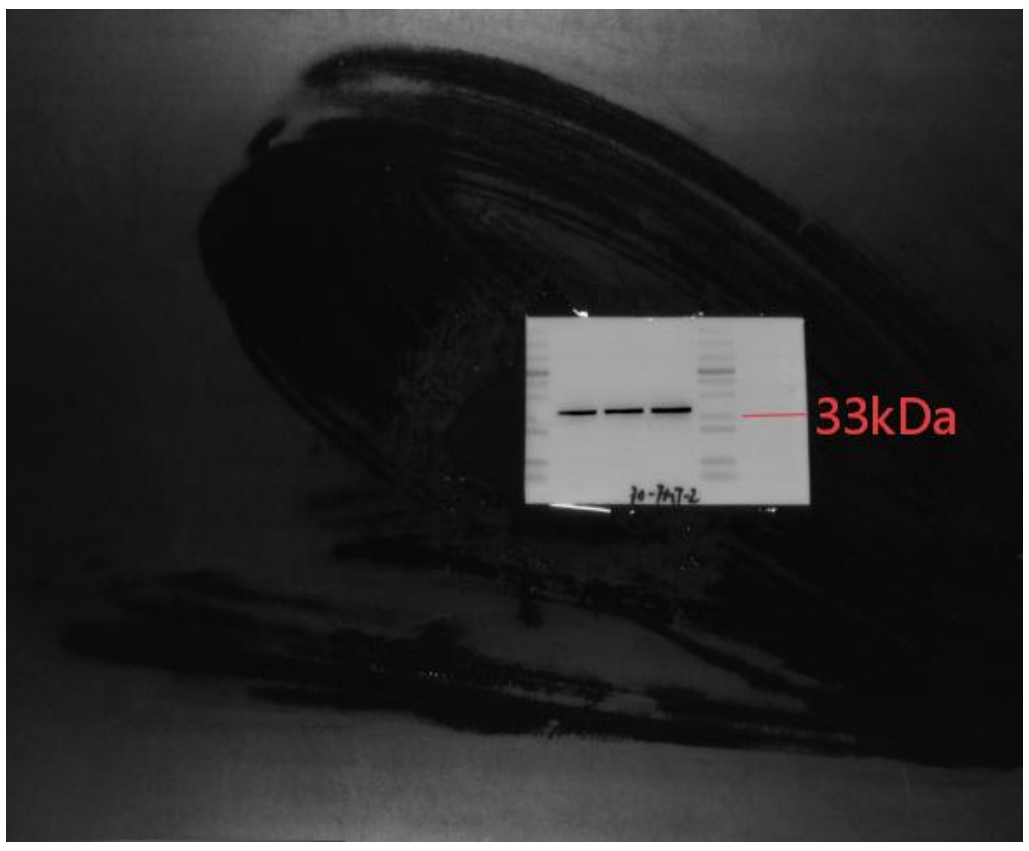

Vimentin GAPDH

Figure S2B

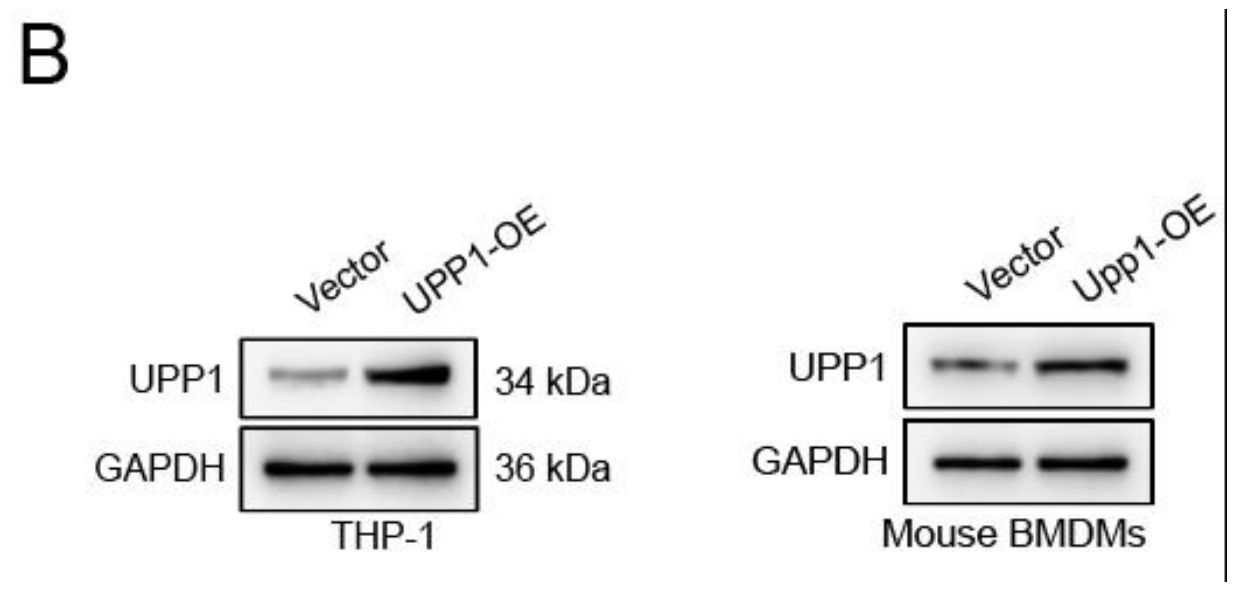

THP-1

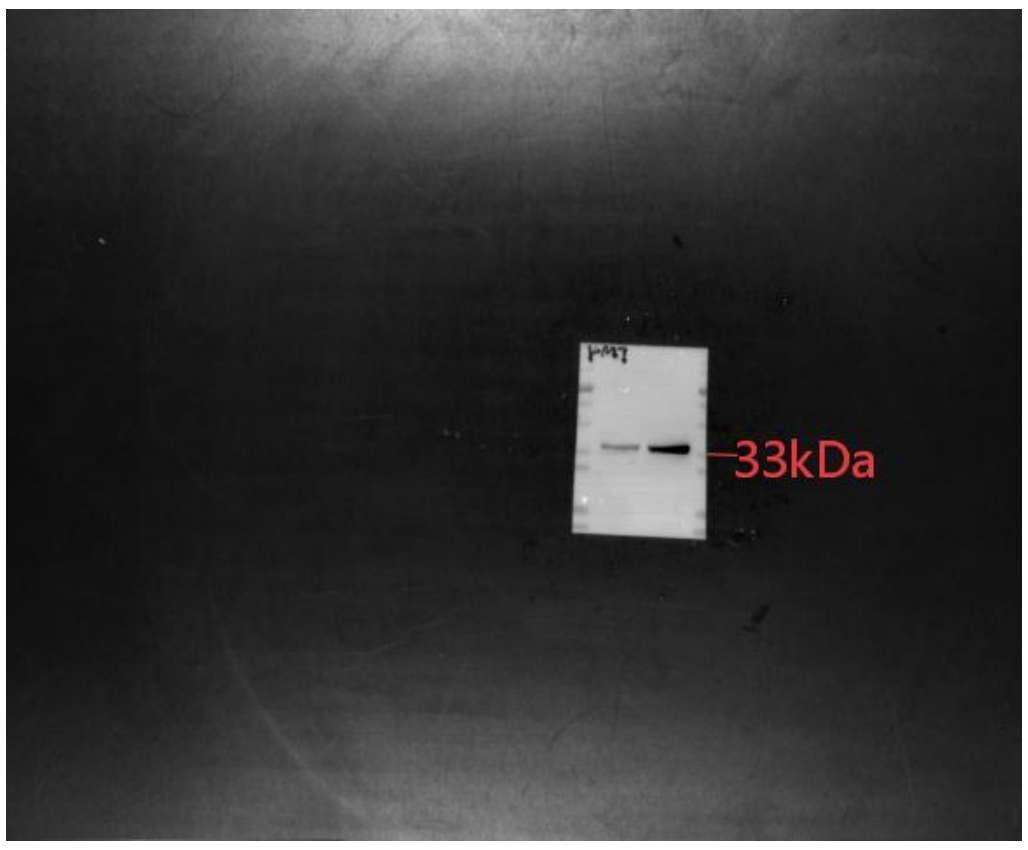

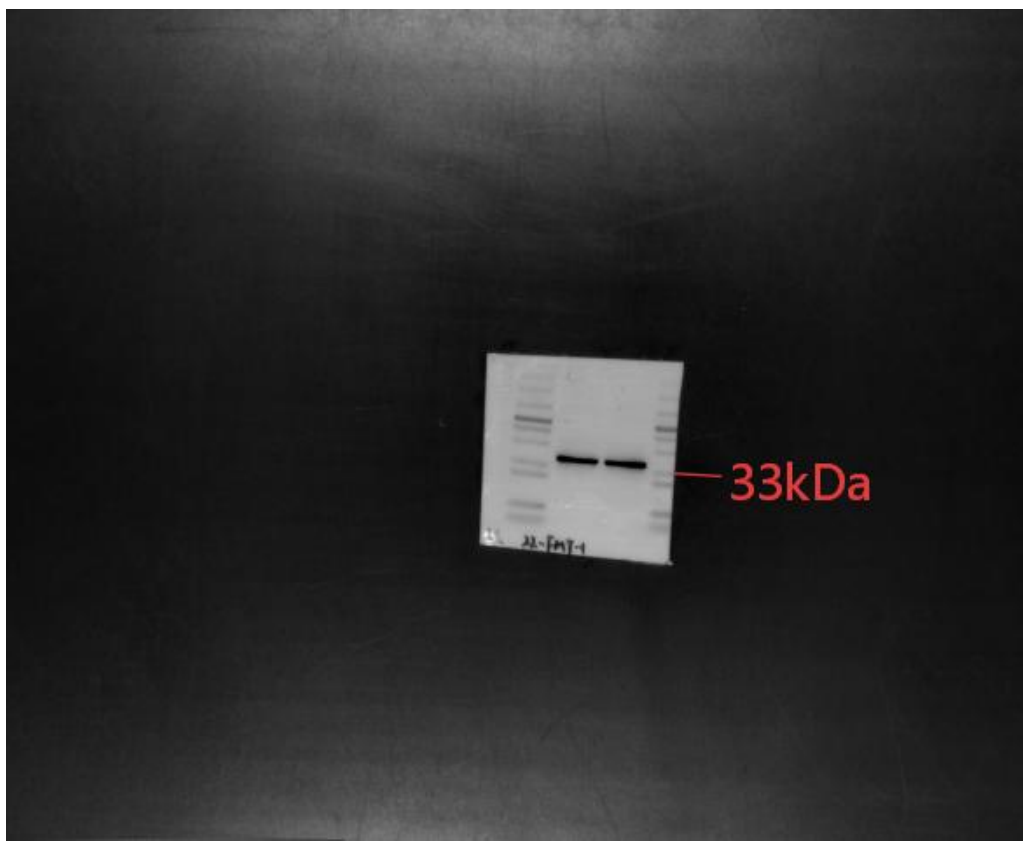

UPP1 GAPDH

Mouse BMDMs

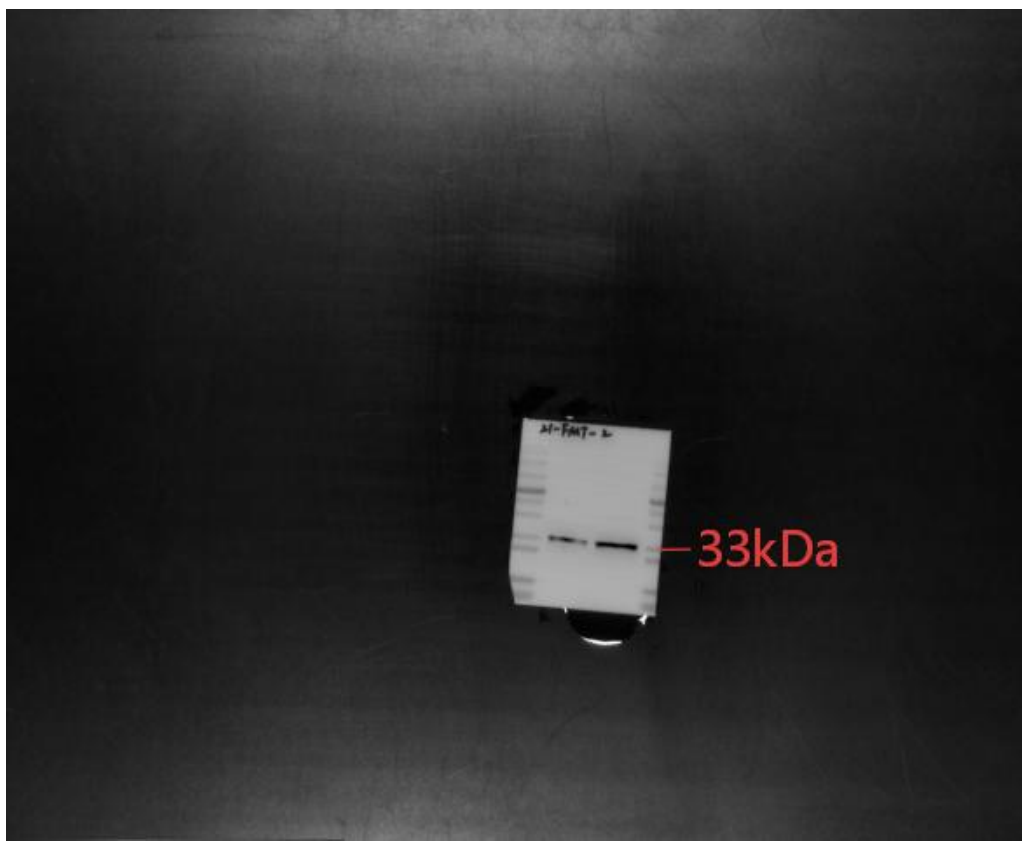

UPP1

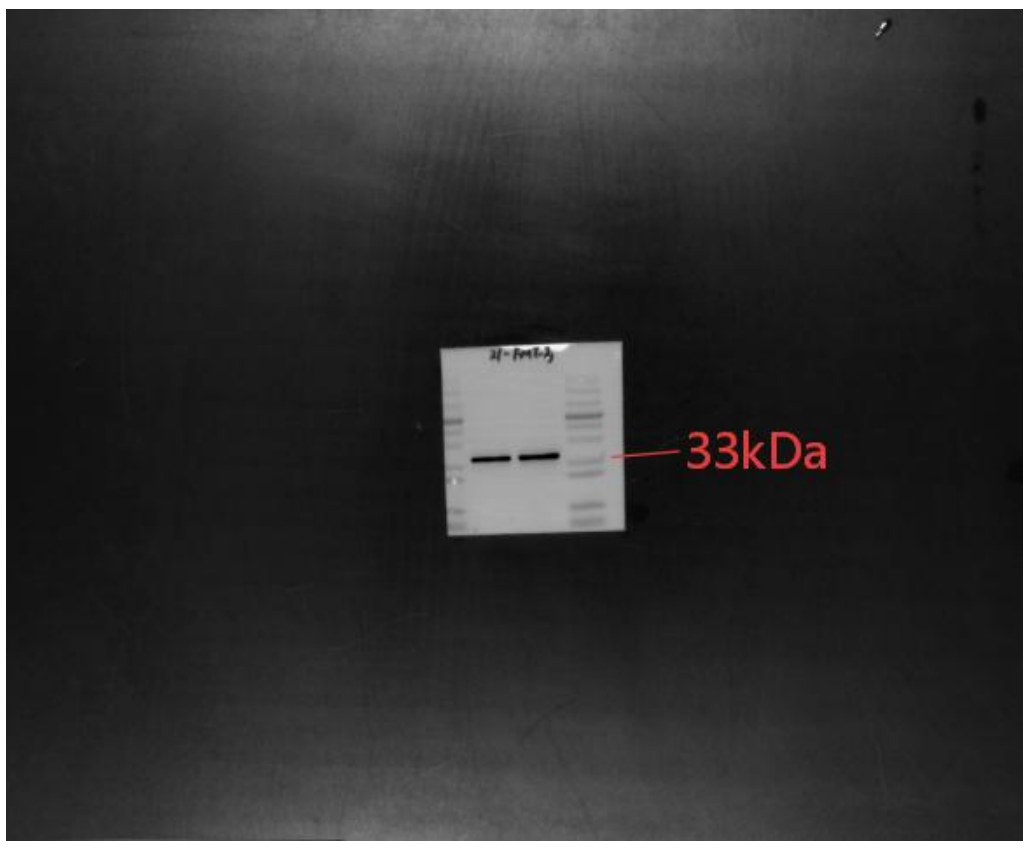

GAPDH

Figure S6

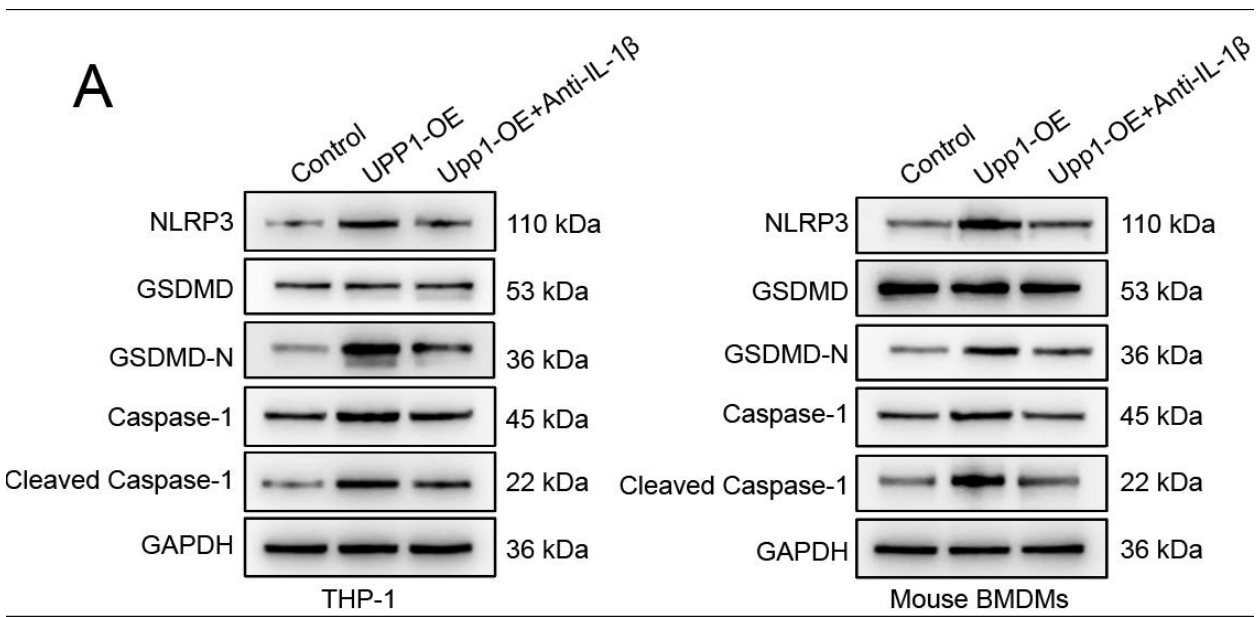

THP-1

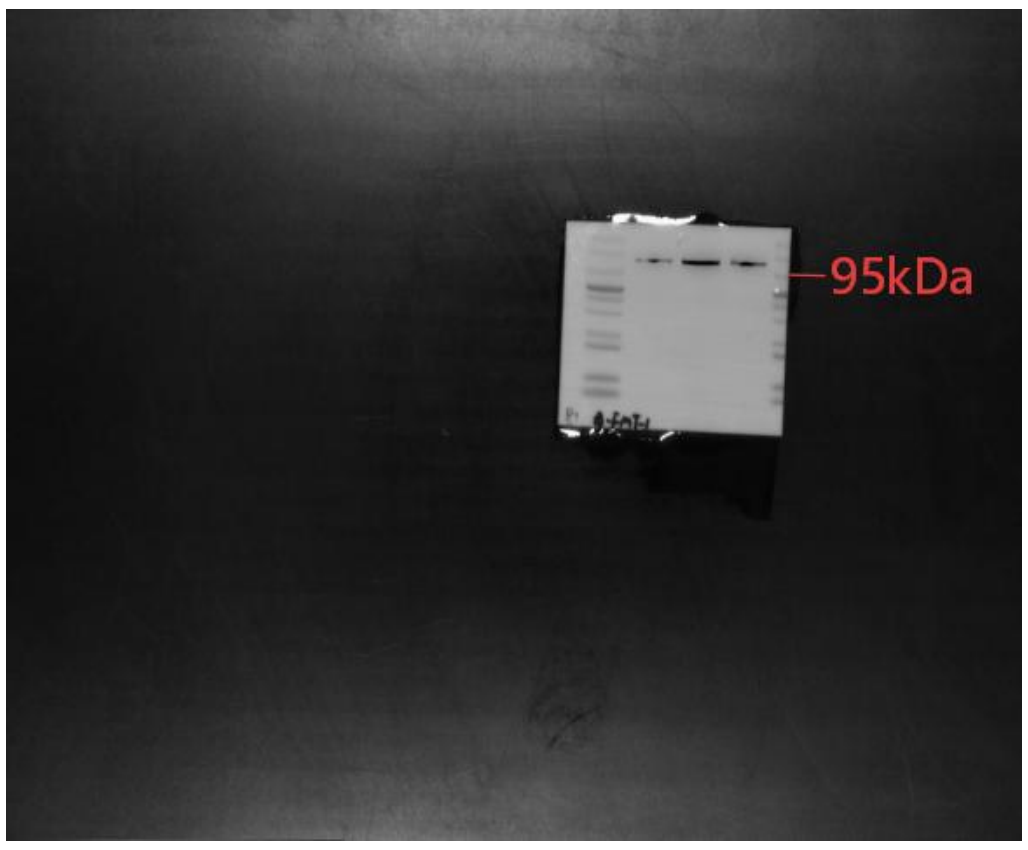

NLRP3

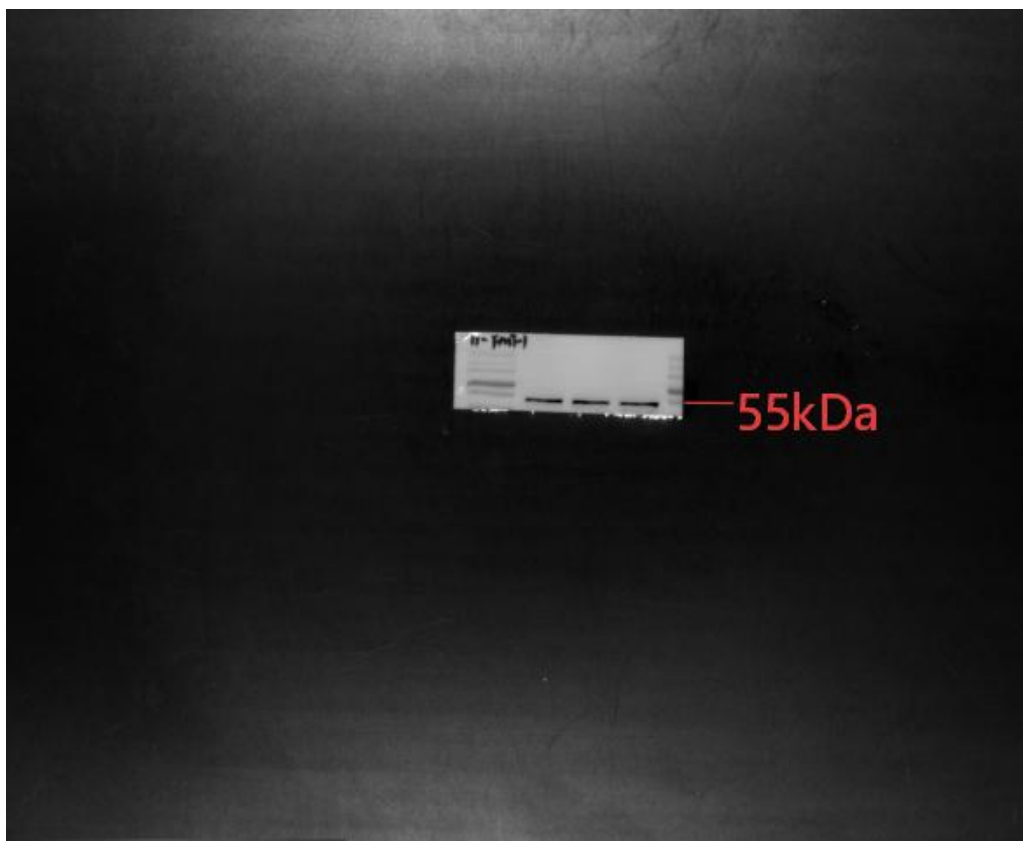

GSDMD

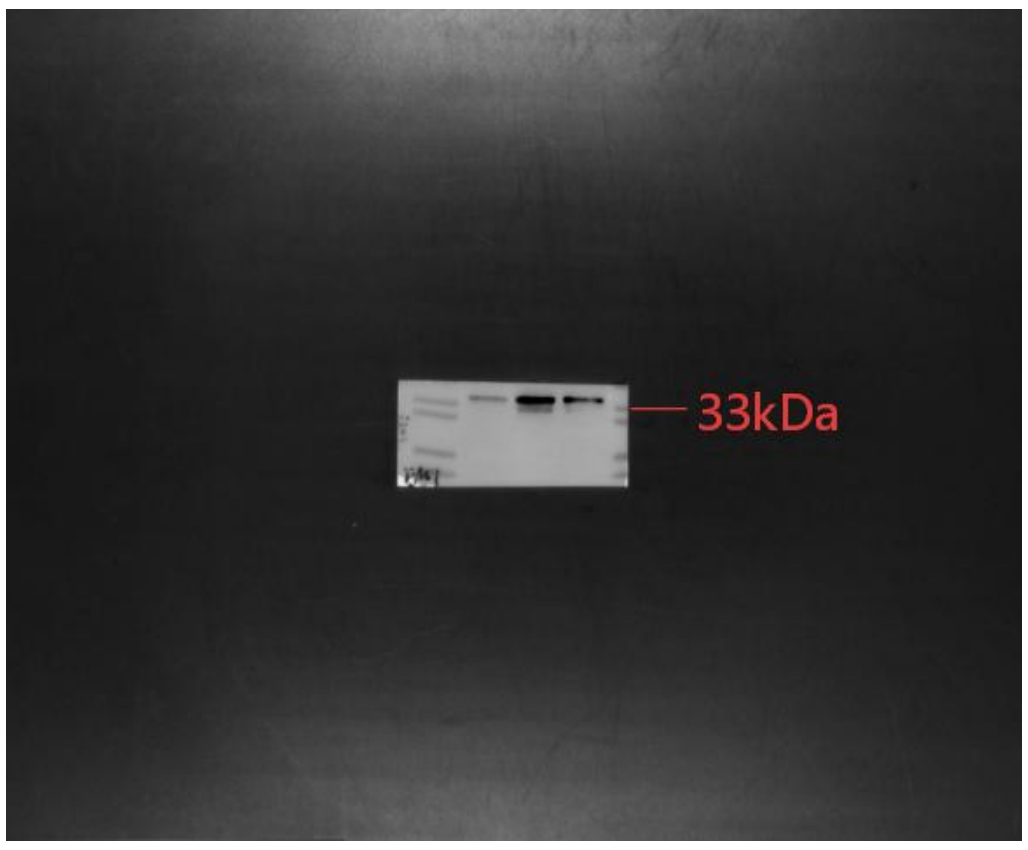

GSDMD-N

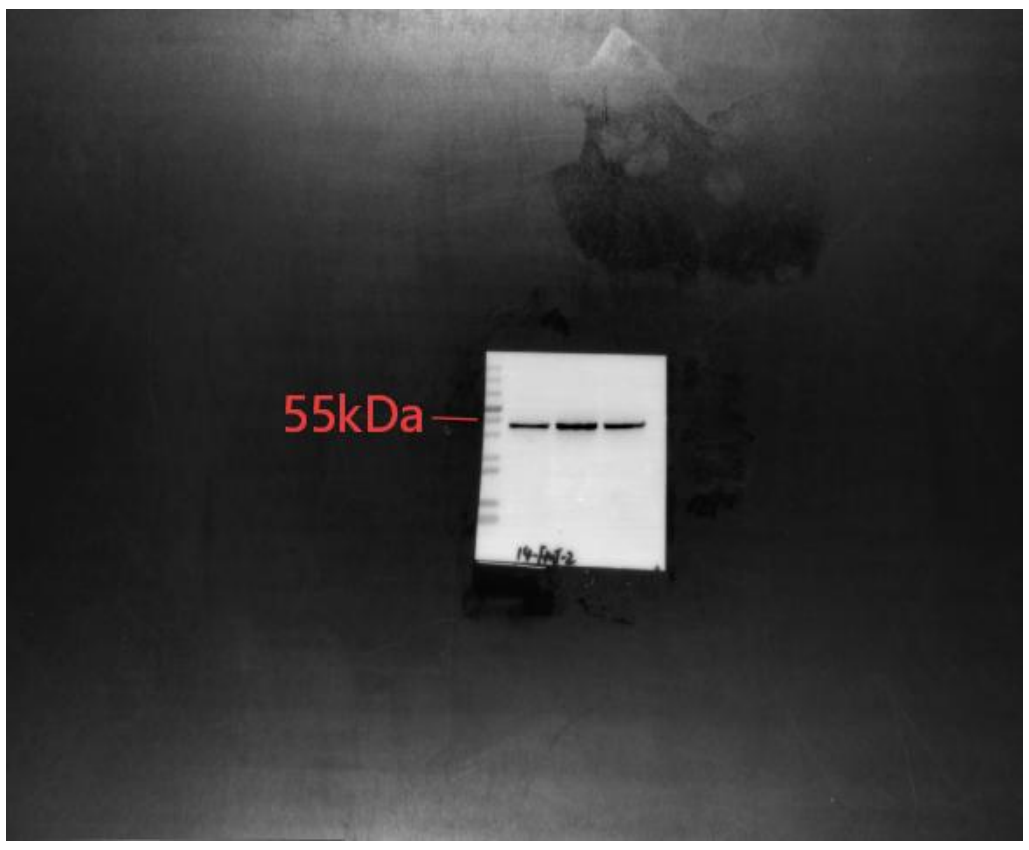

Caspase-1

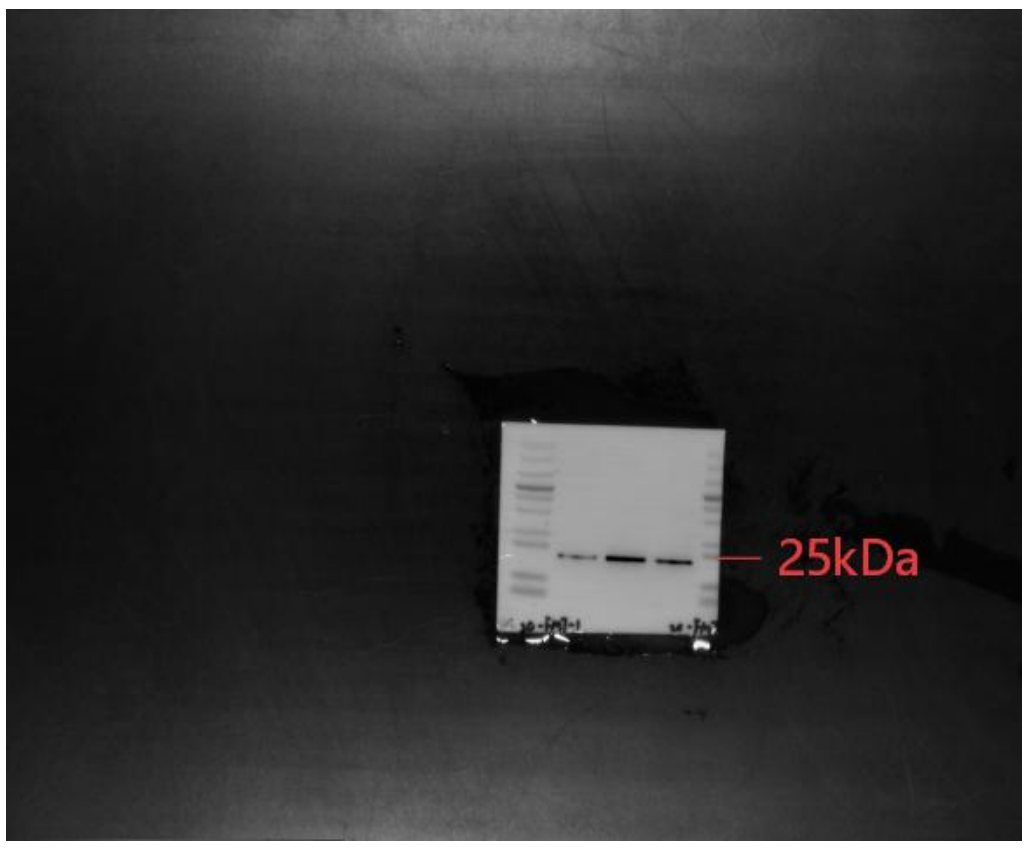

Cleaved Caspase-1

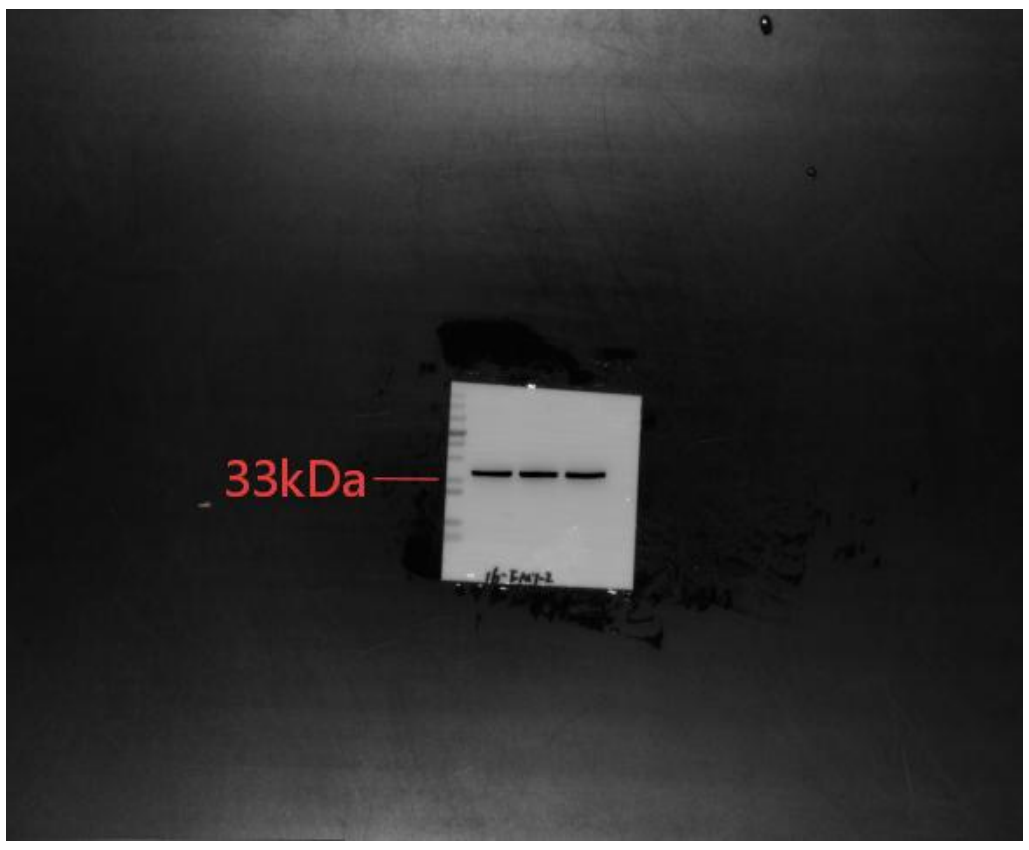

GAPDH

Mouse BMDMs

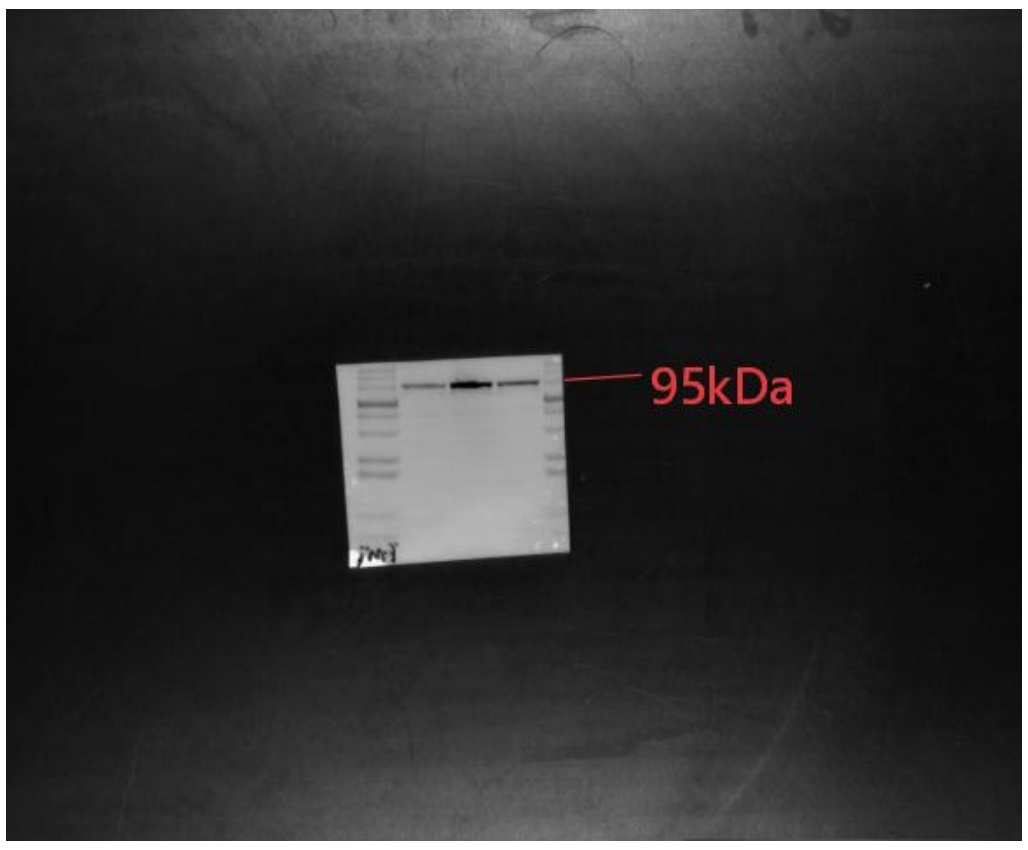

NLRP3

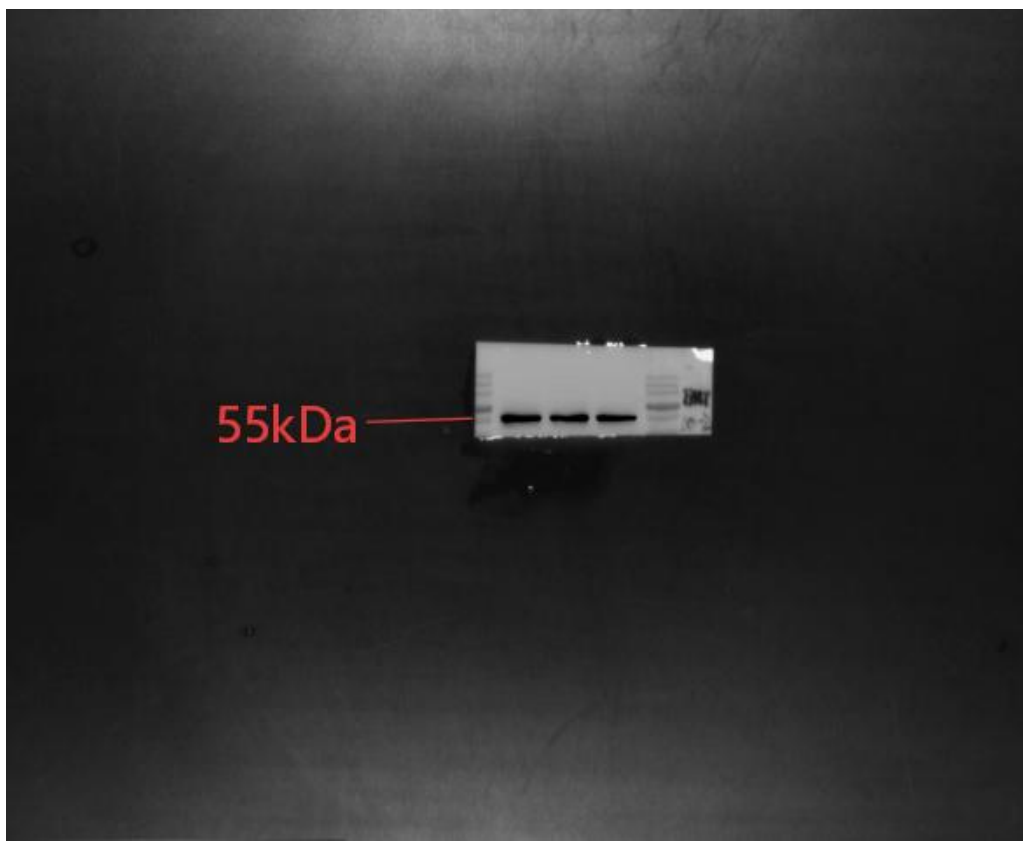

GSDMD

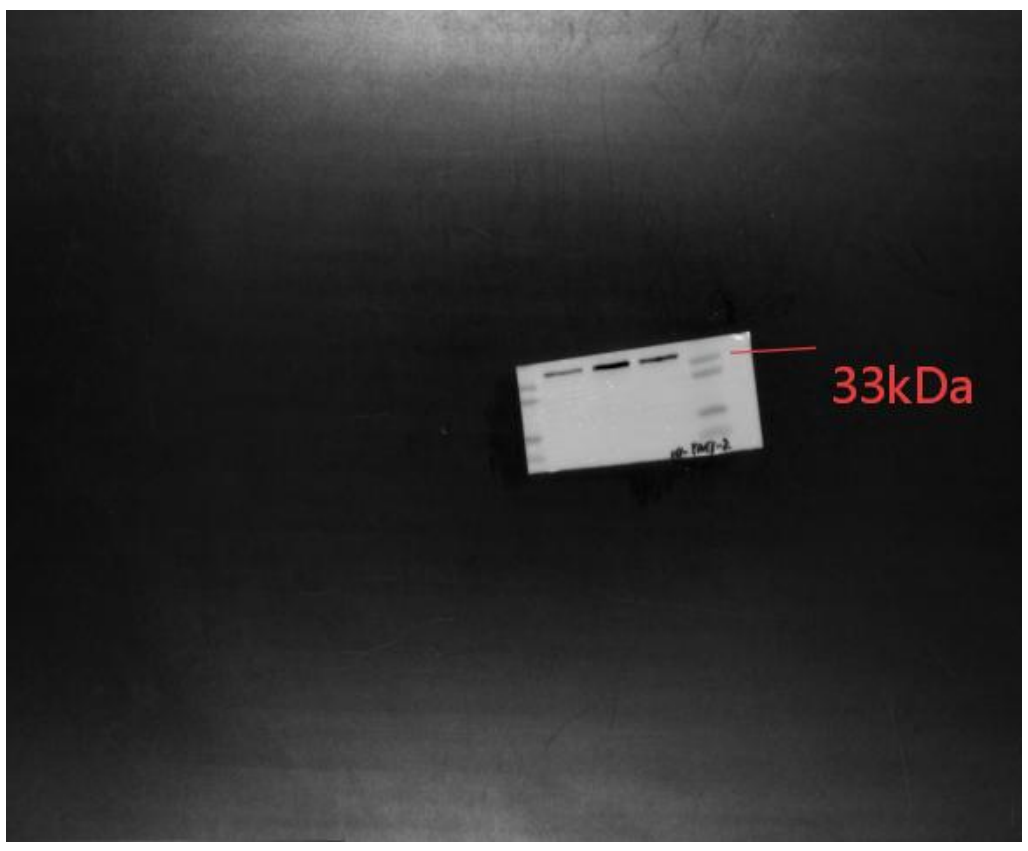

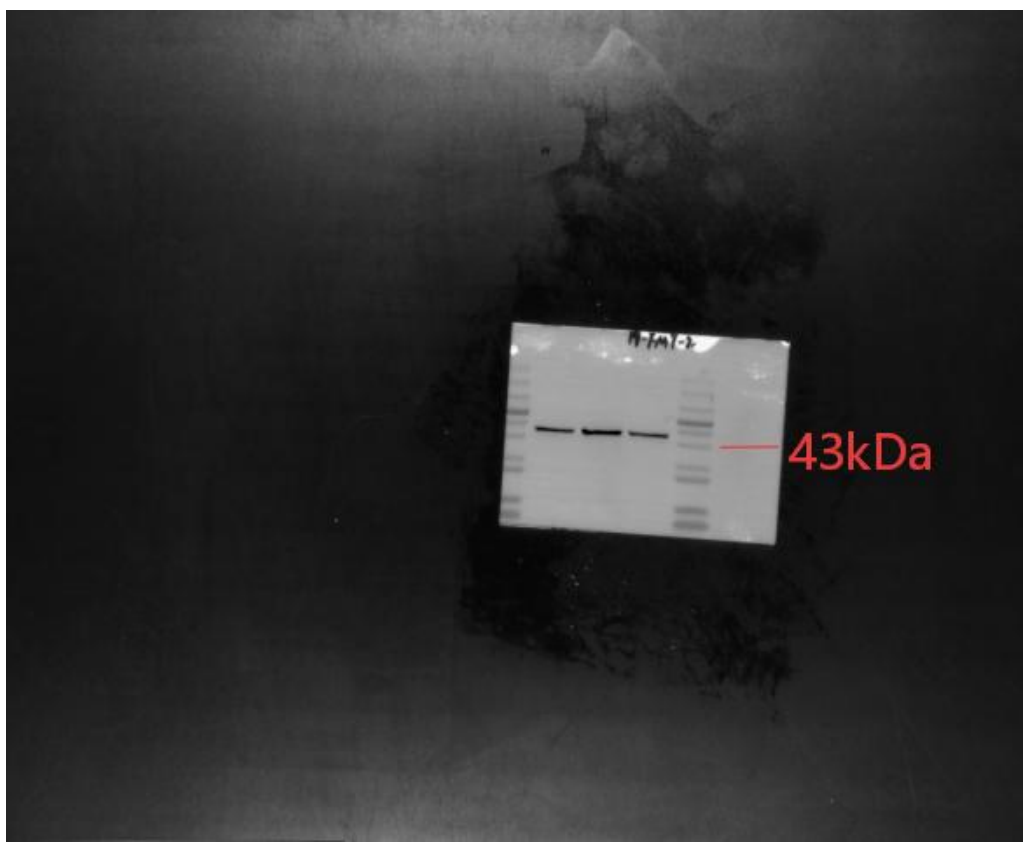

GSDMD-N

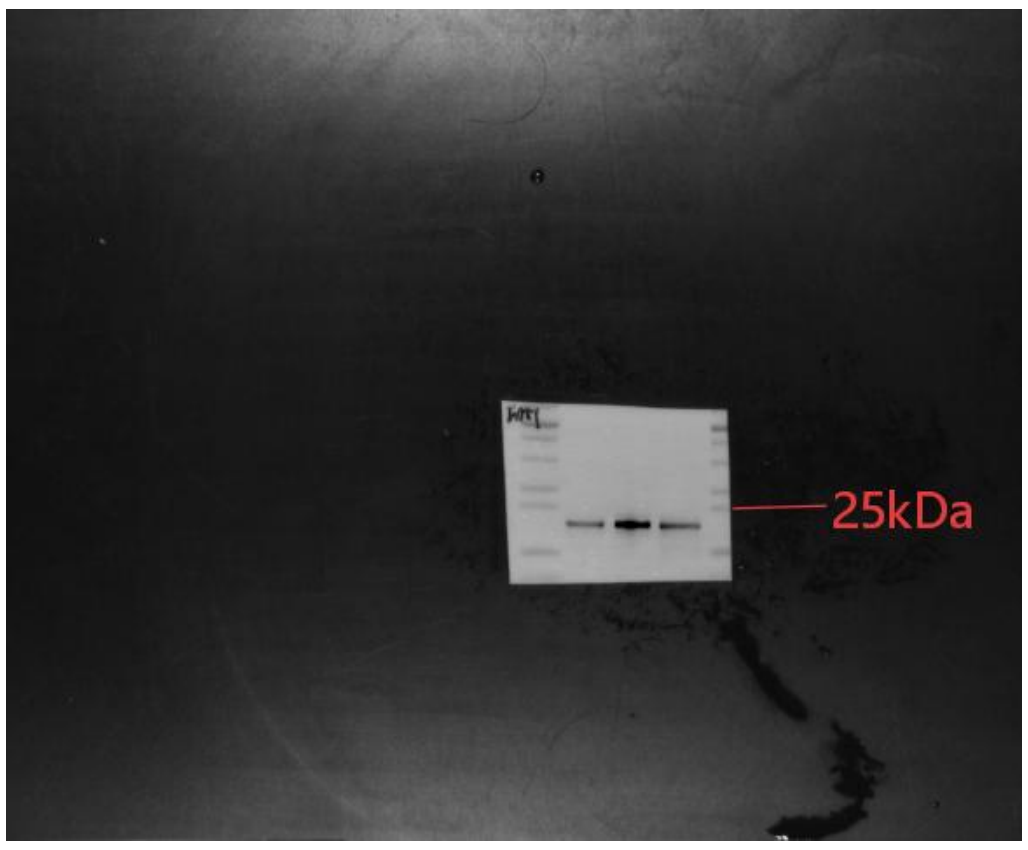

Cleaved Caspase-1

Caspase-1

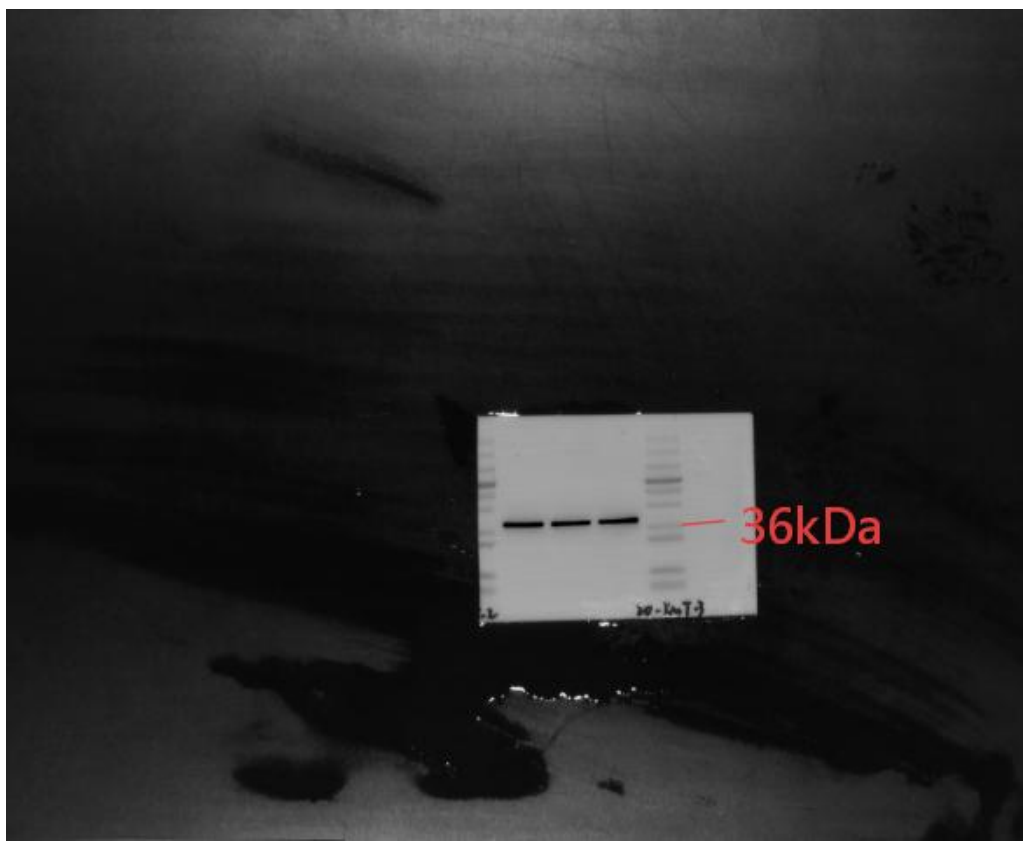

GAPDH
