# Supplementary material for: Upregulation of macrophage UPP1 promotes lung adenocarcinoma metastasis through an mtROS-cGAS-NLRP3 inflammasome axis
Source: Cell Death Discov. 2026 Jul 6;12:385. doi: 10.1038/s41420-026-03226-4 (PMC13620140; doi:10.1038/s41420-026-03226-4)
Supplement: Supplementary file 8 [file 41420_2026_3226_MOESM7_ESM.docx]

Supplementary Fig. S1 Validation of cell type annotation and characterization of UPP1 expression. (A) Dot plot validating unsupervised clustering and cell type annotation of the single-cell RNA-seq dataset using representative lineage-specific marker genes (e.g., GZMA/NKG7 for T&NK cells, CD163/CSF1R for myeloid cells, and EPCAM/KRT19 for epithelial cells). Dot size represents the percentage of cells expressing each gene, and color intensity indicates average expression levels.(B–D) Scatter plots showing modest but statistically significant correlations between UPP1 mRNA levels and tumor microenvironment-related features in the TCGA cohort (n = 513). UPP1 expression showed a negative correlation with inferred CD8+ T-cell abundance (B) and positive correlations with inferred M2 macrophage abundance (C) and PD-L1 (CD274) expression (D). Although the correlation coefficients are small, these associations provide hypothesis-generating evidence linking UPP1 expression to immune-related features. P-values and Pearson’s correlation coefficients (R) are indicated.(E) Hierarchical clustering heatmap of differentially expressed genes (DEGs) comparing parental/primary-like lung cancer cells with highly metastatic sublines. UPP1 is highlighted within a gene cluster enriched in the metastatic group.(F) Distribution plot showing UPP1 mRNA expression (FPKM) in metastatic versus primary-like lung cancer cell groups. UPP1 expression was higher in the metastatic group. P-value was determined by two-tailed Student’s t-test.

**Supplementary Fig. S2 Validation of UPP1 overexpression efficiency.** (A) Quantitative real-time PCR (qRT-PCR) analysis demonstrating a significant increase in UPP1 (human) and Upp1 (mouse) mRNA levels in THP-1 cells and mouse bone marrow-derived macrophages (BMDMs) following lentiviral transduction with UPP1-overexpression (UPP1-OE) constructs compared to Vector controls. mRNA levels were normalized to GAPDH.(B) Western blot analysis confirming the successful upregulation of UPP1 protein expression in both human THP-1 cells and mouse BMDMs. GAPDH served as an internal loading control to ensure equal protein loading. Molecular weights (kDa) are indicated to the right of the representative bands. Data are presented as mean ± SD from three independent biological replicates (n = 3). Statistical significance was determined using a two-tailed Student’s t-test. ***P < 0.001.

**Supplementary Fig. S3 Scavenging mitochondrial ROS attenuates metabolic alterations associated with UPP1 overexpression.**(A) Representative fluorescence images of mitochondrial superoxide accumulation detected by MitoSOX Red staining. Treatment with the mitochondria-targeted antioxidant MitoTEMPO (200 µM) effectively eliminated the UPP1-induced mtROS signal in both human THP-1 cells and mouse BMDMs. Nuclei were counterstained with DAPI (blue). Scale bar = 200 µm.(B–C) Quantitative rescue analysis of intracellular metabolic parameters. Scavenging mtROS with MitoTEMPO significantly restored the levels of PRPP (B) and the NAD+/NADH ratio (C) in UPP1-OE cells, suggesting that UPP1-mediated metabolic collapse is driven by mitochondrial oxidative stress.(D) qPCR analysis of cytosolic mitochondrial DNA (mtDNA) levels. The increase in cytosolic mtDNA (MT-CO1/mt-Co1) observed in UPP1-OE cells was attenuated following MitoTEMPO treatment, supporting a role for mtROS in mitochondrial stress and mtDNA release. Data are presented as mean ± SD from three independent biological replicates (n = 3). Statistical significance was determined by one-way ANOVA followed by Tukey’s post-hoc test. *P < 0.05, **P < 0.01, *P < 0.001.

**Supplementary Fig. S4 Exposure to UPP1-overexpressing macrophages is associated with increased EMT-related transcription factor expression in tumor cells.** (A) qRT-PCR analysis of EMT transcription factors (Snai1/SNAI1, Snai2/SNAI2, Zeb1/ZEB1) in LLC cells (left) and A549 cells (right) incubated with conditioned medium from Vector or UPP1-OE macrophages. Data are mean ± SD (n=3, ***P < 0.001).

**Supplementary Fig. S5 Genetic silencing of GSDMD attenuates UPP1-mediated tumor cell motility.** (A–B) Rescue experiments evaluating the dependency on GSDMD for macrophage-induced tumor cell motility. A549 and LLC cells were co-cultured with macrophages from four groups: Vector, UPP1-OE, Vector+shGSDMD, and UPP1-OE+shGSDMD. Representative images and quantification are shown for Transwell migration (A) and Matrigel-coated invasion (B) assays. Genetic knockdown of GSDMD in UPP1-OE macrophages significantly attenuated their ability to promote the migratory and invasive potential of co-cultured tumor cells. Scale bar = 500 µm.Data are presented as mean ± SD from three independent biological replicates (n = 3). Statistical significance was determined by one-way ANOVA followed by Tukey’s post-hoc test (*P < 0.05, **P < 0.01, ***P < 0.001).

**Supplementary Fig. S6 Neutralization of extracellular IL-1β attenuates inflammasome activation.** A Western blot analysis of NLRP3, GSDMD/GSDMD-N, and Pro-/Cleaved-Caspase-1 in UPP1-OE macrophages treated with IL-1β neutralizing antibody or IgG control, assessing the autocrine positive feedback loop.GAPDH served as the internal loading control. Molecular weights (kDa) are indicated to the right of the bands.
