# Supplementary material for: Upregulation of macrophage UPP1 promotes lung adenocarcinoma metastasis through an mtROS-cGAS-NLRP3 inflammasome axis
Source: Cell Death Discov. 2026 Jul 6;12:385. doi: 10.1038/s41420-026-03226-4 (PMC13620140; doi:10.1038/s41420-026-03226-4)
Supplement: Supplementary file 9 [file 41420_2026_3226_MOESM8_ESM.docx]

**Supplementary Table S1. List of antibodies used in this study.**

| **Target** | **Host Species** | **Dilution (WB)** | **Dilution (IF/IHC)** | **Catalog Number** | **Manufacturer** |
| --- | --- | --- | --- | --- | --- |
| UPP1 | Rabbit | 1:1000 | 1:200 | 14186-1-AP | Proteintech |
| NLRP3 | Rabbit | 1:1000 | 1:100 | #15101 | Cell Signaling Technology (CST) |
| Cleaved Caspase-1 (Asp297) | Rabbit | 1:1000 | - | #4199 | CST |
| Cleaved Caspase-1 (Asp296) | Rabbit | 1:1000 | - | #89332 | CST |
| GSDMD (Full/N-term) | Rabbit | 1:1000 | 1:200 | #96458 | CST |
| cGAS | Rabbit | 1:1000 | - | #15102 | CST |
| cGAS, mouse-specific | Rabbit | 1:1000 | - | #31659 | CST |
| STING | Rabbit | 1:1000 | - | #13647 | CST |
| P-STING(365), mouse | Rabbit | 1:1000 | - | #19781 | CST |
| P-STING(366), human | Rabbit | 1:1000 | - | #72971 | CST |
| p-TBK1 (Ser172) | Rabbit | 1:1000 | - | #5483 | CST |
| p-IRF3 (Ser396) | Rabbit | 1:1000 | 1:100 | #4947 | CST |
| E-Cadherin | Rabbit | 1:1000 | - | #3195 | CST |
| Vimentin | Rabbit | 1:1000 | 1:200 | #5741 | CST |
| N-Cadherin | Rabbit | 1:1000 | - | #13116 | CST |
| ASC / PYCARD, human | Rabbit | 1:1000 | - | #78000 | CST |
| ASC / PYCARD, mouse | Rabbit | 1:1000 | - | #67824 | CST |
| ZEB1 | Rabbit | 1:5000 | - | 85729-1-RR | Proteintech |
| SNAI2 / Slug | Rabbit | 1:1000 | - | #9585 | CST |
| SNAI1 / Snail | Rabbit | 1:1000 | - | #3879 | CST |
| CD68 | Mouse | - | 1:200 | ab955 | Abcam |
| F4/80 | Rat | - | 1:100 | ab6640 | Abcam |
| Ki67 | Rabbit | - | 1:400 | #9027 | CST |
| Lamin B1 | Rabbit | 1:1000 | - | #9087 | CST |
| *β*-Actin | Mouse | 1:5000 | - | #3700 | CST |
| GAPDH | Rabbit | 1:5000 | - | #2118 | CST |

*Secondary antibodies:* HRP-linked anti-rabbit IgG (#7074, CST) and anti-mouse IgG (#7076, CST) were used at 1:3000 for WB. Alexa Fluor 488/594-conjugated secondary antibodies (Thermo Fisher Scientific) were used at 1:500 for IF.

**Supplementary Table S2. Target sequences of shRNAs used for gene knockdown.**

| **Target Gene** | **Species** | **Construct Name** | **Target Sequence (5' -> 3')** |
| --- | --- | --- | --- |
| NLRP3 | Human | shNLRP3 #1 | CCGTAAGAAGTACAGAAAGTA |
|  |  | shNLRP3 #2 | CCAGCCAGAGTCTAACTGAAT |
| GSDMD | Human | shGSDMD #1 | GTGTGTCAACCTGTCTATCAA |
|  |  | shGSDMD #2 | CAGCACCTCAATGAATGTGTA |
| Nlrp3 | Mouse | shNlrp3 #1 | CCGGCCTTACTTCAATCTGTT |
|  |  | shNlrp3 #2 | CCAGGAGAGAACCTCTTATTT |
| Gsdmd | Mouse | shGsdmd #1 | CCTGTTCCTATTGTCAAGTCT |
|  |  | shGsdmd #2 | GCCTCCATGAATGTGTGTATA |
| Negative Control | N/A | shNC (Scramble) | TTCTCCGAACGTGTCACGT |

**Supplementary Table S3. Sequences of primers used for qRT-PCR.**

| **Species** | **Gene Symbol** | **Forward Primer Sequence (5' -> 3')** | **Reverse Primer Sequence (5' -> 3')** |
| --- | --- | --- | --- |
| Human | *UPP1* | TCATCCGCATTGGCACTTCT | GCAGAACACAGCAACAGCTC |
| Human | *NLRP3* | CCACAAGATCGTGAGAAAACCC | CGGTCCTATGTGCTCGTCA |
| Human | *SNAI1* (Snai1) | TCGGAAGCCTAACTACAGCGA | AGATGAGCATTGGCAGCGAG |
| Human | *SNAI2* (Slug) | CGAACTGGACACACATACAGTG | CTGAGGATCTCTGGTTGTGGT |
| Human | *ZEB1* | TTACACCTTTGCATACAGAACCC | TTTACGATTACACCCAGACTGC |
| Human | *MT-CO1* (mtDNA) | GCCCACTTCCACTATGTCCT | TGGCGTAGGTTTGGTCTAGG |
| Mouse | *Gapdh* | CAAAAGGGTCATCATCTCC | CTGACAATCTTGAGTGAGTT |
| Human | *GAPDH* | TCCATGACAACTTTGGTATC | CAGGGATGATGTTCTGGA |
| Mouse | *Upp1* | ACAGGAACTGAAGCAAAGGAC | GTTGAAATGGTAGAGCACGTCTT |
| Mouse | *Nlrp3* | GAATCAGATTGCTGTGTGTG | ATGTACAGTCTGGTCAGAGA |
| Mouse | *Snai1* (Snai1) | CACACGCTGCCTTGTGTCT | GGTCAGCAAAAGCACGGTT |
| Mouse | *Zeb1* | ACTGCAAGAAACGGTTTTCCC | GGCGAGGAACACTGAGATGT |
| Mouse | *Snai2* (Slug) | CAGCGAACTGGACACACACA | ATAGGGCTGTATGCTCCCGAG |
| Mouse | *mt-Co1* (mtDNA) | AACCCCCAGCCATAACACAG | CTCCTCCAGCGGGATCAAAG |
